# Construction of Heteroleptic Copper Complexes in Perylene Diimide-Based COFs for Heterogeneous Metallaphotoredox Catalysis

**DOI:** 10.1021/jacs.5c17585

**Published:** 2026-01-23

**Authors:** Xia Wu, Jun Guo, Meng-Ying Sun, Tao Du, Debo Hao, Dongyi Liu, Deyang Wang, Songwei Wen, Jun Yin, Dan Li, Jian He

**Affiliations:** † Department of Chemistry, 25809 The University of Hong Kong, Hong Kong 999077, China; ‡ State Key Laboratory of Synthetic Chemistry and Shanghai-Hong Kong Joint Laboratory in Chemical Synthesis, The University of Hong Kong, Hong Kong 999077, China; § Materials Innovation Institute for Life Sciences and Energy (MILES), HKU-SIRI, Shenzhen 518048, China; ∥ Department of Applied Physics, 26680 The Hong Kong Polytechnic University, Hung Hom, Kowloon 999077, China; ⊥ Guangdong Provincial Key Laboratory of Supramolecular Coordination Chemistry, 47885 Jinan University, Guangzhou 510632, China

## Abstract

Despite recent advances in developing diimine-containing copper photocatalysts for organic synthesis, further exploration of heteroleptic phenanthroline-ligated copper complexes remains challenging due to rapid ligand exchange in homogeneous solutions. Herein, we employ a framework-based heterogenization strategy to successfully synthesize such previously inaccessible copper complexes through postsynthetic modification. By integrating photoactive perylene diimide units into a one-dimensional covalent organic framework, the visible-light-driven oxo-azidation of styrenes can be accomplished with substantially reduced copper loadings in metallaphotoredox catalysis. Notably, our newly developed heterogeneous photocatalytic platform demonstrates that the reactive copper species facilitates the ketone formation step in the oxo-azidation without dissociation of one of the phenanthroline ligands. These findings highlight the potential of well-defined framework materials to address fundamental challenges in the synthesis of transition-metal complexes and enable detailed mechanistic studies that complement traditional homogeneous catalysis.

## Graphical abstract

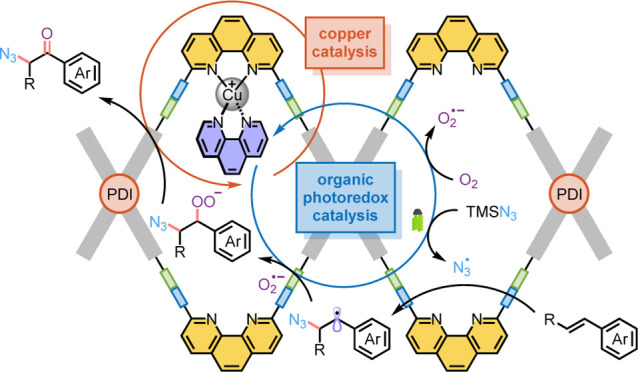

## Introduction

In recent years, the development of efficient transition-metal catalysts utilizing the synergistic and confinement effects provided by framework-based supports has emerged as a promising approach to advancing synthetic methodologies.^1–8^ Beyond enhancing catalyst stability and recyclability, the heterogenization of transition-metal species within crystalline porous frameworks alters their photophysical properties^9^ and offers unique binding modes^10–12^ that are difficult to achieve in homogeneous solutions. The generation of novel transition-metal complexes embedded in framework matrices, combined with their highly tunable coordination environments,^13–17^ opens new opportunities for establishing more effective catalytic platforms and delving deeper insights into reaction mechanisms. Given the importance of sustainability and practicality in catalysis, as well as the challenges associated with improving the reactivity of first-row transition metals in conventional homogeneous systems,^18–20^ developing robust heterogeneous catalysts^21–25^ based on earth-abundant elements for organic synthesis^26,27^ is highly desirable.

Owing to their exceptional single-electron transfer (SET) reactivity, copper-based catalysts have found extensive applications in radical-mediated processes under both thermal and photochemical conditions.^28–32^ For instance, the pioneering work by Sauvage and co-workers on a homoleptic [Cu­(dap)_2_]^+^ complex (where dap is 2,9-di­(4-anisyl)-1,10-phenanthroline)^33^ has enabled a diverse range of atom transfer radical addition (ATRA) reactions over the past decade,^34–38^ with significant contributions from the Reiser group.^39–42^ Introducing aryl substituents to the phenanthroline (phen) ligand in the Sauvage catalyst enhances photoactivity and stability through increased steric hindrance and π–π stacking interactions (Scheme 1a.^43,44^ However, it remains important to explore alternative phen-ligated copper complexes to further improve catalyst turnover numbers (TONs). We postulated that selective removal of the substituents from one of the two phen-based ligands could reduce steric congestion around the copper center, thereby improving substrate accessibility during redox processes while preserving weak π–π stacking interactions (Scheme 1b). Such targeted ligand modification might produce more reactive copper species for photocatalytic reactions. Because 2,9-disubstituted and simple phen ligands have similar binding affinities, adding them to a homogeneous solution of cationic copper­(I) consistently yields a mixture of homoleptic and heteroleptic phen-ligated complexes.^45^ Given the rapid ligand exchange, the equilibria rarely favor copper species coordinated with two different phen-based ligands (Scheme 1c).

**Scheme 1:**
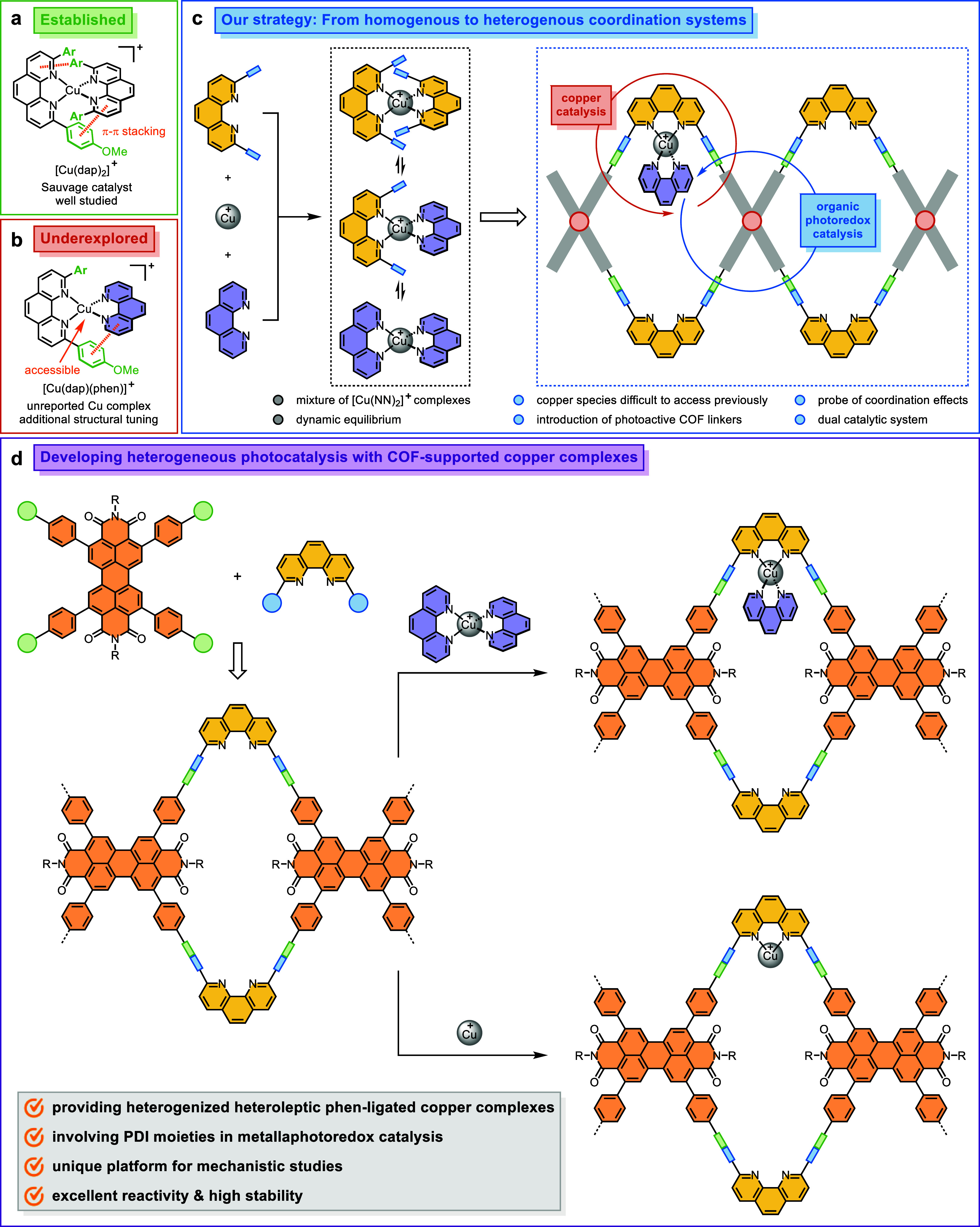
Design of Stable Heteroleptic Phen-Ligated Copper Complexes for Heterogeneous Photocatalysis

In order to suppress the formation of homoleptic phen-ligated copper­(I) complexes via bimolecular pathways, we employ a strategy that incorporates 2,9-disubstituted phen moieties into a covalent organic framework (COF) solid support.^46–50^ In this system, where copper active centers are effectively isolated, the dissociation of either a monophen-ligated copper­(I) cation or a phen ligand from the immobilized heteroleptic complex is thermodynamically disfavored. Furthermore, restricted mass transfer within the COF pores helps stabilize the targeted complex by facilitating recombination after dissociation. As a result, the heterogeneous coordination environment effectively prevents the generation of the homoleptic [Cu­(phen)_2_]^+^ complex, addressing an unresolved challenge present in comparable homogeneous systems. Due to the heterogenization of the phen-based ligand, a monophen-ligated copper complex can also form exclusively on the functional framework, which provides a well-defined platform to study ligand coordination effects in catalysis. Beyond accommodating phen moieties, crystalline COF materials can be tailored through presynthetic modification strategies to promote electron transfer under light irradiation.^51^ By judiciously selecting the photoactive organic linkers, it is possible to design heterogeneous dual catalytic systems that merge copper catalysis with organic photoredox catalysis (Scheme 1c).

In this article, we present the synthesis of one-dimensional (1D) perylene diimide (PDI)-based COFs functionalized with phen moieties. Postsynthetic metalation with [Cu­(phen)_2_]^+^ and cationic copper­(I) yields COF-supported heteroleptic phen-ligated and monophen-ligated complexes, respectively (Scheme 1d). The integration of photoactive PDI units within the porous framework enables efficient generation of azide radicals upon photoexcitation,^52^ thereby facilitating the oxo-azidation of styrenes using trimethylsilyl azide (TMSN_3_) and O_2_. Mechanistic studies provide new insights indicating that the heteroleptic phen-ligated copper complex, rather than the monophen-ligated counterpart, is the primary reactive species responsible for promoting ketone formation in the photocatalytic reaction. Through rational COF structure design, the heterogeneous copper catalyst outperforms its homogeneous analogues and can be recycled multiple times with minimal reactivity loss.

## Results and Discussion

Given the superior photoredox activity of PDI over pyrene units in nucleophile activation for ATRA,^52–55^ we incorporated four aniline connecting moieties into the PDI core to create a tetra-amino-substituted organic linker (**PDI–NH**_**2**_).^56^ Drawing inspiration from the design of the Sauvage complex, we introduced two formyl groups in place of methoxy groups on the phen-based ligand to construct imine-linked COFs. Accordingly, we synthesized both 2,9-di­(4-formylphenyl)-1,10-phenanthroline (**dpp–CHO**) and its phenyl-free analogue (**phen–CHO**), which were then employed to prepare the target PDI-based 1D framework materials (**1D-PDI–dpp** and **1D-PDI–phen**, respectively) under solvothermal conditions using different solvent combinations (Scheme 2). To ensure the formation of heteroleptic phen-ligated copper­(I) complexes on the COF supports, we performed postsynthetic metalation by adding an acetonitrile solution of [Cu­(phen)_2_]­(PF_6_) at room temperature. During immobilization on **1D-PDI–dpp** and **1D-PDI–phen**, one phen ligand dissociated from the copper center, resulting in heterogeneous heteroleptic copper photocatalysts (i.e., **1D-PDI–dpp­(phen)­Cu** and **1D-PDI–phen­(phen)­Cu**). Similarly, the corresponding catalysts containing monophen-ligated copper species, namely **1D-PDI–dppCu** and **1D-PDI–phenCu**, were prepared by treating the COF supports with [Cu­(MeCN)_4_]­(PF_6_). Due to its smaller pore size, the copper loadings in **1D-PDI–phen** were lower than those in **1D-PDI–dpp**. Moreover, the incorporation of the relatively bulky heteroleptic phen-ligated copper complex in **1D-PDI–phen** reduced its crystallinity, which could potentially lead to metal leaching during catalysis (Figure S1). Similarly, replacing [Cu­(phen)_2_]­(PF_6_) with more sterically hindered [Cu­(dmp)_2_]­(PF_6_) (where dmp is 2,9-dimethyl-1,10-phenanthroline) resulted in dramatic decrease in metalation efficiency (Table S1). Therefore, we primarily focused on **1D-PDI–dpp­(phen)­Cu** for detailed characterization and photocatalytic investigations.

**Scheme 2:**
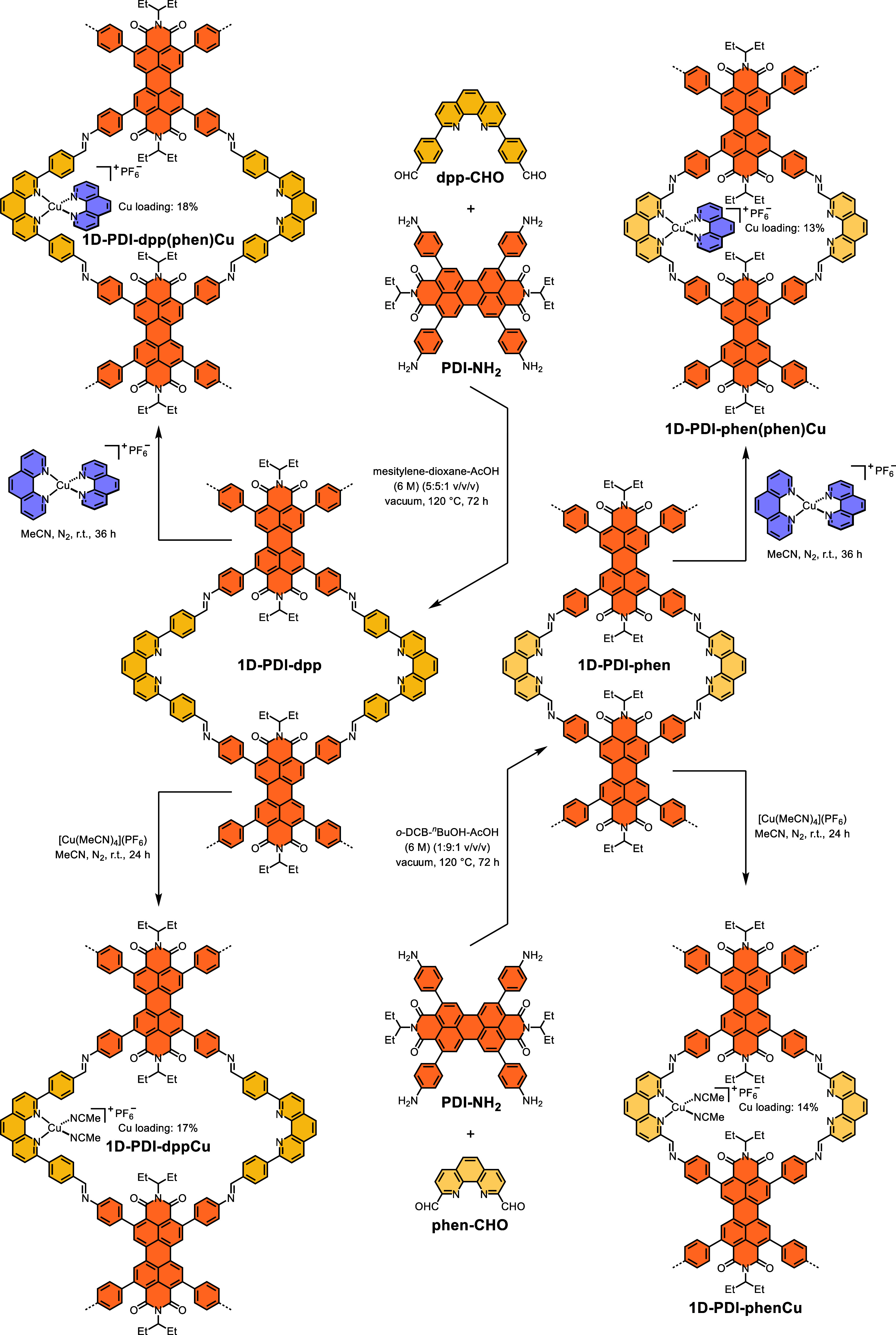
Schematic Representation of Catalyst Synthesis

In addition to employing FT-IR and solid-state ^13^C NMR to analyze the chemical composition of **1D-PDI–dpp** (Figures S2 and S3), we examined its crystallinity using powder X-ray diffraction (PXRD) techniques (Figure 1a). The PXRD patterns displayed a prominent peak at 5.51°, along with two relatively weak peaks around 10°, corresponding to the (110), (020), and (210) reflection planes in a distinctive AB-slipped stacking mode. The Pawley-refined PXRD profiles showed excellent agreement with the experimental data, providing *R*_wp_ and *R*_p_ values of 3.76% and 2.83%, respectively. Similar to other stacking modes, the extended planes formed by the 1D chains adopt a parallel arrangement. However, to achieve overlap between the nearest planes (highlighted in blue or pink) following a perpendicular shift, a slip operation, vertical to the chain with a displacement (*d*) of 6.73 Å, is necessary (Figure 1b). The closest chains on two neighboring planes are offset by half a repeating unit along the 1D chain direction, same as those simulated in the COF structure of the AB-staggered stacking mode (Figure 1c). Notably, both the AB-staggered and AA-eclipsed modes show alternating adjacent chains within the same plane (Figures 1c and S4), giving small interstitial pores that are inconsistent with the PXRD results. In contrast, the simulated structure with the AB-slipped stacking features chains arranged in parallel, with an interchain separation (*s*) of 4.29 Å, which corresponds to the value of *d* minus the distance between two parallel C–C bonds in a benzene ring (Figure 1b). This dramatic change in chain assembly within the extended plane is most likely driven by the strong π–π stacking interactions between the PDI core and the phen moieties from the upper and lower COF layers (Figure S5). Unlike 1D COFs built from pyrene-based organic linkers,^57–59^ the PDI units in **1D-PDI–dpp** experience greater dipole repulsion from the diimide motifs and steric hindrance from the *N*-alkyl substituents,^60^ both of which disfavor the AA-eclipsed stacking mode (Figure S4). While both the AB-slipped and AB-staggered stacking modes alleviate these repulsive interactions, the additional stabilization from the π–π stacking results in **1D-PDI–dpp** adopting a triclinic rather than an orthorhombic crystal system (Figure S5a and Table S2).

**Figure 1:**
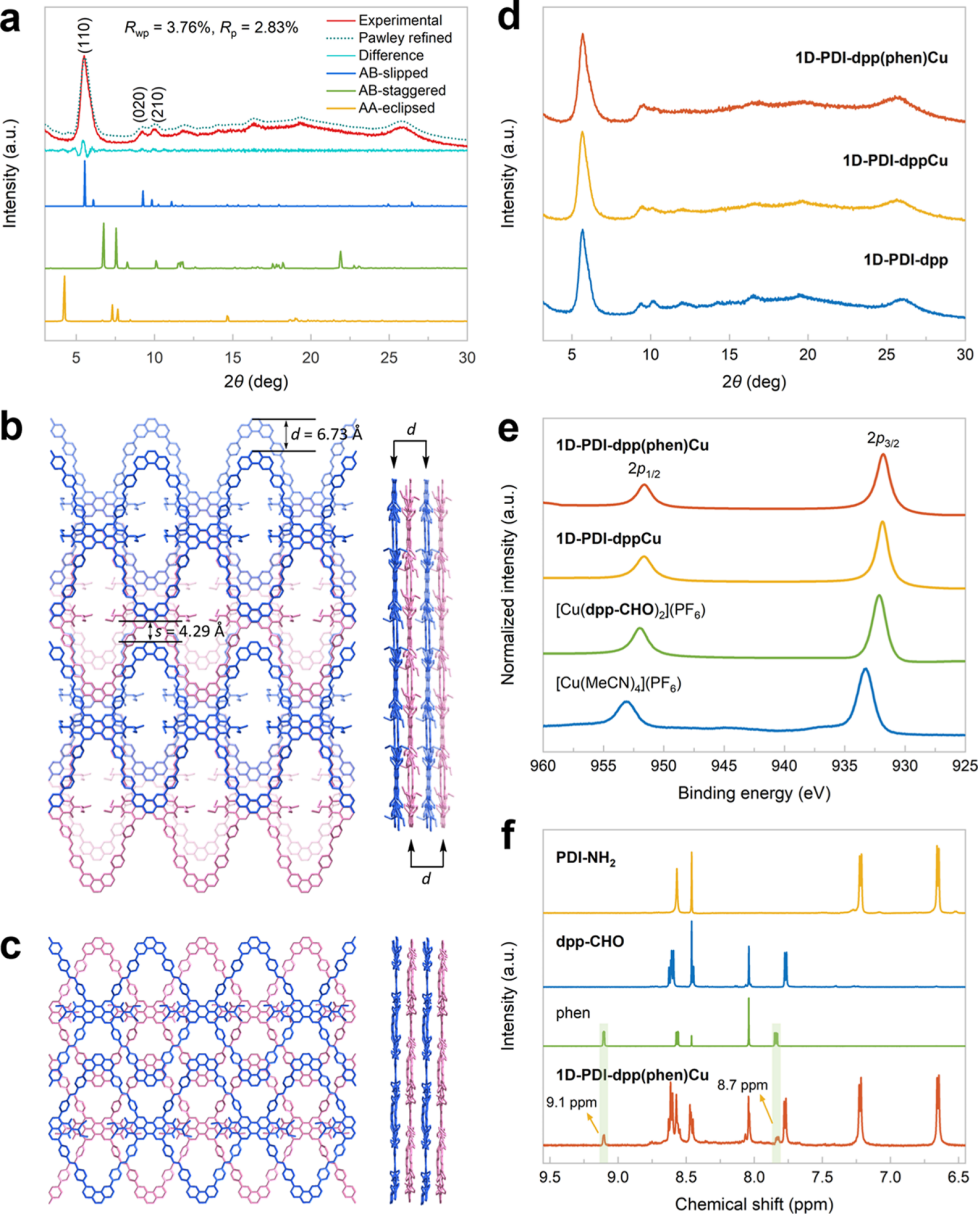
(a) PXRD patterns and refined modeling profiles of **1D-PDI–dpp**. (b) Simulated framework structure of the AB-slipped stacking mode viewed from the extended plane and along the 1D chain direction. (c) Simulated framework structure of the AB-staggered stacking mode. (d) Comparing PXRD patterns before and after postsynthetic metalation. (e) Cu2*p* XPS spectra showing the +1 oxidation state of the Cu centers in **1D-PDI–dpp­(phen)­Cu** and **1D-PDI–dppCu**. (f) NMR analysis of the digested sample of **1D-PDI–dpp­(phen)­Cu**.

Scanning electron microscopy analysis showed that the morphology of **1D-PDI–dpp** remained intact after postsynthetic metalation with [Cu­(phen)_2_]­(PF_6_) or [Cu­(MeCN)_4_]­(PF_6_) (Figures S6–S8). The crystallinity was also preserved, as indicated by the PXRD patterns (Figure 1d). Furthermore, energy-dispersive X-ray spectroscopy mapping revealed a homogeneous distribution of the elements C, N, O, and Cu throughout the framework, which confirmed the consistent composition of both **1D-PDI–dpp­(phen)­Cu** and **1D-PDI–phen­(phen)­Cu** (Figures S11 and S12). The Cu2*p*_1/2_ and Cu2*p*_3/2_ peaks observed in the X-ray photoelectron spectroscopy (XPS) spectra of the COF-supported photocatalysts can be attributed to copper­(I) species (Figure 1e). The shifts toward lower binding energies are due to the coordination of phenanthroline ligands to the copper center.^9^ It is noteworthy that XPS analysis revealed mixed-valence states of copper in [Cu­(**dpp–CHO**)­(MeCN)_2_]­(PF_6_) (Figure S13), confirming that the COF support effectively prevents the oxidation of monophen-ligated copper­(I) species in air (Figure 1e). The digestion of **1D-PDI–dpp­(phen)­Cu** with trifluoroacetic acid, followed by the addition of sodium cyanide, produced characteristic peaks of free phen as well as signals from the organic linkers (Figure 1f). The integration of the phen ligand closely correlated with the copper content in the COF-supported catalyst, suggesting the successful formation of the targeted heteroleptic phen-ligated copper complex within the framework pores (Figure S16 and Table S3).

To efficiently produce α-azido ketones, an important class of versatile synthetic intermediates,^61^ via heterogeneous photocatalysis, we employed **1D-PDI–dpp­(phen)­Cu** as the catalyst to explore the oxo-azidation of simple styrene with TMSN_3_ in air under light irradiation (Table S4).^40^ Following a systematic evaluation of light wavelengths and solvents, we established that a robust photocatalytic system could deliver 2-azido-1-phenylethanone (**1**) in 78% yield using just 0.1 mol % of **1D-PDI–dpp­(phen)­Cu** and green LED lamps as the light source (Table 1, entry 1). Control experiments demonstrated that the oxo-azidation reaction did not proceed in the absence of catalyst, light, or air (Table S4, entries 2–5). Moreover, no styrene was consumed under the irradiation conditions without **1D-PDI–dpp­(phen)­Cu**, indicating that benzaldehyde side product was generated photocatalytically. Notably, when the phen ligand was removed from the immobilized copper active center, the yield of product **1** decreased to 32%, while a comparable amount of peroxide **1-a** was obtained (Table 1, entry 2). In the meantime, the overall conversion of styrene declined to 81%. Using the parent framework support (i.e., **1D-PDI–dpp**) as the photocatalyst, despite achieving high substrate conversion, the desired product yield further dropped to 20% (Table 1, entry 3). Extending the reaction time did not promote the conversion of **1-a** to **1** (Table 1, entry 4), which demonstrates the crucial role of the copper active center in facilitating the oxo-azidation of styrene. The PDI or phen linker alone exhibited fairly low activity, underscoring the importance of framework construction in this photocatalytic system (Table 1, entries 5 and 6). It is worth noting that the employment of **1D-PDI–phen** as the solid support reduced both the product yield and overall conversion (Table 1, entries 7 and 8). Recycled **1D-PDI–phen­(phen)­Cu**, which showed diminished PXRD signals consistent with the trend observed in freshly prepared **1D-PDI–phen**-based catalysts via postsynthetic metalation (Figure S1), further decreased the yield of product **1** to 17% in the second run (Table S8 and Figure S34). This indicates that **1D-PDI–phen** is not a suitable matrix for hosting heteroleptic phen-ligated copper complexes in photocatalytic applications. Nevertheless, their performance remained superior to that of copper catalysts embedded in well-established pyrene-based COFs^62–64^ (Table 1, entries 9–12), demonstrating the benefits of incorporating photoactive PDI units into catalyst design. Very importantly, across all four framework supports tested, the heteroleptic phen-ligated copper complexes consistently outperformed their monophen-ligated counterparts (Table 1, entries 1, 2, and 7–12). Regarding the survey of homogeneous copper photocatalysis, while conventional Sauvage-type catalysts showed low reactivity at a copper loading of 0.1 mol % (Table 1, entries 13 and 14), mixing the simple phen ligand with **dpp–CHO** in a 1:1 ratio in the presence of [Cu­(MeCN)_4_]­(PF_6_) markedly increased the yields of compounds **1** and **1-a** (Table 1, entry 15). Such findings provide strong evidence that heteroleptic phen-ligated copper complexes can function as promising catalysts for photoinduced oxo-azidation reactions. In addition, phosphine-based monomeric and dimeric copper photocatalysts proved to be ineffective and were excluded from the heterogenization approach (Table 1, entries 16–18).^45,65,66^

**Table 1:**
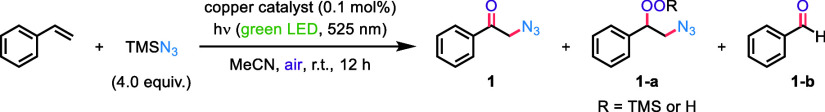
Effect of Different Copper Photocatalytic Systems on the Oxo-Azidation of Styrene^a^

entry	catalyst	yield of **1** (%)	yield of **1-a** (%)	yield of **1-b** (%)	conversion of styrene (%)
1	**1D-PDI–dpp(phen)Cu**	78	<5	21	>95
2	**1D-PDI–dppCu**	32	31	9	81
3^b^	**1D-PDI–dpp**	20	40	17	95
4^b^ ^,^ ^c^	**1D-PDI–dpp**	23	36	19	>95
5^b^	**PDI–NH_2_**	<5	<5	<5	5
6^b^	**dpp–CHO**	<5	<5	<5	10
7	**1D-PDI–phen(phen)Cu**	34	7	8	50
8	**1D-PDI–phenCu**	21	11	<5	39
9	**1D-Py–dpp(phen)Cu**	17	27	8	64
10	**1D-Py–dppCu**	8	13	7	30
11	**1D-Py–phen(phen)Cu**	12	22	8	52
12	**1D-Py–phenCu**	7	8	<5	26
13	[Cu(phen)_2_](PF_6_)	<5	6	<5	14
14	[Cu(**dpp–CHO**)_2_](PF_6_)	<5	10	<5	16
15	[Cu(**L**)_2_](PF_6_)	12	36	6	52
16	[Cu(phen)(xantphos)](PF_6_)	<5	<5	<5	<5
17	[Cu(phen)(binap)](PF_6_)	<5	15	<5	23
18	(binap)_2_Cu_2_I_2_	6	13	<5	21

a Reaction conditions: styrene (0.10 mmol, 1.0 equiv), TMSN_3_ (4.0 equiv), and copper catalyst (0.1 mol %) in acetonitrile (0.4 mL) under air atmosphere at room temperature with green-LED light irradiation (525 nm) for 12 h. Xantphos, (9,9-dimethyl-9*H*-xanthene-4,5-diyl) bis­(diphenylphosphane); binap, 2,2′-bis­(diphenylphosphino)-1,1′-binaphthyl. Yield and conversion were determined by ^1^H NMR of the crude reaction mixture using 1,1,2,2-tetrachloroethane as an internal standard.

b The amount of the framework or organic linker used was the same as that in the case of **1D-PDI–dpp­(phen)­Cu**.

c Reaction time was extended to 24 h.


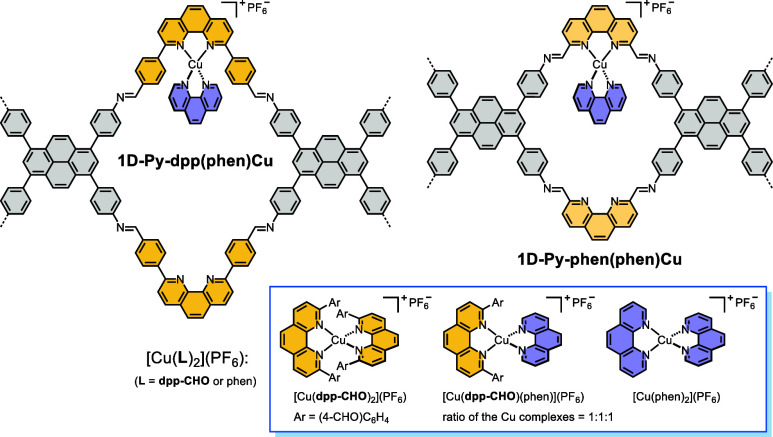
With the optimized conditions established, we explored the scope of styrene substrates in the heterogeneous photocatalytic oxo-azidation (Table 2). Both electron-donating and electron-withdrawing functional groups at various positions on the aryl ring were well tolerated, furnishing α-azido ketones in synthetically useful yields (products **1**–**18**). The copper catalyst achieved a turnover number (TON) of up to 760, surpassing the performance of existing homogeneous and heterogeneous systems (Table S5). The high compatibility with nitrogen-containing functionalities and halogen atoms demonstrated the robustness of the current methodology for chemical synthesis (products **6** and **11–17**). In addition to achieving higher TONs compared to conventional copper catalysis (Table S6), the use of highly electron-rich 2-methoxy-4-vinylphenol, which was previously incompatible with the homogeneous catalytic system,^67^ successfully yielded the α-azido ketone (18) with only 0.1 mol % of **1D-PDI–dpp­(phen)­Cu**. Furthermore, the substrate scope could be further expanded to include naphthalene and heterocycles such as pyridines, indoles, and thiophenes (products **19–22**). Notably, while allylbenzene and trisubstituted alkenes were not suitable for oxo-azidation, the use of β-methylstyrene, an internal alkene, afforded desired product **23** in 45% yield. The double bond functionalization process is also applicable to the alkenes derived from complex bioactive molecules (products **24** and **25**), including estrone and formononetin. Moreover, this heterogeneous photocatalysis can be employed in a variety of alkene difunctionalization reactions with low copper loadings (Figures S27–S31), underscoring its versatility and promising potential for practical applications in organic synthesis.

**Table 2:**
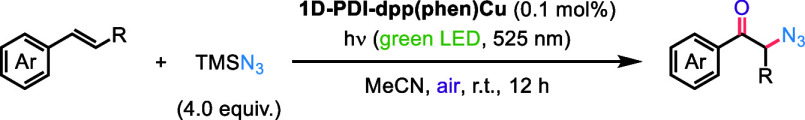

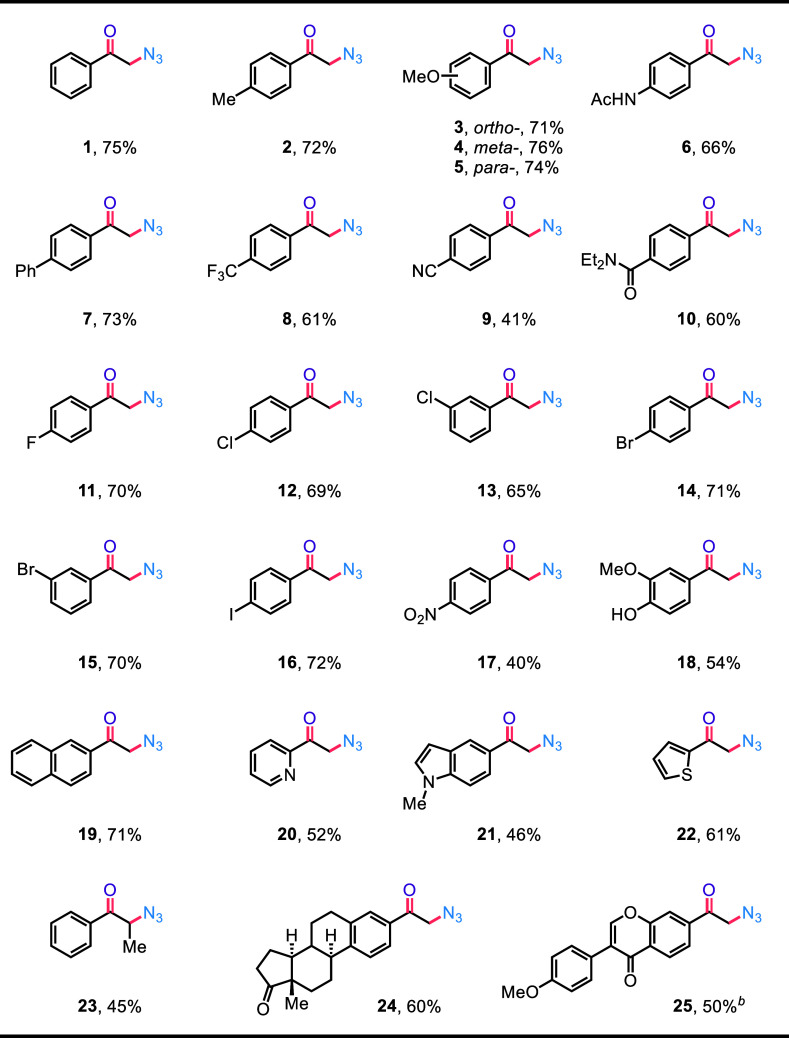
Substrate Scope of Heterogeneous Photocatalytic Oxo-Azidation^a^

a Reaction conditions: styrene derivative (0.10 mmol, 1.0 equiv), TMSN_3_ (4.0 equiv), and **1D-PDI** **–** **dpp­(phen)­Cu** (0.1 mol %) in acetonitrile (0.4 mL) under air atmosphere at room temperature with green-LED light irradiation (525 nm) for 12 h. Data are reported as isolated yields.

b Dichloromethane was used as the solvent instead, due to the substrate’s high solubility.

Compared to **1D-PDI** **–** **phen­(phen)­Cu**, which showed significantly reduced activity and lower signal intensity in PXRD measurements after the first catalytic run (Table S8 and Figure S34),^68^ the optimal catalyst assembled from **dpp–CHO** and **PDI–NH**_**2**_ demonstrated excellent recyclability (Figure 2a,b). The template oxo-azidation reaction delivered product **1** with a 70% isolated yield in the fourth run, alongside well-preserved PXRD patterns of the framework. XPS analysis revealed that the heterogenized heteroleptic phen-ligated copper complex was partially oxidized to copper­(II)^69^ during the photoredox catalysis in air (Figure 2c). Critically, ICP and NMR analyses of the digested samples evidenced no leaching of copper or the phen ligand, supporting the role of this heteroleptic complex as the actual catalytic species (Figure 2d and Table S3). To further elucidate the details of the heterogeneous photocatalytic process involving the photoactive framework support, we performed electron paramagnetic resonance (EPR) experiments using 5,5-dimethyl-1-pyrroline *N*-oxide (DMPO) as the radical trapping reagent (Figure 2e,f). Under a nitrogen atmosphere, green light irradiation triggered the formation of a DMPO**–**N_3_ adduct,^70^ demonstrating that photoexcited **1D-PDI** **–** **dpp** can effectively extract an electron from TMSN_3_ to generate an azide radical. However, the expected photoluminescent quenching of **1D-PDI** **–** **dpp** in the presence of TMSN_3_ was not observed (Figure S35), likely due to self-quenching effects within the COF layers. On the other hand, when the atmosphere was switched to air, the EPR signals clearly revealed distinct peaks corresponding to a DMPO**–**O_2_^–^ adduct after 5 min of irradiation.^71^ This suggests that the superoxide ion was most likely produced through electron transfer between reduced **1D-PDI** **–** **dpp** and O_2_.

**Figure 2:**
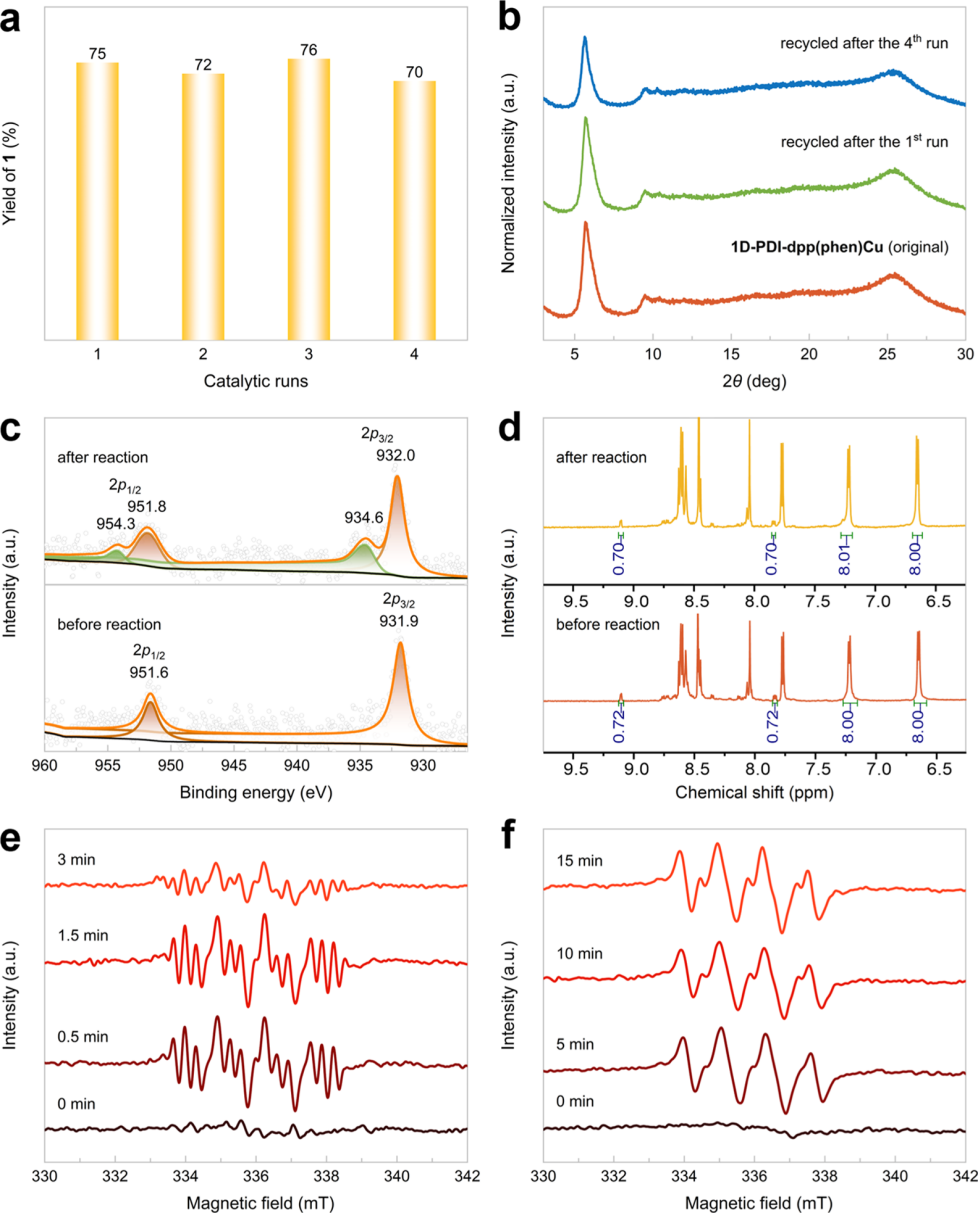
(a) Recycling experiments for photocatalytic oxo-azidation of styrene. (b) PXRD patterns of the original (orange line) and recycled **1D-PDI** **–** **dpp­(phen)­Cu** after one run (green line) and four runs (blue line). (c) Cu2*p* XPS spectra of **1D-** **PDI–dpp­(phen)­Cu** before and after oxo-azidation. (d) ^1^H NMR spectra of digested **1D-PDI** **–** **dpp­(phen)­Cu** samples before and after oxo-azidation. (e) X-band EPR spectra (9.43 GHz, 298 K) of **1D-PDI** **–** **dpp**, DMPO, and TMSN_3_ in acetonitrile under nitrogen atmosphere, recorded under irradiation (525 nm) for 0–3 min. (f) X-band EPR spectra (9.43 GHz, 298 K) of **1D-PDI** **–** **dpp**, DMPO, and TMSN_3_ in acetonitrile under air atmosphere, recorded under irradiation (525 nm) for 0–15 min.

To clarify the role of the immobilized copper species in the oxo-azidation, we removed the framework catalyst and benzaldehyde (**1-b**) from the reaction mixture by simple filtration through a short pad of silica gel, followed by evacuation (Figure 3a). Subsequently, we mimicked the original photocatalytic conditions by reintroducing TMSN_3_ and various types of COF-based catalysts into the mixture of compounds **1** and **1-a** in acetonitrile under air atmosphere. Control experiments were conducted in parallel without light exposure. Not surprisingly, the addition of **1D-PDI–dpp** did not lead to any further formation of product **1**. Peroxide **1-a** was not photochemically stable; it gradually decomposed and produced benzaldehyde upon light irradiation. These results demonstrate that the framework support itself is incapable of converting **1-a** to **1**. Using **1D-PDI–dppCu** as the catalyst slightly increased the yield of product **1**, from 20% to 25% in the absence of light and to 28% in its presence. The poor mass balance observed under green LED light again reflected the photochemical instability of peroxide **1-a**. Notably, the complete consumption of peroxide **1-a** was confirmed when **1D-PDI–dpp­(phen)­Cu** was employed. Substantial amounts of product **1** were obtained regardless of light irradiation, indicating that the heteroleptic phen-ligated copper complex on the framework support acts as the active site for the transformation of **1-a** into **1**. This nonphotoinduced process occurred rapidly enough to prevent the photochemical decomposition of peroxide **1-a**. Initial rate analysis revealed that the reaction follows first-order kinetics with respect to both **1-a** and **1D-PDI** **–** **dpp­(phen)­Cu** (Figures S36–S39). Furthermore, **1D-PDI** **–** **dppCu** was found to be considerably less effective (Figure S40), aligning with the observed trends in the studies of Cu^2+^ and phen ratios for the corresponding homogeneous catalytic system (Table S9).

**Figure 3:**
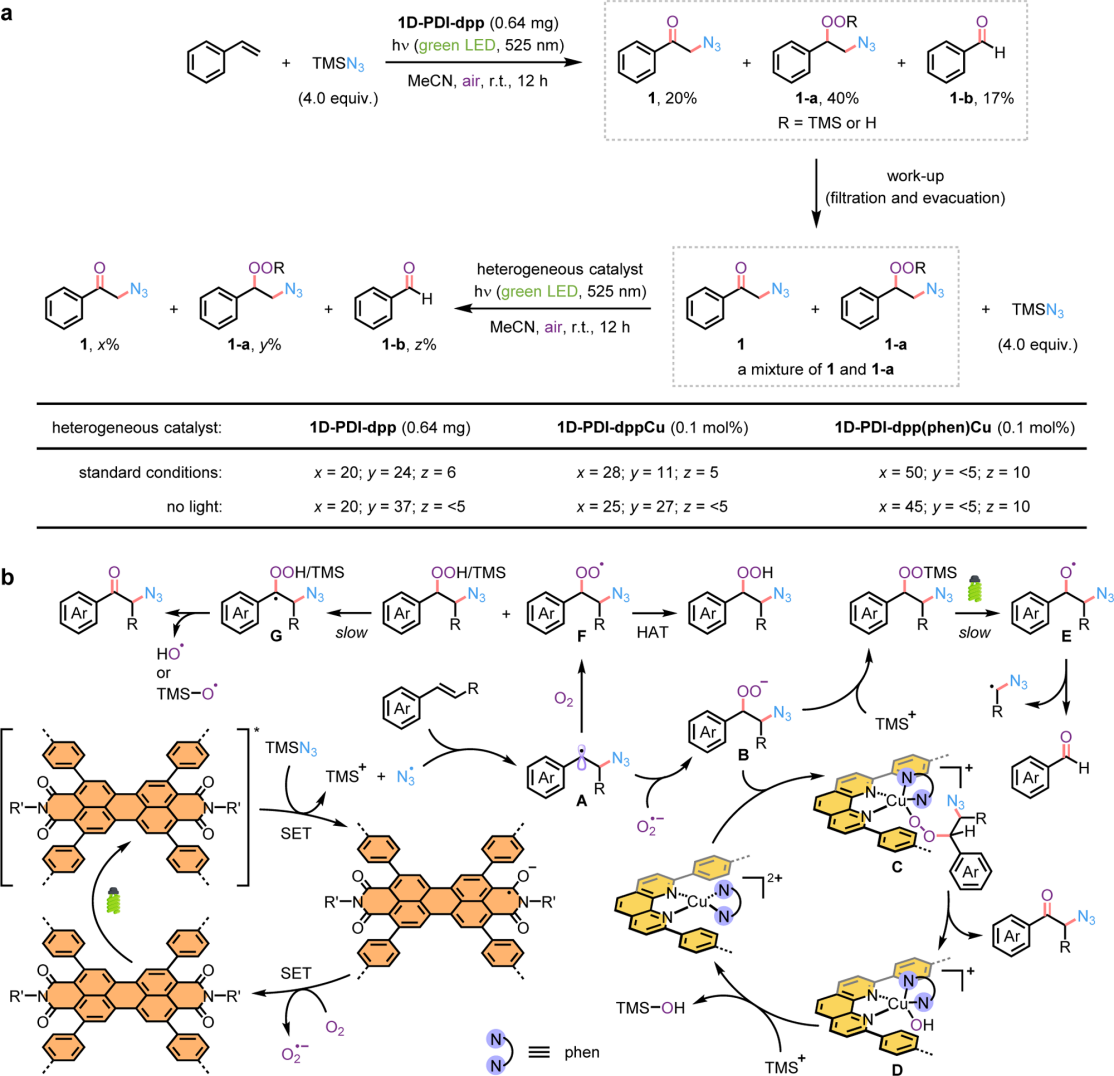
(a) Investigating the transformations of side-product **1-a** in the presence of different COF-based catalysts. Data are reported as crude ^1^H NMR yields, calculated with styrene as the limiting reagent. (b) Plausible mechanism for oxo-azidation of styrene derivatives with **1D-PDI** **–** **dpp­(phen)­Cu**. HAT, hydrogen atom transfer.

A comprehensive mechanistic understanding, obtained through materials characterization, EPR spectroscopy, and well-designed control experiments, enabled us to propose the reaction pathways for the heterogeneous metallaphotoredox catalysis depicted in Figure 3b. When exposed to green LED light, the photoexcited PDI unit in **1D-PDI** **–** **dpp** undergoes SET with TMSN_3_, generating a PDI radical anion, TMS^+^, and an azide radical. The nitrogen-centered radical then reacts with styrene via radical addition, forming a more stable benzylic radical­(**A**). The superoxide ion, provided by the single-electron reduction of O_2_ by the PDI radical anion, couples with intermediate **A** through radical recombination to form a β-azido peroxide (**B**). The COF-supported, heteroleptic phen-ligated copper­(II) complex utilizes its vacant coordination sites to capture intermediate **B**, yielding a five- or six-coordinate copper–alkylperoxo adduct (**C**), which rapidly affords the desired oxo-azidation product and hydroxide-ligated copper complex **D**. Subsequently, trapping with TMS^+^ produces a strong Si–O bond and regenerates the cationic copper­(II) catalyst, thereby closing the catalytic cycle.

In addition to the primary reaction pathway, parallel radical mechanisms operate. First, intermediate **B** can be trapped by TMS^+^ to form a silyl-substituted peroxide. Upon light irradiation, this peroxide undergoes homolytic O–O bond cleavage to generate a benzyloxy radical (**E**), which then fragments via β-scission of its C–C bond to yield benzaldehyde. Simultaneously, intermediate **A** gradually reacts with O_2_ to form a benzyl peroxide radical intermediate (**F**). This highly reactive species can abstract a hydrogen atom either from a solvent molecule to form a hydrogen peroxide derivative or from the benzylic position of peroxide side products at a slow rate, leading to an alkyl radical intermediate (**G**) adjacent to a peroxide group. A final homolytic cleavage of the O–O bond in intermediate **G** delivers the desired product via a noncopper-catalyzed pathway, along with an oxygen-centered radical. Obviously, in the absence of intermediate **F**, the peroxide side products cannot be converted to the desired oxo-azidation product without the assistance of the copper active center bearing two different phen-type ligands.

## Conclusions

In the current study, we report the synthesis and characterization of a heteroleptic phen-ligated copper complex embedded within a stable PDI-based covalent organic framework featuring a unique AB-slipped stacking mode. Compared to the corresponding monophen-ligated copper complex and its pyrene-based 1D framework-supported counterparts, this recyclable heterogeneous copper photocatalyst exhibits superior activity in the oxo-azidation of styrenes with TMSN_3_ under air atmosphere. Mechanistic investigations reveal that the ketone formation step in the three-component radical-mediated process is facilitated by the copper active center containing two phen ligands, rather than one —a critical insight unattainable in conventional homogeneous photocatalytic systems. Further applications in heterogeneous metallaphotoredox catalysis are currently underway in our laboratory.

## Supplementary Material


