# Supplementary material for: Construction of Heteroleptic Copper Complexes in Perylene Diimide-Based COFs for Heterogeneous Metallaphotoredox Catalysis
Source: J Am Chem Soc. 2026 Jan 23;148(4):4283–94. doi: 10.1021/jacs.5c17585 (PMC12879942; doi:10.1021/jacs.5c17585)
Supplement: Supplementary file 1 [file ja5c17585_si_001.pdf]

Supporting Information

Construction of Heteroleptic Copper Complexes in Perylene Diimide-Based COFs for Heterogeneous Metallaphotoredox Catalysis

Xia Wu[1], Jun Guo[1,2], Meng-Ying Sun[1,3], Tao Du[4], Debo Hao[5], Dongyi Liu[1], Deyang Wang[1,2], Songwei Wen[1], Jun Yin[4], Dan Li[5], and Jian He[1,2,3]*

[1]Department of Chemistry, The University of Hong Kong, Hong Kong 999077, P. R. China.

[2] State Key Laboratory of Synthetic Chemistry and Shanghai-Hong Kong Joint Laboratory in Chemical Synthesis, The University of Hong Kong, Hong Kong 999077, China.

[3]Materials Innovation Institute for Life Sciences and Energy (MILES), HKU-SIRI, Shenzhen 518048, P. R. China.

[4]Department of Applied Physics, The Hong Kong Polytechnic University, Hung Hom, Kowloon, Hong Kong, 999077, P. R. China

[5]Guangdong Provincial Key Laboratory of Supramolecular Coordination Chemistry, Jinan University, Guangzhou 510632, P. R. China.

*Email: jianhe@hku.hk

Table of Contents

1. General Information S–3
2. Preparation of Organic Linkers S–5
3. Preparation of Copper Complexes S–10
4. Preparation of **1D-PDI-COFs** and **1D-Py-COFs** S–12
   4.1 Synthesis of **1D-PDI-dpp**, **1D-PDI-dppCu** and **1D-PDI-dpp(phen)Cu** S–12
   4.2 Synthesis of **1D-PDI-phen**, **1D-PDI-phenCu** and **1D-PDI-phen(phen)Cu** S–14
   4.3 Synthesis of **1D-Py-dpp**, **1D-Py-dppCu** and **1D-Py-dpp(phen)Cu** S–16
   4.4 Synthesis of **1D-Py-phen**, **1D-Py-phenCu** and **1D-Py-phen(phen)Cu** S–18
5. Characterizations of COF Materials S–20

6. Photocatalytic Applications of COF Materials S–42

6.1 Condition Screening for Heterogeneous Copper Catalysis S–42

6.2 Synthesis of Substrates S–44

6.3 Heterogeneous Photoinduced Oxo-Azidation of Styrenes S–44

6.4 Other Photocatalytic Applications S–57

7. Stability Tests of **1D-PDI-COFs** S–62

7.1 Solvent Stability Studies of **1D-PDI-dpp** S–62

7.2 Recycling Experiments of **1D-PDI-dpp(phen)Cu** S–62

7.3 Recycling Experiments of **1D-PDI-dppCu** S–63

7.4 Recycling Experiments of **1D-PDI-phen(phen)Cu** S–64

8. Mechanistic Studies S–65

8.1 Electron Paramagnetic Resonance (EPR) Studies S–65

8.2 Luminescence Studies S–66

8.3 Rate Profile Measurements S–66

8.4 Investigation of Copper Coordination Modes in Peroxide Conversion S–71

9. References S–72

10. NMR Spectra S–73

1. General Information

Unless otherwise noted, materials were either purchased from commercial suppliers and used as received or prepared via literature procedures. Unless otherwise noted, all reactions were carried out in reaction tubes using common solvents (analytical reagent) under air atmosphere.

^{1}H NMR spectra were recorded on a Bruker 400 (400 MHz), Bruker 500 (500 MHz) or Bruker 600 (600 MHz) spectrometer in Acetonitrile-d_3, DMSO-d_6 or $CDCl_3$. Chemical shifts were quoted in parts per million (ppm) referenced to 1.94 ppm for the solvent residual peak of Acetonitrile-d_3, 2.50 ppm for the solvent residual peak of DMSO-d_6, 0.0 ppm for tetramethylsilane (TMS) or 7.26 ppm for the solvent residual peak of $CDCl_3$. The following abbreviations (or combinations thereof) were used to explain multiplicities: s = singlet, d = doublet, t = triplet, q = quartet, m = multiplet, br = broad. Coupling constants, *J*, were reported in Hertz unit (Hz). ^{13}C NMR spectra were recorded on a Bruker 400 (100 MHz), Bruker 500 (125 MHz) or Bruker 600 (150 MHz) spectrometer in Acetonitrile-d_3, DMSO-d_6 or $CDCl_3$ and were fully decoupled by broad band proton decoupling. Chemical shifts were reported in ppm referenced to the center line of a multiplet at 1.32 ppm of Acetonitrile-d_3, the center line of a multiplet at 39.5 ppm of DMSO-d_6 or the center line of a triplet at 77.0 ppm of $CDCl_3$.

Powder X-ray diffraction (PXRD) patterns were recorded on a Rigaku MiniFlex600 X-ray diffractometer (CuKα, λ = 1.5418 Å), operating at 40 kV and 30 mA. The measurement parameters included a scan speed of 10° min^{-1}, a step size of 0.01°.

Scanning electron microscopy (SEM) and EDS mapping were collected on a Zeiss Gemini 300 scanning electron microscope. Transmission electron microscopy (TEM) and EDS mapping were collected on a FEI Talos F200X scanning transmission electron Microscope with a Super X EDS detector.

X-ray photoelectron spectroscopy (XPS) was carried out on a Thermo ESCALAB QXi system, and the spectra were analyzed using the Thermo Scientific Avantage Data System software.

Inductively coupled plasma atomic emission spectroscopy (ICP-AES) measurements were conducted on an Agilent 7700x system.

The thermogravimetry analyses (TGA) were carried out on HITACHI STA200 with a heating rate of 10 °C min^{-1} from 30 to 800 °C in N_2 atmosphere.

Fourier transfomm infrared (FT-IR) spectroscopy was collected on a Themmo Nicolet iS10 FT-IR spectrometer.

The solid-state ultraviolet-visible absorption spectra were recorded on a Cary 5000 UV−vis−NIR spectrophotometer.

High-resolution EI mass spectra were recorded on a Thermo Scientific DFS Magnetic Sector GC-HRMS system. High-resolution ESI-MS measurements were performed on a Bruker impact II high-resolution LC-QTOF mass spectrometer. Accurate masses from high-resolution mass spectra were reported for the molecular ion $[M]^+$ or $[M+H]^+$.

Nitrogen gas sorption experiments were performed on a Micrometritics ASAP 2020 Plus Physisorption system. The samples were dried upon heating at 90 °C for 12 h under vacuum before the gas sorption experiments. Surface areas of the materials were obtained based on adsorption data analyzed by Brunauer−Emmett−Teller (BET) methods. The pore size distributions were calculated from the adsorption branches by density functional theory (DFT) method.

The steady-state emission spectroscopy and the time-resolved emission spectroscopy were measured at room temperature on an Edinburgh FLS1000 spectrofluorometer, using Xenon Arc Lamp (230 nm to >1000 nm) as the excitation source. The emission lifetime measurements were performed on a PMT detector (185–900 nm), using Picosecond pulsed diode laser 375 nm (65ps @10MHz) as the excitation source.

X-band electron paramagnetic resonance (EPR) measurements were carried out at room temperature using a Bruker EMX A200 spectrometer.

The ^{13}C cross-polarization magic angle spinning (CP-MAS) NMR spectra were recorded on a Bruker Avance NEO wide-bore spectrometer of 14 T, with ^1H Larmor frequency of 600 MHz, equipped with a 4 mm MAS probe in double resonance mode (Bruker, Germany). The MAS rate was set to 10 and 12 kHz to identify spinning sidebands. The NMR parameters used were: 90° pulse lengths ^1H: 3 μs, ^{13}C: 5 μs, CP contact time 1.5 ms, 83 kHz ^1H decoupling. A ramped shape was applied on the proton channel during the CP contact. A recycle delay of 3 s was used. The NMR experiments were carried out at room temperature.

The Pawley refinements were performed using Materials Studio 2018.

2. Preparation of Organic Linkers

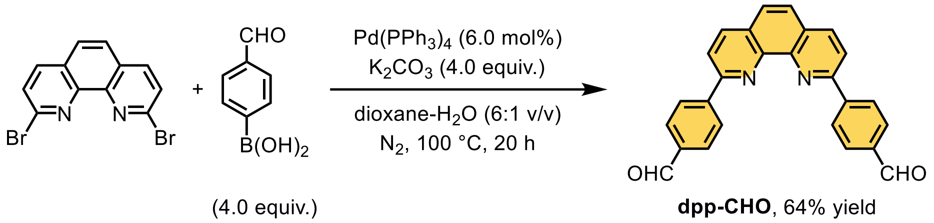

4,4'-(1,10-Phenanthroline-2,9-diyl)dibenzaldehyde (dpp-CHO).[1] To a solution of 2,9-dibromo-1,10-phenanthroline (0.34 g, 1.0 mmol, 1.0 equiv.) in 1,4-dioxane (4.0 mL) and water (0.67 mL), (4-formylphenyl)boronic acid (0.60 g, 4.0 mmol, 4.0 equiv.), $Pd(PPh_3)_4$ (0.069 g, 0.06 mmol, 6.0 mol%), and potassium carbonate (0.55 g, 4.0 mmol, 4.0 equiv.) was added. After stirring at 100 °C for 20 hours under N_2 atmosphere, the reaction mixture was diluted with water, and the aqueous layer was extracted with dichloromethane (DCM) (3 × 20 mL). The combined organic layers were washed with water and brine, dried over anhydrous sodium sulfate, and concentrated *in vacuo*. The crude mixture was purified by flash column chromatography using DCM-ethyl acetate (15:1 v/v) as the eluent to give title compound as a white solid (0.25 g, 0.64 mmol, 64% yield).

^{1}H NMR ($CDCl_3$, 600 MHz): δ 10.15 (s, 2H), 8.62 (d, *J* = 8.4 Hz, 4H), 8.39 (d, *J* = 8.4 Hz, 2H), 8.22 (d, *J* = 8.4 Hz, 2H), 8.11 (d, *J* = 8.4 Hz, 4H), 7.87 (s, 2H).

^{13}C NMR ($CDCl_3$, 150 MHz): δ 192.1, 155.5, 146.2, 144.9, 137.3, 136.8, 130.3, 128.5, 128.2, 126.7, 120.6.

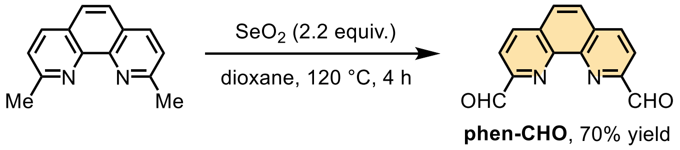

1,10-Phenanthroline-2,9-dicarbaldehyde (phen-CHO).[2] To a solution of 2,9-dimethyl-1,10-phenanthroline (1.04 g, 5.0 mmol, 1.0 equiv.) in dioxane (40 mL), selenium dioxide (1.22 g, 11.0 mmol, 2.2 equiv.) was added. After stirring at 120 °C for 4 hours, the suspension was filtered while hot through celite and the filtrate was cooled down at room temperature. The

formed precipitate was collected by filtration, washed with diethyl ether thoroughly and dried under vacuum to give **phen-CHO** as a light-yellow solid (0.83 g, 3.5 mmol, 70% yield).

^{1}H NMR (DMSO-d_6, 600 MHz): δ 10.35 (s, 2H), 8.79 (d, J = 7.8 Hz, 2H), 8.31 (d, J = 8.4 Hz, 2H), 8.28 (s, 2H).

^{13}C NMR (DMSO-d_6, 150 MHz): δ 193.7, 152.2, 145.2, 138.4, 131.4, 129.2, 120.1.

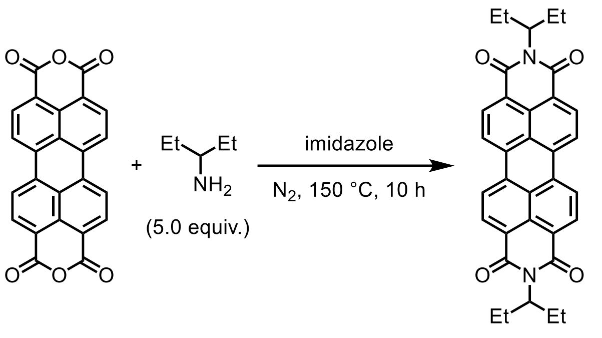

***N,N'*-bis(3-pentyl)perylene-3,4,9,10-bis(dicarboximide)** (**L1**).[3] To a mixture of anthra[2,1,9-*def*:6,5,10-*d'e'f'*]diisochromene-1,3,8,10-tetraone (2.26 g, 5.76 mmol, 1.0 equiv.) and pentan-3-amine (2.51 g, 28.8 mmol, 5.0 equiv.), imidazole (16.0 g, 235 mmol, 40.8 equiv.) was added. After stirring at 150 °C for 10 hours under N_2 atmosphere, an aqueous solution of hydrochloric acid (1.0 M) was added to neutralize the reaction mixture to pH = 6. The formed precipitate was collected by filtration, washed extensively with water. Finally, the solid was collected and dried at 100 °C for 12 h to give **L1** as a red solid (2.90 g, 5.47 mmol, 95% yield).

^{1}H NMR ($CDCl_3$, 600 MHz): δ 8.64 (d, J = 7.8 Hz, 4H), 8.57 (d, J = 7.8 Hz, 4H), 5.13 – 5.00 (m, 2H), 2.35 – 2.18 (m, 4H), 2.01 – 1.88 (m, 4H), 0.93 (t, J = 7.8 Hz, 12H).

^{13}C NMR ($CDCl_3$, 150 MHz): δ 164.1, 134.4, 131.4, 129.6, 126.4, 123.5, 123.0, 57.7, 25.0, 11.4.

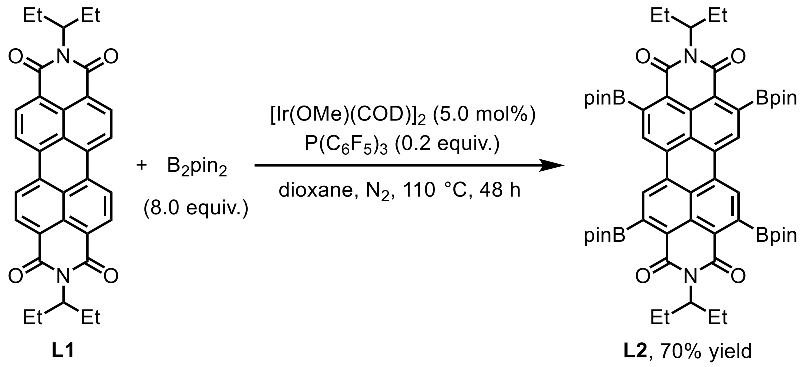

***N,N'*-bis(3-pentyl)perylene-3,4,9,10-bis(dicarboximide)-2,5,8,11-tetrakis(boronic acid pinacol ester)** (**L2**).[3] To a solution of **L1** (1.33 g, 2.5 mmol, 1.0 equiv.) in dioxane (100 mL), bis(pinacolato)diboron (5.08 g, 20.0 mmol, 8.0 equiv.), $[Ir(OMe)(COD)]_2$ (82.9 mg, 0.125 mmol, 5 mol%) and tris(pentafluorophenyl)phosphin (256 mg, 0.5 mmol, 0.2 equiv.) was added. After stirring at 110 °C for 48 hours under N_2 atmosphere, the crude reaction mixture was concentrated under vacuum and purified by flash column chromatography using DCM as the eluent to give **L2** as a red solid (1.81 g, 1.75 mmol, 70% yield).

^{1}H NMR ($CDCl_3$, 400 MHz): δ 8.52 (s, 4H), 4.98 – 4.82 (m, 2H), 2.25 – 2.10 (m, 4H), 2.00 – 1.88 (m, 4H), 1.54 (s, 48H), 0.91 (t, J = 7.2 Hz, 12H).

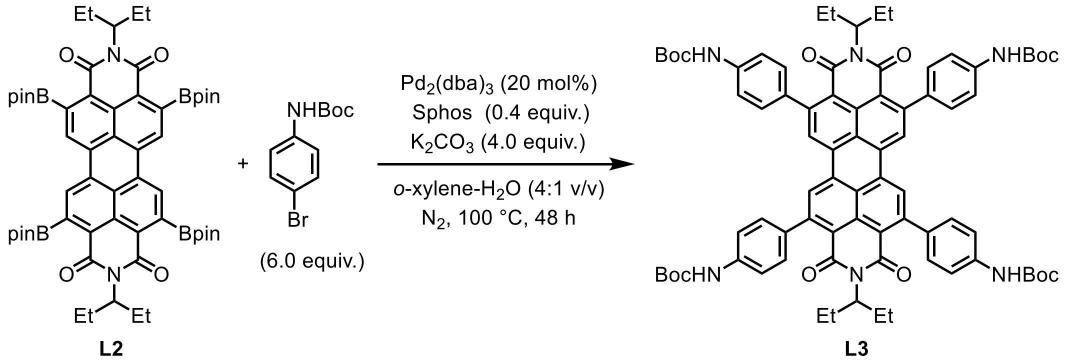

***N,N'*-bis(3-pentyl)-2,5,8,11-tetrakis(4-*N*-Boc-aminophenyl)perylene-3,4,9,10-bis(dicarboximide)** (**L3**).[3] To a solution of **L2** (0.83 g, 0.8 mmol, 1.0 equiv.) in *o*-xylene (12.8 mL) and water (3.2 mL), *tert*-butyl (4-bromophenyl)carbamate (1.31 g, 4.8 mmol, 6.0 equiv.), tris(dibenzylideneacetone)dipalladium (147 mg, 0.16 mmol, 20 mol%), Sphos (131 mg, 0.32 mmol, 0.4 equiv.), and potassium carbonate (0.44 g, 3.2 mmol, 4.0 equiv.) was added. After stirring at 100 °C for 48 hours under N_2 atmosphere, the crude reaction mixture collected by filtration, washed with water and ethyl acetate. This compound was used directly in the next step.

^{1}H NMR (DMSO-d_6, 400 MHz): δ 9.50 (s, 4H), 8.73 (s, 4H), 7.53 (d, J = 8.4 Hz, 8H), 7.39 (d, J = 8.4 Hz, 8H), 4.72 – 4.53 (m, 2H), 2.11 – 1.88 (m, 4H), 1.73 – 1.58 (m, 4H), 1.52 (s, 36H), 0.84 (d, J = 7.2 Hz, 12H).

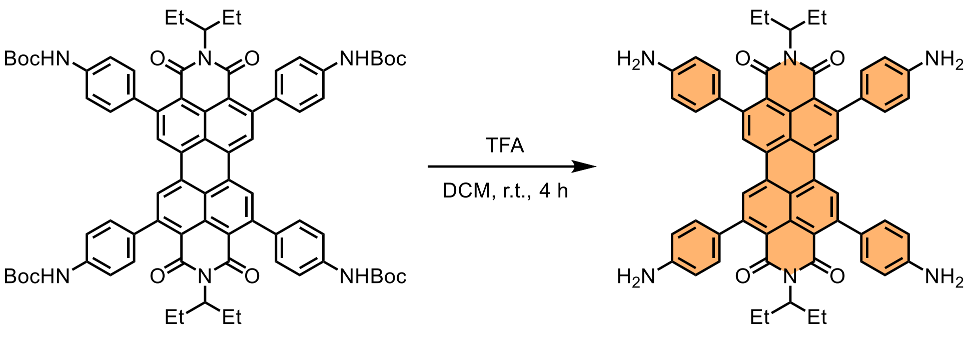

L3 PDI-NH$_2$, 60% yield

***N,N'*-bis(3-pentyl)-2,5,8,11-tetrakis(4-aminophenyl)perylene-3,4,9,10-bis(dicarboximide)** (**PDI-NH$_2$**).[3] To a solution of **L3** (1.04 g, 0.8 mmol, 1.0 equiv.) in DCM (50 mL), TFA (15 mL) was added. After stirring at room temperature for 4 hours, the crude reaction mixture was concentrated under vacuum. A saturated solution of sodium bicarbonate was added to neutralize the reaction mixture to pH = 8. The formed precipitate was collected by filtration, and purified by flash column chromatography using DCM-ethyl acetate (2:1 v/v) as the eluent to give **PDI-NH$_2$** as a black solid (430 mg, 0.48 mmol, 60% yield).

^{1}H NMR (DMSO-d_6, 600 MHz): δ 8.57 (s, 4H), 7.22 (d, J = 8.4 Hz, 8H), 6.64 (d, J = 8.4 Hz, 8H), 5.36 (s, 8H), 4.74 – 4.59 (m, 2H), 2.08 – 1.97 (m, 4H), 1.78 – 1.61 (m, 4H), 0.87 (t, J = 7.6 Hz, 12H).

^{13}C NMR (DMSO-d_6, 150 MHz): δ 163.9, 148.9, 147.6, 132.0, 130.8, 130.0, 128.7, 127.1, 123.2, 119.6, 113.4, 56.9, 24.5, 11.2.

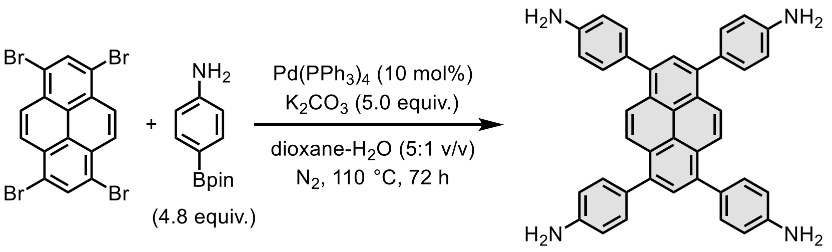

Py-NH$_2$, 70% yield

4,4',4'',4'''-(Pyrene-1,3,6,8-tetrayl)tetraaniline (**Py-NH$_2$**).[4] To a solution of 1,3,6,8-tetrabromopyrene (0.98 g, 1.9 mmol, 1.0 equiv.) in dioxane (40 mL) and water (8.0 mL), 4-

(4,4,5,5-tetramethyl-1,3,2-dioxaborolan-2-yl)aniline (2.0 g, 9.1 mmol, 4.8 equiv.), palladium-tetrakis(triphenylphosphine) (220 mg, 0.19 mmol, 10 mol%), and potassium carbonate (1.31 g, 9.5 mmol, 5.0 equiv.) was added. After stirring at 110 °C for 72 hours under N_2 atmosphere, the crude reaction mixture collected by filtration, washed with water and chloroform to give **Py-NH$_2$** as a yellow solid (754 mg, 1.33 mmol, 70% yield).

^{1}H NMR (DMSO-d_6, 600 MHz): δ 8.12 (s, 4H), 7.78 (s, 2H), 7.34 (d, J = 8.4 Hz, 8H), 6.77 (d, J = 8.4 Hz, 8H), 5.30 (s, 8H).

3. Preparation of Copper Complexes

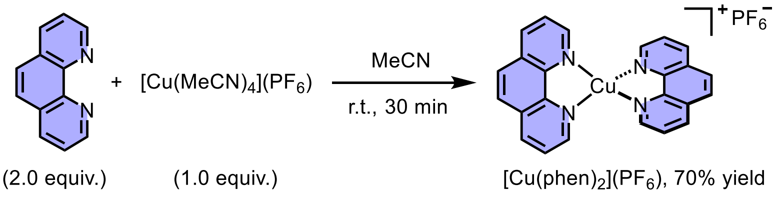

[Cu(phen)$_2$](PF$_6$).[5] To a solution of 1,10-phenanthroline (36 mg, 0.2 mmol, 2.0 equiv.) in MeCN (1.0 mL), [Cu(MeCN)$_4$](PF$_6$) (37 mg, 0.1 mmol, 1.0 equiv.) was added. After stirring at room temperature for 30 min under N_2 atmosphere, the product was then precipitated by adding 5.0 mL diethyl ether. The formed precipitate was collected by filtration, washed with diethyl ether and dried under vacuum to give the [Cu(phen)$_2$](PF$_6$) (39.8 mg, 0.07 mmol, 70% yield).

^{1}H NMR (acetonitrile-d_3, 400 MHz): δ 8.91 (d, J = 4.7 Hz, 4H), 8.69 (d, J = 8.1 Hz, 4H), 8.18 (s, 4H), 7.92 (dd, J = 8.2, 4.7 Hz, 4H).

^{13}C NMR (acetonitrile-d_3, 150 MHz): δ 150.2, 144.9, 138.1, 130.4, 128.1, 126.5.

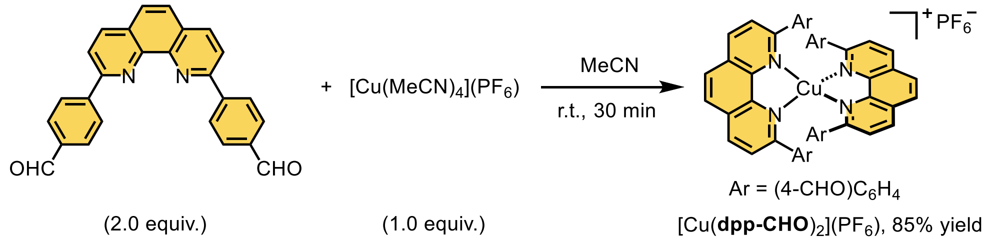

[Cu(dpp-CHO)$_2$](PF$_6$). To a solution of **dpp-CHO** (77.7 mg, 0.2 mmol, 2.0 equiv.) in MeCN (2.0 mL) and DCM (3.0 mL), [Cu(MeCN)$_4$](PF$_6$) (37 mg, 0.1 mmol, 1.0 equiv.) was added. After stirring at room temperature for 30 min under N_2 atmosphere, the solvent precipitate was collected by filtration, washed with diethyl ether thoroughly and dried under vacuum to give the [Cu(**dpp-CHO**)$_2$](PF$_6$) (83.8 mg, 0.085 mmol, 85% yield).

^{1}H NMR (acetonitrile-d_3, 400 MHz): δ 9.64 (s, 4H), 8.64 (d, J = 8.4 Hz, 4H), 8.05 – 8.00 (m, 8H), 7.58 (d, J = 8.1 Hz, 8H), 7.06 (d, J = 8.2 Hz, 8H).

^{13}C NMR (acetonitrile-d_3, 150 MHz): δ 192.6, 156.7, 145.2, 144.4, 139.0, 136.9, 129.8, 129.4, 129.2, 127.9, 126.2.

HRMS m/z (ESI) calc. for $[C_{52}H_{32}CuN_4O_4]^+$: 839.1715; found: 839.1713.

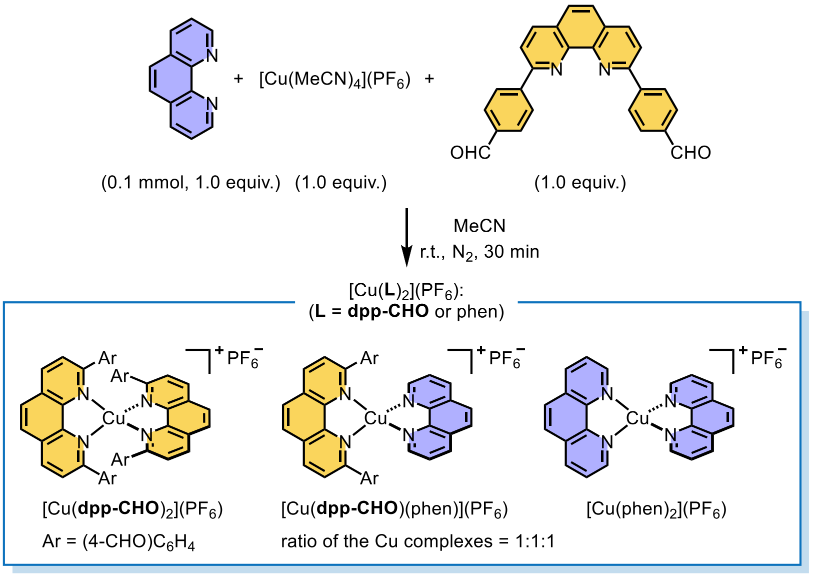

[Cu(phen-CHO)(phen)](PF$_6$). To a solution of 1,10-phenanthroline (17.8 mg, 0.1 mmol, 1.0 equiv.) and **dpp-CHO** (38.8 mg, 0.1 mmol, 1.0 equiv.) in MeCN (1.0 mL), [Cu(MeCN)$_4$](PF$_6$) (37.2 mg, 0.1 mmol, 1.0 equiv.) was added. After stirring at room temperature for 30 min under N$_2$ atmosphere, MeCN was removed under vacuum. The solid was transferred to glovebox, CD$_3$CN (1.0 mL) was added to test the ^{1}H NMR.

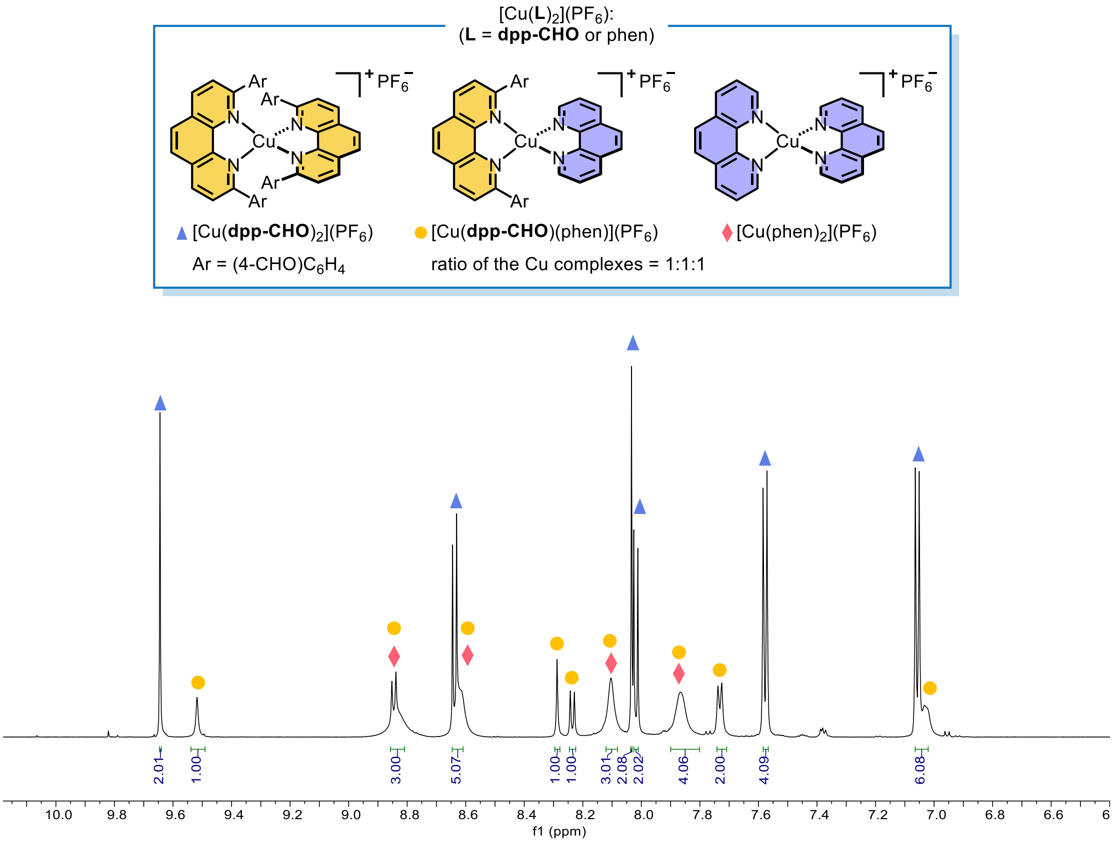

4. Preparation of 1D-PDI-COFs and 1D-Py-COFs

Table S1. Loadings of Heteroleptic Phen-Ligated and Mono-Phen-Ligated Copper Species in Various 1D COFs

Entry	**Cu(phen)@COF** or **Cu@COF**	Metalation time (h)	Cu loading (wt%)[a]	Metalation efficiency (%)[a]
1	**1D-PDI-dpp(phen)Cu**	36	0.95	18
2	**1D-PDI-phen(phen)Cu**	36	0.75	13
3	**1D-Py-dpp(phen)Cu**	36	1.5	25
4	**1D-Py-phen(phen)Cu**	36	1.4	20
5	**1D-PDI-dppCu**	24	0.95	17
6	**1D-PDI-phenCu**	24	0.90	14
7	**1D-Py-dppCu**	24	1.5	23
8	**1D-Py-phenCu**	24	1.3	17
9	**1D-PDI-dpp(dmp)Cu**	36	0.21	4

[a]Copper loadings were determined by ICP-AES. Dmp, 2,9-dimethyl-1,10-phenanthroline.

4.1 Synthesis of 1D-PDI-dpp, 1D-PDI-dppCu and 1D-PDI-dpp(phen)Cu

4.1.1 Synthesis of 1D-PDI-dpp

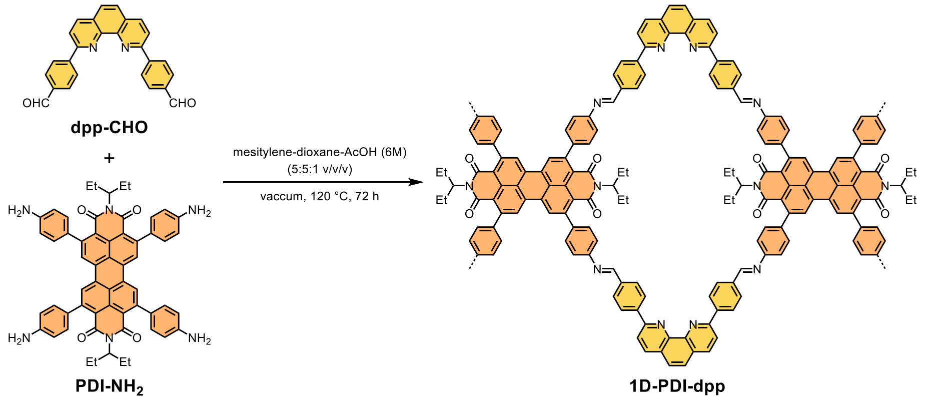

PDI-NH$_2$ (26.9 mg, 0.03 mmol) and **dpp-CHO** (23.3 mg, 0.06 mmol) were weighed into a 10-mL glass tube. Afterwards, mesitylene (0.75 mL) and dioxane (0.75 mL) were added, and the mixture was sonicated for 10 min. After addition of 0.15 mL 6 M aqueous acetic acid, the tube was degassed by the three freeze-pump thaw cycles and was then sealed. Upon warming to room temperature, the sealed tube was heated in an oven at 120 °C for 72 h. After reaction completion, the formed precipitate was collected by filtration, washed with DMF, acetone and dried at room temperature to give **1D-PDI-dpp** as a red powder. TGA data showed a 9.5% weight loss below 200 °C (Figure S14), attributed to the residual solvent in **1D-PDI-dpp** (40 mg, 75%).

4.1.2 Synthesis of 1D-PDI-dppCu

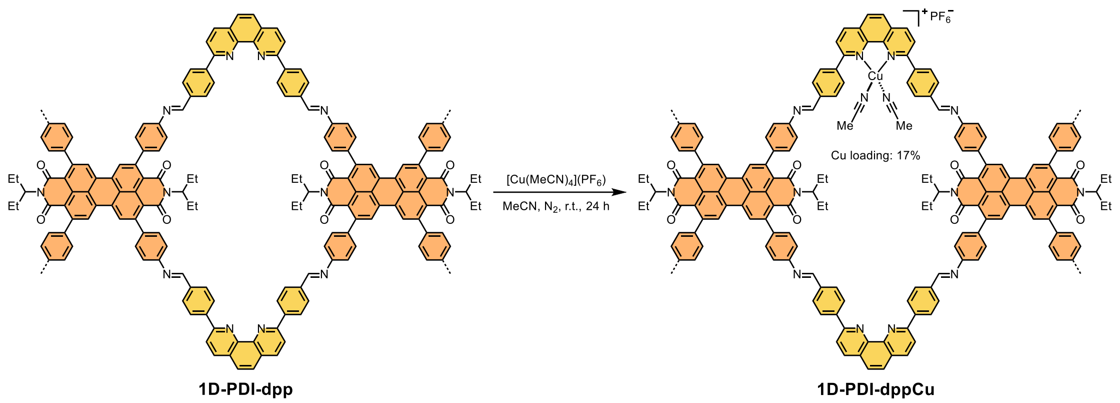

1D-PDI-dpp (30.0 mg, 37.5 μmol based on **dpp-CHO**) was immersed in a 3.0 mL MeCN solution of $[Cu(MeCN)_4](PF_6)$ (28 mg, 75 μmol) and stirred under N_2 atmosphere at room temperature for 24 hours. After reaction completion, the resulting solid was isolated by filtration and washed with a large amount of MeCN, then dried under vacuum at room temperature for 5 minutes to yield the **1D-PDI-dppCu**. ICP-AES analysis indicated a Cu loading of 0.95 wt%.

4.1.3 Synthesis of 1D-PDI-dpp(phen)Cu

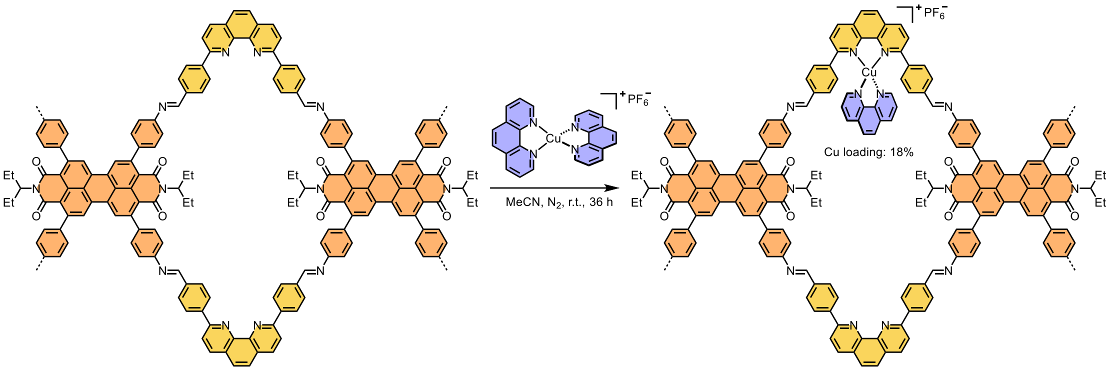

1D-PDI-dpp **1D-PDI-dpp(phen)Cu**

1D-PDI-dpp (30.0 mg, 37.5 μmol based on **dpp-CHO**) was immersed in a 3.0 mL MeCN solution of $[Cu(phen)_2](PF_6)$ (43 mg, 75 μmol) and stirred under N_2 atmosphere at room temperature for 36 hours. After reaction completion, the resulting solid was isolated by filtration and washed with a large amount of MeCN, then dried under vacuum at room temperature for 5 minutes to yield the **1D-PDI-dpp(phen)Cu**. ICP-AES analysis indicated a Cu loading of 0.95 wt%.

4.2 Synthesis of 1D-PDI-phen, 1D-PDI-phenCu and 1D-PDI-phen(phen)Cu

4.2.1 Synthesis of 1D-PDI-phen

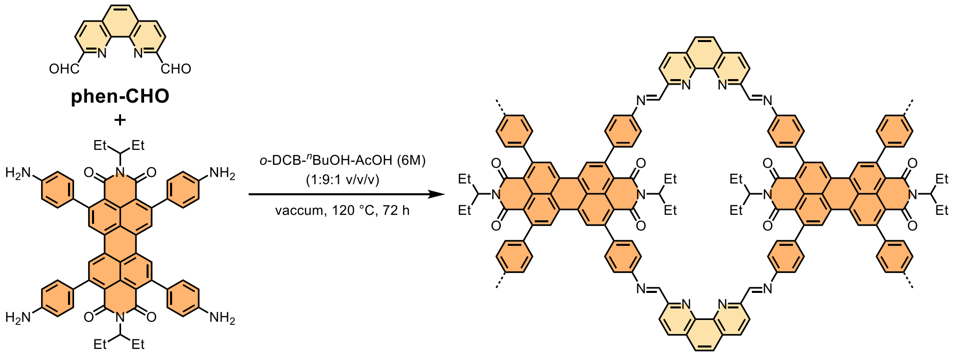

PDI-NH$_2$ **1D-PDI-phen**

PDI-NH$_2$ (26.9 mg, 0.03 mmol) and **phen-CHO** (14.2 mg, 0.06 mmol) were weighed into a 10-mL glass tube. Afterwards, *o*-dichlorobenzene (*o*-DCB, 0.1 mL) and n-butanol (^{n}BuOH, 0.9 mL) were added, and the mixture was sonicated for 10 min. After addition of 0.1 mL 6 M aqueous acetic acid, the tube was degassed by the three freeze-pump thaw cycles and was then sealed. Upon warming to room temperature, the sealed tube was heated in an oven at 120 °C

for 72 h. After reaction completion, the formed precipitate was collected by filtration, washed with DCM and further purification was carried out by Soxhlet extraction with acetone for 24 h and dried at room temperature to give **1D-PDI-phen** as a red powder. TGA data showed a 6.8% weight loss below 200 °C (Figure S14), attributed to the residual solvent in **1D-PDI-phen** (30 mg, 72%).

4.2.2 Synthesis of 1D-PDI-phenCu

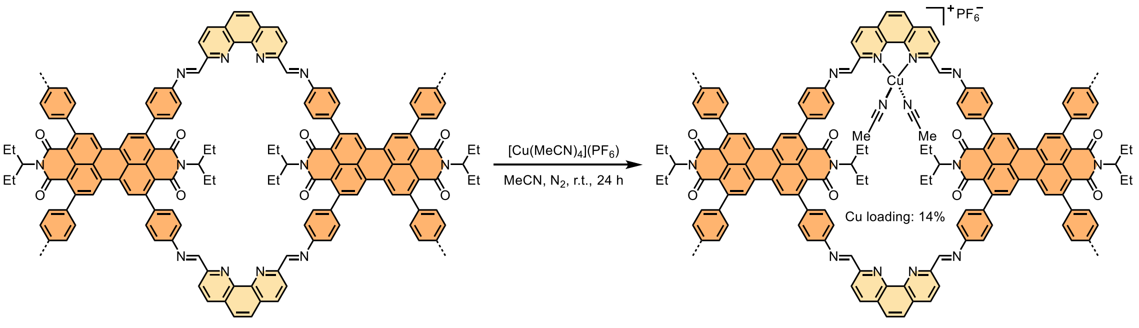

1D-PDI-phen 1D-PDI-phenCu

1D-PDI-phen (30 mg, 46 μmol based on **phen-CHO**) was immersed in a 3.0 mL MeCN solution of $[Cu(MeCN)_4](PF_6)$ (34 mg, 92 μmol) and stirred under N_2 atmosphere at room temperature for 24 hours. After reaction completion, the resulting solid was isolated by filtration and washed with a large amount of MeCN, then dried under vacuum at room temperature for 5 minutes to yield the **1D-PDI-phenCu**. ICP-AES analysis indicated a Cu loading of 0.90 wt%.

4.2.3 Synthesis of 1D-PDI-phen(phen)Cu

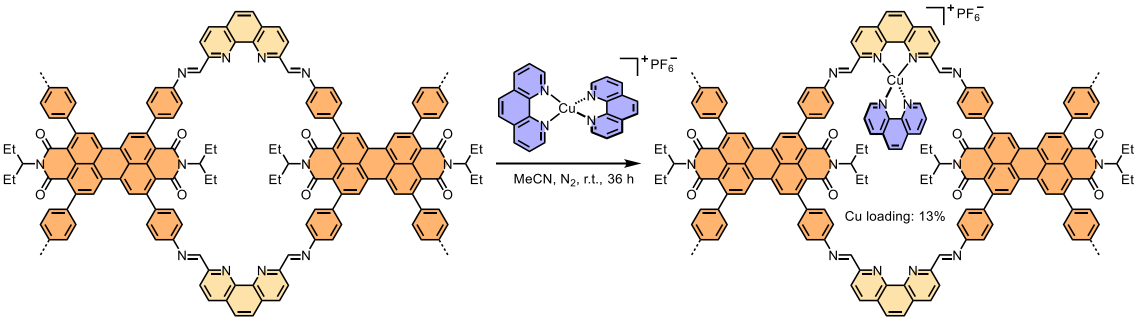

1D-PDI-phen 1D-PDI-phen(phen)Cu

1D-PDI-phen (30 mg, 46 μmol based on **phen-CHO**) was immersed in a 3.0 mL MeCN solution of $[Cu(phen)_2](PF_6)$ (52 mg, 92 μmol) and stirred under N_2 atmosphere at room temperature for 36 hours. After reaction completion, the resulting solid was isolated by filtration

and washed with a large amount of MeCN, then dried at room temperature under vacuum for 5 minutes to yield the **1D-PDI-phen(phen)Cu**. ICP-AES analysis indicated a Cu loading of 0.75 wt%.

4.3 Synthesis of 1D-Py-dpp, 1D-Py-dppCu and 1D-Py-dpp(phen)Cu

4.3.1 Synthesis of 1D-Py-dpp

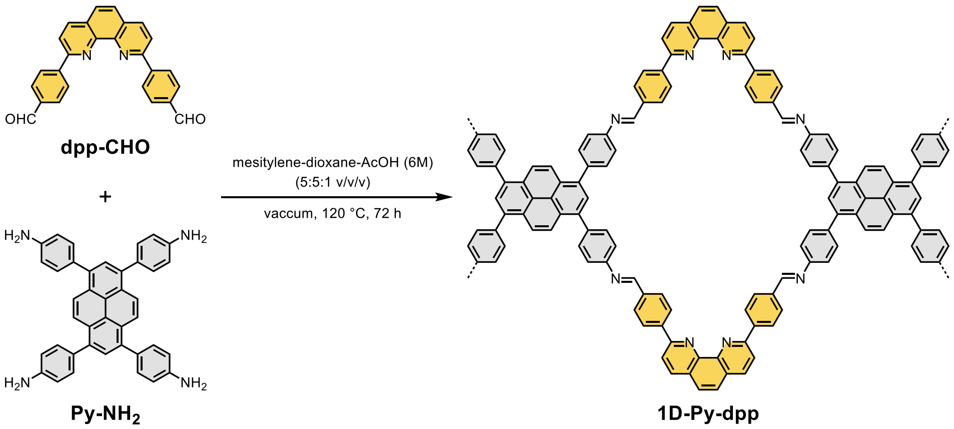

Py-NH$_2$ (5.7 mg, 0.01 mmol) and **dpp-CHO** (7.8 mg, 0.02 mmol) were weighed into a 10-mL glass tube. Afterwards, mesitylene (0.2 mL) and dioxane (0.2 mL) were added, and the mixture was sonicated for 10 min. After addition of 0.04 mL 6 M aqueous acetic acid, the tube was degassed by the three freeze-pump thaw cycles and was then sealed. Upon warming to room temperature, the sealed tube was heated in an oven at 120 °C for 72 h. After reaction completion, the formed precipitate was collected by filtration, washed with DCM and further purification was carried out by Soxhlet extraction in $CHCl_3$ for 24 h. Finally, the solid was collected and dried at 60 °C for 12 h to give **1D-Py-dpp** as a yellow powder. TGA data showed a 2.5% weight loss below 200 °C (Figure S15), attributed to the residual solvent in **1D-Py-dpp** (10 mg, 77%).

4.3.2 Synthesis of 1D-Py-dppCu

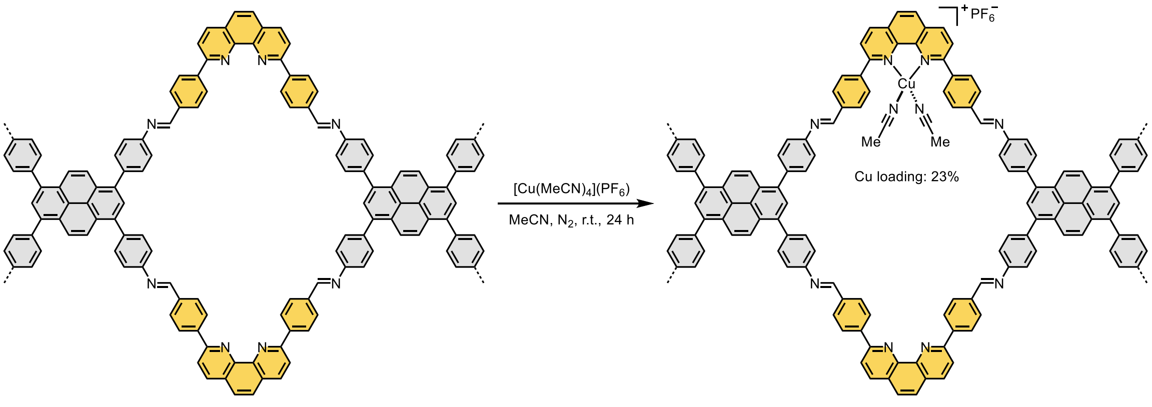

1D-Py-dpp (30 mg, 47 μmol based on **dpp-CHO**) was immersed in a 3.0 mL MeCN solution of $[Cu(MeCN)_4](PF_6)$ (35.0 mg, 94 μmol) and stirred under N_2 atmosphere at room temperature for 24 hours. After reaction completion, the resulting solid was isolated by filtration and washed with a large amount of MeCN, then dried at room temperature under vacuum for 5 minutes to yield the **1D-Py-dppCu**. ICP-AES analysis indicated a Cu loading of 1.5 wt%.

4.3.3 Synthesis of 1D-Py-dpp(phen)Cu

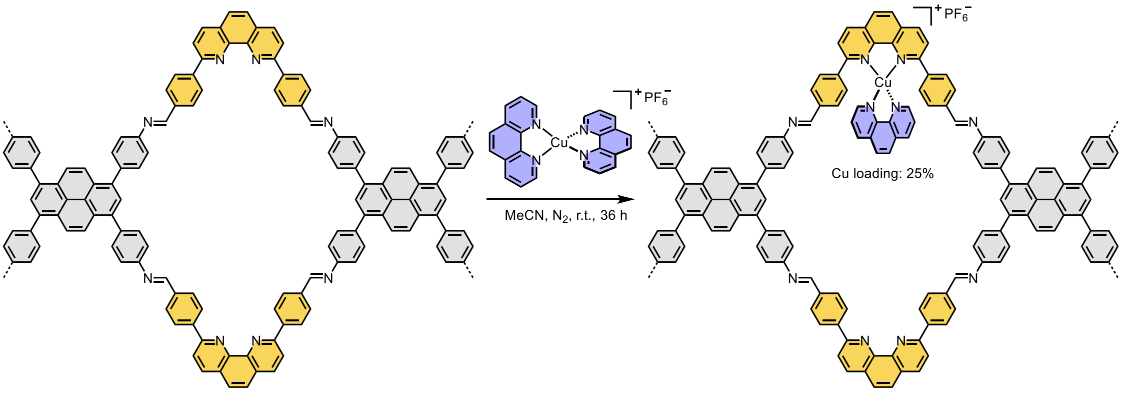

1D-Py-dpp (30.0 mg, 47 μmol based on **dpp-CHO**) was immersed in a 3.0 mL MeCN solution of $[Cu(phen)_2](PF_6)$ (53.5 mg, 94 μmol) and stirred under N_2 atmosphere at room temperature for 36 hours. After reaction completion, the resulting solid was isolated by filtration and washed with a large amount of MeCN, then dried at room temperature under vacuum for 5 minutes to yield the **1D-Py-dpp(phen)Cu**. ICP-AES analysis indicated a Cu loading of 1.5 wt%.

4.4 Synthesis of 1D-Py-phen, 1D-Py-phenCu and 1D-Py-phen(phen)Cu

4.4.1 Synthesis of 1D-Py-phen

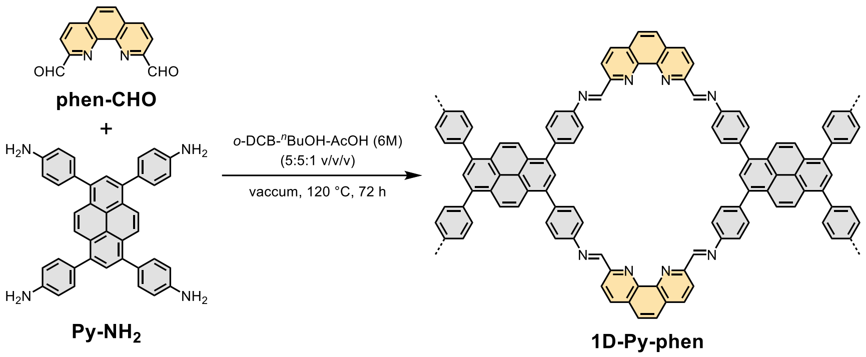

Py-NH₂ (5.7 mg, 0.01 mmol) and **phen-CHO** (4.7 mg, 0.02 mmol) were weighed into a 10-mL glass tube. Afterwards, *o*-dichlorobenzene (*o*-DCB, 0.2 mL) and *n*-butanol (^{n}BuOH, 0.2 mL) were added, and the mixture was sonicated for 10 min. After addition of 0.04 mL 6 M aqueous acetic acid, the tube was degassed by the three freeze-pump thaw cycles and was then sealed. Upon warming to room temperature, the sealed tube was heated in an oven at 120 °C for 72 h. After reaction completion, the formed precipitate was collected by filtration, washed with DCM and further purification was carried out by Soxhlet extraction in $CHCl_3$ for 24 h. Finally, the solid was collected and dried at 60 °C for 12 h to give **1D-Py-phen** as a yellow powder. TGA data showed a 5.6% weight loss below 200 °C (Figure S15), attributed to the residual solvent in **1D-Py-phen** (8 mg, 78%).

4.4.2 Synthesis of 1D-Py-phenCu

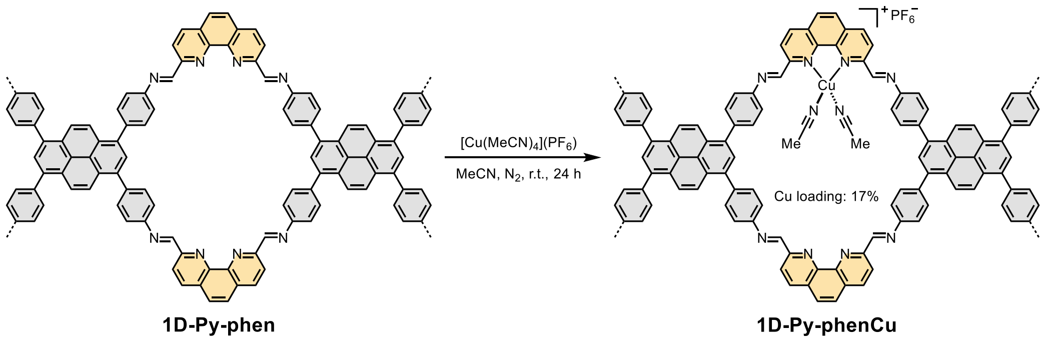

1D-Py-phen (30 mg, 62 μmol based on **phen-CHO**) was immersed in a 3.0 mL MeCN solution of $[Cu(MeCN)_4](PF_6)$ (46.2 mg, 124 μmol) and stirred under N_2 atmosphere at room temperature for 24 hours. After reaction completion, the resulting solid was isolated by filtration

and washed with a large amount of MeCN, then dried at room temperature under vacuum for 5 minutes to yield the **1D-Py-phenCu**. ICP-AES analysis indicated a Cu loading of 1.3 wt%.

4.4.3 Synthesis of 1D-Py-phen(phen)Cu

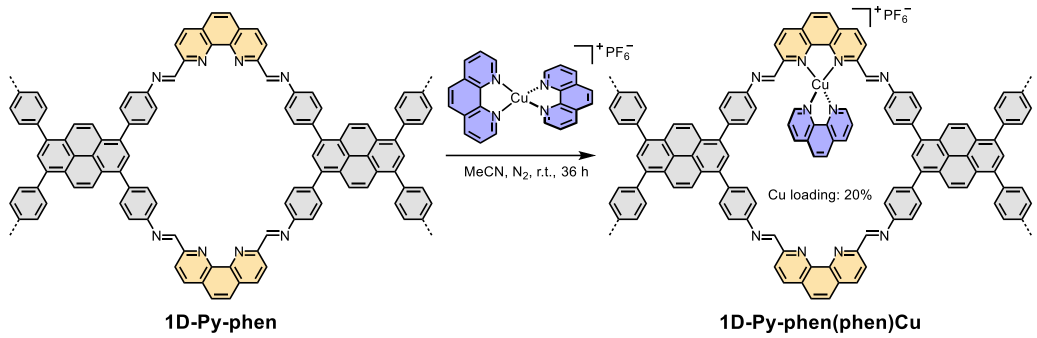

1D-Py-phen (30 mg, 62 μmol based on **phen-CHO**) was immersed in a 3.0 mL MeCN solution of [Cu(phen)$_2$](PF_6) (70.5 mg, 124 μmol) and stirred under N_2 atmosphere at room temperature for 36 hours. After reaction completion, the resulting solid was isolated by filtration and washed with a large amount of MeCN, then dried at room temperature under vacuum for 5 minutes to yield the **1D-Py-phen(phen)Cu**. ICP-AES analysis indicated a Cu loading of 1.4 wt%.

5. Characterizations of COF Materials

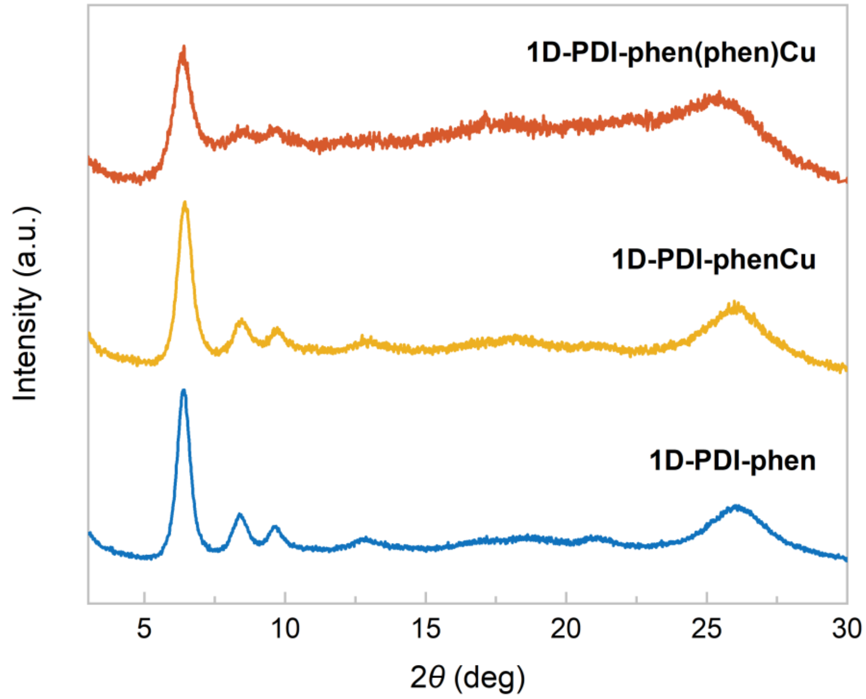

Figure S1. PXRD patterns of **1D-PDI-phen**, **1D-PDI-phenCu**, and **1D-PDI-phen(phen)Cu**.

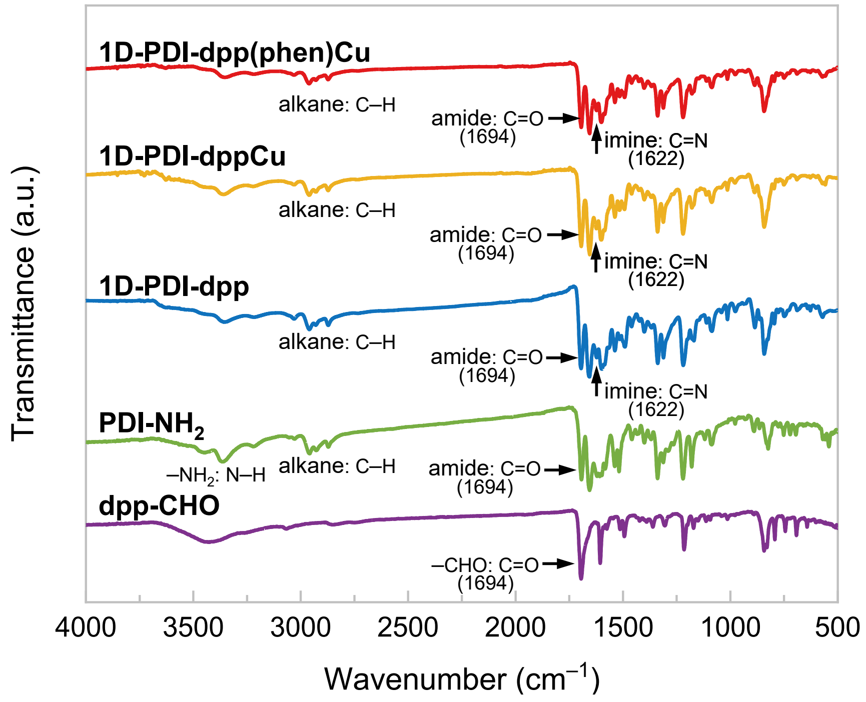

Figure S2. FT-IR spectra of **dpp-CHO**, **PDI-NH$_2$**, **1D-PDI-dpp**, **1D-PDI-dppCu** and **1D-PDI-dpp(phen)Cu**.

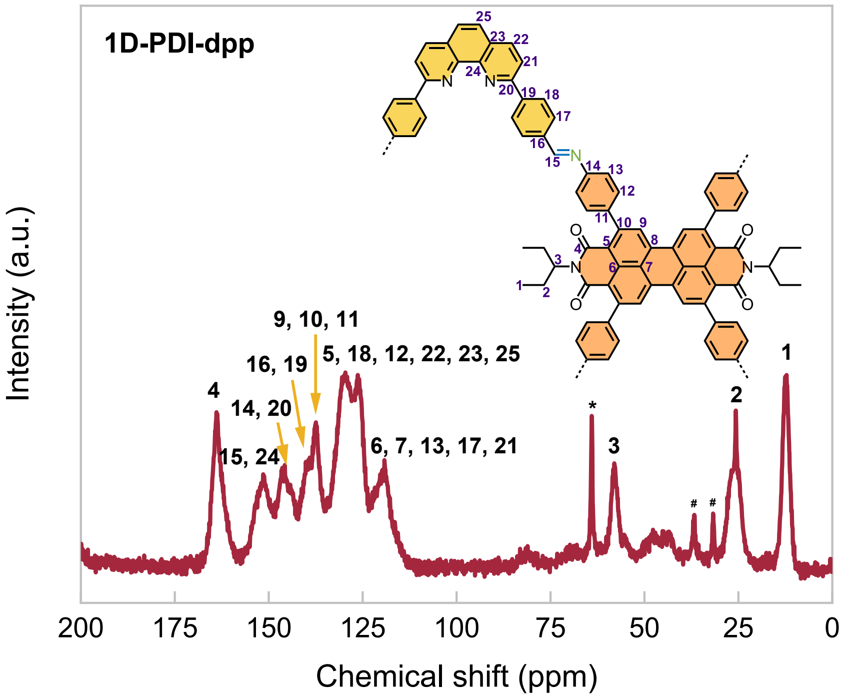

Figure S3. ^{13}C CP-MAS NMR spectrum of **1D-PDI-dpp**. The assignments of the ^{13}C chemical shifts are shown in the chemical structure ('*': peak assigned to 1,4-dioxane; '#': peaks assigned to DMF).

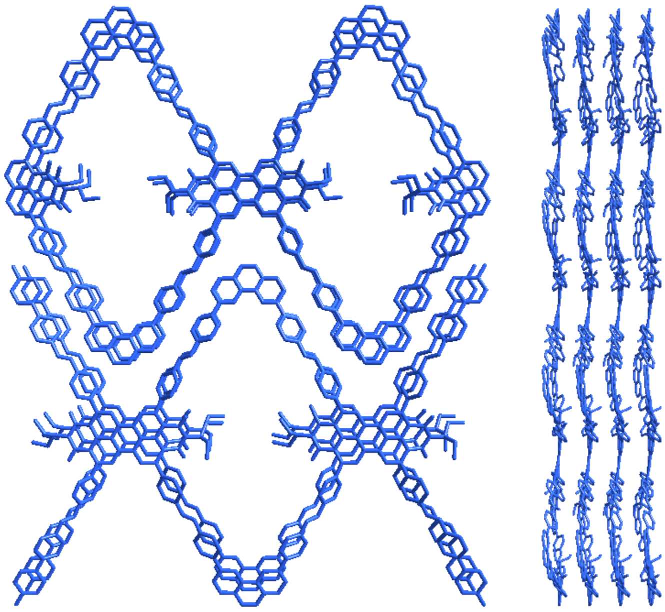

Figure S4. Simulated framework structure of **1D-PDI-dpp** in an AA-eclipsed stacking mode viewed from the extended plane and along the 1D chain direction.

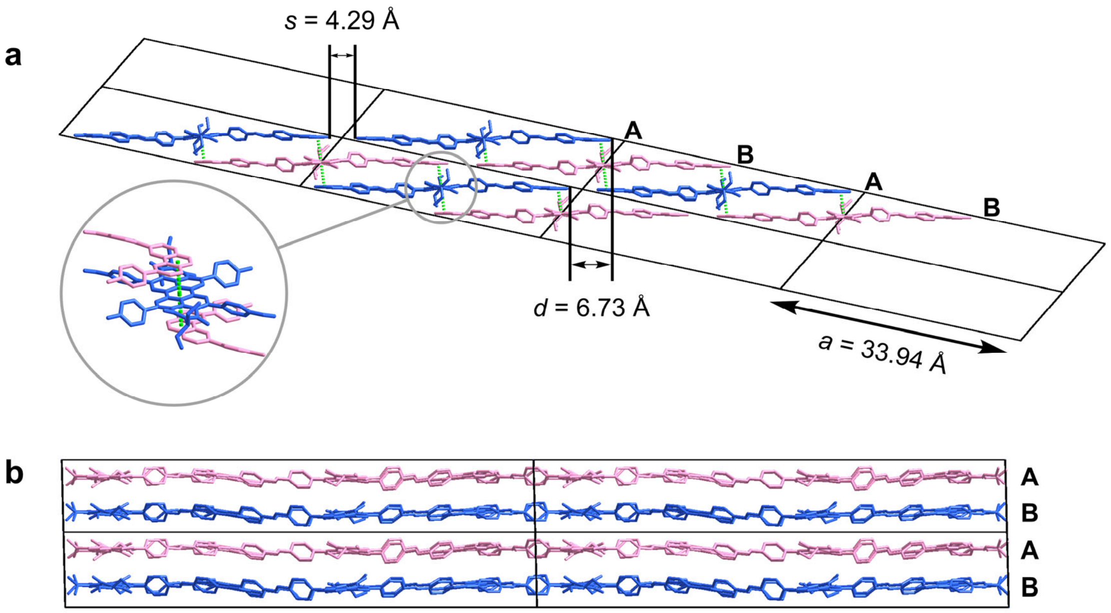

Figure S5. (a) Side view of **1D-PDI-dpp** adopting the simulated AB-slipped stacking mode; the enlarged inset highlights the strong π-π interactions (green dash lines) between PDI and phen units. (b) Side view of **1D-PDI-dpp** adopting the simulated AB-staggered stacking mode.

Table S2. Fractional Atomic Coordinates for the Unit Cell of 1D-PDI-dpp with AB Stacking

Crystal system		Monoclinic	
Space group		$P1$	
Unit cell		a = 33.50 Å, b = 19.10 Å, c = 9.00 Å<br>$\alpha = \gamma = 90°$, $\beta = 120°$	
Rietveld refinement		R_{wp} = 3.76%, R_p = 2.83%	
Atom	x	y	z
C1	-4542	5361.3	9612.9
C2	-4233.6	5716.6	9259.3
C3	-3902.2	5339.2	9060.2
C4	-4253.1	6442.9	9065.9
C5	-3942.5	6783.6	8698.6
C6	-3611	6392.4	8540.5
N7	-3602.4	5684.9	8721.2
C8	-3273	6727.2	8167.8
C9	-648	8504.3	5466.1
C10	-619.8	9233.2	5583.4
C11	-315.4	9616.1	5273.2
C12	-357.8	8138	4987.4
C13	-400.5	7374.4	4592.6
C14	-983.3	8176.1	5883.5
C15	-1389.9	8538.3	5484.7
C16	-1705.6	8262.3	5905.8
C17	-1621.4	7614.6	6741.3
C18	-1211.5	7258	7186.8
C19	-891.9	7541.1	6782
O20	-756.7	7065.2	4221.7
C21	-3312.1	7433.2	7682.7
C22	-2994	7731.7	7292.3
C23	-2631.8	7330	7394.3
C24	-2589.8	6626.6	7887.7
C25	-2908.6	6328.2	8267.3
N26	-1943.3	7316.8	7170.3
C27	-2304.7	7651	6965.4
C28	468.1	371.9	9721.1
C29	790.8	746.7	9483.8
C30	1107.4	386.2	9166.4
C31	799.7	1477.5	9519.3
C32	1122.4	1838.3	9252.8
C33	1438.1	1461.3	8971.1
N34	1419.9	750.2	8934.3
C35	1784.5	1813.1	8676.1
C36	4395.9	3551.3	5868.4

C37	4403.8	4281.2	5798.7
C38	4695.8	4642.3	5383.8
C39	4693.3	3160.6	5465.8
C40	4664.2	2386.6	5219.3
C41	4069.8	3248.8	6370.4
C42	3667.3	3621.1	5986.2
C43	3354.2	3359.7	6438.2
C44	3436.9	2716.1	7288.7
C45	3844.2	2352.7	7732.6
C46	4161.2	2622.3	7300.3
O47	4309.1	2075.7	4858.8
C48	1746.5	2523.7	8230.6
C49	2066.2	2832.5	7862.9
C50	2428.6	2435.7	7951.1
C51	2470.4	1728.7	8413.9
C52	2149.8	1420.2	8767.4
N53	3114.6	2424.6	7721.8
C54	2754.9	2761.2	7525.7
C55	4526	5367.4	244.2
C56	4205	5745.1	485.3
C57	3886.1	5387.5	795.4
C58	4200	6476.1	460.7
C59	3878.5	6839.9	728.4
C60	3560.1	6465.8	1000.2
N61	3574.9	5754.5	1028.7
C62	3213.8	6820.5	1290.4
C63	595.9	8552.3	4070.8
C64	588.3	9281.9	4152
C65	296.6	9640.2	4572.1
C66	297.6	8158.9	4464.4
C67	325.5	7384.4	4697
C68	923.2	8253.5	3569.9
C69	1325.5	8627.9	3960.1
C70	1640	8369.4	3511.5
C71	1558.9	7726.6	2658.8
C72	1152	7361.2	2209.4
C73	833.5	7628	2637.7
O74	680.3	7073.4	5055.1
C75	3252.8	7531.3	1735.2
C76	2932.7	7842.2	2098.3
C77	2568.9	7447.5	2005.3
C78	2526.2	6740.5	1541.8
C79	2847	6429.9	1193.1
N80	1882.4	7437.2	2229.4
C81	2242.2	7774	2427.9
C82	9536.9	361.4	353.7

C83	9230.5	718.9	714.2
C84	8897.3	344.2	907.6
C85	9253.7	1444.7	919.5
C86	8944.4	1787.8	1290.5
C87	8610.7	1399.3	1440.8
N88	8599	692.1	1250.8
C89	8273.1	1736.5	1811.8
C90	5640	3513.3	4473.8
C91	5612.3	4242.6	4368.9
C92	5307.9	4623.2	4683.3
C93	5348.6	3143.8	4941.9
C94	5389.8	2378.5	5320.8
C95	5976.9	3188.4	4059.4
C96	6384.3	3551.2	4470.4
C97	6702	3276.9	4056.4
C98	6618.9	2630.4	3216.6
C99	6208.3	2273.7	2758.6
C100	5886.9	2555.1	3155.2
O101	5745.4	2069	5685.7
C102	8313.8	2442.3	2299.5
C103	7995.8	2743	2687.6
C104	7632.1	2343.6	2579.9
C105	7588.4	1640.5	2083
C106	7907.1	1339.9	1706.2
N107	6942.3	2333.3	2794
C108	7304.7	2666.4	3005.6
C109	4538.4	4634.8	356.4
C110	4229.7	4279.9	709
C111	3898.1	4657.7	906.2
C112	4249	3553.7	903.5
C113	3938.1	3213.3	1269.6
C114	3606.3	3604.8	1425.3
N115	3597.9	4312.3	1244
C116	3267.9	3270.2	1795.9
C117	640.5	1488.5	4483.1
C118	612.9	759.4	4369
C119	308.7	376.2	4679.9
C120	349.6	1854.9	4958.8
C121	390.5	2619.3	5345.1
C122	976.1	1817	4067.2
C123	1383.2	1455.5	4469.6
C124	1699.6	1732.5	4053
C125	1615.7	2380.5	3219
C126	1205.2	2736.1	2769.1
C127	884.9	2452	3168.8
O128	746	2929.9	5713.8

C129	3306.9	2564.1	2280.7
C130	2988.7	2265.5	2670.3
C131	2626.4	2667.2	2567.9
C132	2584.4	3370.6	2074.3
C133	2903.4	3669.2	1695.5
N134	1937.9	2679.4	2792.8
C135	2299.3	2345.5	2996
C136	9520.4	9629.1	229.4
C137	9197.7	9254.3	466.9
C138	8881.4	9614.7	786.1
C139	9188.5	8523.4	429.6
C140	8866	8162.6	697.3
C141	8550.8	8539.5	981.3
N142	8569	9250.6	1018.7
C143	8204.9	8187.6	1279
C144	5596.1	6450.3	4102
C145	5588.1	5720.4	4171.9
C146	5296.3	5359.5	4587.6
C147	5298.9	6841.1	4505.8
C148	5328.2	7614.9	4753.2
C149	5921.9	6752.6	3598.2
C150	6324.1	6379.9	3980.2
C151	6636.9	6641	3526.8
C152	6554.4	7284.7	2676.9
C153	6147.3	7648.4	2234.7
C154	5830.5	7379.2	2668.7
O155	5683.3	7925.7	5112.4
C156	8243.3	7477	1725.2
C157	7924.1	7168.3	2096.1
C158	7562	7565	2010.4
C159	7519.9	8271.9	1546.8
C160	7839.8	8580.4	1190.1
N161	6876.5	7575.9	2242.5
C162	7236.1	7239.4	2437.9
C163	-4529.5	4628.7	9724.1
C164	-4208.6	4251.3	9481.4
C165	-3890	4609.3	9170.4
C166	-4203.2	3520.4	9506.5
C167	-3881.9	3157	9236.8
C168	-3564	3531.5	8962.7
N169	-3578.9	4242.8	8935.4
C170	-3218.3	3177	8668.6
C171	-603	1439.7	5881
C172	-595.1	710.1	5801.3
C173	-303.2	352	5381.2
C174	-305.3	1833.2	5484.6

C175	-334.9	2607.5	5245
C176	-929.8	1738.8	6383.1
C177	-1332.7	1365.9	5990.7
C178	-1646.7	1625.2	6441.3
C179	-1564.3	2267.1	7298.4
C180	-1156.7	2630.9	7750.1
C181	-838.8	2363.4	7319.5
O182	-690	2917.4	4889
C183	-3257.8	2466.4	8221.9
C184	-2938.2	2155.2	7856.6
C185	-2574.4	2549.5	7949
C186	-2531.1	3256.4	8414.1
C187	-2851.4	3567.3	8764.9
N188	-1887.4	2557.5	7729
C189	-2248.2	2222.2	7525.7
C190	451.8	9639.6	9598.7
C191	758.5	9281.9	9240
C192	1091.7	9656.6	9046.4
C193	735.6	8556	9036.6
C194	1045	8212.8	8666.6
C195	1378.6	8601.3	8515.5
N196	1390.1	9308.6	8704.3
C197	1716.5	8264.3	8145.7
C198	4352.1	6488.4	5497.6
C199	4379.7	5759.2	5602.2
C200	4684.2	5378.6	5288.3
C201	4643.8	6857.9	5030.7
C202	4602.8	7623.3	4653.6
C203	4015.1	6813.3	5911
C204	3606.8	6451.6	5493.1
C205	3289	6725.6	5906.1
C206	3372.6	7370.8	6751.9
C207	3784	7726.4	7216.8
C208	4105.7	7445.2	6821
O209	4247.4	7933	4290.5
C210	1676.3	7558.2	7660
C211	1994.7	7257.7	7274.1
C212	2358.4	7657.3	7382
C213	2401.5	8360.6	7876.7
C214	2082.3	8661.1	8251.3
N215	3049	7667.7	7173.1
C216	2686.2	7334.6	6958.9
C217	-25.9	8516.9	4769.9
C218	-15.4	9257.6	4869.2
N219	-42.2	7020	4588.9
C220	5017.1	3520.8	5166

C221	5007.4	4262	5080.3
N222	5031	2023.1	5318.9
C223	18.4	1475.5	5179.9
C224	8.6	734.7	5083.3
N225	31.3	2972.8	5343.8
C226	4975.2	6480.9	4805.8
C227	4984.7	5739.8	4891.3
N228	4961.7	7978.6	4655.4
C229	4964.9	8749.5	4555.8
C230	5027.9	1252.2	5419.6
C231	-39.4	6249.4	4481.4
C232	25.9	3743.7	5435.6
C233	4575.2	9019.1	2850.6
C234	4654.9	8809.2	1394.4
C235	4946.1	9092.4	6047.8
C236	5390.4	8959.3	7716.4
C237	5046.5	908.9	3927.9
C238	4601.7	1040.2	2260.2
C239	5417.6	982.8	7125
C240	5337.2	1191.1	8580.6
C241	-427.3	5982.1	2769
C242	-345.8	6198.2	1320.7
C243	-61.8	5903.5	5963.4
C244	378.1	6042.2	7643.4
C245	43.3	4084.8	3939
C246	-398.1	3939.7	2268.9
C247	414.9	4017.2	7137.5
C248	336.2	3808.7	8597

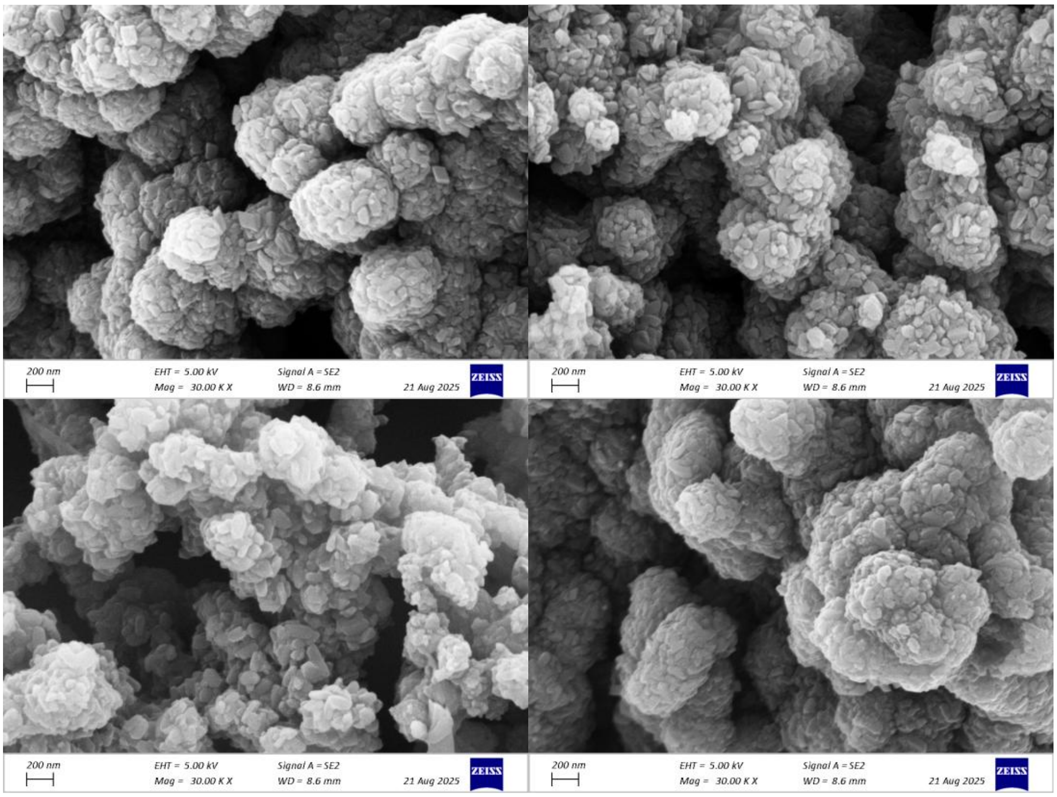

Figure S6. SEM images of **1D-PDI-dpp**.

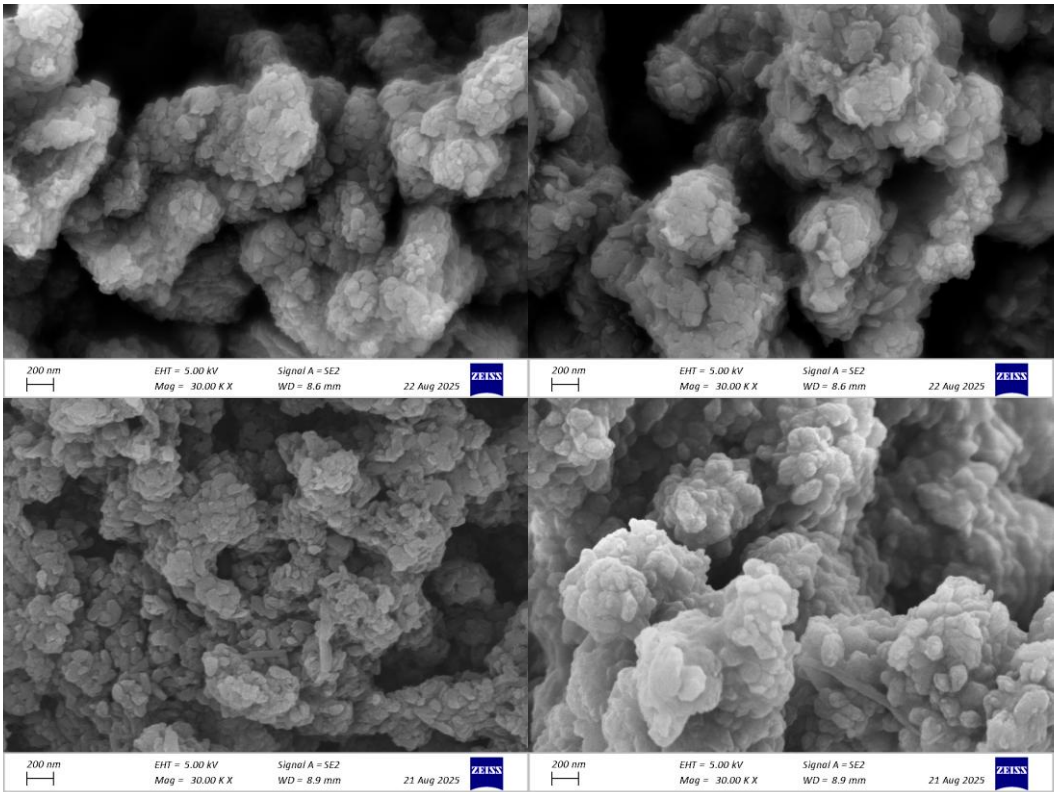

Figure S7. SEM images of **1D-PDI-dppCu**.

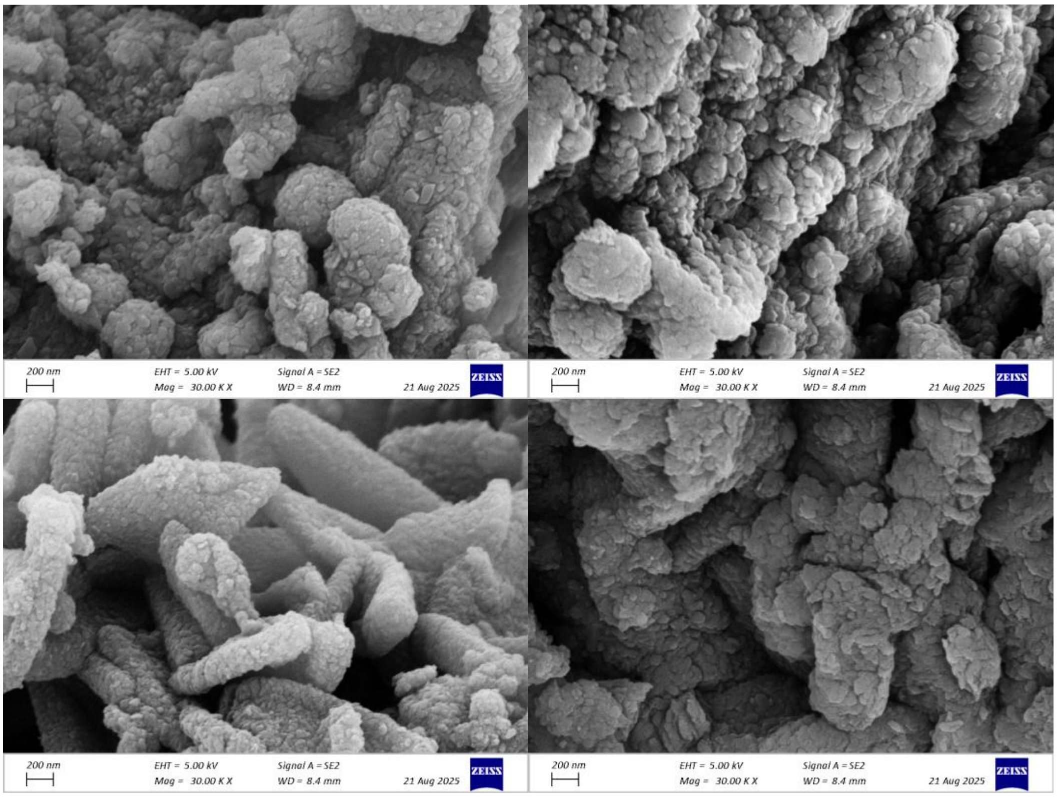

Figure S8. SEM images of **1D-PDI-dpp(phen)Cu**.

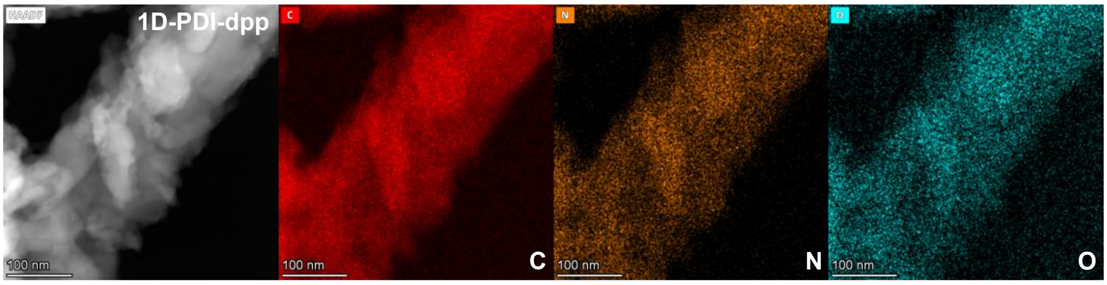

Figure S9. HADDF image and EDS mapping images of **1D-PDI-dpp**. EDS mapping images of C, N, and O are represented in red, orange, and cyan, respectively.

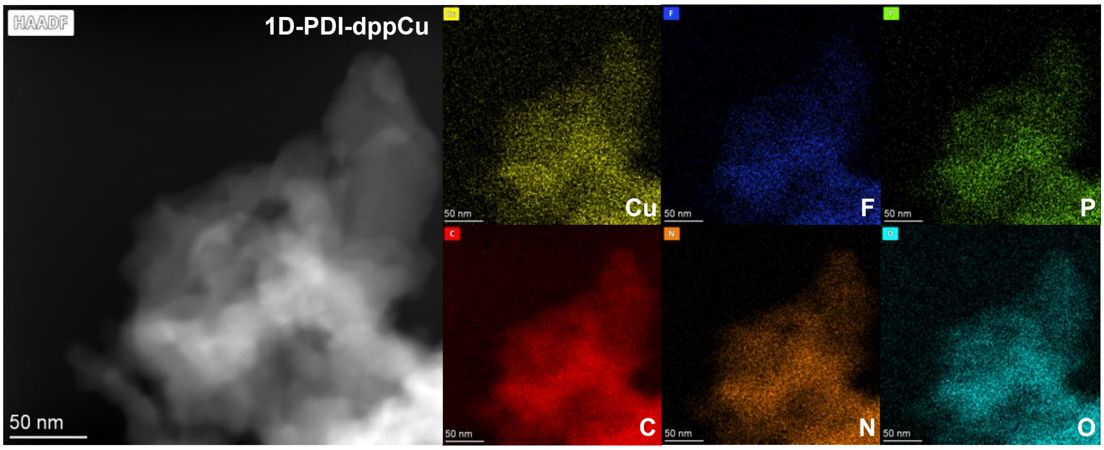

Figure S10. HADDF image and EDS mapping images of **1D-PDI-dppCu**. EDS mapping images of Cu, F, P, C, N, and O are represented in yellow, blue, green, red, orange, and cyan, respectively.

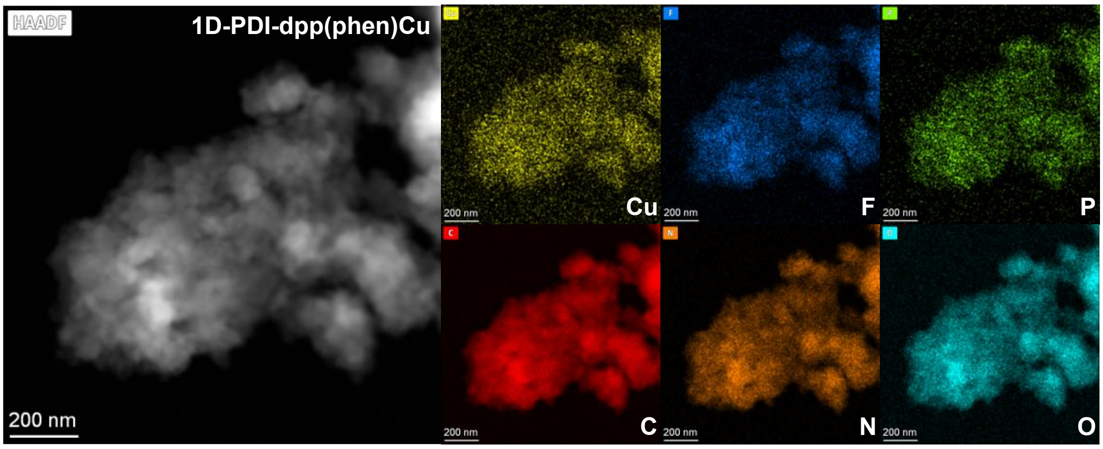

Figure S11. HADDF image and EDS mapping images of **1D-PDI-dpp(phen)Cu**. EDS mapping images of Cu, F, P, C, N, and O are represented in yellow, blue, green, red, orange, and cyan, respectively.

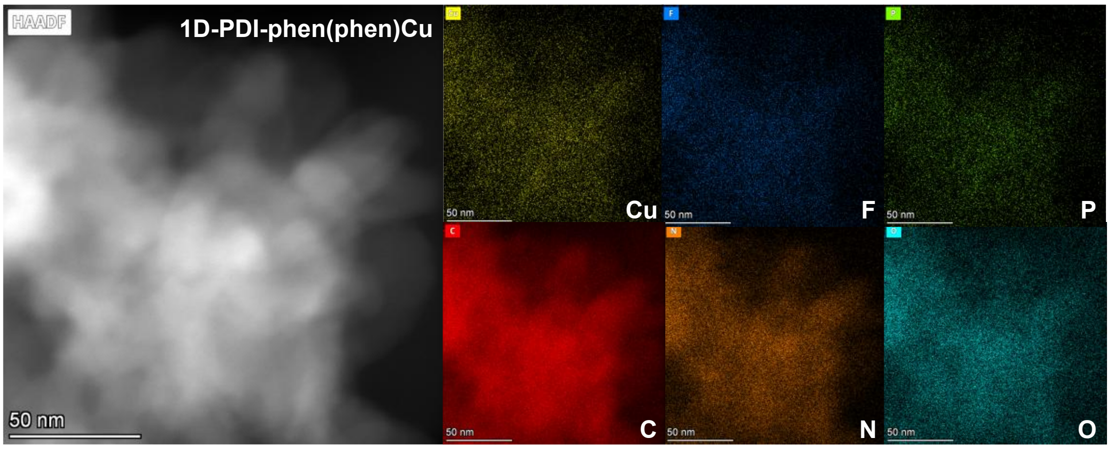

Figure S12. HADDF image and EDS mapping images of **1D-PDI-phen(phen)Cu**. EDS mapping images of Cu, F, P, C, N, and O are represented in yellow, blue, green, red, orange, and cyan, respectively.

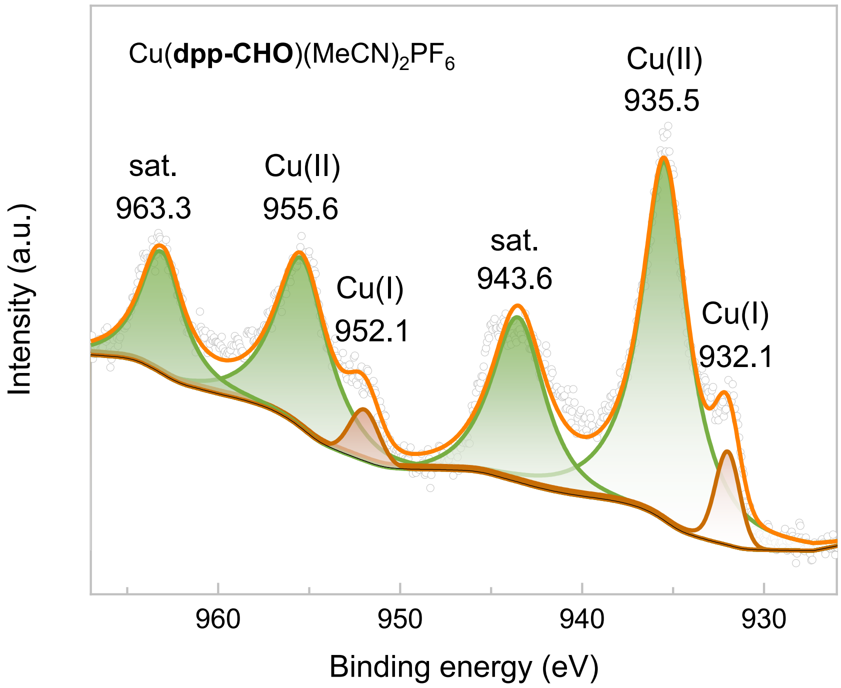

Figure S13. Cu2*p* XPS spectrum of measured Cu(**dpp-CHO**)(MeCN)$_2$PF$_6$.

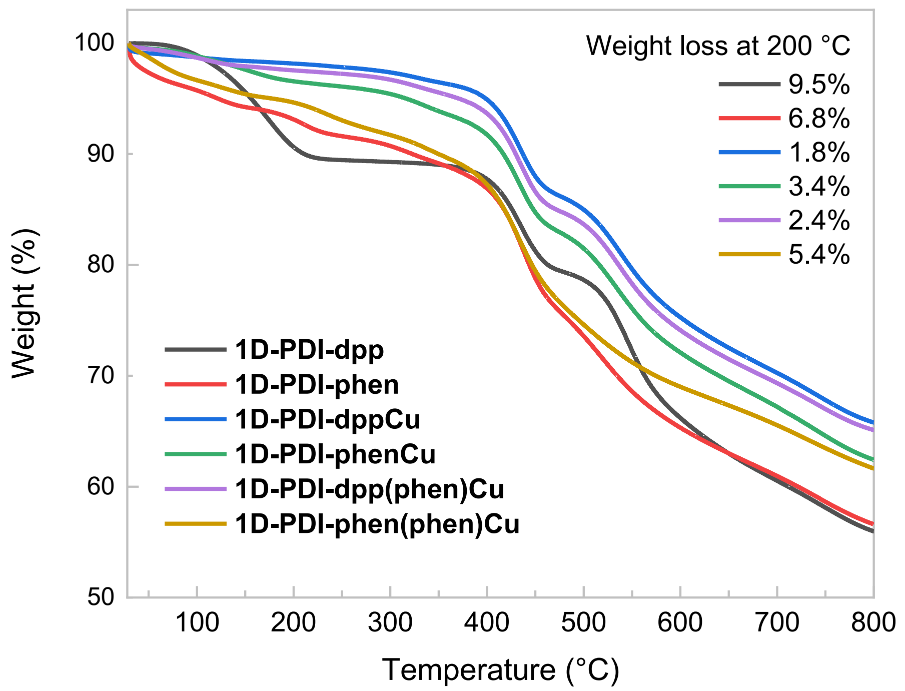

Figure S14. TGA plots of PDI-based COF materials under N_2 atmosphere.

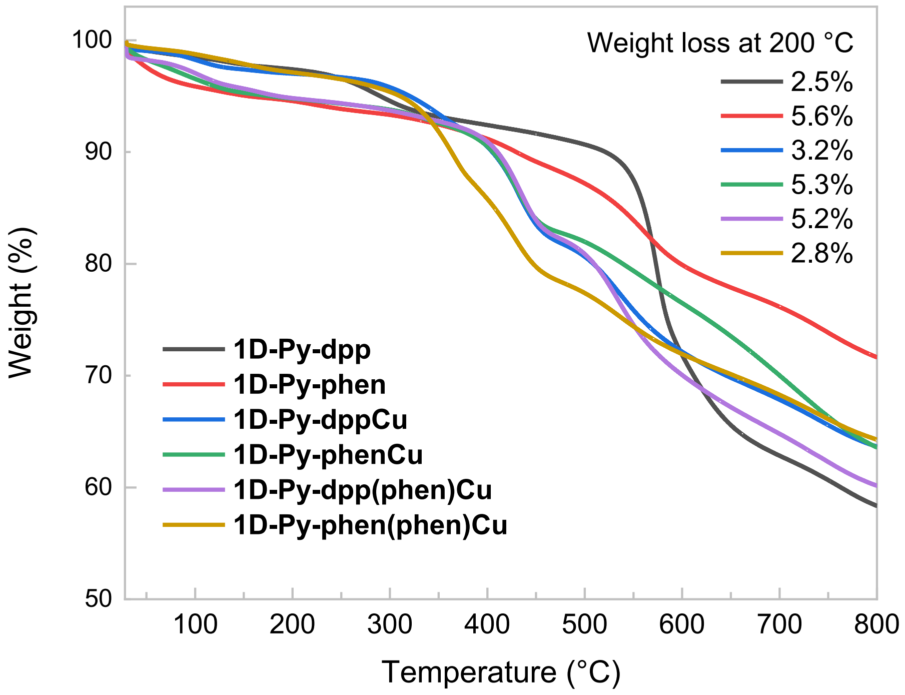

Figure S15. TGA plots of pyrene-based COF materials under N_2 atmosphere.

NMR digestion experiment of 1D-PDI-dpp(phen)Cu

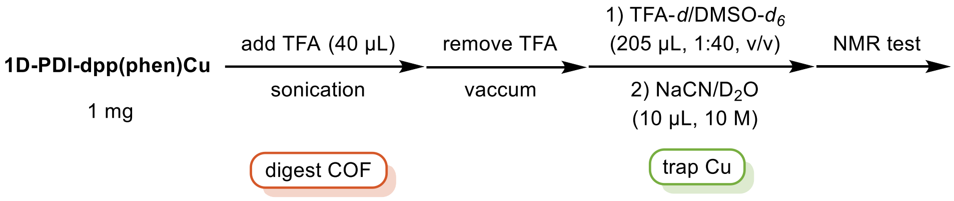

1 mg of the **1D-PDI-dpp(phen)Cu** powder was digested in 40 μL of trifluoroacetic acid (TFA). The TFA was then removed under vacuum. The residue was dissolved in a 205 μL mixture of trifluoroacetic acid-*d* and DMSO-d_6 (1:40, v/v) solution. To trap copper ions, 10 μL of NaCN (10 M) in D_2O was added to the solution. Finally, the resulting solution was analyzed by ^{1}H NMR spectroscopy.

According to the ^{1}H NMR spectrum, the ratio of **PDI-NH$_2$** and phen was found to be 1:0.36. The calculated metalation efficiency is 0.36/2 × 100% = 18%, matched with ICP-AES analysis results (0.95 wt%, 18% metalation efficiency).

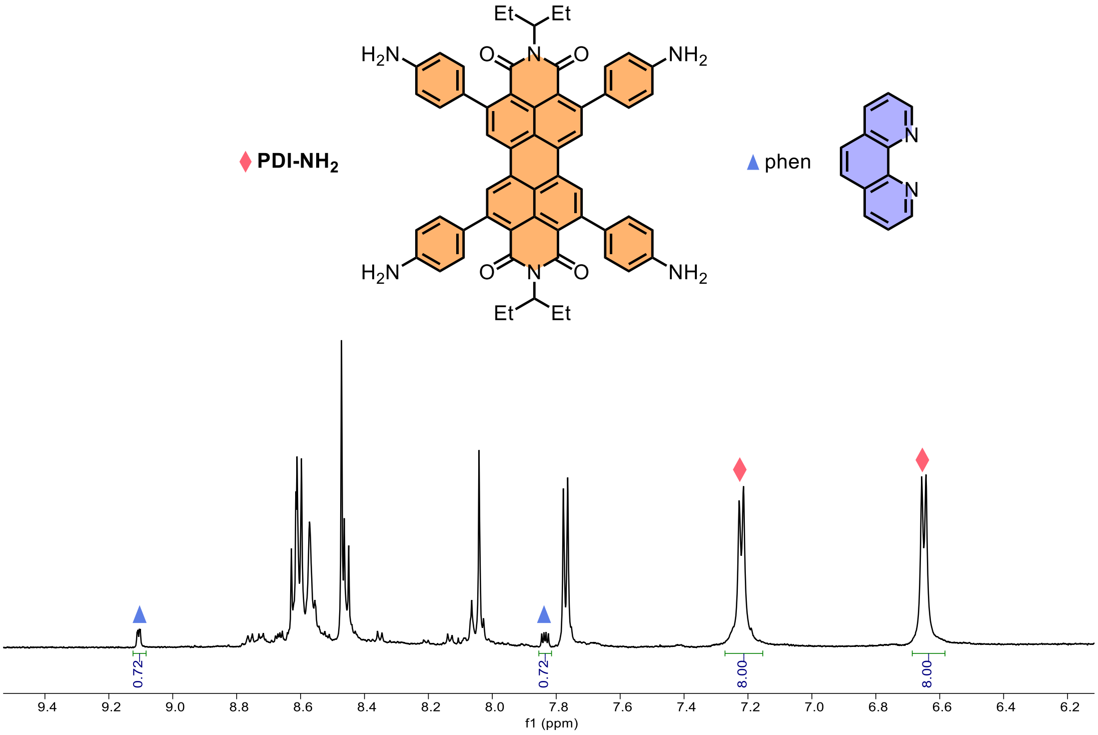

Figure S16. NMR analysis of the digested sample of **1D-PDI-dpp(phen)Cu**.

TGA data showed a 2.4% weight loss below 200 °C (Figure S14), attributed to the residual solvent in **1D-PDI-dpp(phen)Cu**. Theoretically, the minimal repeating unit of **1D-PDI-dpp(phen)Cu** was determined to be [$C_{67}H_{45}Cu_1F_6N_7O_2P$] with a molecular weight of 1188.7 g mol^{-1}. Including the solvent content, the molecular weight of **1D-PDI-dpp(phen)Cu** should be adjusted as follows: 1188.7/(1 – 2.4%) ≈ 1217.9 g mol^{-1}. Consequently, 1 mg of **1D-PDI-dpp(phen)Cu** corresponds to a theoretical Cu content: (63.5/1217.9) × 100% = 5.2 wt%. ICP-AES analysis showed an actual Cu loading of 0.95 wt%. Therefore, the metalation efficiency is approximately 0.95 wt%/5.2 wt% * 100% ≈ 18%.

Table S3. NMR Digestion and ICP Data of 1D-PDI-dpp(phen)Cu before and after Photocatalysis

	NMR digestion analysis for		ICP analysis for	
	**PDI-NH$_2$**/**dpp-CHO**/phen	**dpp-CHO**/phen	Cu loading (wt%)[a]	Metalation efficiency (%)[a]
After metalation (before reaction)	1:2:0.36	1:0.18	0.95	18
After reaction	1:2:0.35	1:0.18	0.95	18

[a]Copper loadings were determined by ICP-AES.

The amount of the COF-supported catalysts used in each reaction was determined based on ICP-AES. For example, the corresponding Cu content of **1D-PDI-dpp(phen)Cu** was 0.95 wt%, which means that 1 mg of **1D-PDI-dpp(phen)Cu** contains 0.0095 mg (0.15 μmol) of Cu catalyst. To run a catalytic reaction (on a 0.1-mmol scale) with 0.1 mol% of the Cu catalyst: the amount of **1D-PDI-dpp(phen)Cu** = (0.1 mmol × 0.1 mol%) × 63.5 g mol^{-1}/0.0095 mg = 0.67 mg.

NMR Digestion Experiment of 1D-PDI-dppCu

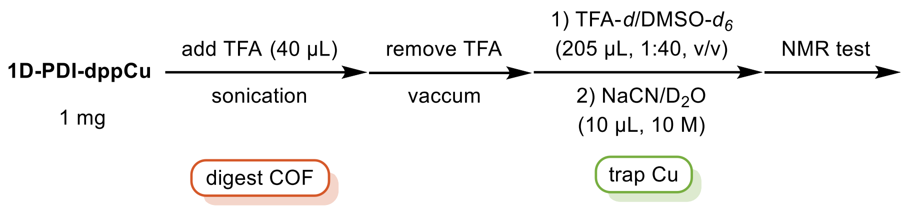

1 mg of the **1D-PDI-dppCu** powder was digested in 40 μL of trifluoroacetic acid (TFA). The TFA was then removed under vacuum. The residue was dissolved in a 205 μL mixture of trifluoroacetic acid-*d* and DMSO-d_6 (1:40, v/v) solution. To trap copper ions, 10 μL of 10 M NaCN in D_2O was added to the solution. Finally, the resulting solution was analyzed by ^1H NMR spectroscopy.

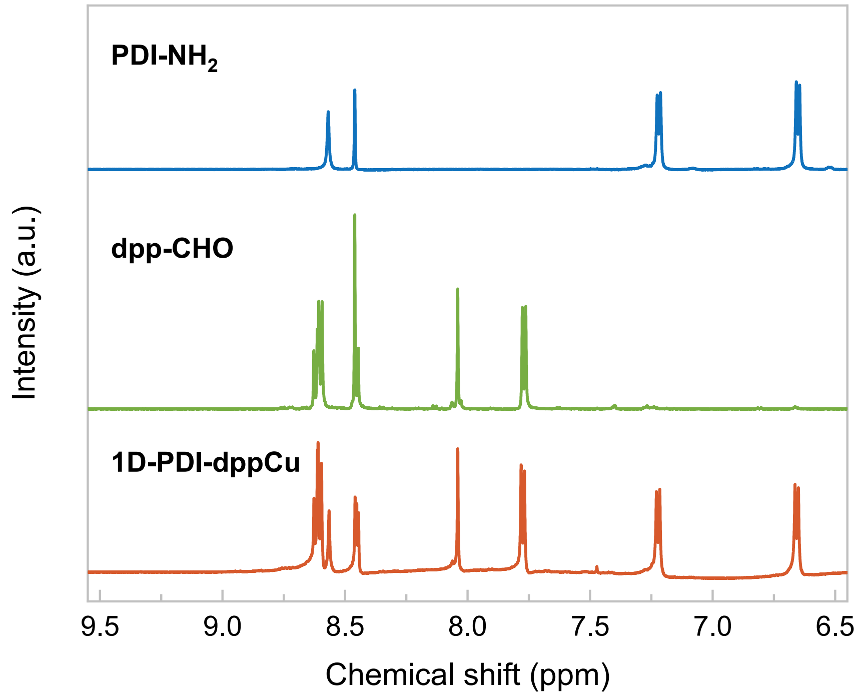

Figure S17. ^1H NMR spectra of **PDI-NH$_2$**, **dpp-CHO**, and **1D-PDI-dppCu** under digestion conditions.

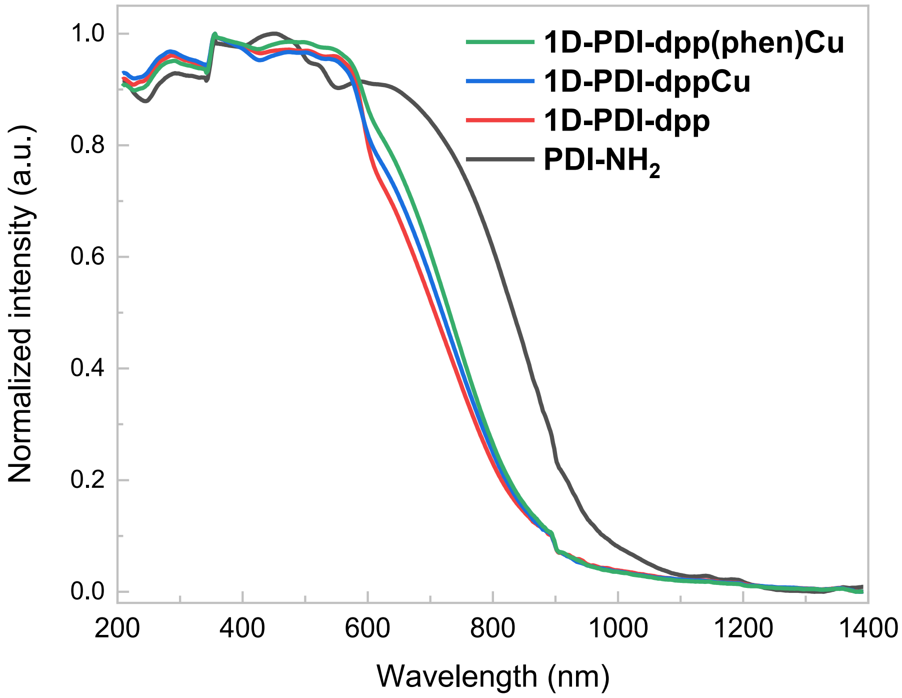

Figure S18. Solid-state absorption spectra of **PDI-NH₂**, **1D-PDI-dpp**, **1D-PDI-dppCu**, and **1D-PDI-dpp(phen)Cu**.

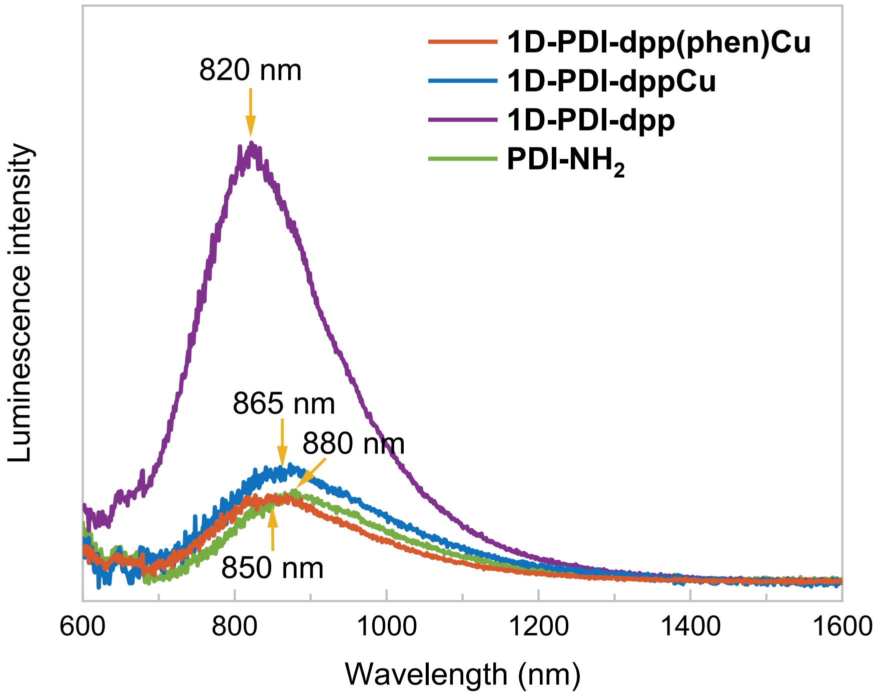

Figure S19. Solid-state emission spectra (excitation wavelength of 525 nm) of **PDI-NH₂**, **1D-PDI-dpp**, **1D-PDI-dppCu**, and **1D-PDI-dpp(phen)Cu**.

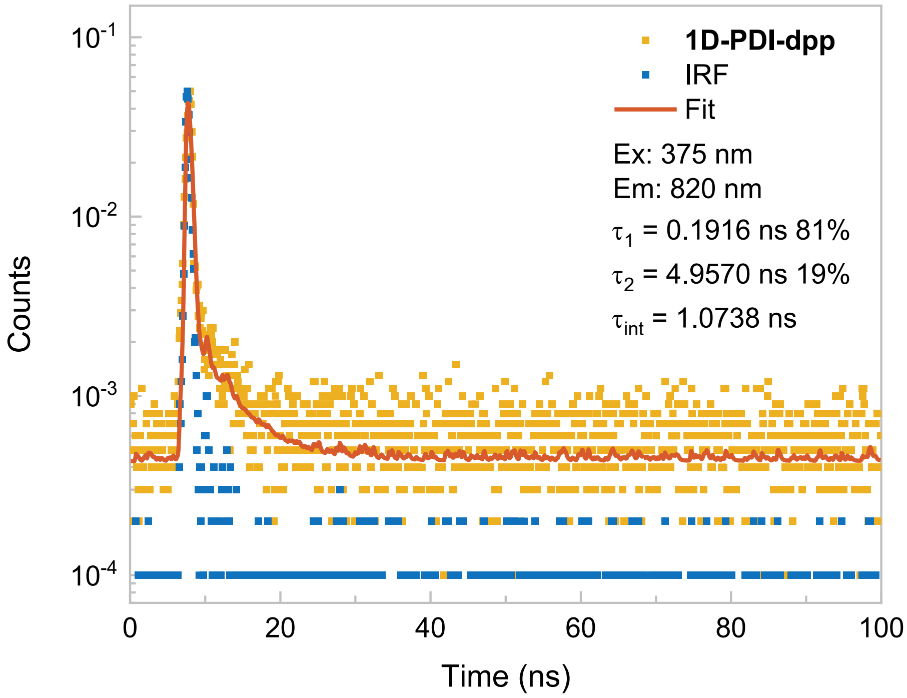

Figure S20. Emission decay of **1D-PDI-dpp** measured at the excitation wavelength of 375 nm and emission wavelength of 820 nm.

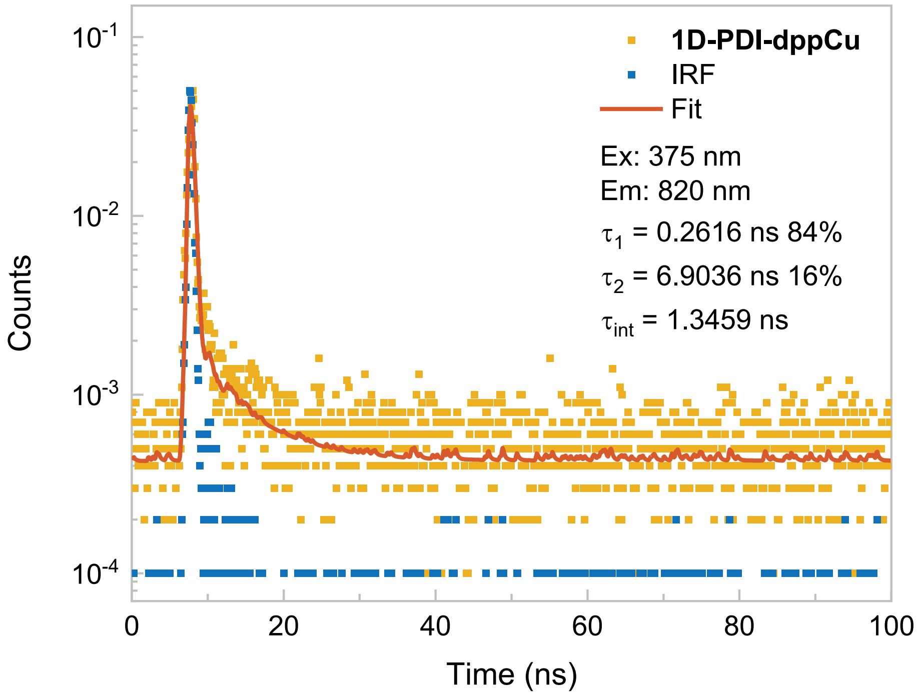

Figure S21. Emission decay of **1D-PDI-dppCu** measured at the excitation wavelength of 375 nm and emission wavelength of 820 nm.

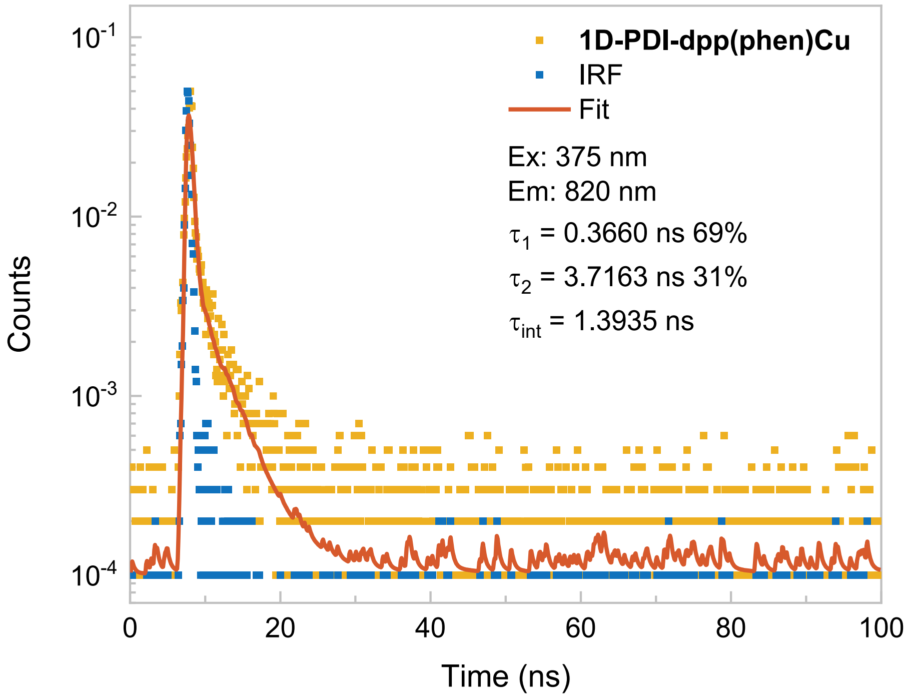

Figure S22. Emission decay of **1D-PDI-dpp(phen)Cu** measured at the excitation wavelength of 375 nm and emission wavelength of 820 nm.

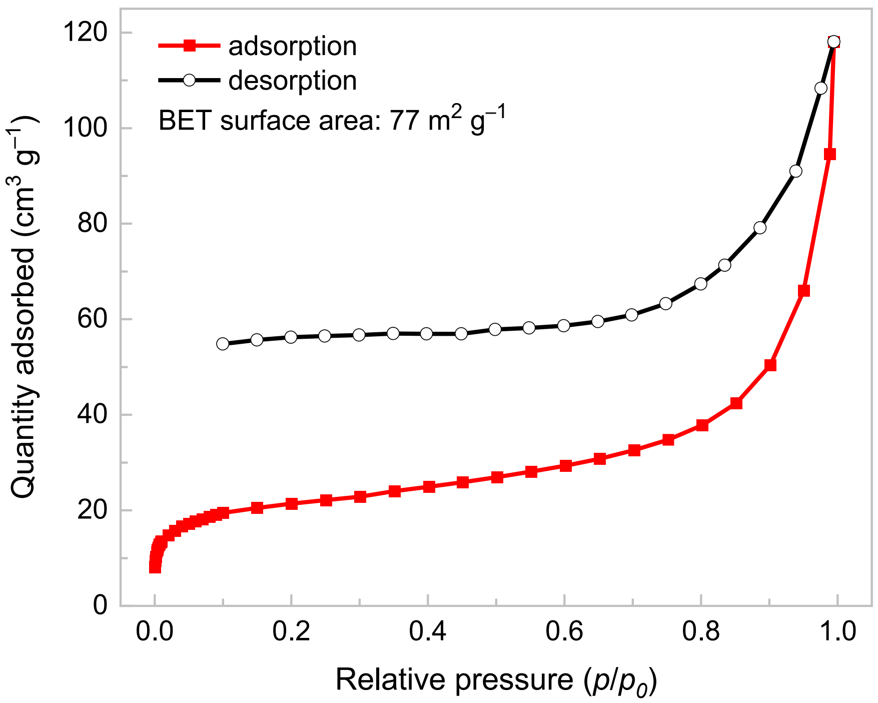

Figure S23. N_2 adsorption/desorption isotherms of **1D-PDI-dpp** collected at 77 K.

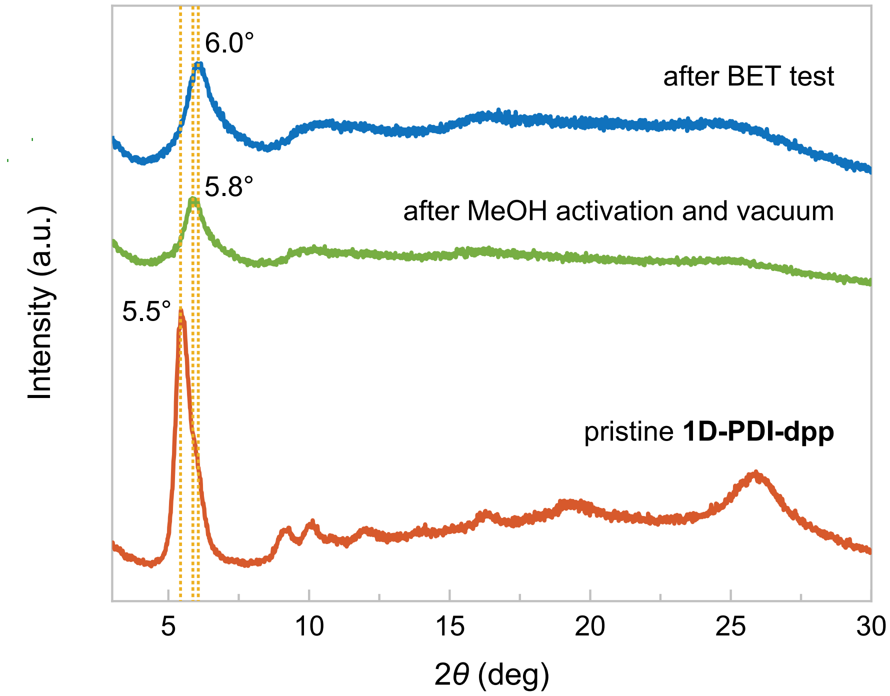

Figure S24. PXRD patterns of pristine **1D-PDI-dpp**, after activation, and after the BET test.

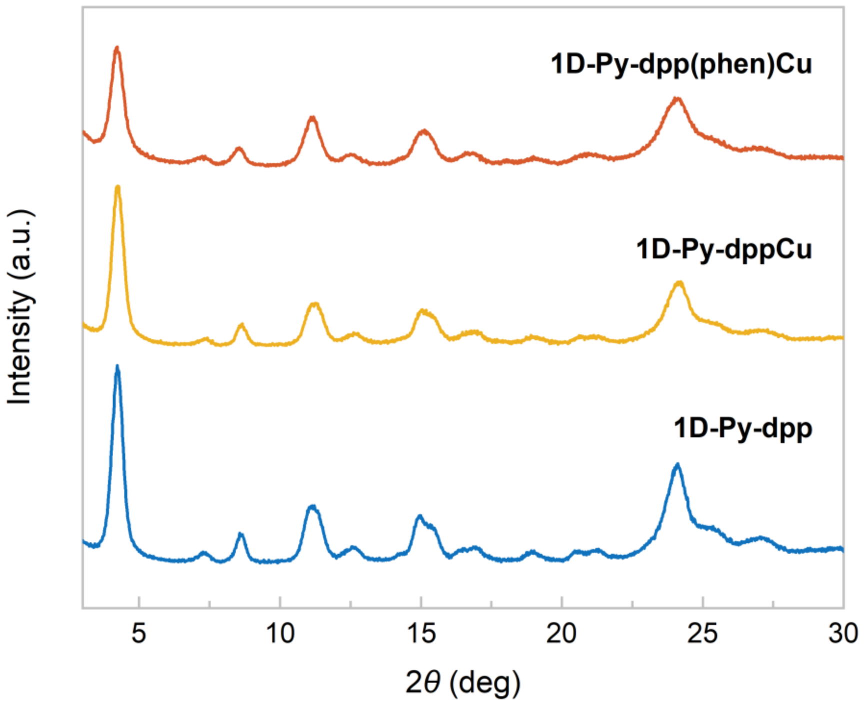

Figure S25. PXRD patterns of **1D-Py-dpp**, **1D-Py-dppCu** and **1D-Py-dpp(phen)Cu**.

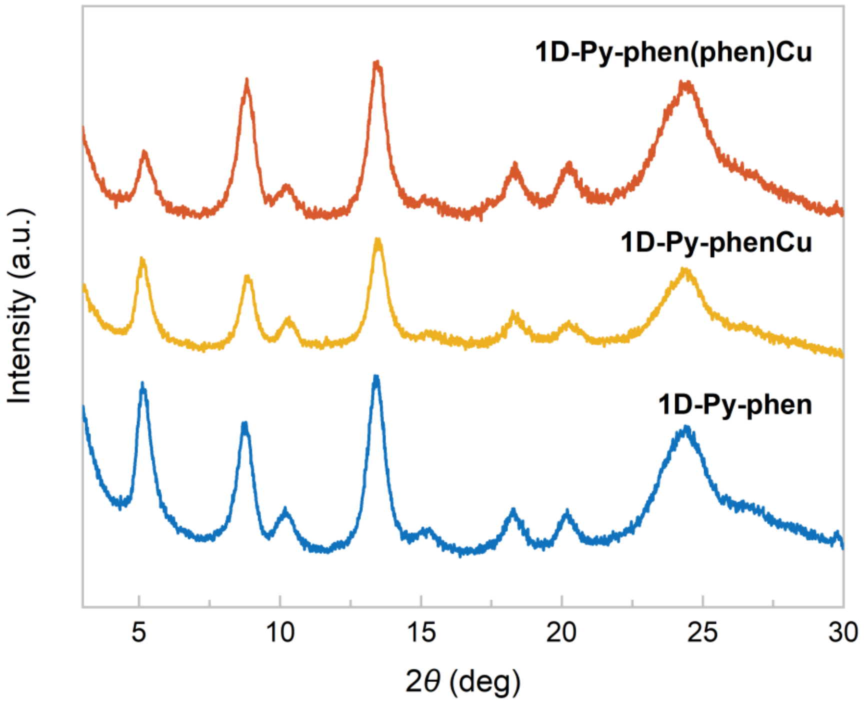

Figure S26. PXRD patterns of **1D-Py-phen**, **1D-Py-phenCu** and **1D-Py-phen(phen)Cu**.

6. Photocatalytic Applications of COF Materials

6.1 Condition Screening for Heterogeneous Copper Catalysis

General Procedure A. To a 20-mL flame-dried glass tube were added **1D-PDI-dpp(phen)Cu** (0.67 mg, 0.1 mol%), styrene (0.10 mmol, 1.0 equiv.), $TMSN_3$ (46.1 mg, 0.40 mmol, 4.0 equiv.) and MeCN (0.4 mL) sequentially. The reaction mixture was irradiated by two 40-watt Kessil PR160L-525 green-LED lamps at room temperature (with one fan) for 12 hours. After irradiation, 1,1,2,2-tetrachloroethane as an internal standard (0.1 mmol, 10.4 μL) was added into the crude mixture. A volume of 50 μL of the crude mixture was taken and diluted with 450 μL of $CDCl_3$. The resulting solution was subsequently filtered through a syringe filter prior to the NMR analysis.

Table S4. Effects of Various Reaction Parameters on the Oxo-Azidation of Styrene[a]

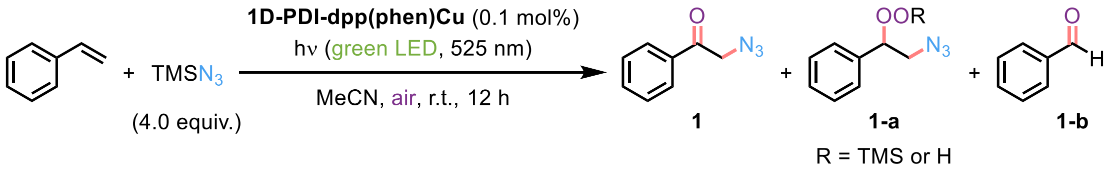

Entry	Change from the "standard conditions"	Yield of **1** (%)	Yield of **1-a** (%)	Yield of **1-b** (%)	Styrene conversion (%)
1	none	75	<5	21	>95
2	no catalyst	<5	<5	<5	<5
3	w/o light, r.t.	<5	<5	<5	<5
4	w/o light, 80 °C	<5	<5	<5	<5
5	under nitrogen, instead of air	<5	<5	<5	<5
6	456 nm, instead of 525 nm	55	<5	15	>95
7	467 nm, instead of 525 nm	66	<5	21	>95
8	660 nm, instead of 525 nm	61	<5	17	92
9	DCM, instead of MeCN	70	<5	20	>95
10	toluene, instead of MeCN	20	<5	10	38
11	MeOH, instead of MeCN	48	<5	17	76

12	DMF, instead of MeCN	13	27	5	48
13	$CHCl_3$, instead of MeCN	10	<5	5	20
14	1,4-dioxane, instead of MeCN	6	<5	<5	11
15	THF, instead of MeCN	6	<5	<5	8
16	$TMSN_3$ (2 equiv.)	67	<5	20	>95
17	MeCN (0.125 M, 0.8 mL)	53	30	17	>95
18	MeCN (0.50 M, 0.2 mL)	70	11	17	>95

[a]Standard conditions: styrene (0.10 mmol, 1.0 equiv.), $TMSN_3$ (4.0 equiv.), **1D-PDI-dpp(phen)Cu** (0.1 mol%) in MeCN (0.4 mL) under air atmosphere at room temperature for 12 h. Yield was determined by ^1H NMR using 1,1,2,2-tetrachloroethane as an internal standard.

6.2 Synthesis of Substrates

The alkenes **S-1**,[6] **S-2**,[6] **S-3**,[7] **S-4**,[8] **S-5**,[6] **S-6**[9] were synthesized according to the literature.

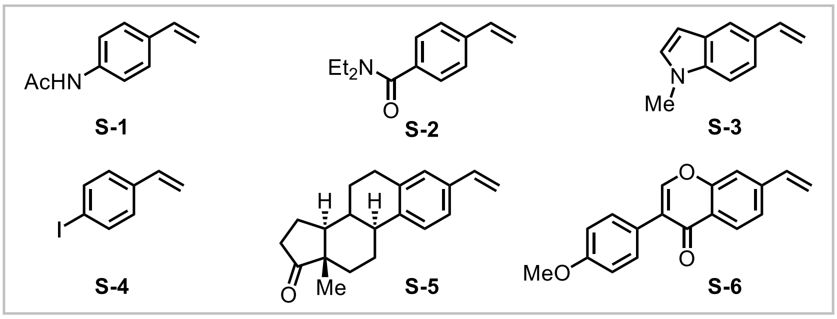

6.3 Heterogeneous Photoinduced Oxo-Azidation of Styrenes

General Procedure B. To a 20-mL flame-dried glass tube were added **1D-PDI-dpp(phen)Cu** (0.67 mg, 0.1 mol%), olefin (0.10 mmol, 1.0 equiv.), $TMSN_3$ (46.1 mg, 0.40 mmol, 4.0 equiv.) and MeCN (0.4 mL) sequentially. The reaction mixture was irradiated by two 40-watt Kessil PR160L-525 green-LED lamps at room temperature (with one fan) for 12 hours. After irradiation, ethyl acetate (5 mL) was added into the crude mixture; the resulting solution was filtered through a pad of silica gel and washed three times with ethyl acetate (3 × 5 mL), dried over Na_2SO_4 and evaporated under reduced pressure. The residue was purified by flash column chromatography on silica gel to afford the corresponding product.

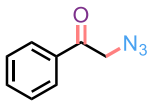

2-Azido-1-phenylethan-1-one (1). The title compound was synthesized according to General Procedure B using styrene (10.4 mg, 0.10 mmol, 1.0 equiv.). The crude mixture was purified by flash column chromatography using hexanes-ethyl acetate (30:1 v/v) as the eluent to give the title compound as a colorless oil 12.1 mg (0.075 mmol, 75% yield).

^{1}H NMR ($CDCl_3$, 600 MHz): δ 7.91 (dd, J = 8.4, 1.2 Hz, 2H), 7.63 (t, J = 7.8 Hz, 1H), 7.50 (t, J = 7.8 Hz, 2H), 4.56 (s, 2H).

^{13}C NMR ($CDCl_3$, 150 MHz): δ 193.2, 134.4, 134.1, 129.0, 127.9, 54.9.

HRMS m/z (EI) calc. for [$C_8H_7N_3O$]: 161.1640; found: 161.1640.

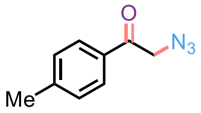

2-Azido-1-(*p*-tolyl)ethan-1-one (2).[10] The title compound was synthesized according to General Procedure B using 1-methyl-4-vinylbenzene (11.8 mg, 0.10 mmol, 1.0 equiv.). The crude mixture was purified by flash column chromatography using hexanes-ethyl acetate (30:1 v/v) as the eluent to give the title compound as a white solid 12.6 mg (0.072 mmol, 72% yield).

^{1}H NMR ($CDCl_3$, 600 MHz): δ 7.80 (d, *J* = 8.4 Hz, 2H), 7.29 (d, *J* = 7.8 Hz, 2H), 4.53 (s, 2H), 2.42 (s, 3H).

^{13}C NMR ($CDCl_3$, 150 MHz): δ 192.8, 145.1, 131.9, 129.6, 128.0, 54.7, 21.7.

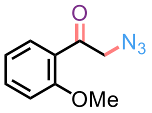

2-Azido-1-(2-methoxyphenyl)ethan-1-one (3). The title compound was synthesized according to General Procedure B using 1-methoxy-2-vinylbenzene (13.4 mg, 0.10 mmol, 1.0 equiv.). The crude mixture was purified by flash column chromatography using hexanes-ethyl acetate (30:1 v/v) as the eluent to give the title compound as a white solid 13.6 mg (0.071 mmol, 71% yield).

^{1}H NMR ($CDCl_3$, 600 MHz): δ 7.92 (dd, *J* = 7.8, 1.8 Hz, 1H), 7.57 – 7.50 (m, 1H), 7.08 – 7.03 (m, 1H), 6.99 (d, *J* = 8.4 Hz, 1H), 4.52 (s, 2H), 3.94 (s, 3H).

^{13}C NMR ($CDCl_3$, 150 MHz): δ 194.4, 159.3, 135.0, 131.1, 124.6, 121.1, 111.6, 59.4, 55.6.

HRMS *m*/*z* (ESI) calc. for $[C_9H_{10}N_3O_2]^+$: 192.0768; found: 192.0763.

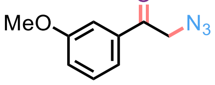

2-Azido-1-(3-methoxyphenyl)ethan-1-one (4). The title compound was synthesized according to General Procedure B using 1-methoxy-3-vinylbenzene (13.4 mg, 0.10 mmol, 1.0 equiv.). The crude mixture was purified by flash column chromatography using hexanes-ethyl acetate (30:1 v/v) as the eluent to give the title compound as a white solid 14.5 mg (0.076 mmol, 76% yield).

^{1}H NMR ($CDCl_3$, 600 MHz): δ 7.48 – 7.42 (m, 2H), 7.40 (t, J = 7.8 Hz, 1H), 7.16 (dd, J = 7.8, 2.4 Hz, 1H), 4.54 (s, 2H), 3.86 (s, 3H).

^{13}C NMR ($CDCl_3$, 150 MHz): δ 193.0, 160.0, 135.6, 129.9, 120.6, 120.3, 112.2, 55.5, 54.9.

HRMS m/z (ESI) calc. for $[C_9H_{10}N_3O_2]^+$: 192.0768; found: 192.0765.

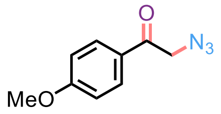

2-Azido-1-(4-methoxyphenyl)ethan-1-one (5).[10] The title compound was synthesized according to General Procedure B using 1-methoxy-4-vinylbenzene (13.4 mg, 0.10 mmol, 1.0 equiv.). The crude mixture was purified by flash column chromatography using hexanes-ethyl acetate (30:1 v/v) as the eluent to give the title compound as a white solid 14.1 mg (0.074 mmol, 74% yield).

^{1}H NMR ($CDCl_3$, 600 MHz): δ 7.88 (d, J = 9.0 Hz, 2H), 6.96 (d, J = 9.0 Hz, 2H), 4.50 (s, 2H), 3.88 (s, 3H).

^{13}C NMR ($CDCl_3$, 150 MHz): δ 191.6, 164.2, 130.2, 127.3, 114.1, 55.5, 54.5.

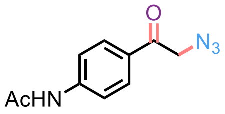

***N*-(4-(2-azidoacetyl)phenyl)acetamide (6).**[11] The title compound was synthesized according to General Procedure B using *N*-(4-vinylphenyl)acetamide (16.1 mg, 0.10 mmol, 1.0 equiv.). The crude mixture was purified by flash column chromatography using hexanes-ethyl acetate (1:1 v/v) as the eluent to give the title compound as a colorless oil 14.4 mg (0.066 mmol, 66% yield).

^{1}H NMR ($CDCl_3$, 600 MHz): δ 7.89 (d, J = 9.0 Hz, 2H), 7.65 (d, J = 8.4 Hz, 2H), 7.33 (br, 1H), 4.52 (s, 2H), 2.23 (s, 3H).

^{13}C NMR ($CDCl_3$, 150 MHz): δ 191.8, 168.4, 143.0, 130.0, 129.5, 119.0, 54.7, 24.9.

HRMS m/z (ESI) calc. for $[C_{10}H_{11}N_4O_2]^+$: 219.0877; found: 219.0873.

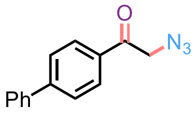

1-([1,1'-Biphenyl]-4-yl)-2-azidoethan-1-one (7). The title compound was synthesized according to General Procedure B using 4-vinyl-1,1'-biphenyl (18.0 mg, 0.10 mmol, 1.0 equiv.). The crude mixture was purified by flash column chromatography using hexanes-ethyl acetate (30:1 v/v) as the eluent to give the title compound as a white solid 17.3 mg (0.073 mmol, 73% yield).

^{1}H NMR ($CDCl_3$, 600 MHz): δ 7.98 (d, J = 8.4 Hz, 2H), 7.72 (d, J = 8.4 Hz, 2H), 7.63 (d, J = 6.6 Hz, 2H), 7.49 (t, J = 7.2 Hz, 2H), 7.42 (t, J = 7.2 Hz, 1H), 4.59 (s, 2H).

^{13}C NMR ($CDCl_3$, 150 MHz): δ 192.8, 146.8, 139.5, 133.0, 129.0, 128.5, 127.6, 127.3, 54.9.

HRMS *m/z* (ESI) calc. for $[C_{14}H_{12}N_3O]^+$: 238.0975; found: 238.0972.

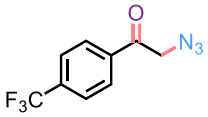

2-Azido-1-(4-(trifluoromethyl)phenyl)ethan-1-one (8).[12] The title compound was synthesized according to General Procedure B using 1-(trifluoromethyl)-4-vinylbenzene (17.2 mg, 0.10 mmol, 1.0 equiv.). The crude mixture was purified by flash column chromatography using hexanes-ethyl acetate (30:1 v/v) as the eluent to give the title compound as a white solid 14.0 mg (0.061 mmol, 61% yield).

^{1}H NMR ($CDCl_3$, 600 MHz): δ 8.02 (d, J = 7.8 Hz, 2H), 7.77 (d, J = 7.8 Hz, 2H), 4.58 (s, 2H).

^{13}C NMR ($CDCl_3$, 150 MHz): δ 192.4, 137.0, 135.4 (q, J = 33.0 Hz), 128.3, 126.1 (q, J = 3.5 Hz), 123.3 (q, J = 272.9 Hz), 55.1.

^{19}F NMR ($CDCl_3$, 565 MHz): δ –63.3.

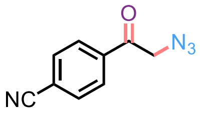

4-(2-Azidoacetyl)benzonitrile (9).[12] The title compound was synthesized according to General Procedure B using 4-vinylbenzonitrile (12.9 mg, 0.10 mmol, 1.0 equiv.). The crude

mixture was purified by flash column chromatography using hexanes-ethyl acetate (10:1 v/v) as the eluent to give the title compound as a white solid 7.6 mg (0.041 mmol, 41% yield).

^{1}H NMR ($CDCl_3$, 600 MHz): δ 8.01 (d, J = 7.8 Hz, 2H), 7.81 (d, J = 8.4 Hz, 2H), 4.57 (s, 2H).

^{13}C NMR ($CDCl_3$, 150 MHz): δ 192.1, 137.2, 132.8, 128.4, 117.5, 117.4, 55.1.

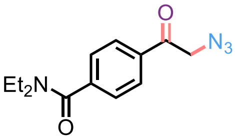

4-(2-Azidoacetyl)-*N,N*-diethylbenzamide (10). The title compound was synthesized according to the General Procedure B using *N,N*-diethyl-4-vinylbenzamide (20.3 mg, 0.10 mmol, 1.0 equiv.). The crude mixture was purified by flash column chromatography using hexanes-ethyl acetate-DCM (2:1:1 v/v) as the eluent to give the title compound as a colorless oil 15.6 mg (0.060 mmol, 60% yield).

^{1}H NMR ($CDCl_3$, 600 MHz): δ 7.94 (d, J = 7.8 Hz, 2H), 7.49 (d, J = 7.8 Hz, 2H), 4.56 (s, 2H), 3.55 (q, J = 7.2, 14.4 Hz, 2H), 3.21 (q, J = 7.2, 14.4 Hz, 2H), 1.26 (t, J = 7.8 Hz, 3H), 1.11 (t, J = 7.2 Hz, 3H).

^{13}C NMR ($CDCl_3$, 150 MHz): δ 192.6, 169.7, 142.7, 134.6, 128.2, 126.9, 54.9, 43.2, 39.4, 14.2, 12.9.

HRMS *m/z* (ESI) calc. for $[C_{13}H_{17}N_4O_2]^+$: 261.1347; found: 261.1341.

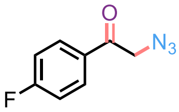

2-Azido-1-(4-fluorophenyl)ethan-1-one (11).[12] The title compound was synthesized according to General Procedure B using 1-fluoro-4-vinylbenzene (12.2 mg, 0.10 mmol, 1.0 equiv.). The crude mixture was purified by flash column chromatography using hexanes-ethyl acetate (30:1 v/v) as the eluent to give the title compound as a white solid 12.5 mg (0.070 mmol, 70% yield).

^{1}H NMR ($CDCl_3$, 600 MHz): δ 7.96 – 7.91 (m, 2H), 7.17 (t, J = 8.4 Hz, 2H), 4.53 (s, 2H).

^{13}C NMR (CDCl$_3$, 150 MHz): δ 191.7, 166.2 (d, J = 255.2 Hz), 130.8 (d, J = 3.0 Hz), 130.7 (d, J = 9.6 Hz), 116.2 (d, J = 22.1 Hz), 54.7.

^{19}F NMR (CDCl$_3$, 565 MHz): δ –102.9.

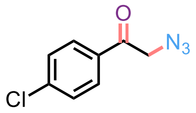

2-Azido-1-(4-chlorophenyl)ethan-1-one (12).[12] The title compound was synthesized according to General Procedure B using 1-chloro-4-vinylbenzene (13.9 mg, 0.10 mmol, 1.0 equiv.). The crude mixture was purified by flash column chromatography using hexanes-ethyl acetate (30:1 v/v) as the eluent to give the title compound as a white solid 13.5 mg (0.069 mmol, 69% yield).

^{1}H NMR (CDCl$_3$, 600 MHz): δ 7.85 (d, J = 9.0 Hz, 4H), 7.48 (d, J = 8.4 Hz, 4H), 4.53 (s, 5H).

^{13}C NMR (CDCl$_3$, 150 MHz): δ 192.1, 140.7, 132.6, 129.3, 129.3, 54.8.

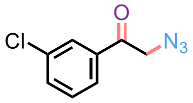

2-Azido-1-(3-chlorophenyl)ethan-1-one (13). The title compound was synthesized according to General Procedure B using 1-chloro-3-vinylbenzene (13.9 mg, 0.10 mmol, 1.0 equiv.). The crude mixture was purified by flash column chromatography using hexanes-ethyl acetate (20:1 v/v) as the eluent to give the title compound as a white solid 12.7 mg (0.065 mmol, 65% yield).

^{1}H NMR (CDCl$_3$, 600 MHz): δ 7.89 (t, J = 1.8 Hz, 1H), 7.78 (dt, J = 7.8, 1.8 Hz, 1H), 7.60 (ddd, J = 7.8, 2.4, 1.2 Hz, 1H), 7.45 (t, J = 8.4 Hz, 1H), 4.54 (s, 2H).

^{13}C NMR (CDCl$_3$, 150 MHz): δ 192.1, 135.8, 135.4, 134.1, 130.3, 128.1, 126.0, 54.9.

HRMS m/z (ESI) calc. for $[C_8H_7ClN_3O]^+$: 196.0273; found: 196.0272.

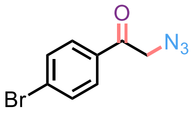

2-Azido-1-(4-bromophenyl)ethan-1-one (14). The title compound was synthesized according to General Procedure B using 1-bromo-4-vinylbenzene (18.3 mg, 0.10 mmol, 1.0 equiv.). The crude mixture was purified by flash column chromatography using hexanes-ethyl acetate (30:1 v/v) as the eluent to give the title compound as a white solid 17.0 mg (0.071 mmol, 71% yield).

^{1}H NMR ($CDCl_3$, 600 MHz): δ 7.77 (d, *J* = 9.0 Hz, 4H), 7.64 (d, *J* = 8.4 Hz, 4H), 4.52 (s, 5H).

^{13}C NMR ($CDCl_3$, 150 MHz): δ 192.3, 133.0, 132.3, 129.4, 129.4, 54.8.

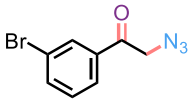

2-Azido-1-(3-bromophenyl)ethan-1-one (15).[13] The title compound was synthesized according to General Procedure B using 1-bromo-3-vinylbenzene (18.3 mg, 0.10 mmol, 1.0 equiv.). The crude mixture was purified by flash column chromatography using hexanes-ethyl acetate (20:1 v/v) as the eluent to give the title compound as a white solid 16.8 mg (0.070 mmol, 70% yield).

^{1}H NMR ($CDCl_3$, 600 MHz): δ 8.03 (t, *J* = 1.8 Hz, 1H), 7.84 – 7.80 (m, 1H), 7.75 (ddd, *J* = 7.8, 1.8, 1.2 Hz, 1H), 7.38 (t, *J* = 7.8 Hz, 1H), 4.53 (s, 2H).

^{13}C NMR ($CDCl_3$, 150 MHz): δ 192.0, 137.0, 136.0, 131.0, 130.5, 126.4, 123.3, 54.9.

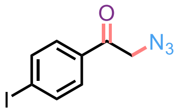

2-Azido-1-(4-iodophenyl)ethan-1-one (16). The title compound was synthesized according to General Procedure B using 1-iodo-4-vinylbenzene (23.0 mg, 0.10 mmol, 1.0 equiv.). The crude mixture was purified by flash column chromatography using hexanes-ethyl acetate (30:1 v/v) as the eluent to give the title compound as a white solid 20.7 mg (0.072 mmol, 72% yield).

^{1}**H NMR** ($CDCl_3$, 600 MHz): δ 7.87 (d, J = 8.4 Hz, 1H), 7.61 (d, J = 8.4 Hz, 1H), 4.51 (s, 2H).

^{13}**C NMR** ($CDCl_3$, 150 MHz): δ 192.6, 138.3, 133.6, 129.2, 102.3, 54.7.

HRMS *m*/*z* (ESI) calc. for $[C_8H_7IN_3O]^+$: 287.9629; found: 287.9622.

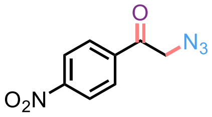

2-Azido-1-(4-nitrophenyl)ethan-1-one (17).[11] The title compound was synthesized according to General Procedure B using 1-nitro-4-vinylbenzene (14.9 mg, 0.10 mmol, 1.0 equiv.). The crude mixture was purified by flash column chromatography using hexanes-ethyl acetate (10:1 v/v) as the eluent to give the title compound as a light yellow solid 8.2 mg (0.040 mmol, 40% yield).

^{1}**H NMR** ($CDCl_3$, 600 MHz): δ 8.36 (d, J = 9.0 Hz, 2H), 8.09 (d, J = 8.4 Hz, 2H), 4.60 (s, 2H).

^{13}**C NMR** ($CDCl_3$, 150 MHz): δ 192.0, 150.9, 138.7, 129.1, 124.2, 55.2.

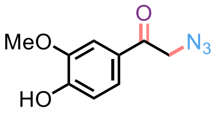

2-Azido-1-(4-hydroxy-3-methoxyphenyl)ethan-1-one (18). The title compound was synthesized according to General Procedure B using 2-methoxy-4-vinylphenol (15.0 mg, 0.10 mmol, 1.0 equiv.). The crude mixture was purified by flash column chromatography using hexanes-ethyl acetate (2:1 v/v) as the eluent to give the title compound as a white solid 11.2 mg (0.054 mmol, 54% yield).

^{1}**H NMR** ($CDCl_3$, 600 MHz): δ 7.53 (d, J = 1.8 Hz, 1H), 7.41 (dd, J = 8.4, 1.8 Hz, 1H), 6.95 (d, J = 8.4 Hz, 1H), 6.21 (s, 1H), 4.51 (s, 2H), 3.96 (s, 3H).

^{13}**C NMR** ($CDCl_3$, 150 MHz): δ 191.7, 151.2, 147.0, 127.3, 123.1, 114.1, 109.8, 56.1, 54.4.

HRMS *m*/*z* (ESI) calc. for $[C_9H_{10}N_3O_3]^+$: 208.0717; found: 208.0717.

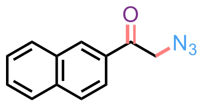

2-Azido-1-(naphthalen-2-yl)ethan-1-one (19). The title compound was synthesized according to General Procedure B using 2-vinylnaphthalene (15.4 mg, 0.10 mmol, 1.0 equiv.). The crude mixture was purified by flash column chromatography using hexanes-ethyl acetate (20:1 v/v) as the eluent to give the title compound as a white solid 15.0 mg (0.071 mmol, 71% yield).

^{1}H NMR ($CDCl_3$, 600 MHz): δ 8.40 (s, 1H), 8.08 – 7.87 (m, 4H), 7.67 – 7.55 (m, 2H), 4.69 (s, 2H).

^{13}C NMR ($CDCl_3$, 150 MHz): δ 193.1, 135.9, 132.3, 131.7, 129.8, 129.6, 129.1, 129.0, 127.9, 127.2, 123.3, 54.9.

HRMS *m/z* (ESI) calc. for $[C_{12}H_{10}N_3O]^+$: 212.0819; found: 212.0818.

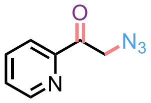

2-Azido-1-(pyridin-2-yl)ethan-1-one (20). The title compound was synthesized according to General Procedure B using 2-vinylpyridine (10.5 mg, 0.10 mmol, 1.0 equiv.). The crude mixture was purified by flash column chromatography using hexanes-ethyl acetate (5:1 v/v) as the eluent to give the title compound as a colorless oil 8.4 mg (0.052 mmol, 52% yield).

^{1}H NMR ($CDCl_3$, 600 MHz): δ 8.69 – 8.62 (m, 1H), 8.11 – 8.05 (m, 1H), 7.88 (td, *J* = 7.8, 1.8 Hz, 1H), 7.53 (ddd, *J* = 7.8, 4.8, 1.2 Hz, 1H), 4.87 (s, 2H).

^{13}C NMR ($CDCl_3$, 150 MHz): δ 194.8, 151.5, 149.2, 137.2, 128.0, 122.1, 55.2.

HRMS *m/z* (ESI) calc. for $[C_7H_7N_4O]^+$: 163.0615; found: 163.0614.

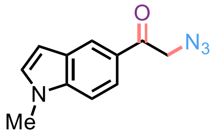

2-Azido-1-(1-methyl-1*H*-indol-5-yl)ethan-1-one (21). The title compound was synthesized according to General Procedure B using 1-methyl-5-vinyl-1*H*-indole (15.7 mg, 0.10 mmol, 1.0 equiv.). The crude mixture was purified by flash column chromatography using

hexanes-ethyl acetate (10:1 v/v) as the eluent to give the title compound as a colorless oil 9.9 mg (0.046 mmol, 46% yield).

^{1}H NMR ($CDCl_3$, 600 MHz): δ 8.22 (d, J = 1.2 Hz, 1H), 7.84 (dd, J = 9.0, 1.8 Hz, 1H), 7.37 (d, J = 8.4 Hz, 1H), 7.15 (d, J = 3.0 Hz, 1H), 6.62 (d, J = 3.0 Hz, 1H), 4.63 (s, 2H), 3.84 (s, 3H).

^{13}C NMR ($CDCl_3$, 150 MHz): δ 192.9, 139.5, 130.8, 128.0, 126.4, 122.6, 121.4, 109.6, 103.2, 54.7, 33.1.

HRMS *m*/*z* (ESI) calc. for [$C_{11}H_{11}N_4O$]: 215.0928; found: 215.0922.

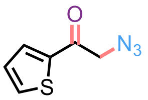

2-Azido-1-(thiophen-2-yl)ethan-1-one (22). The title compound was synthesized according to General Procedure B using 2-vinylthiophene (11.0 mg, 0.10 mmol, 1.0 equiv.). The crude mixture was purified by flash column chromatography using hexanes-ethyl acetate (5:1 v/v) as the eluent to give the title compound as a colorless oil 10.2 mg (0.061 mmol, 61% yield).

^{1}H NMR ($CDCl_3$, 600 MHz): δ 7.75 – 7.69 (m, 2H), 7.19 – 7.15 (m, 1H), 4.45 (s, 2H).

^{13}C NMR ($CDCl_3$, 150 MHz): δ 186.2, 140.7, 134.9, 132.4, 128.4, 54.8.

HRMS *m*/*z* (ESI) calc. for [$C_6H_6N_3OS]^+$: 168.0227; found: 168.0227.

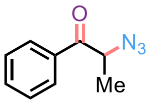

2-Azido-1-phenylpropan-1-one (23).[11] The title compound was synthesized according to General Procedure B using (*E*)-prop-1-en-1-ylbenzene (11.8 mg, 0.10 mmol, 1.0 equiv.). The crude mixture was purified by flash column chromatography using hexanes-ethyl acetate (30:1 v/v) as the eluent to give the title compound as a white solid 7.9 mg (0.045 mmol, 45% yield).

^{1}H NMR ($CDCl_3$, 600 MHz): δ 7.95 (d, J = 7.2 Hz, 2H), 7.62 (t, J = 7.2 Hz, 1H), 7.50 (t, J = 7.8 Hz, 2H), 4.71 (q, J = 7.2 Hz, 1H), 1.58 (d, J = 7.2 Hz, 3H).

^{13}C NMR ($CDCl_3$, 150 MHz): δ 196.7, 134.3, 133.9, 128.9, 128.6, 58.4, 16.4.

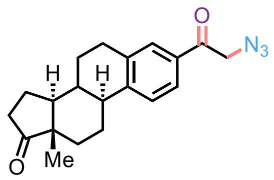

(9*S*,13*S*,14*S*)-3-(2-Azidoacetyl)-13-methyl-6,7,8,9,11,12,13,14,15,16-decahydro-17*H*-cyclopenta[*a*]phenanthren-17-one (24). The title compound was synthesized according to General Procedure B using (9*S*,13*S*,14*S*)-13-methyl-3-vinyl-6,7,8,9,11,12,13,14,15,16-decahydro-17*H*-cyclopenta[*a*]phenanthren-17-one (28 mg, 0.10 mmol, 1.0 equiv.). The crude mixture was purified by flash column chromatography using hexanes-ethyl acetate (4:1 v/v) as the eluent to give the title compound as a white solid 20.2 mg (0.060 mmol, 60% yield).

^{1}H NMR ($CDCl_3$, 600 MHz): δ 7.70 – 7.62 (m, 2H), 7.40 (d, *J* = 8.4 Hz, 1H), 4.53 (s, 2H), 3.04 – 2.90 (m, 2H), 2.57 – 2.48 (m, 1H), 2.48 – 2.41(m, 1H), 2.39 – 2.30(m, 1H), 2.16 (dt, *J* = 18.0, 9.0 Hz, 1H), 2.11 – 2.04 (m, 2H), 2.03 – 1.93 (m, 1H), 1.84 – 1.34 (m, 8H), 0.92 (s, 3H).

^{13}C NMR ($CDCl_3$, 150 MHz): δ 220.4, 193.0, 146.7, 137.5, 132.0, 128.6, 125.9, 125.3, 54.8, 50.5, 47.8, 44.7, 37.7, 35.8, 31.5, 29.2, 26.1, 25.5, 21.5, 13.8.

HRMS *m*/*z* (ESI) calc. for $[C_{20}H_{24}N_3O_2]^+$: 338.1864; found: 338.1856.

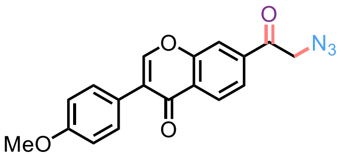

7-(2-Azidoacetyl)-3-(4-methoxyphenyl)-4*H*-chromen-4-one (25). The title compound was synthesized according to General Procedure B using 3-(4-methoxyphenyl)-7-vinyl-4*H*-chromen-4-one (27.8 mg, 0.10 mmol, 1.0 equiv.). The crude mixture was purified by flash column chromatography using hexanes-DCM (1:3 v/v) as the eluent to give the title compound as a white solid 16.8 mg (0.05 mmol, 50% yield).

^{1}H NMR ($CDCl_3$, 600 MHz): δ 8.43 (d, *J* = 8.4 Hz, 1H), 8.08 (s, 1H), 8.05 (s, 1H), 7.88 (d, *J* = 8.4 Hz, 1H), 7.51 (d, *J* = 8.4 Hz, 2H), 6.99 (d, *J* = 9.0 Hz, 2H), 4.64 (s, 2H), 3.85 (s, 3H).

^{13}C NMR ($CDCl_3$, 150 MHz): δ 192.2, 175.6, 159.9, 155.9, 153.2, 138.0, 130.0, 127.8, 127.7, 125.8, 123.5, 123.3, 118.4, 114.1, 55.4, 55.3.

HRMS *m*/*z* (ESI) calc. for $[C_{18}H_{14}N_3O_4]^+$: 336.0979; found: 336.0971.

Table S5. Summary of Existing Homogeneous and Heterogeneous Systems for Oxo-Azidation of Styrene

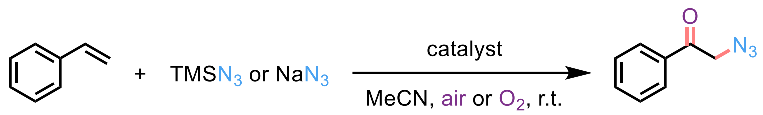

Entry	Homogeneous or Heterogeneous	Catalyst & Additive	N_3 source (x equiv.)	Light	Air or O_2	Time (h)	TON[a]	Ref.
1	Heterogeneous	0.1 mol% **1D-PDI-dpp(phen)Cu**	$TMSN_3$ (4 equiv.)	with	air	12	780	This work
2	Heterogeneous	0.02 mol% cages **3** (0.16 mol% [Cu(Me$_2$**L**)$_2$]PF$_6$)	$TMSN_3$ (2 equiv.)	with	air	36	525	*J. Am. Chem. Soc.* **2024**, *146*, 3147–3159
3	Homogeneous	1 mol% [Cu(dap)$_2$]Cl	$TMSN_3$ (2 equiv.)	with	air	12	79	*Angew. Chem. Int. Ed.* **2018**, *57*, 8288–8292
4	Homogeneous	1 mol% Rose Bengal, 5 mol% PhSeSePh	$TMSN_3$ (2 equiv.)	with	air	2	92	*Green Chem.*, **2018**, *20,* 3197
5	Homogeneous	15 mol% $Cu(OTf)_2$	$TMSN_3$ (4 equiv.)	w/o	O_2	18	5	*J. Org. Chem.* **2018**, *83,* 7852–7859
6	Homogeneous	10 mol% **TetMe-IA**, NBS (1 equiv.), oxone (1 equiv.), $NaHCO_3$ (2.3 equiv.)	NaN_3 (1.5 equiv.)	w/o	air	10	9	*Tetrahedron* **2017**, *73*, 5827e5832

[a]TON values were calculated based on ^{1}H NMR yields rather than isolated yields.

Table S6. TON Comparison in Copper Catalysis[a]

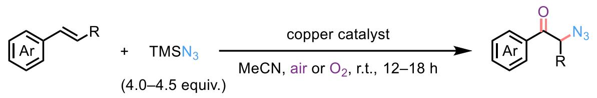

		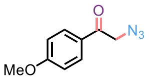	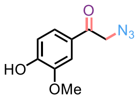	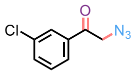	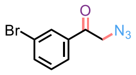	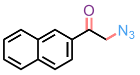	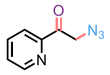
our results **1D-PDI-dpp(phen)Cu** (0.1 mol%)[a] hν (green LED, 525 nm)	Yield:	74%	54%	65%	70%	71%	52%
	TON:	740	540	650	700	710	520
$Cu(OTf)_2$ (15 mol%)[b] reported in *J. Org. Chem.* **2018**, *83*, 7852–7859	Yield:	27%	traces	55%	51%	53%	traces
	TON:	1.8	0	3.7	3.4	3.5	0

[a]Reaction conditions: styrene derivative (0.10 mmol, 1.0 equiv.), $TMSN_3$ (4.0 equiv.), and **1D-PDI-dpp(phen)Cu** (0.1 mol%) in acetonitrile (0.4 mL) under air atmosphere at room temperature with green-LED light irradiation (525 nm) for 12 h. Data are reported as isolated yields. [b]Reaction conditions: styrene derivative (0.50 mmol, 1.0 equiv.), $TMSN_3$ (4.5 equiv.), and catalyst (15 mol%) in acetonitrile (4.0 mL) under O_2 atmosphere at room temperature for 18 h. Data are reported as isolated yields.

6.4 Other Photocatalytic Applications

Chlorosulfonylation of Styrene

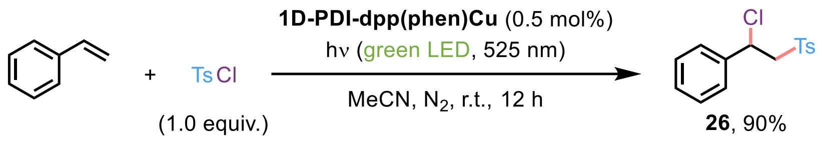

1-((2-Chloro-2-phenylethyl)sulfonyl)-4-methylbenzene (26). To a 10-mL flame-dried glass tube were added **1D-PDI-dpp(phen)Cu** (3.35 mg, 0.5 mol%), styrene (0.10 mmol, 1.0 equiv.), TsCl (19.1 mg, 0.10 mmol, 1.0 equiv.) and MeCN (0.4 mL) under nitrogen atmosphere sequentially. The reaction mixture was irradiated by two 40-watt Kessil PR160L-525 green-LED lamps at room temperature (with one fan) for 12 hours. After irradiation, ethyl acetate (5 mL) was added into the crude mixture; the resulting solution was filtered through a pad of silica gel and washed three times with ethyl acetate (3 × 5 mL), dried over Na_2SO_4 and evaporated under reduced pressure. The residue was purified by flash column chromatography using hexanes-ethyl acetate (5:1 v/v) as the eluent to give the title compound as a white solid 26.5 mg (0.090 mmol, 90% yield).

^{1}H NMR ($CDCl_3$, 600 MHz): δ 7.62 (d, J = 7.8 Hz, 1H), 7.28 – 7.24 (m, 5H), 7.23 (d, J = 1.2 Hz, 1H), 5.32 (t, J = 7.2 Hz, 1H), 3.93 (dd, J = 15.0, 6.6 Hz, 1H), 3.83 (dd, J = 15.0, 6.6 Hz, 1H), 2.40 (s, 2H).

^{13}C NMR ($CDCl_3$, 150 MHz): δ 144.9, 138.6, 136.2, 129.7, 129.0, 128.9, 128.1, 127.1, 64.1, 55.1, 21.6.

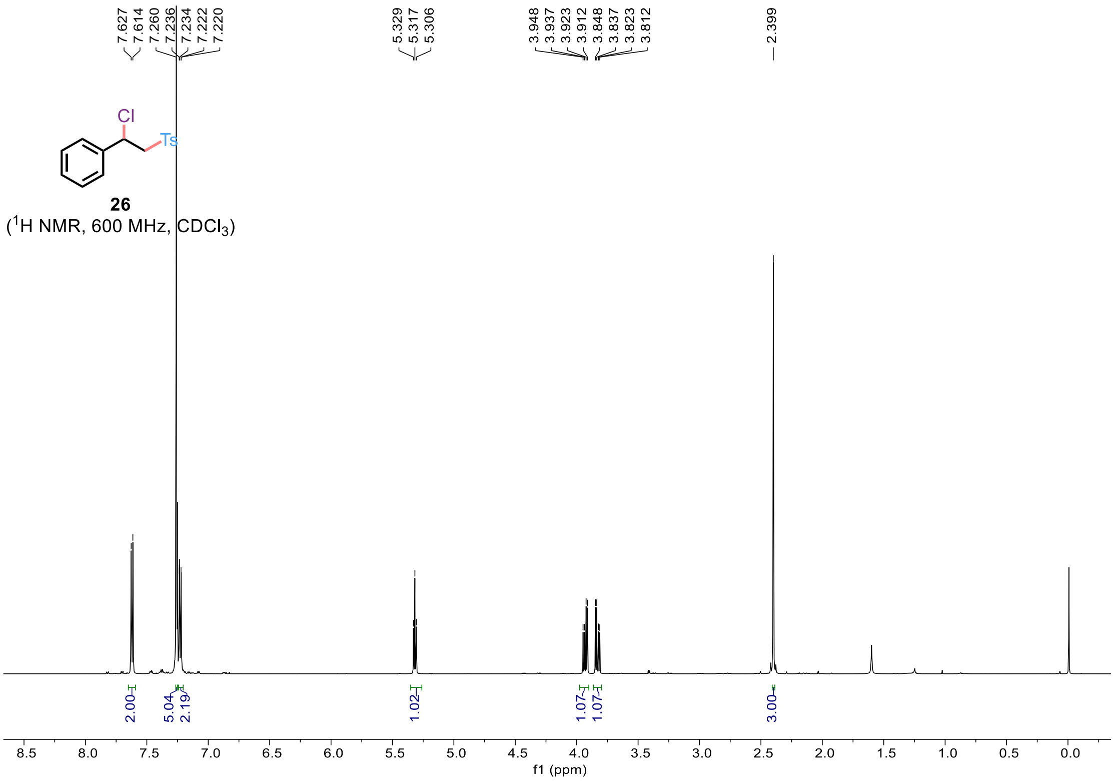

Figure S27. ^{1}H NMR spectrum of **26**.

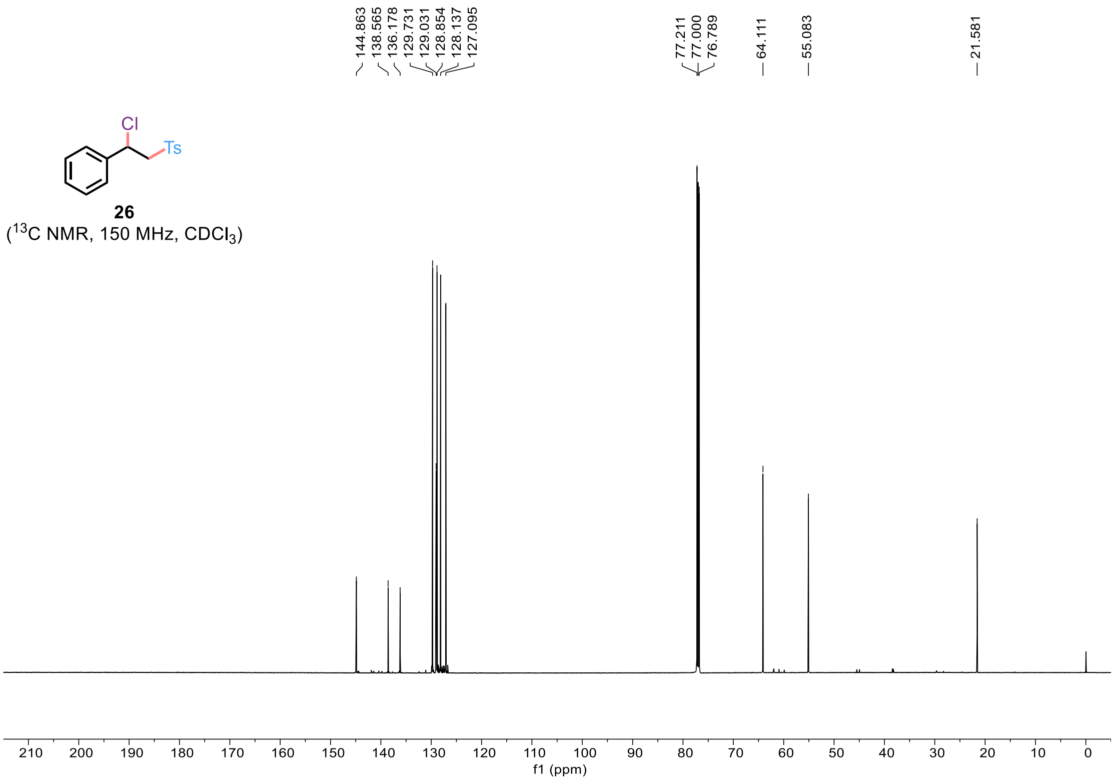

Figure S28. ^{13}C NMR spectrum of **26**.

Atom Transfer Radical Addition of Fluoroalkylsulfonyl Chloride with *N*-Phenylacrylamide

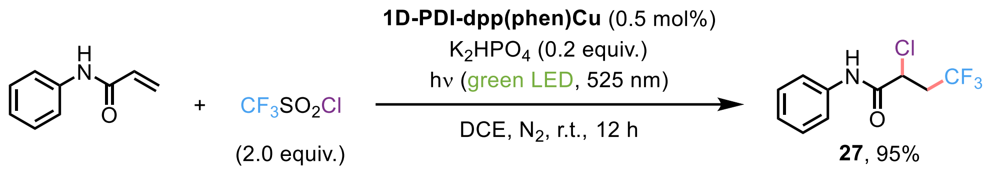

3-Chloro-5,5,5-trifluoro-1-phenylpentan-2-one (27). To a 10-mL flame-dried glass tube were added **1D-PDI-dpp(phen)Cu** (0.3.35 mg, 0.5 mol%), *N*-phenylacrylamide (0.10 mmol, 1.0 equiv.), CF_3SO_2Cl (33.6 mg, 0.20 mmol, 2.0 equiv.), K_2HPO_4 (3.5 mg, 0.02 mmol, 0.2 equiv.) and DCE (0.4 mL) under nitrogen atmosphere sequentially. The reaction mixture was irradiated by two 40-watt Kessil PR160L-525 green-LED lamps at room temperature (with one fan) for 12 hours. After irradiation, ethyl acetate (5 mL) was added into the crude mixture; the resulting solution was filtered through a pad of silica gel and washed three times with ethyl acetate (3 × 5 mL), dried over Na_2SO_4 and evaporated under reduced pressure. The residue was purified by flash column chromatography using hexanes-ethyl acetate (2:1 v/v) as the eluent to give the title compound as a white solid 23.8 mg (0.095 mmol, 95% yield).

^{1}H NMR ($CDCl_3$, 600 MHz): δ 8.35 (br, 1H), 7.52 (d, J = 7.8 Hz, 2H), 7.36 (t, J = 8.4 Hz, 2H), 7.19 (t, J = 7.8 Hz, 1H), 4.65 (dd, J = 9.0, 3.6 Hz, 1H), 3.46 – 3.13 (m, 1H), 2.91 – 2.65 (m, 1H).

^{13}C NMR ($CDCl_3$, 150 MHz): δ 164.4, 136.4, 129.2, 125.6, 125.1 (q, J = 277 Hz), 120.3, 52.4 (q, J = 3.2 Hz), 39.0 (q, J = 29.8 Hz).

^{19}F NMR ($CDCl_3$, 565 MHz): δ –64.0 (t, J = 9.9 Hz).

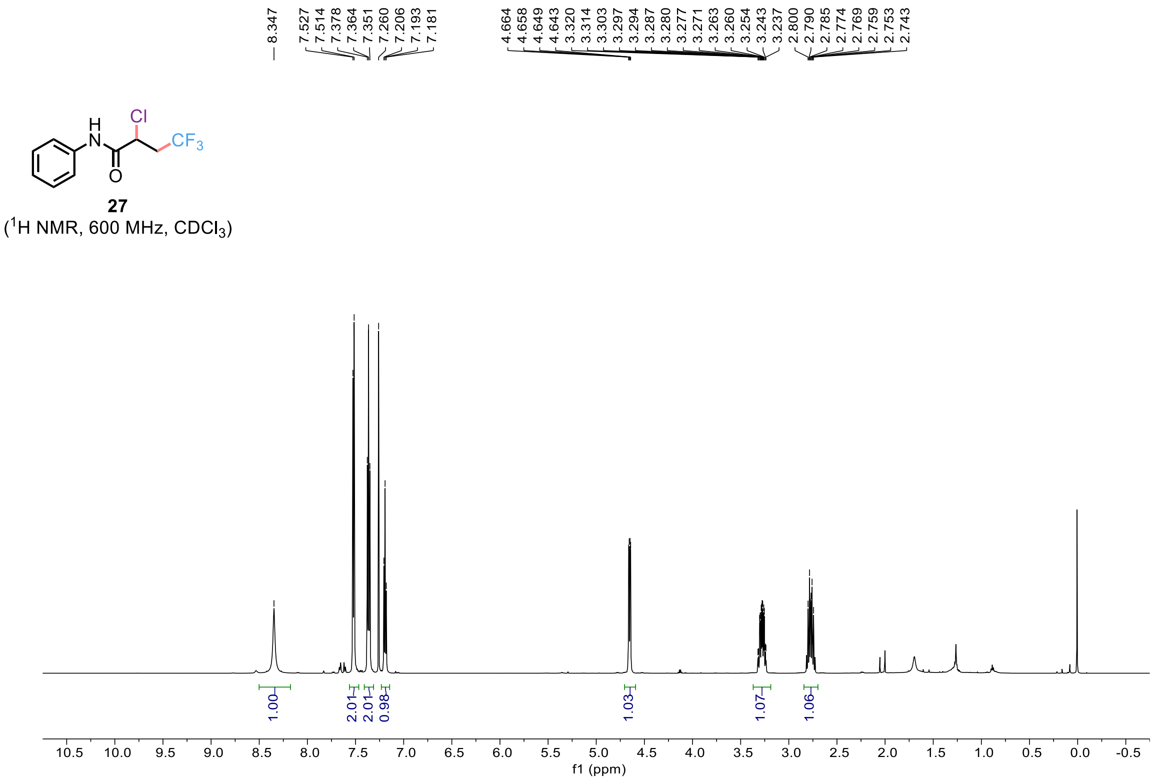

Figure S29. ^{1}H NMR spectrum of **27**.

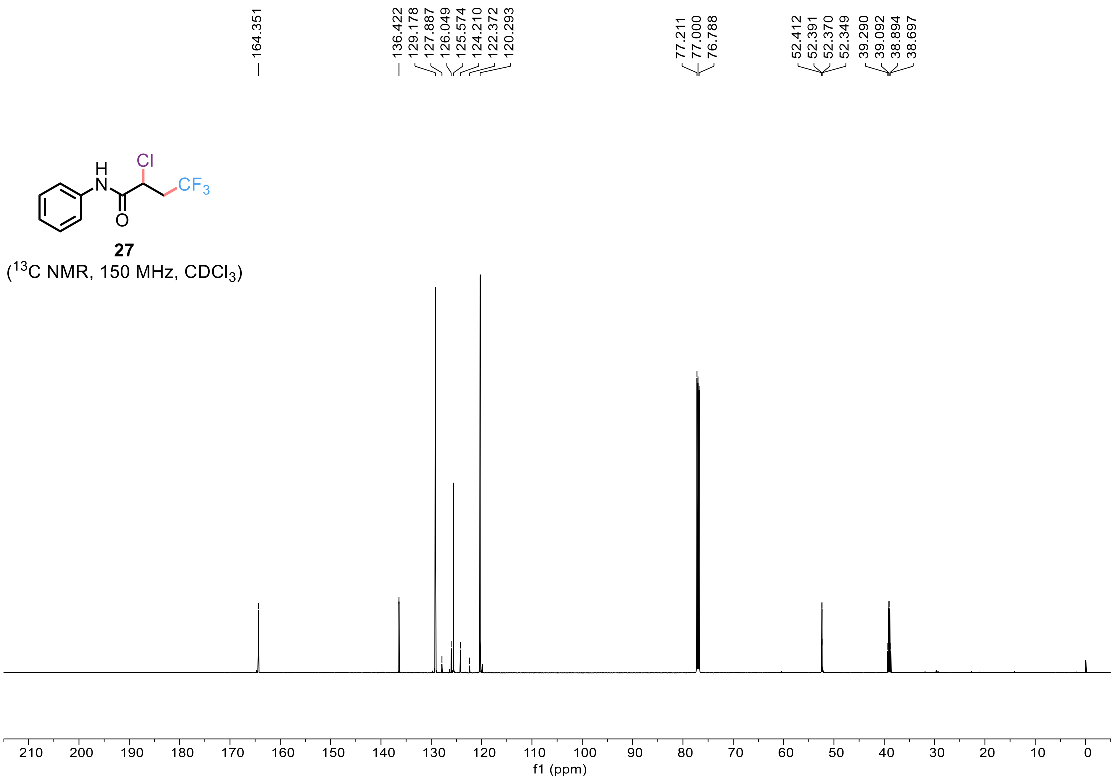

Figure S30. ^{13}C NMR spectrum of **27**.

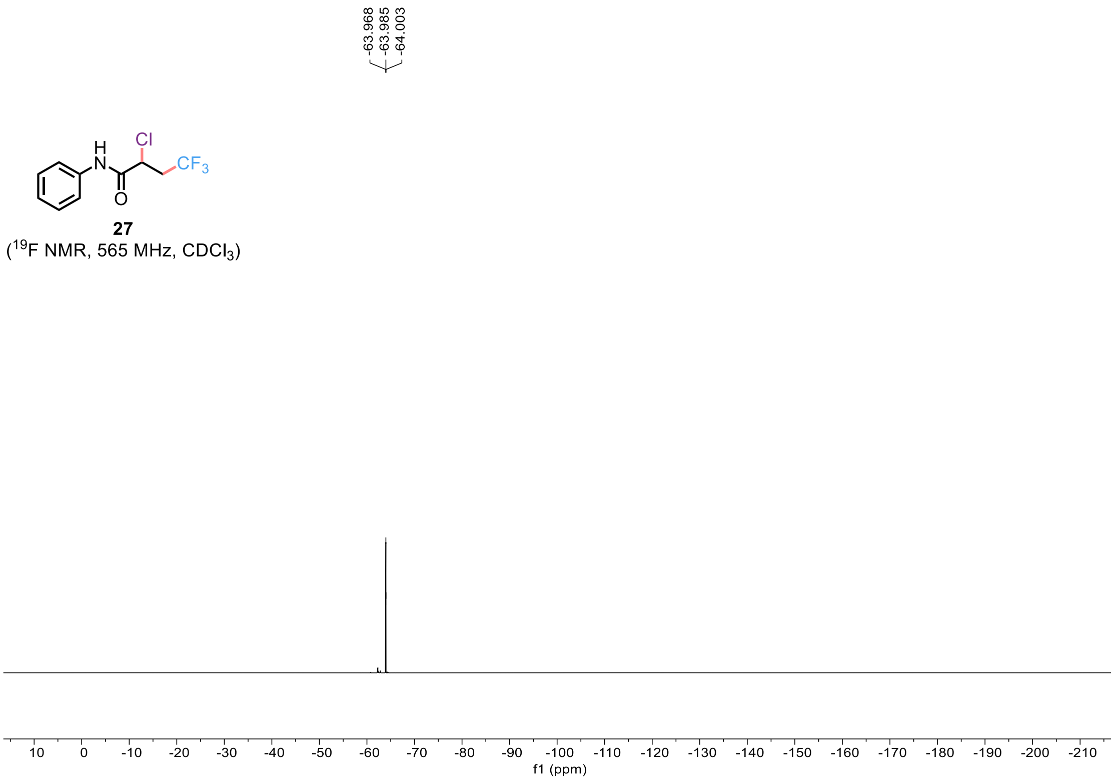

Figure S31. ^{19}F NMR spectrum of **27**.

7. Stability Tests of 1D-PDI-COFs

7.1 Solvent Stability Studies of 1D-PDI-dpp

To a 10-mL centrifuge tube was added **1D-PDI-dpp** powder (5.0 mg) and solvent (3.0 mL). After the treatment with organic solvents or H_2O under stirring (200 rpm) for 24 hours, **1D-PDI-dpp** was recovered by filtration. The resulting powder was then subjected to PXRD testing.

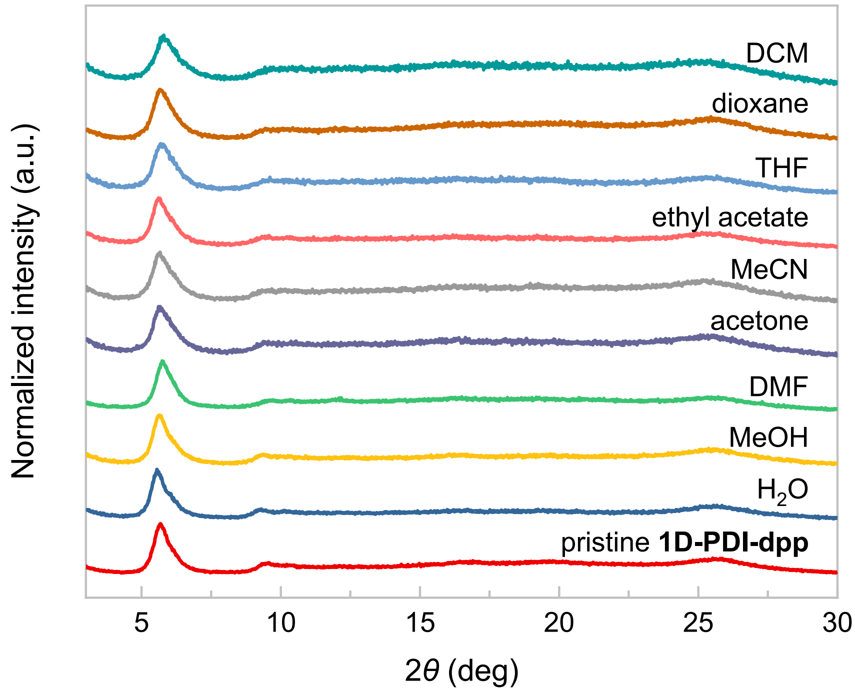

Figure S32. PXRD patterns of the pristine **1D-PDI-dpp** and after the treatment with various solvents.

7.2 Recycling Experiments of 1D-PDI-dpp(phen)Cu

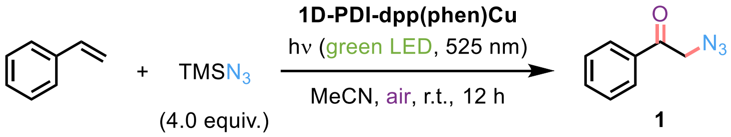

In the recycling experiments for the synthesis of **1**, styrene (0.1 mmol, 1.0 equiv.), $TMSN_3$ (0.4 mmol, 4.0 equiv.), **1D-PDI-dpp(phen)Cu** (0.2 mol%) and MeCN (0.4 mL) were used. The reaction mixture was irradiated by two 40-watt Kessil PR160L-525 green-LED lamps at room temperature (with one fan) for 12 hours. After each cycle, the catalyst was dried and then used directly without further treatment.

7.3 Recycling Experiments of 1D-PDI-dppCu

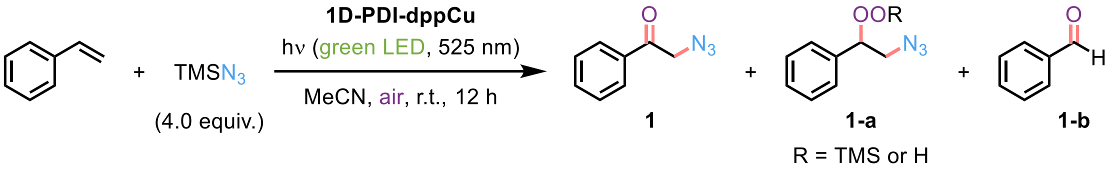

Table S7. Results of Recycling Experiments with 1D-PDI-dppCu[a]

Entry	Recycling	Yield of **1** (%)	Yield of **1-a** (%)	Yield of **1-b** (%)	Conversion of styrene (%)
1	1st run	32	9	31	81
2	2nd run	31	12	28	78

[a]Reaction conditions: styrene (0.1 mmol), $TMSN_3$ (4.0 equiv.), **1D-PDI-dppCu** (0.1 mol%) in MeCN (0.4 mL) under air atmosphere at room temperature for 12 h. Yield was determined by ^1H NMR of the crude product using 1,1,2,2-tetrachloroethane as an internal standard.

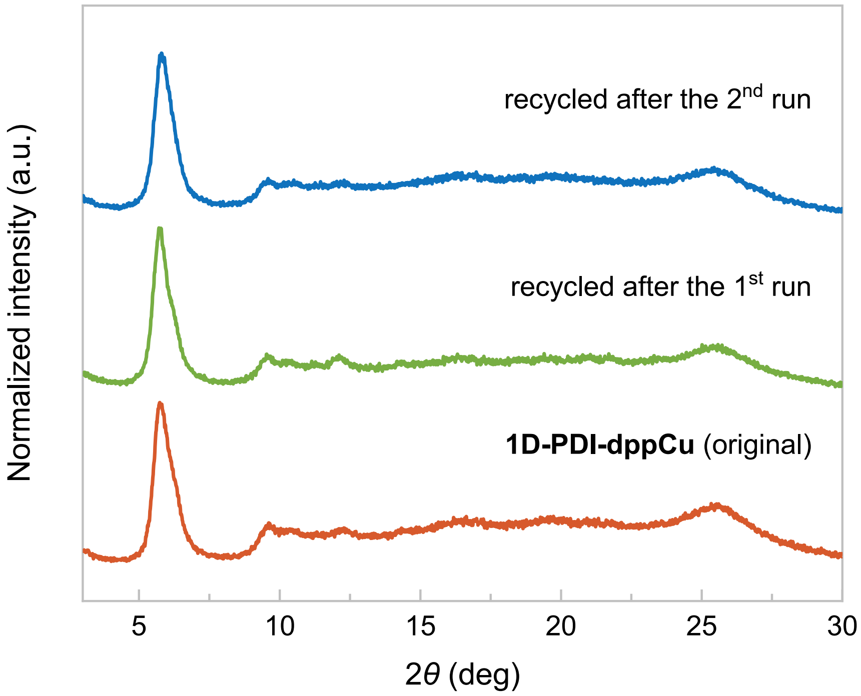

Figure S33. PXRD patterns of the original (red line) and recycled **1D-PDI-dppCu** after first run (green line), and second run (blue line).

7.4 Recycling Experiments of 1D-PDI-phen(phen)Cu

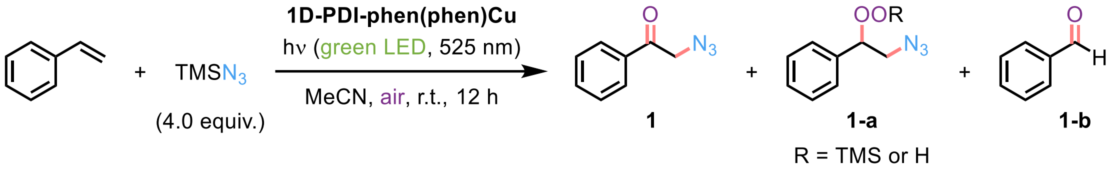

Table S8. Results of Recycling Experiments with 1D-PDI-phen(phen)Cu[a]

Entry	Recycling	Yield of **1** (%)	Yield of **1-a** (%)	Yield of **1-b** (%)	Conversion of styrene (%)
1	1st run	34	7	9	50
2	2nd run	17	5	<5	25

[a]Reaction conditions: styrene (0.1 mmol), $TMSN_3$ (4.0 equiv.), **1D-PDI-phen(phen)Cu** (0.1 mol%) in MeCN (0.4 mL) under air atmosphere at room temperature for 12 h. Yield was determined by ^{1}H NMR of the crude product using 1,1,2,2-tetrachloroethane as an internal standard.

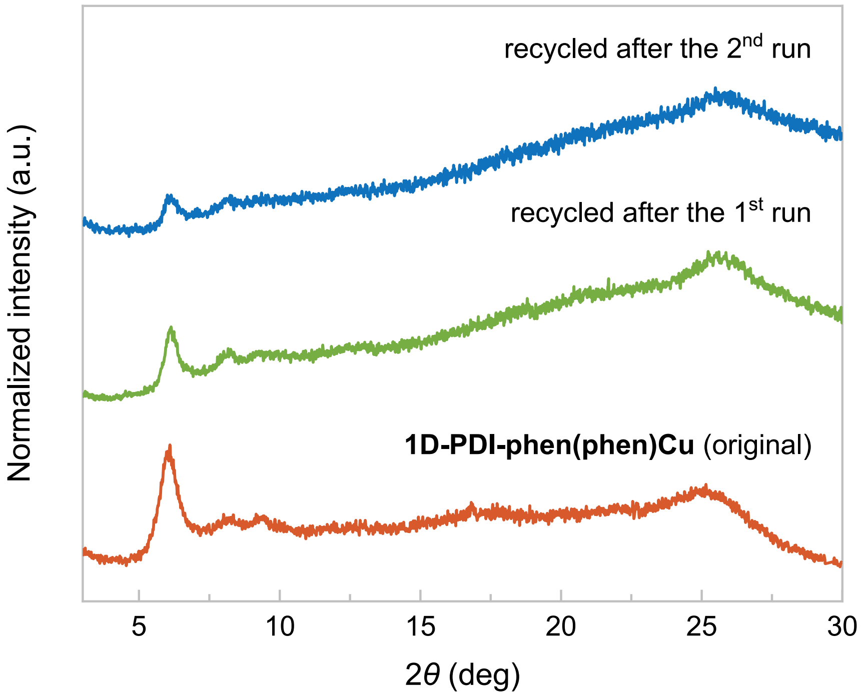

Figure S34. PXRD patterns of the original (red line), recycled **1D-PDI-phen(phen)Cu** after first run (green line), and second run (blue line).

8. Mechanistic Studies

8.1 Electron Paramagnetic Resonance (EPR) Studies

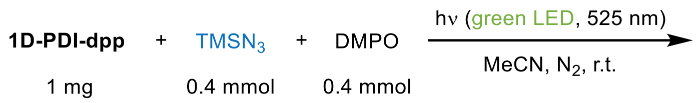

To a 10-mL flame-dried Schlenk tube with a stir bar were added **1D-PDI-dpp** (1 mg), $TMSN_3$ (46.1 mg, 0.40 mmol), DMPO (45.3 mg, 0.4 mmol), and MeCN (0.4 mL) under N_2 atmosphere. Upon vigorous stirring for 5 minutes, the solution sample was transferred into an EPR tube for X-band EPR measurement at room temperature to obtain the black line. Subsequently, the reaction mixture was irradiated with one 40-watt green-LED lamp (525 nm) at room temperature for 0, 0.5, 1.5, and 3 minutes and then analyzed by EPR. The EPR spectra were recorded on a Bruker ESR 5000 spectrometer operating at 9.431 GHz with a sweep time of 60 seconds.

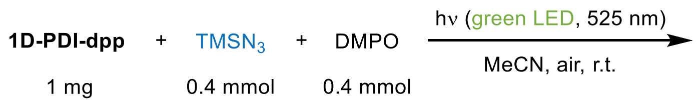

To a 10-mL flame-dried Schlenk tube with a stir bar were added **1D-PDI-dpp** (1 mg), $TMSN_3$ (46.1 mg, 0.40 mmol), DMPO (45.3 mg, 0.4 mmol), and MeCN (0.4 mL) under air atmosphere. Upon vigorous stirring for 5 minutes, the solution sample was transferred into an EPR tube for X-band EPR measurement at room temperature to obtain the black line. Subsequently, the reaction mixture was irradiated with one 40-watt green-LED lamp (525 nm) at room temperature for 0, 5, 10, and 15 minutes and then analyzed by EPR. The EPR spectra were recorded on a Bruker ESR 5000 spectrometer operating at 9.431 GHz with a sweep time of 60 seconds.

8.2 Luminescence Studies

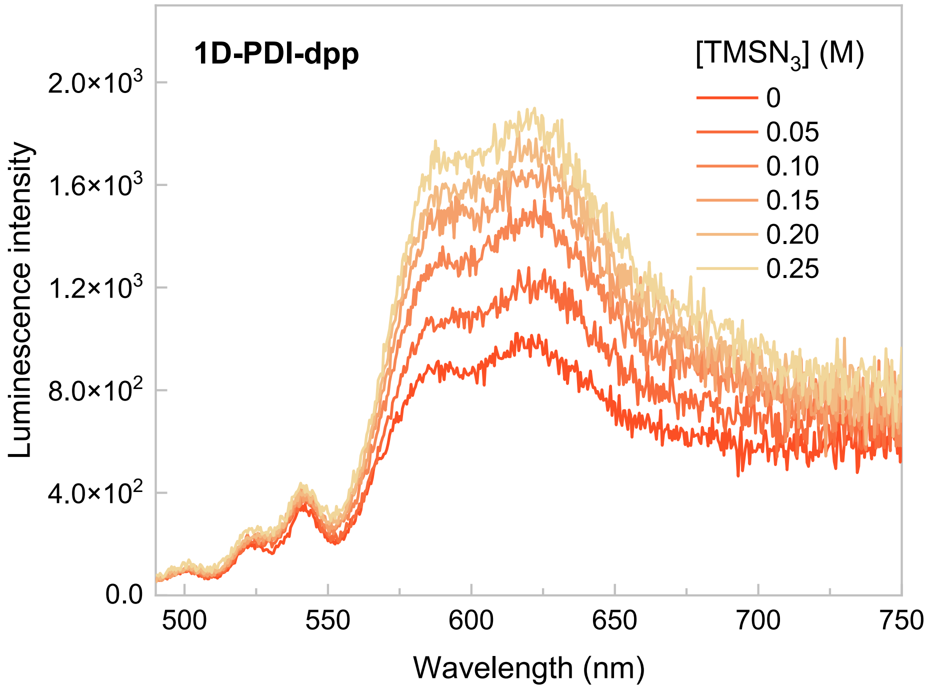

Figure S35. Luminescence emission spectra of **1D-PDI-dpp** as a function of the concentration of $TMSN_3$ upon excitation at 467 nm.

8.3 Rate Profile Measurements

Effect of Peroxide Concentration

To a 10-mL flame-dried glass tube were added **1D-PDI-dpp(phen)Cu** (0.1 mol%), peroxide **1-a** (*z* mmol), $TMSN_3$ (46.1 mg, 0.40 mmol) and MeCN (0.4 mL) sequentially. The reaction mixture was irradiated by two 40-watt Kessil PR160L-525 green-LED lamps at room temperature (with one fan) for certain time. The amounts of **1** were determined by ^{1}H NMR spectroscopy using 1,1,2,2-tetrachloroethane as an internal standard.

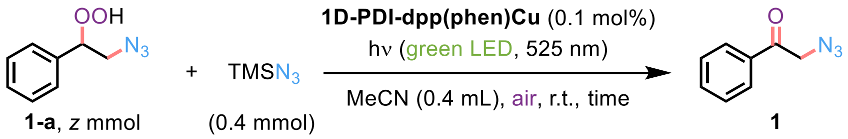

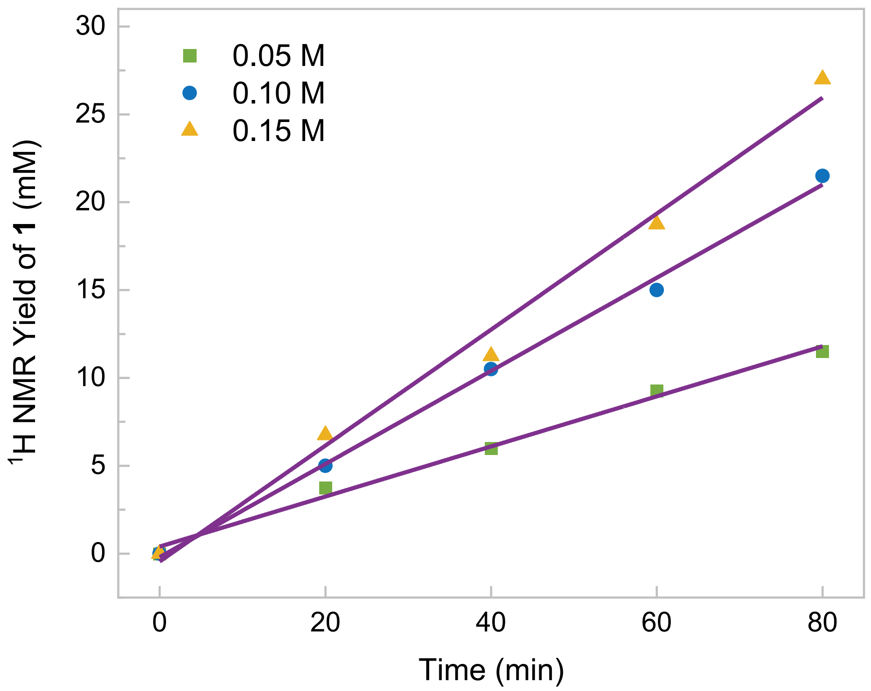

Figure S36. Initial rate studies on different peroxide **1-a** loadings.

peroxide **1-a** 0.05 M: $y = 0.1425x + 0.4$, $R^2 = 0.9927$

peroxide **1-a** 0.10 M: $y = 0.265x – 0.2$, $R^2 = 0.9972$

peroxide **1-a** 0.15 M: $y = 0.33x – 0.45$, $R^2 = 0.9903$

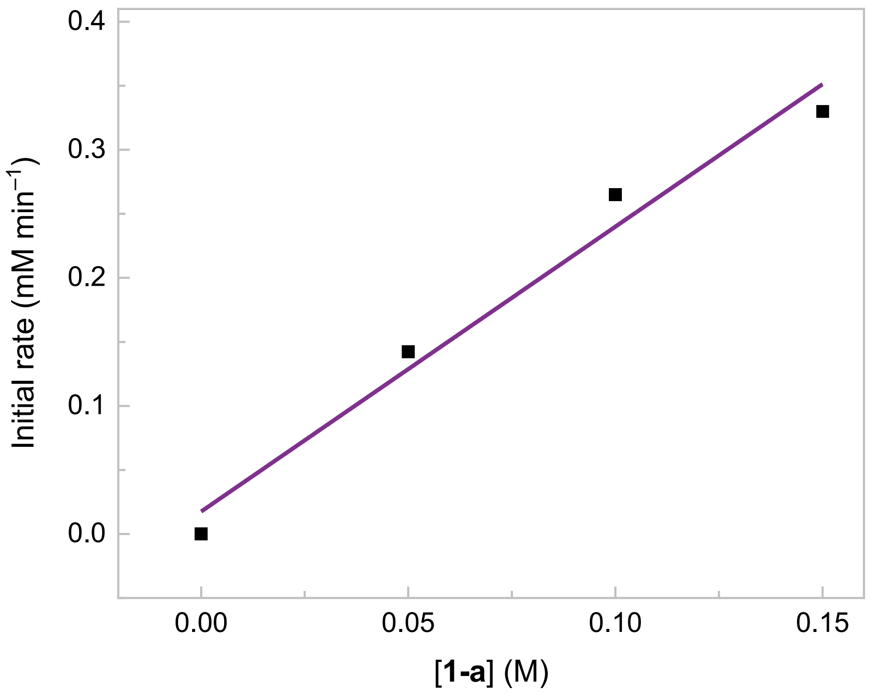

Figure S37. Dependence of the reaction rate on the concentration of peroxide **1-a**.

$y = 2.225x + 0.0175$, $R^2 = 0.9752$

Effect of Catalyst Concentration

To a 10-mL flame-dried glass tube were added **1D-PDI-dpp(phen)Cu** (*z* mol%), peroxide **1-a** (0.04 mmol, 1.0 equiv.), $TMSN_3$ (46.1 mg, 0.40 mmol, 4.0 equiv.) and MeCN (0.4 mL) sequentially. The reaction mixture was irradiated by two 40-watt Kessil PR160L-525 green-LED lamps at room temperature (with one fan) for certain time. The amounts of **1** were determined by ^1H NMR spectroscopy using 1,1,2,2-tetrachloroethane as an internal standard.

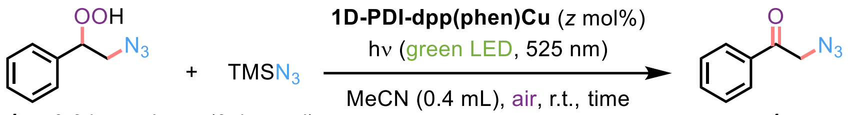

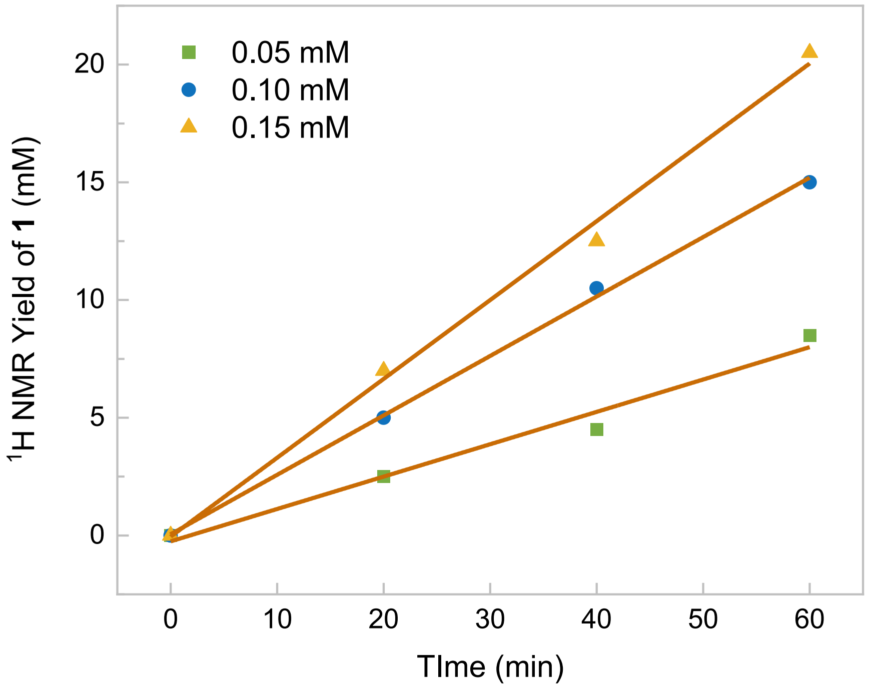

Figure S38. Initial rate studies on different loadings of **1D-PDI-dpp(phen)Cu**.

1D-PDI-dpp(phen)Cu 0.05 mM: $y = 0.1375x - 0.25$, $R^2 = 0.9774$

1D-PDI-dpp(phen)Cu 0.10 mM: $y = 0.2525x + 0.05$, $R^2 = 0.9986$

1D-PDI-dpp(phen)Cu 0.15 mM: $y = 0.3350x - 0.05$, $R^2 = 0.9953$

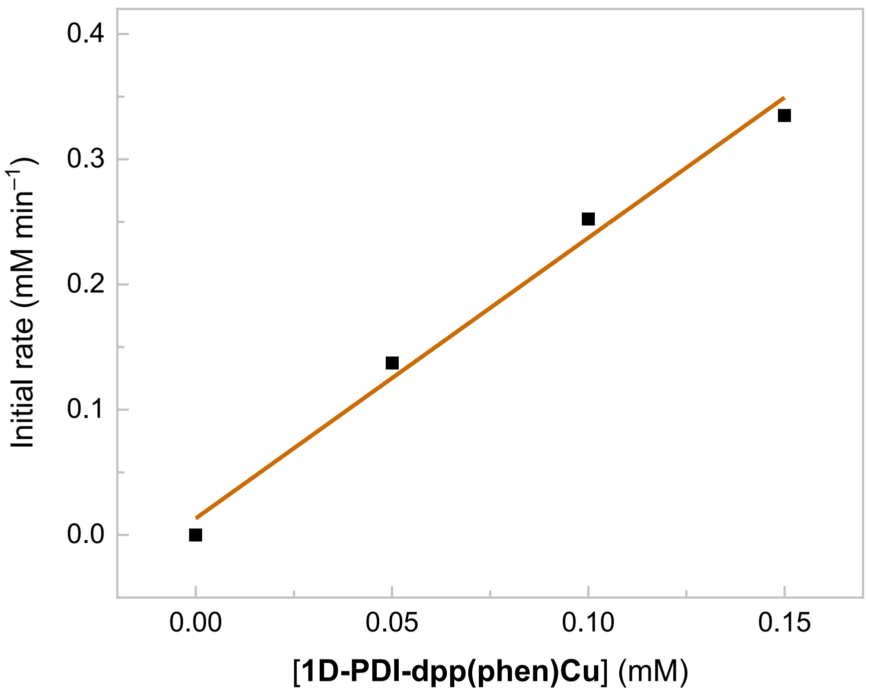

Figure S39. Dependence of the reaction rate on the concentration of **1D-PDI-dpp(phen)Cu**.

$y = 2.24x + 0.013$, $R^2 = 0.9880$

Kinetic Studies on Peroxide Conversion with 1D-PDI-dppCu

To a 10-mL flame-dried glass tube were added **1D-PDI-dppCu** (0.1 mol%), peroxide **1-a** (0.04 mmol, 1.0 equiv.), $TMSN_3$ (46.1 mg, 0.40 mmol, 4.0 equiv.) and MeCN (0.4 mL) sequentially. The reaction mixture was irradiated by two 40-watt Kessil PR160L-525 green-LED lamps at room temperature (with one fan) for *x* hours. The amounts of **1** were determined by ^{1}H NMR spectroscopy using 1,1,2,2-tetrachloroethane as an internal standard.

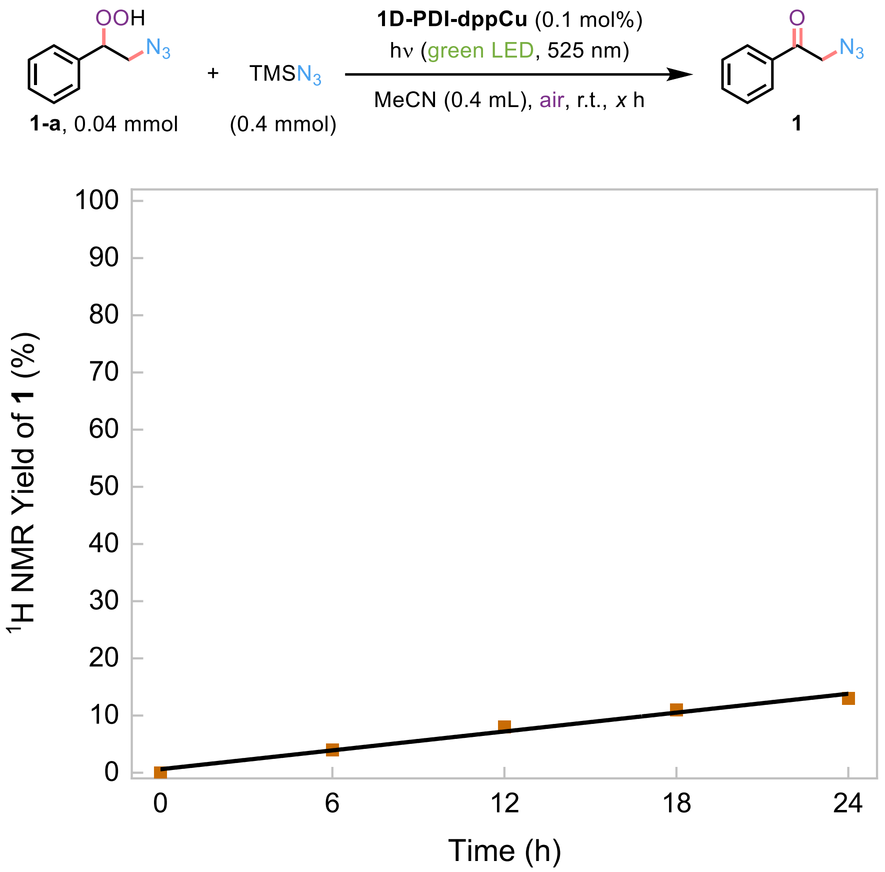

Figure S40. Initial rate studies for α-azido ketone formation with **1D-PDI-dppCu**.

$y = 0.55x + 0.6$, $R^2 = 0.9829$

8.4 Investigation of Copper Coordination Modes in Peroxide Conversion

Table S9. Effect of Ligand and Copper Ratios[a]

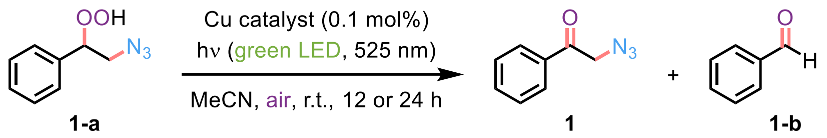

Entry	Ratio of Phen and Cu^{2+}	Yield of **1** (%)	Yield of **1-b** (%)	Peroxide **1-a** conversion (%)
1	1:1	5	5	20
2[b]	1:1	12	8	36
3	2:1	13	4	34
4[b]	2:1	31	11	56
5	3:1	6	3	25
6[b]	3:1	13	5	35

[a]Standard conditions: peroxide **1-a** (0.10 mmol, 1.0 equiv.), Cu catalyst (0.1 mol%) in MeCN (0.4 mL) under air atmosphere at room temperature for 12 h. The catalyst was prepared in situ by adding $AgPF_6$ to an acetonitrile solution of $CuCl_2$. Yield was determined by ^1H NMR using 1,1,2,2-tetrachloroethane as an internal standard. [b]Reaction time was extended to 24 h.

9. References

(1) Linke, M.; Chambron, J.-C.; Heitz, V.; Sauvage, J.-P.; Encinas, S.; Barigelletti, F.; Flamigni, L. Multiporphyrinic Rotaxanes: Control of Intramolecular Electron Transfer rate by Steering the Mutual Arrangement of the Chromophores. *J. Am. Chem. Soc.* **2000**, *122*, 11834–11844.

(2) Lewis, F. W.; Harwood, L. M.; Hudson, M. J.; Drew, M. G.; Desreux, J. F.; Vidick, G.; Bouslimani, N.; Modolo, G.; Wilden, A.; Sypula, M. Highly Efficient Separation of Actinides from Lanthanides by a Phenanthroline-Derived Bis-triazine Ligand. *J. Am. Chem. Soc.* **2011**, *133*, 13093–13102.

(3) Heckelmann, I.; Lu, Z.; Prentice, J. C.; Auras, F.; Ronson, T. K.; Friend, R. H.; Nitschke, J. R.; Feldmann, S. Supramolecular Self-Assembly as a Tool To Preserve the Electronic Purity of Perylene Diimide Chromophores. *Angew. Chem., Int. Ed.* **2023**, *62*, e202216729.

(4) Gordo-Lozano, M.; Martínez-Fernández, M.; Paitandi, R. P.; Martínez, J. I.; Segura, J. L.; Seki, S. Boosting Photoconductivity by Increasing the Structural Complexity of Multivariate Covalent Organic Frameworks. *Small* **2025**, *21*, 2406211.

(5) Ruthkosky, M.; Castellano, F. N.; Meyer, G. J. Photodriven Electron and Energy Transfer from Copper Phenanthroline Excited States. *Inorg. Chem.* **1996**, *35*, 6406–6412.

(6) Yuan, P. F.; Yang, Z.; Zhang, S. S.; Zhu, C. M.; Yang, X. L.; Meng, Q. Y. Deconstructive Carboxylation of Activated Alkenes with Carbon Dioxide. *Angew. Chem., Int. Ed.* **2024**, *136*, e202313030.

(7) Music, A.; Baumann, A. N.; Spieß, P.; Hilgert, N.; Köllen, M.; Didier, D. Single-pot Access to Bisorganoborinates: Applications in Zweifel Olefination. *Org. Lett.* **2019**, *21*, 2189–2193.

(8) Cao, J.; Lv, D.; Yu, F.; Chiou, M.-F.; Li, Y.; Bao, H. Regioselective Three-Component Synthesis of Vicinal Diamines via 1, 2-Diamination of Styrenes. *Org. Lett.* **2021**, *23*, 3184–3189.

(9) Wang, G.; Xie, X.; Xu, W.; Liu, Y. Nickel-Catalyzed Highly Regioselective Hydrocyanation of Alkenes with $Zn(CN)_2$. *Org. Chem. Front.* **2019**, *6*, 2037–2042.

(10) Wei, W.; Cui, H.; Yue, H.; Yang, D. Visible-Light-Enabled Oxyazidation of Alkenes Leading to α-Azidoketones in Air. *Green Chem.* **2018**, *20*, 3197–3202.

(11) Hossain, A.; Vidyasagar, A.; Eichinger, C.; Lankes, C.; Phan, J.; Rehbein, J.; Reiser, O. Visible-Light-Accelerated Copper (II)-Catalyzed Regio-and Chemoselective Oxo-Azidation of Vinyl Arenes. *Angew. Chem., Int. Ed.* **2018**, *57*, 8288–8292.

(12) Ye, Z.; Zhu, R.; Wang, F.; Jiang, H.; Zhang, F. Electrochemical Difunctionalization of Styrenes via Chemoselective Oxo-Azidation or Oxo-Hydroxyphthalimidation. *Org. Lett.* **2021**, *23*, 8240–8245.

(13) Hussain, M. I.; Feng, Y.; Hu, L.; Deng, Q.; Zhang, X.; Xiong, Y. Copper-Catalyzed Oxidative Difunctionalization of Terminal Unactivated Alkenes. *J. Org. Chem.* **2018**, *83*, 7852–7859.

10. NMR Spectra

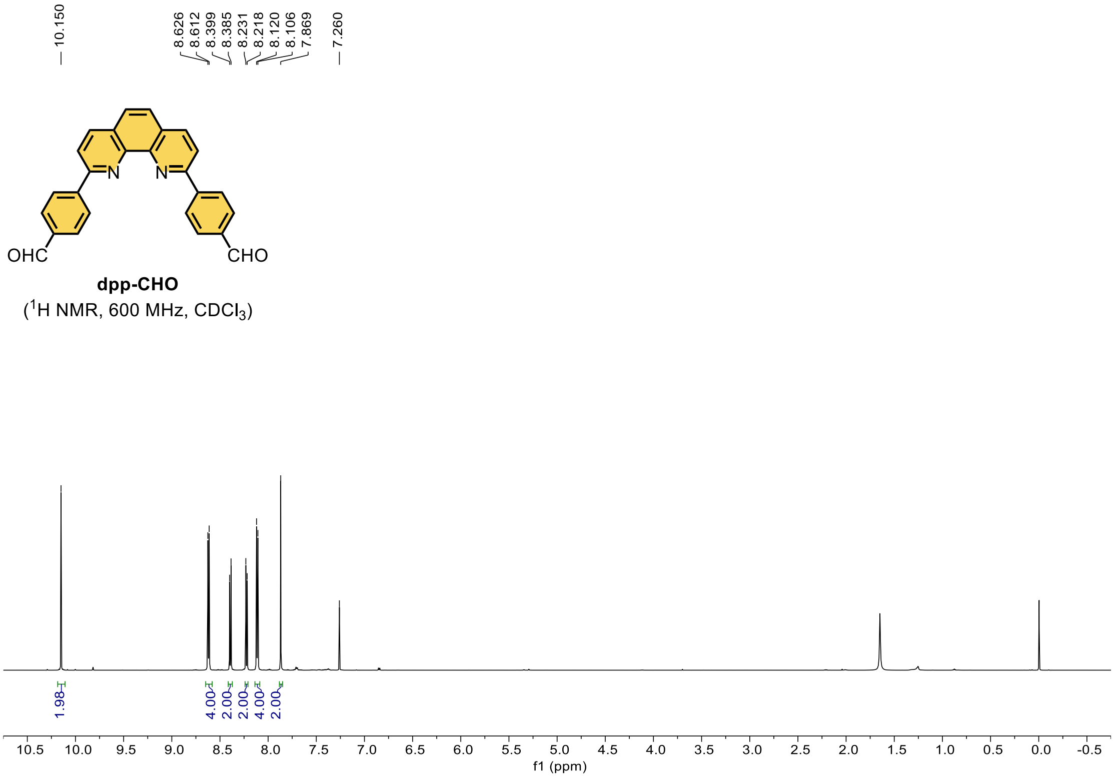

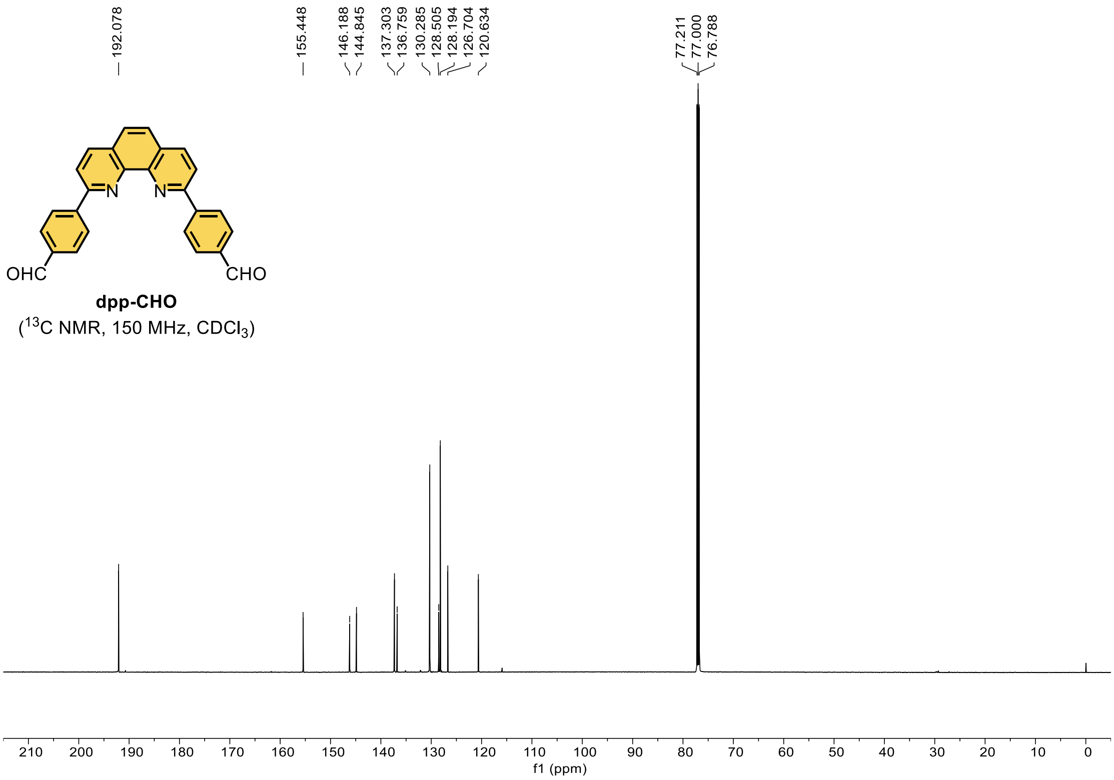

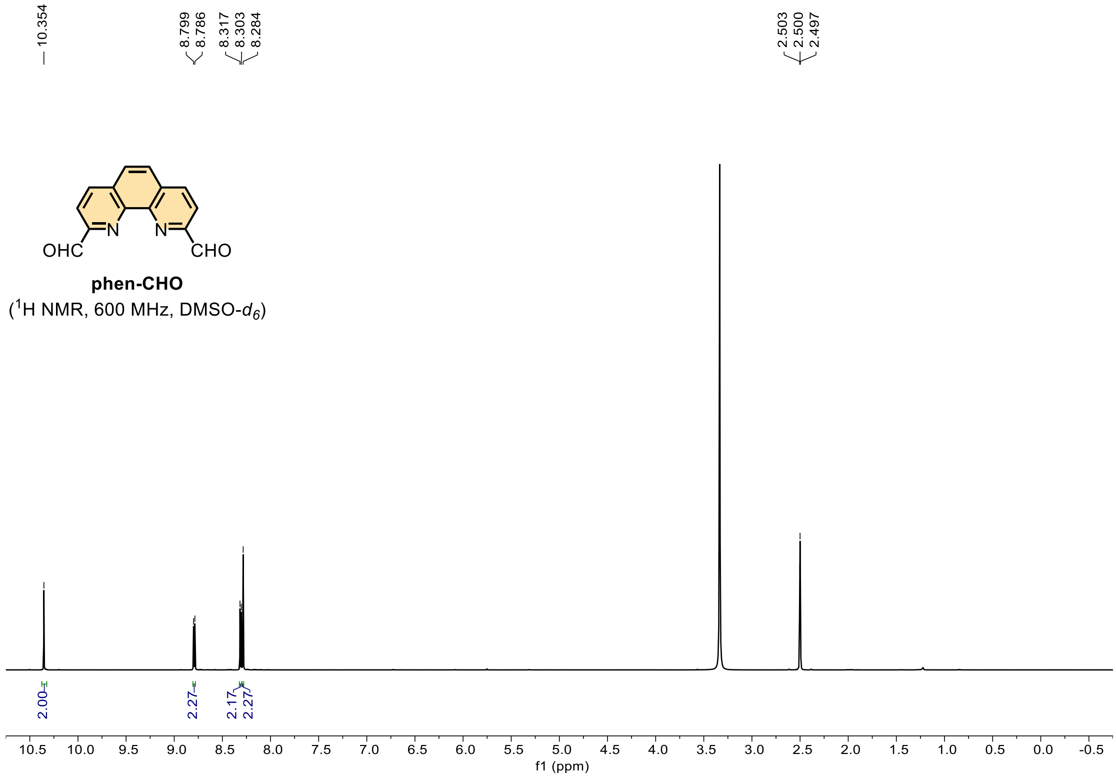
10.354
8.799
8.786
8.317
8.303
8.284
2.503
2.500
2.497
OHC
N
N
CHO
phen-CHO
(1H NMR, 600 MHz, DMSO-d6)
2.00
2.27
2.17
2.27
10.5 10.0 9.5 9.0 8.5 8.0 7.5 7.0 6.5 6.0 5.5 5.0 4.5 4.0 3.5 3.0 2.5 2.0 1.5 1.0 0.5 0.0 -0.5
f1 (ppm)

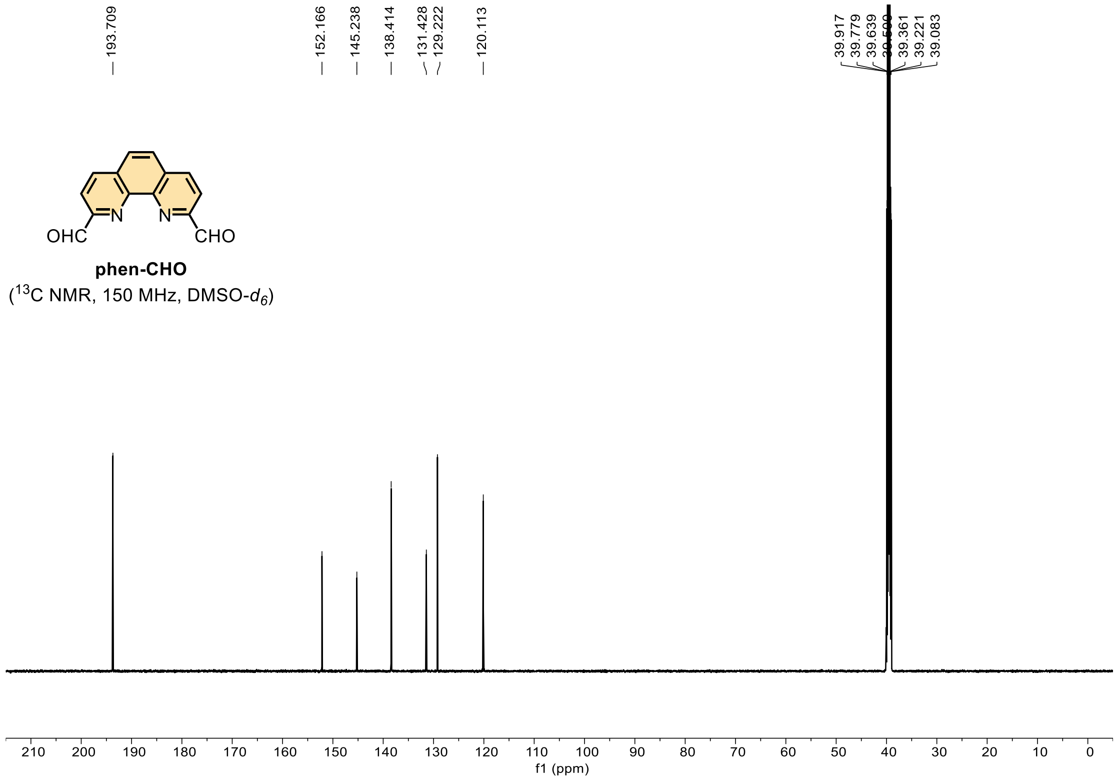
193.709
152.166
145.238
138.414
131.428
129.222
120.113
39.917
39.779
39.639
39.361
39.221
39.083
OHC
N
N
CHO
phen-CHO
(13C NMR, 150 MHz, DMSO-d6)
210 200 190 180 170 160 150 140 130 120 110 100 90 80 70 60 50 40 30 20 10 0
f1 (ppm)

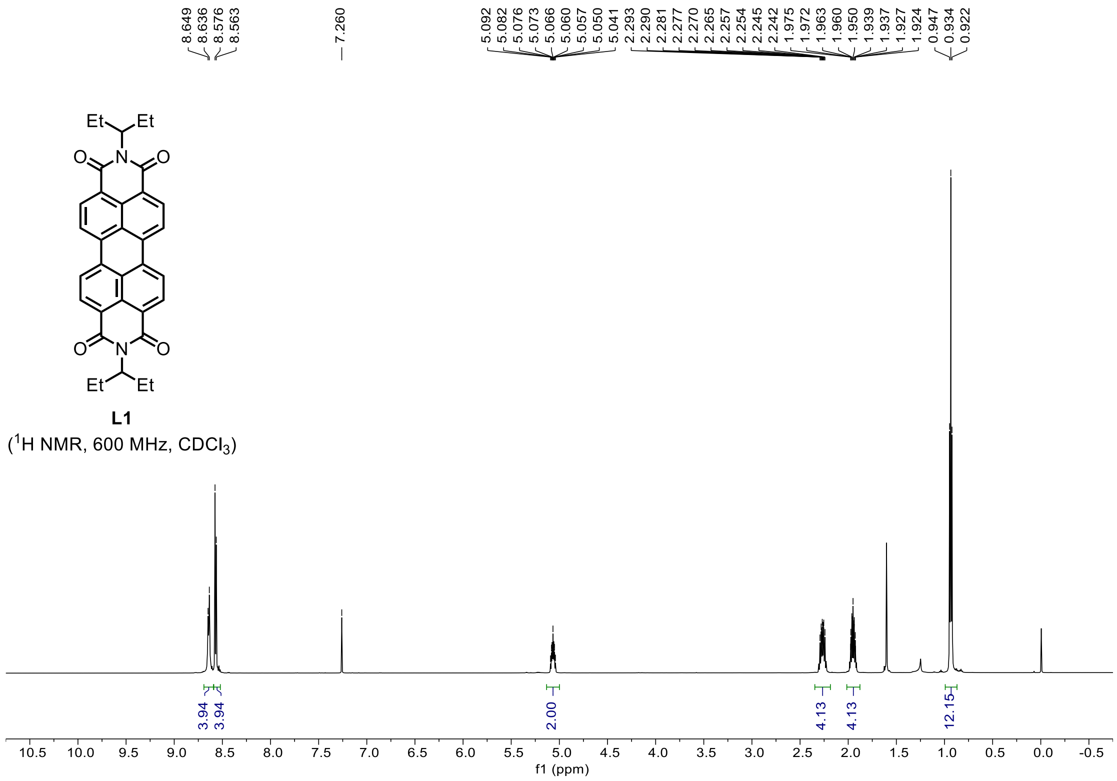
L1
(^{1}H NMR, 600 MHz, CDCl$_3$)
f1 (ppm)

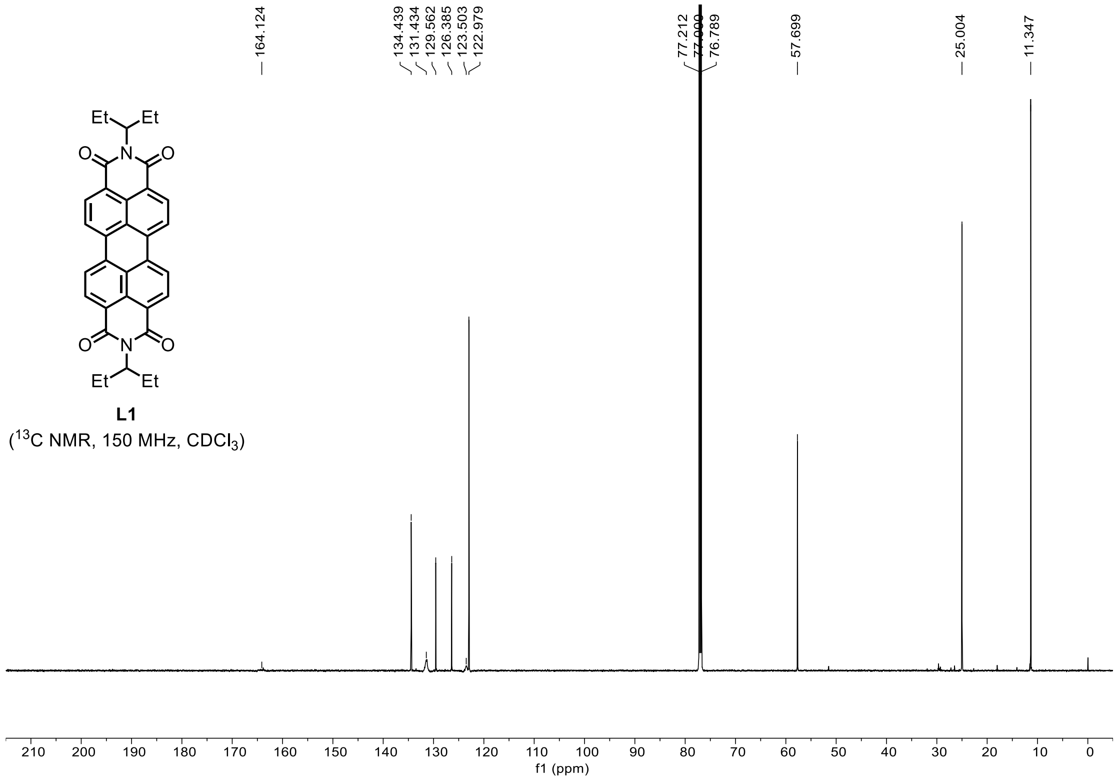
L1
(^{13}C NMR, 150 MHz, CDCl$_3$)
f1 (ppm)

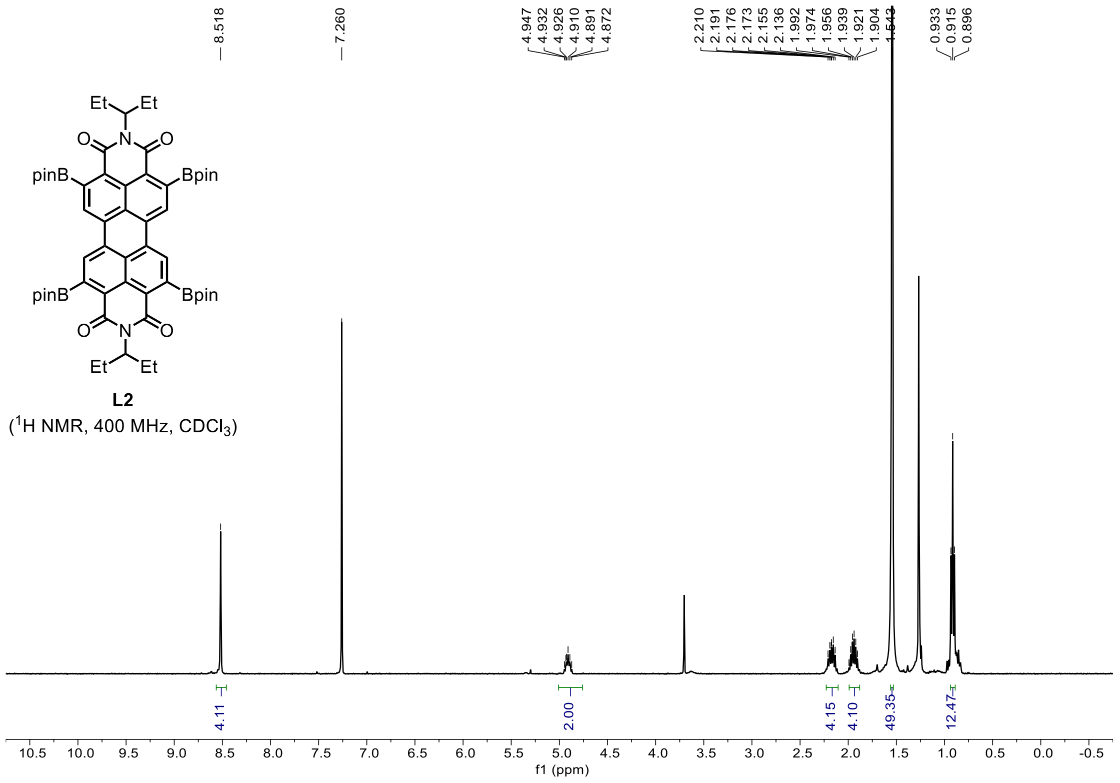
L2
(^{1}H NMR, 400 MHz, $CDCl_3$)
8.518
7.260
4.947
4.932
4.926
4.910
4.891
4.872
2.210
2.191
2.176
2.173
2.155
2.136
1.992
1.974
1.956
1.939
1.921
1.904
1.542
0.933
0.915
0.896
4.11
2.00
4.15
4.10
49.35
12.47
10.5 10.0 9.5 9.0 8.5 8.0 7.5 7.0 6.5 6.0 5.5 5.0 4.5 4.0 3.5 3.0 2.5 2.0 1.5 1.0 0.5 0.0 -0.5
f1 (ppm)

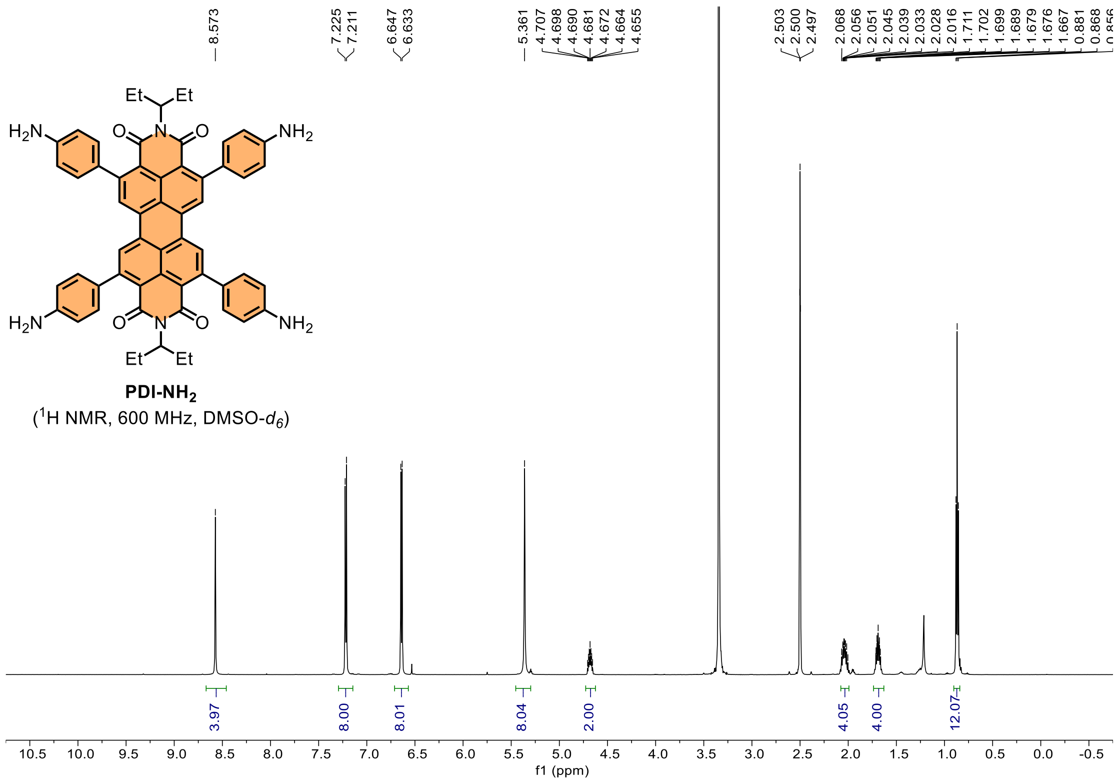
PDI-NH$_2$
(^{1}H NMR, 600 MHz, DMSO-d_6)
8.573
7.225
7.211
6.647
6.633
5.361
4.707
4.698
4.690
4.681
4.672
4.664
4.655
2.503
2.500
2.497
2.068
2.056
2.051
2.045
2.039
2.033
2.028
2.016
1.711
1.702
1.699
1.689
1.679
1.676
1.667
0.881
0.868
[illegible]
3.97
8.00
8.01
8.04
2.00
4.05
4.00
12.07
10.5 10.0 9.5 9.0 8.5 8.0 7.5 7.0 6.5 6.0 5.5 5.0 4.5 4.0 3.5 3.0 2.5 2.0 1.5 1.0 0.5 0.0 -0.5
f1 (ppm)

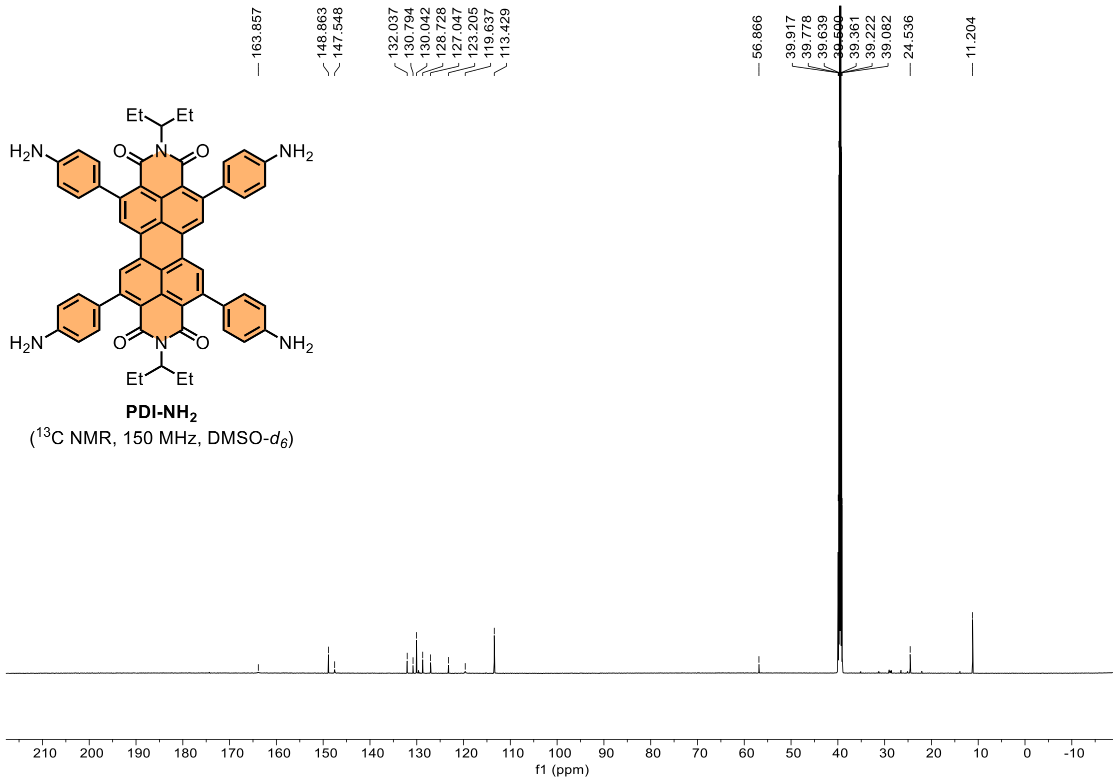

PDI-NH$_2$
(^{13}C NMR, 150 MHz, DMSO-d_6)

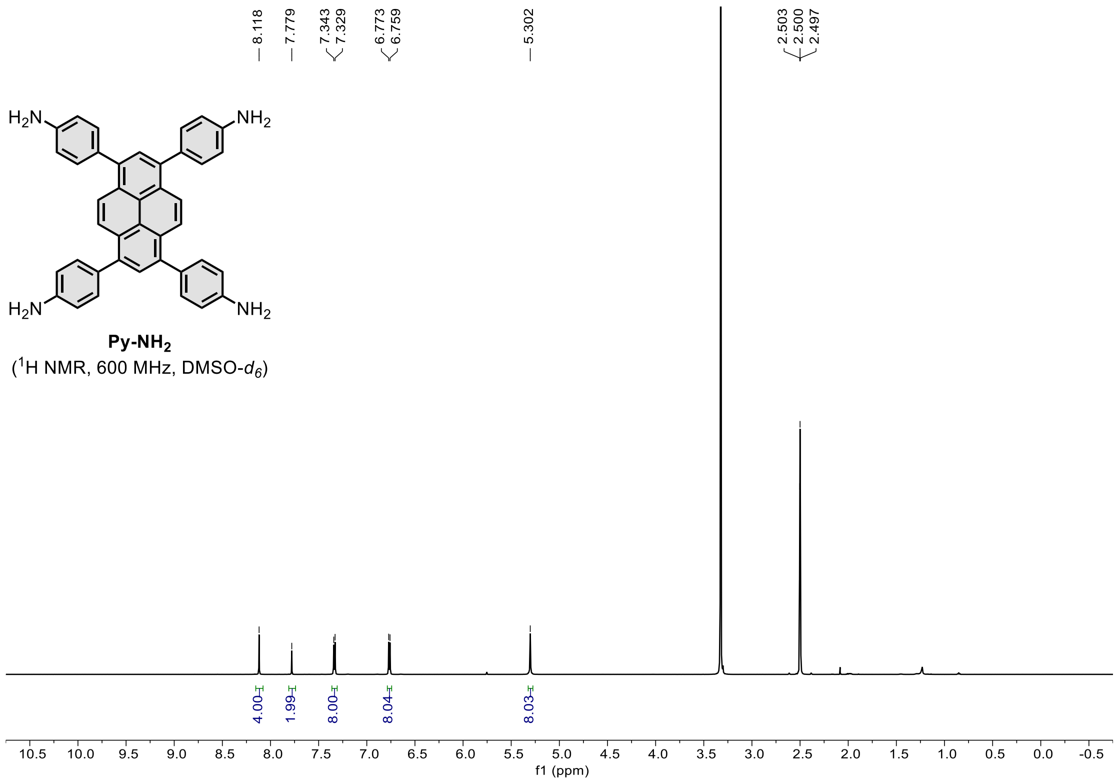

Py-NH$_2$
(^{1}H NMR, 600 MHz, DMSO-d_6)

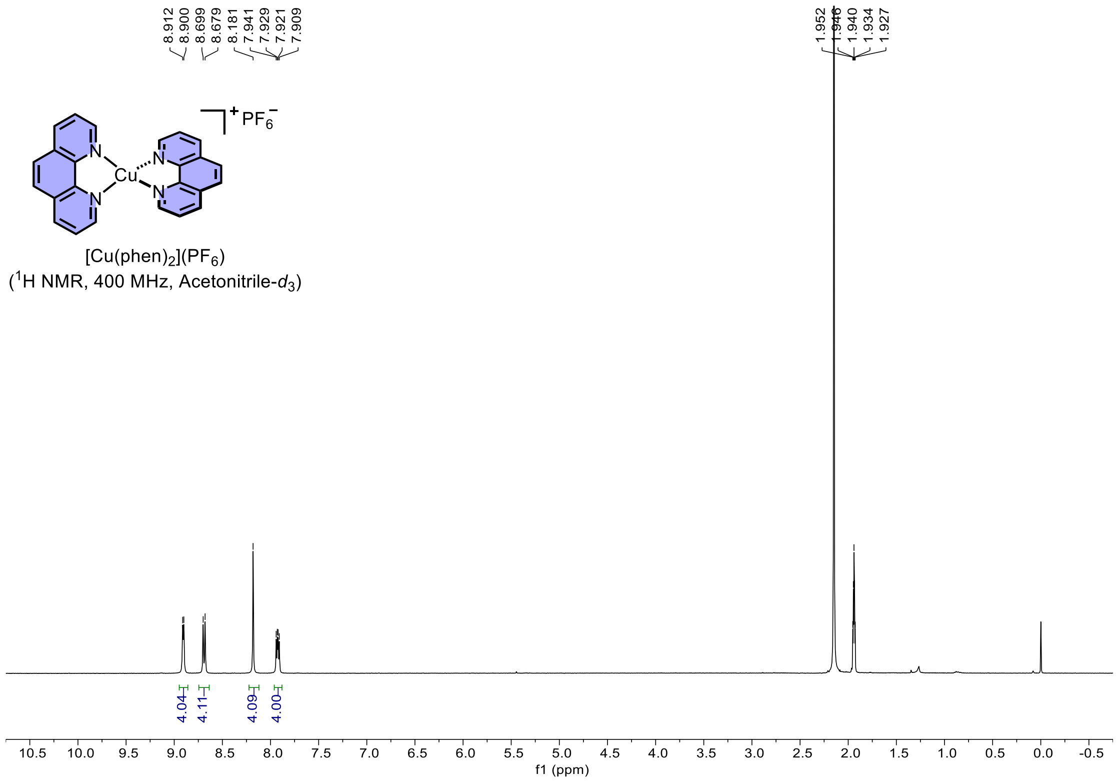

8.912
8.900
8.699
8.679
8.181
7.941
7.929
7.921
7.909
1.952
1.946
1.940
1.934
1.927
+ PF6−
N
Cu
N
N
N
[Cu(phen)2](PF6)
(1H NMR, 400 MHz, Acetonitrile-d3)
4.04
4.11
4.09
4.00
10.5 10.0 9.5 9.0 8.5 8.0 7.5 7.0 6.5 6.0 5.5 5.0 4.5 4.0 3.5 3.0 2.5 2.0 1.5 1.0 0.5 0.0 -0.5
f1 (ppm)

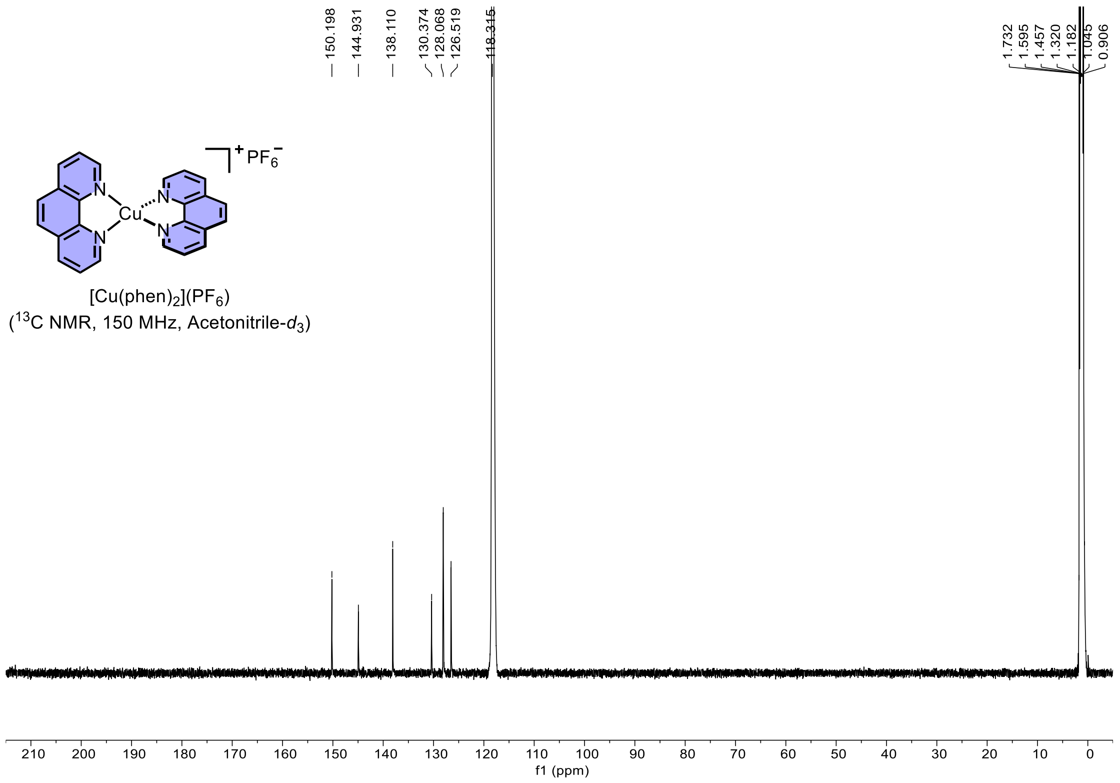

150.198
144.931
138.110
130.374
128.068
126.519
118.315
1.732
1.595
1.457
1.320
1.182
1.045
0.906
+ PF6−
N
Cu
N
N
N
[Cu(phen)2](PF6)
(13C NMR, 150 MHz, Acetonitrile-d3)
210 200 190 180 170 160 150 140 130 120 110 100 90 80 70 60 50 40 30 20 10 0
f1 (ppm)

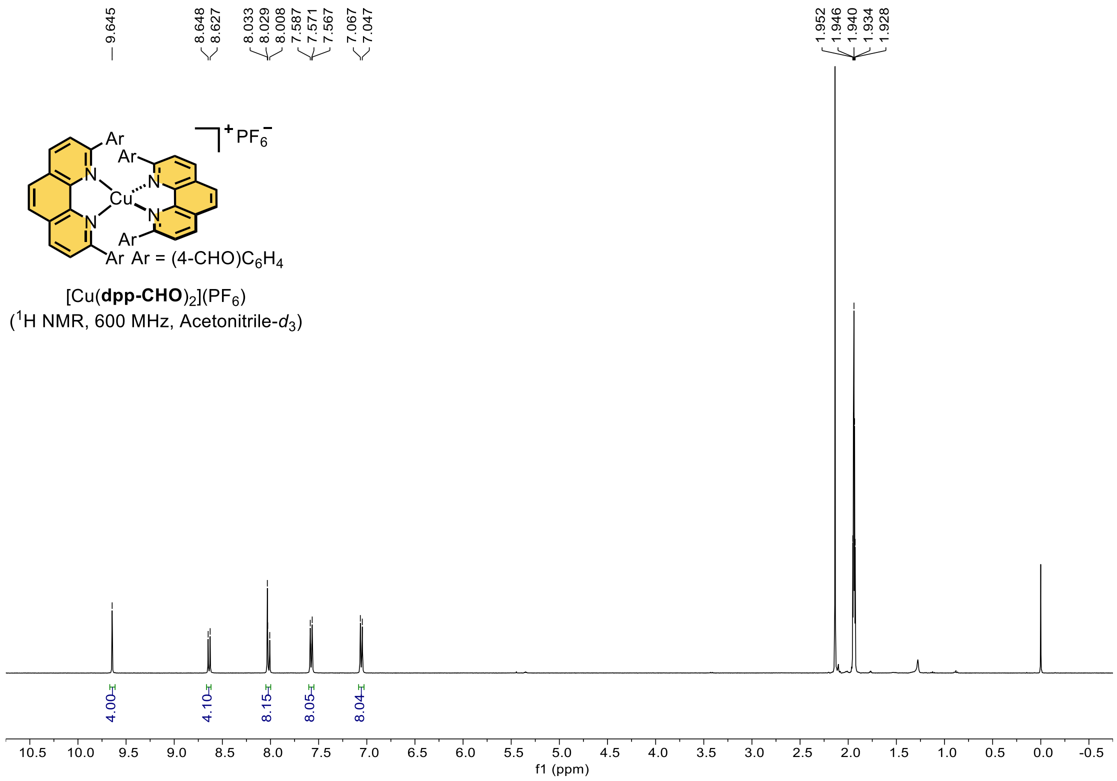
9.645
8.648
8.627
8.033
8.029
8.008
7.587
7.571
7.567
7.067
7.047
1.952
1.946
1.940
1.934
1.928
Ar
Ar
N
N
Cu
N
N
Ar
Ar
+ PF6−
Ar = (4-CHO)C6H4
[Cu(dpp-CHO)2](PF6)
(1H NMR, 600 MHz, Acetonitrile-d3)
4.00
4.10
8.15
8.05
8.04
10.5 10.0 9.5 9.0 8.5 8.0 7.5 7.0 6.5 6.0 5.5 5.0 4.5 4.0 3.5 3.0 2.5 2.0 1.5 1.0 0.5 0.0 -0.5
f1 (ppm)

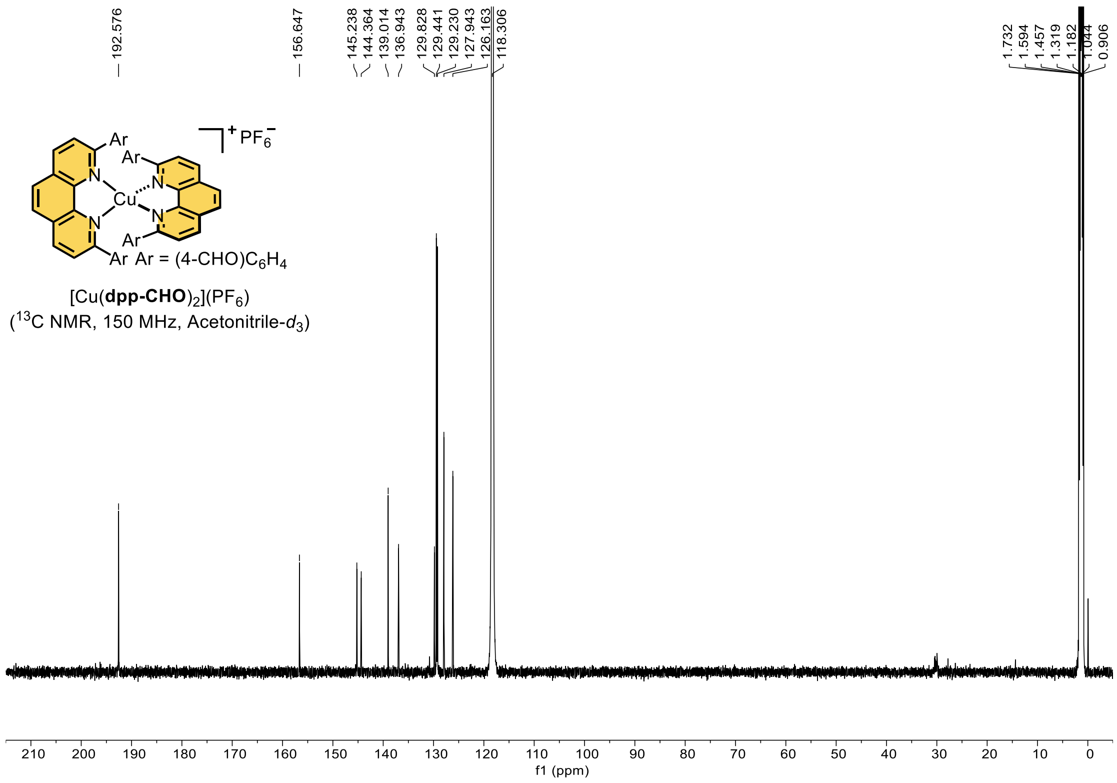
192.576
156.647
145.238
144.364
139.014
136.943
129.828
129.441
129.230
127.943
126.163
118.306
1.732
1.594
1.457
1.319
1.182
1.044
0.906
Ar
Ar
N
N
Cu
N
N
Ar
Ar
+ PF6−
Ar = (4-CHO)C6H4
[Cu(dpp-CHO)2](PF6)
(13C NMR, 150 MHz, Acetonitrile-d3)
210 200 190 180 170 160 150 140 130 120 110 100 90 80 70 60 50 40 30 20 10 0
f1 (ppm)

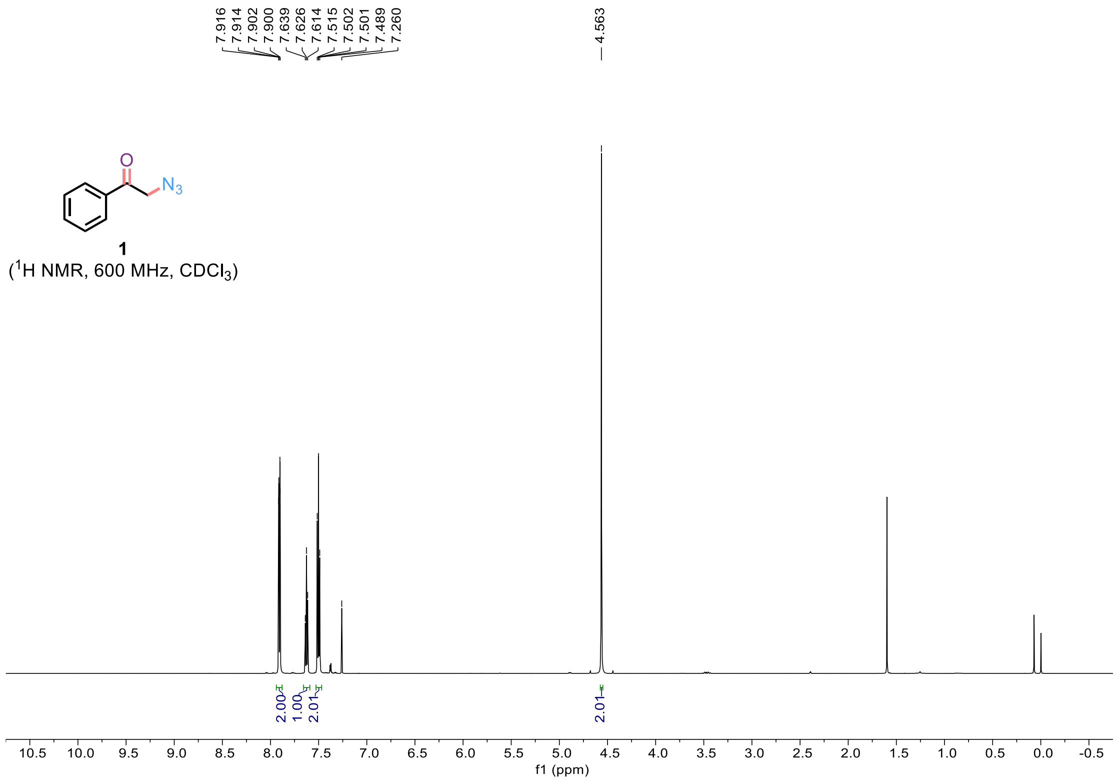
7.916
7.914
7.902
7.900
7.639
7.626
7.614
7.515
7.502
7.501
7.489
7.260
4.563
O
N3
1
(1H NMR, 600 MHz, CDCl3)
2.00
1.00
2.01
2.01
10.5 10.0 9.5 9.0 8.5 8.0 7.5 7.0 6.5 6.0 5.5 5.0 4.5 4.0 3.5 3.0 2.5 2.0 1.5 1.0 0.5 0.0 -0.5
f1 (ppm)

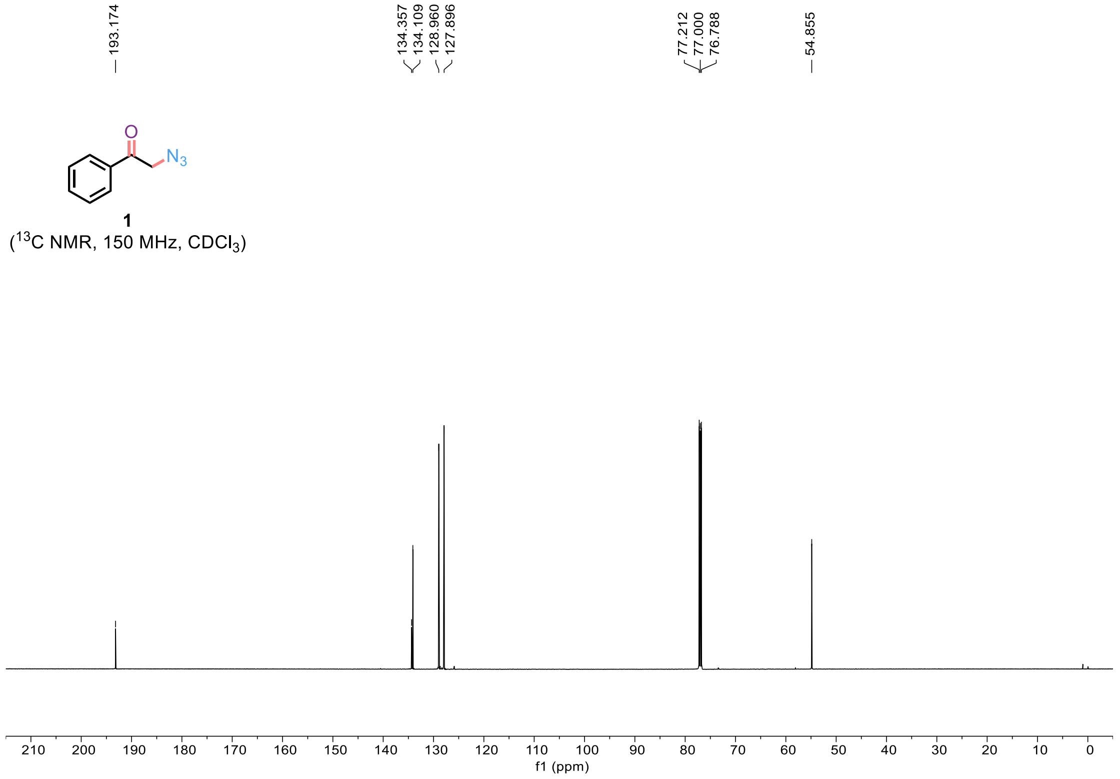
193.174
134.357
134.109
128.960
127.896
77.212
77.000
76.788
54.855
O
N3
1
(13C NMR, 150 MHz, CDCl3)
210 200 190 180 170 160 150 140 130 120 110 100 90 80 70 60 50 40 30 20 10 0
f1 (ppm)

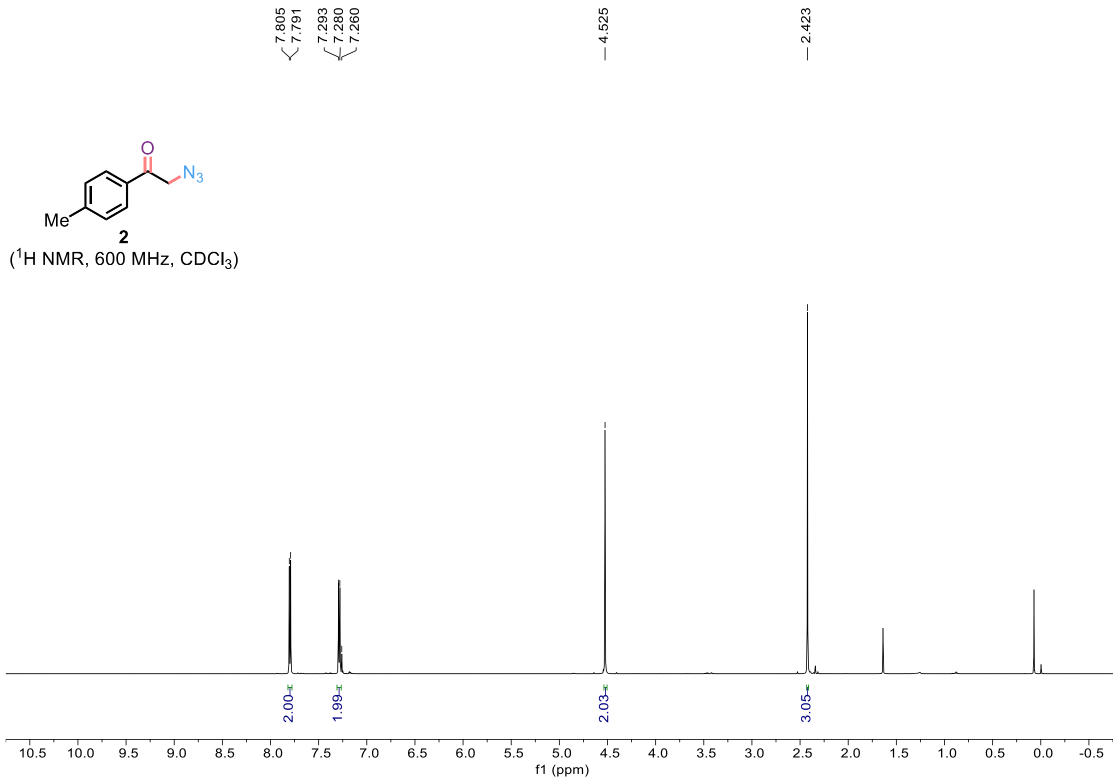
7.805
7.791
7.293
7.280
7.260
4.525
2.423
O
N3
Me
2
(1H NMR, 600 MHz, CDCl3)
2.00
1.99
2.03
3.05
10.5 10.0 9.5 9.0 8.5 8.0 7.5 7.0 6.5 6.0 5.5 5.0 4.5 4.0 3.5 3.0 2.5 2.0 1.5 1.0 0.5 0.0 -0.5
f1 (ppm)

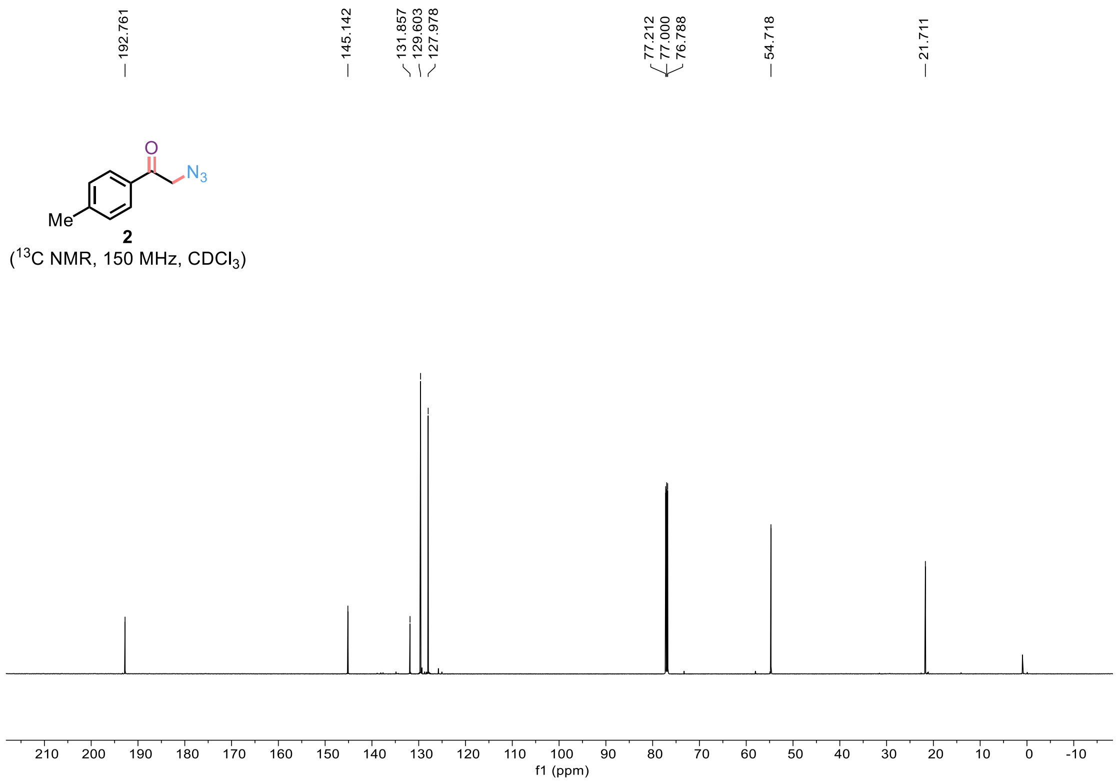
192.761
145.142
131.857
129.603
127.978
77.212
77.000
76.788
54.718
21.711
O
N3
Me
2
(13C NMR, 150 MHz, CDCl3)
210 200 190 180 170 160 150 140 130 120 110 100 90 80 70 60 50 40 30 20 10 0 -10
f1 (ppm)

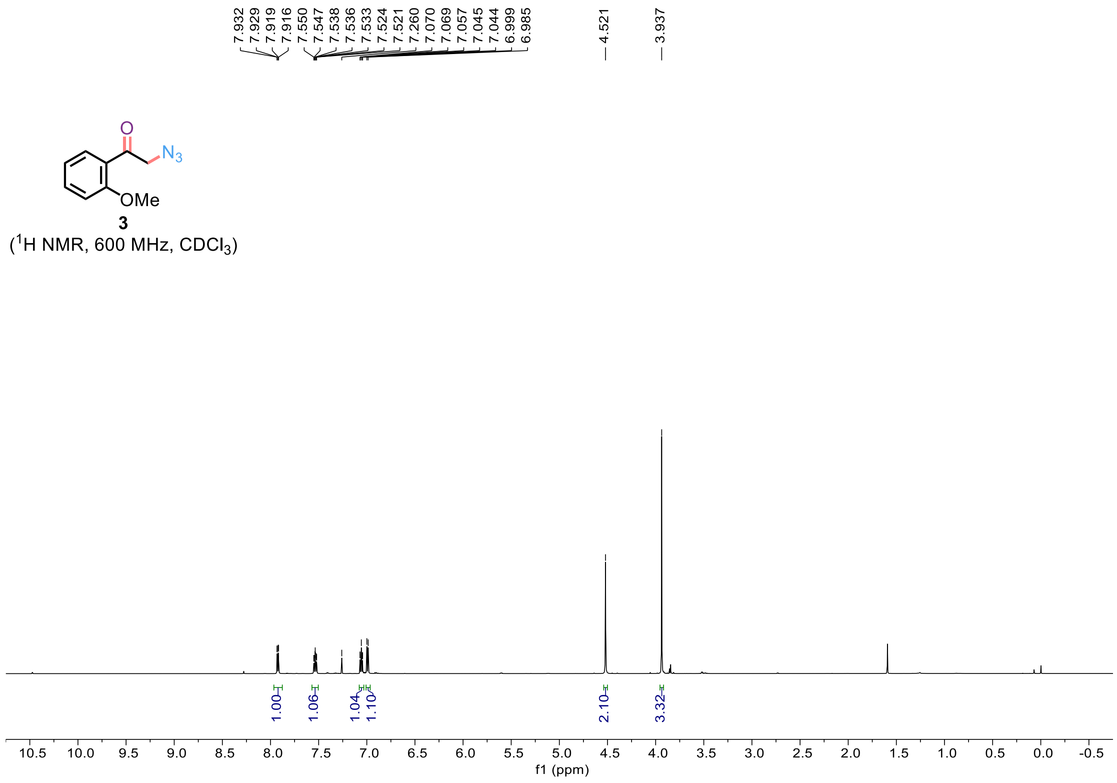

3

(^{1}H NMR, 600 MHz, $CDCl_3$)

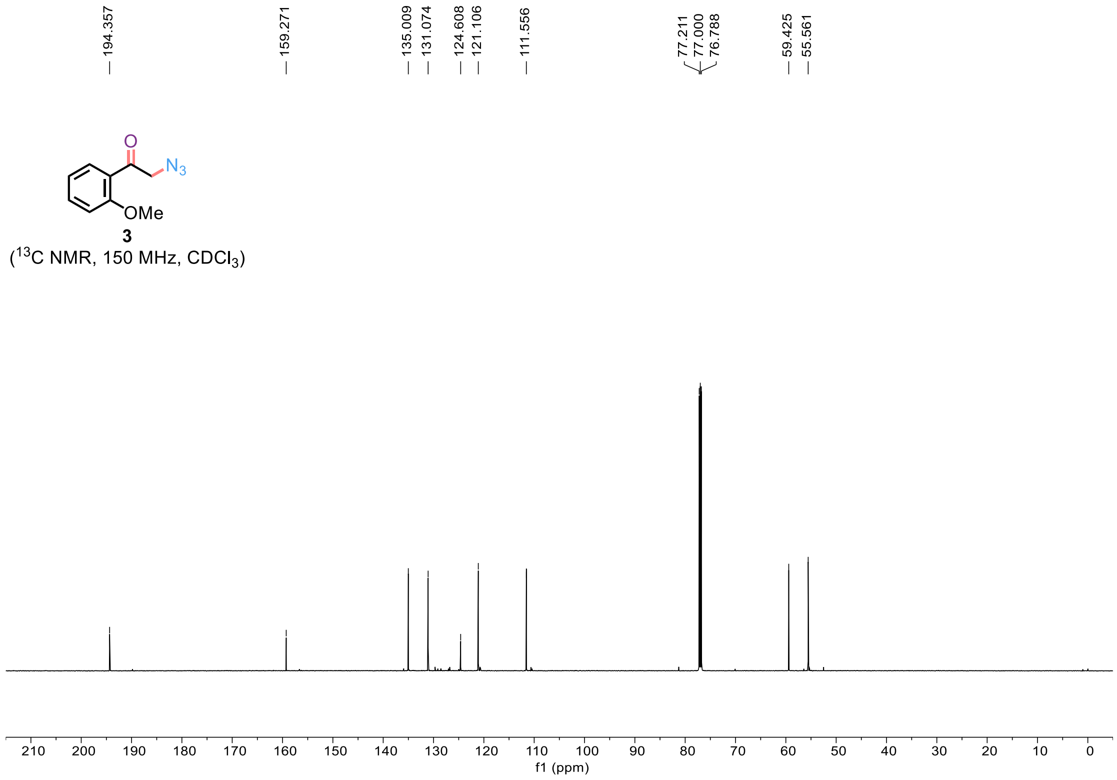

3

(^{13}C NMR, 150 MHz, $CDCl_3$)

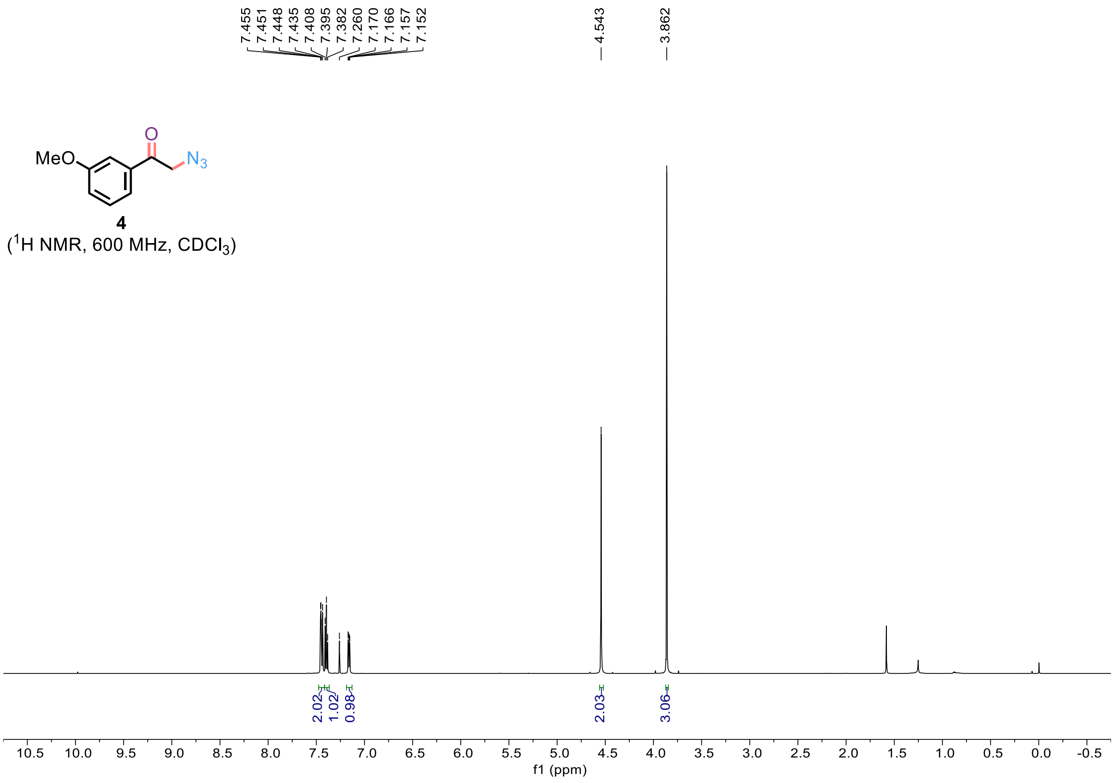
7.455
7.451
7.448
7.435
7.408
7.395
7.382
7.260
7.170
7.166
7.157
7.152
4.543
3.862
MeO
O
N3
4
(1H NMR, 600 MHz, CDCl3)
2.02
1.02
0.98
2.03
3.06
f1 (ppm)

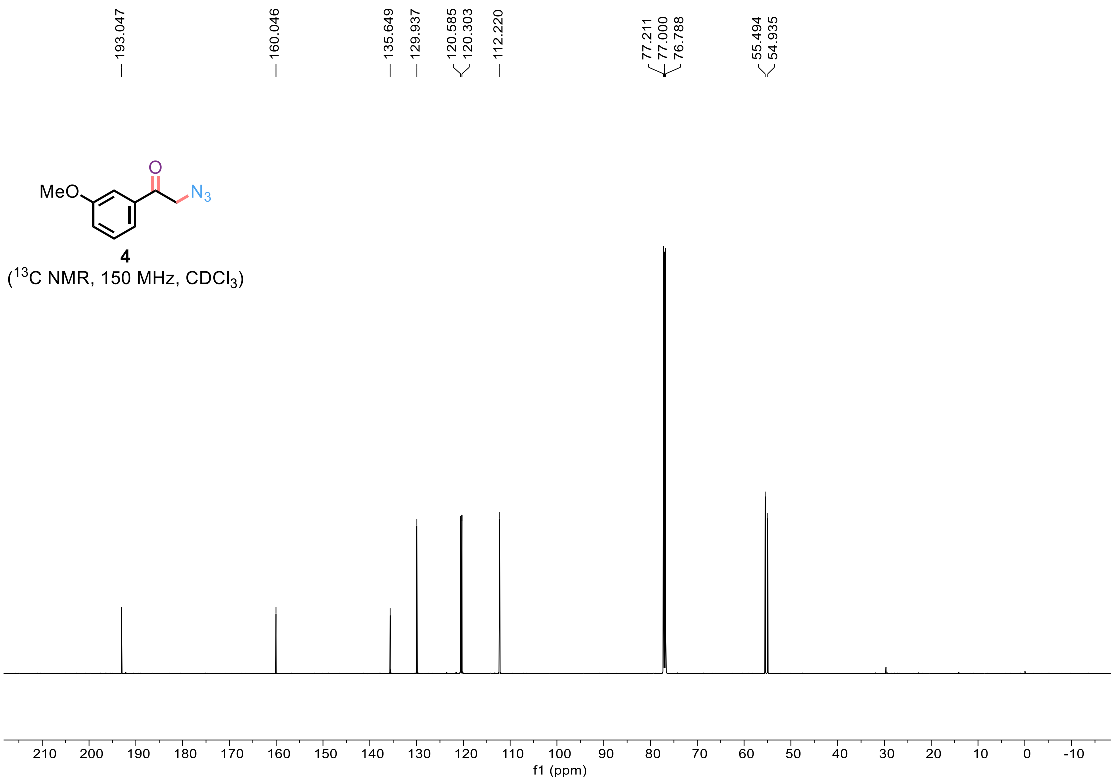
193.047
160.046
135.649
129.937
120.585
120.303
112.220
77.211
77.000
76.788
55.494
54.935
MeO
O
N3
4
(13C NMR, 150 MHz, CDCl3)
f1 (ppm)

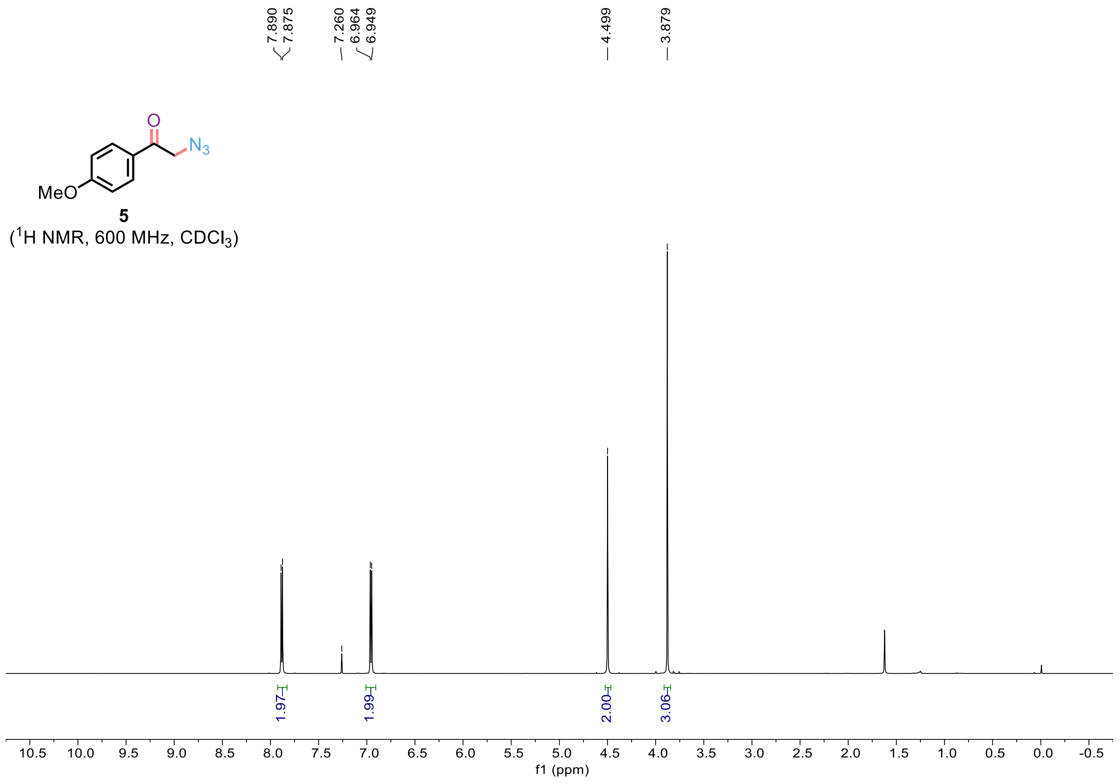

5
(^{1}H NMR, 600 MHz, $CDCl_3$)

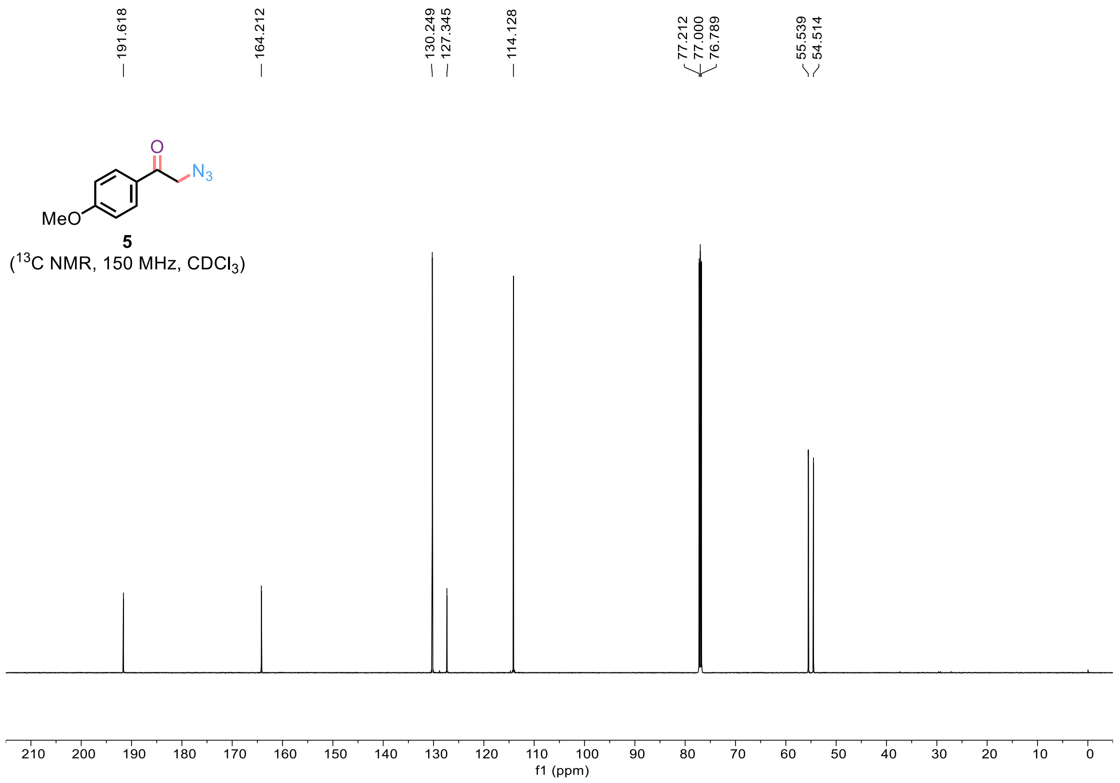

5
(^{13}C NMR, 150 MHz, $CDCl_3$)

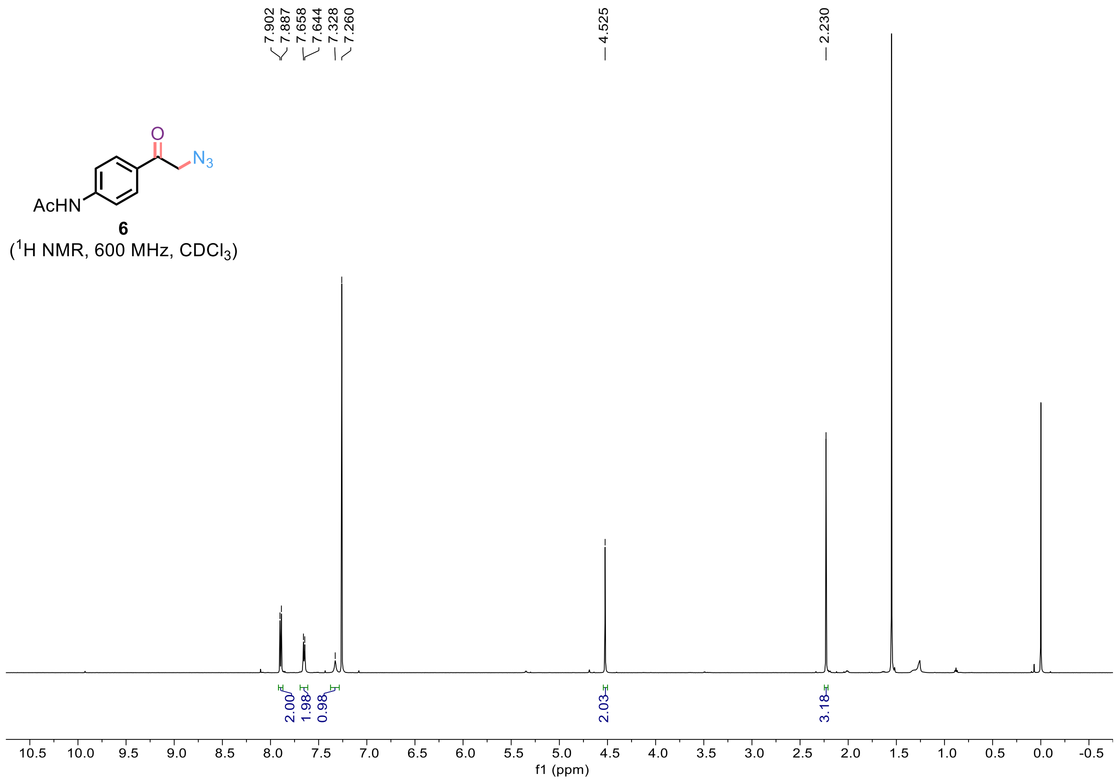
7.902
7.887
7.658
7.644
7.328
7.260
4.525
2.230
O
N3
AcHN
6
(1H NMR, 600 MHz, CDCl3)
2.00
1.98
0.98
2.03
3.18
10.5 10.0 9.5 9.0 8.5 8.0 7.5 7.0 6.5 6.0 5.5 5.0 4.5 4.0 3.5 3.0 2.5 2.0 1.5 1.0 0.5 0.0 -0.5
f1 (ppm)

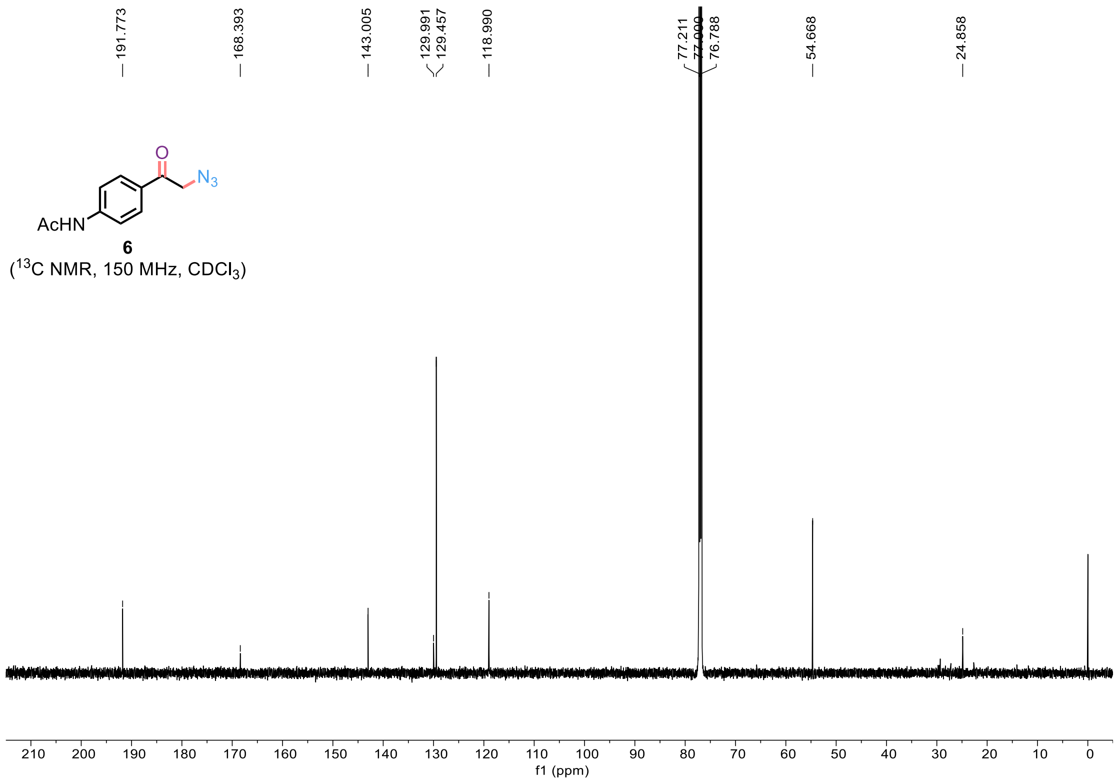
191.773
168.393
143.005
129.991
129.457
118.990
77.211
77.000
76.788
54.668
24.858
O
N3
AcHN
6
(13C NMR, 150 MHz, CDCl3)
210 200 190 180 170 160 150 140 130 120 110 100 90 80 70 60 50 40 30 20 10 0
f1 (ppm)

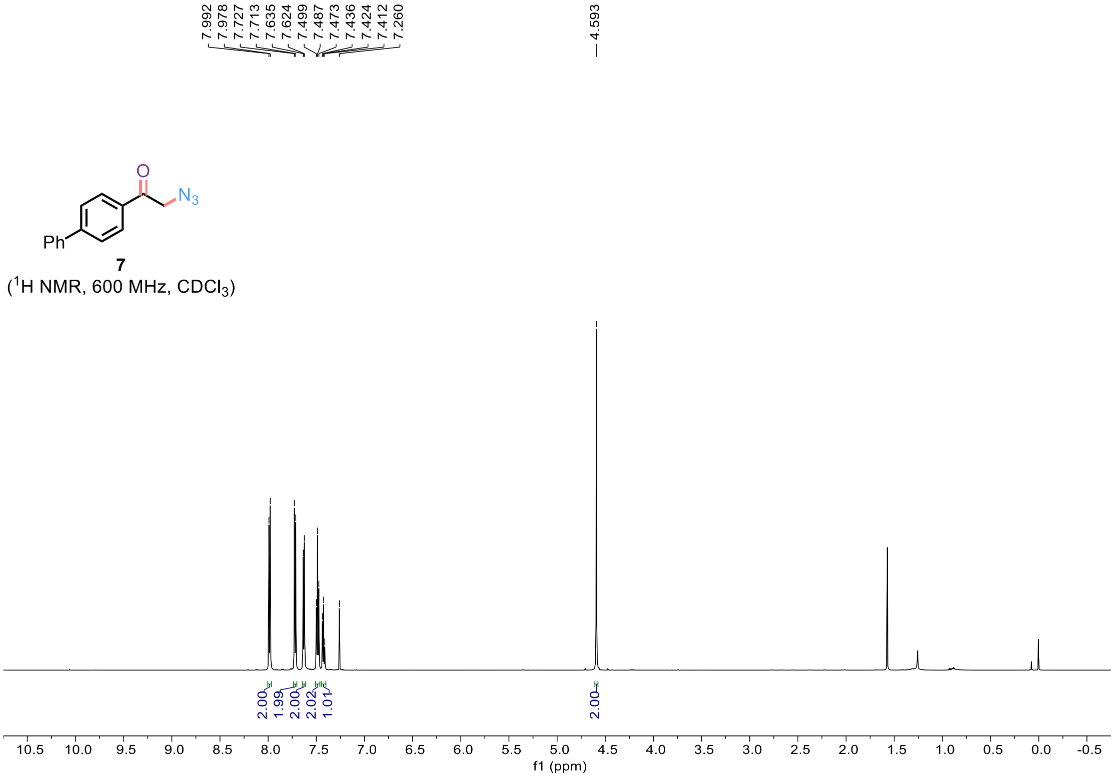

7
(^{1}H NMR, 600 MHz, $CDCl_3$)

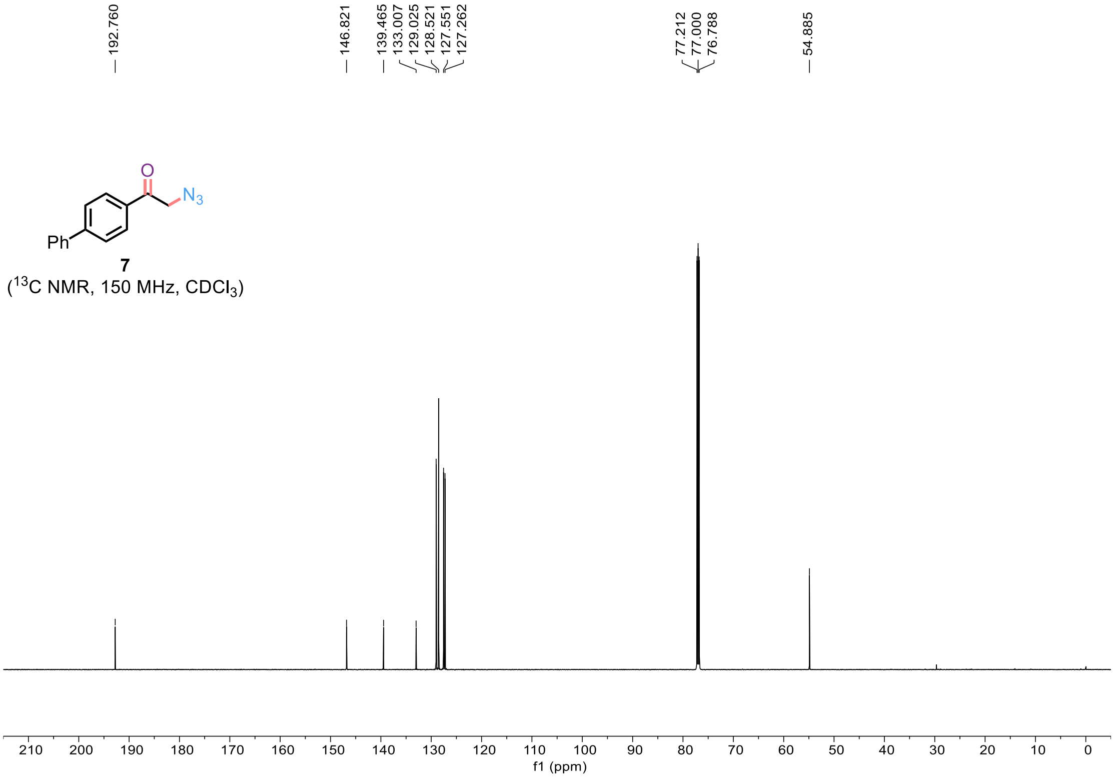

7
(^{13}C NMR, 150 MHz, $CDCl_3$)

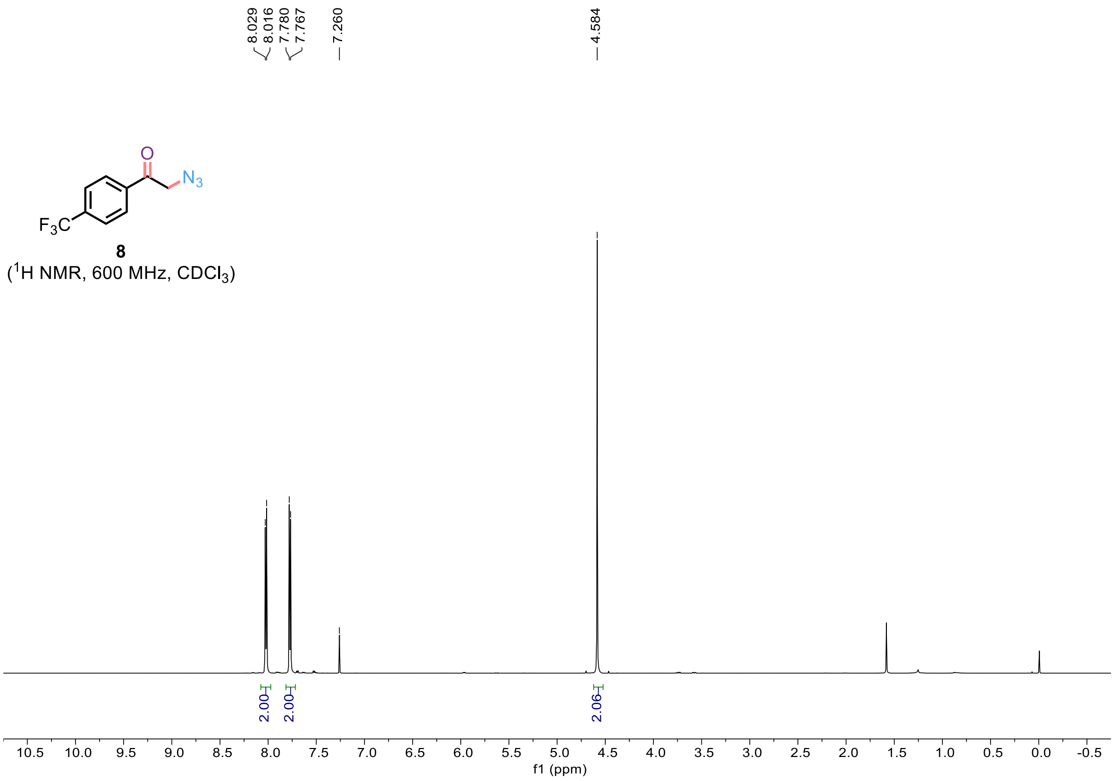

8

(^{1}H NMR, 600 MHz, $CDCl_3$)

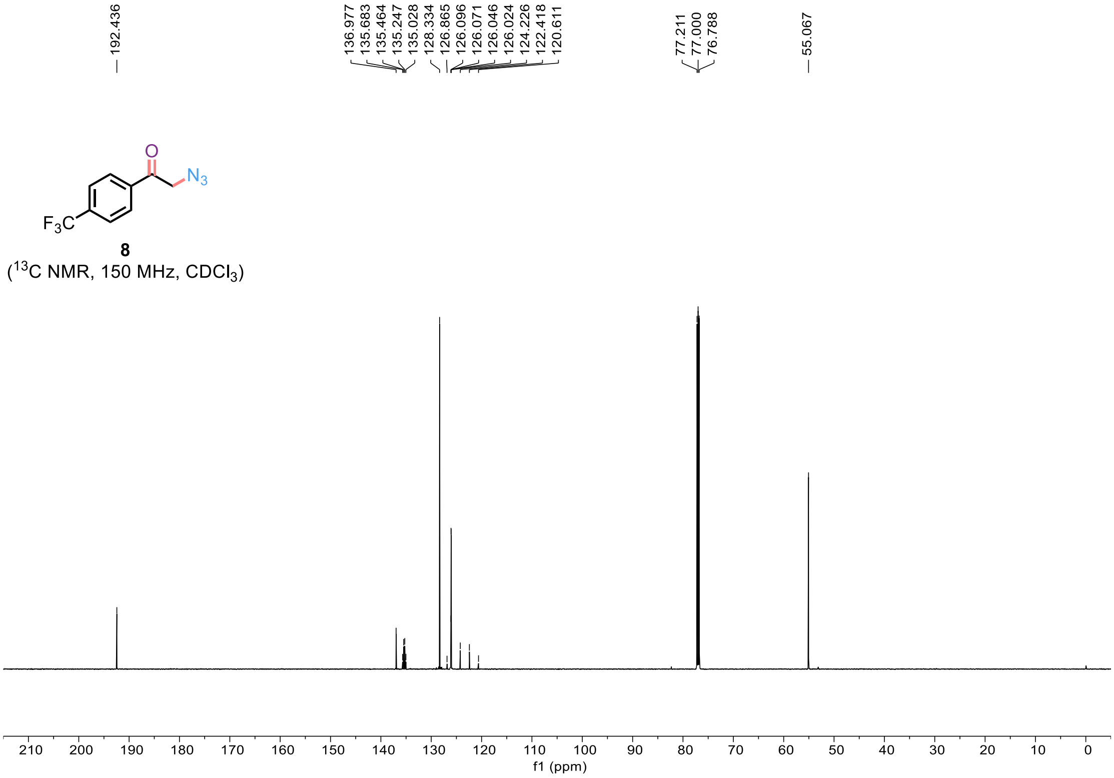

8

(^{13}C NMR, 150 MHz, $CDCl_3$)

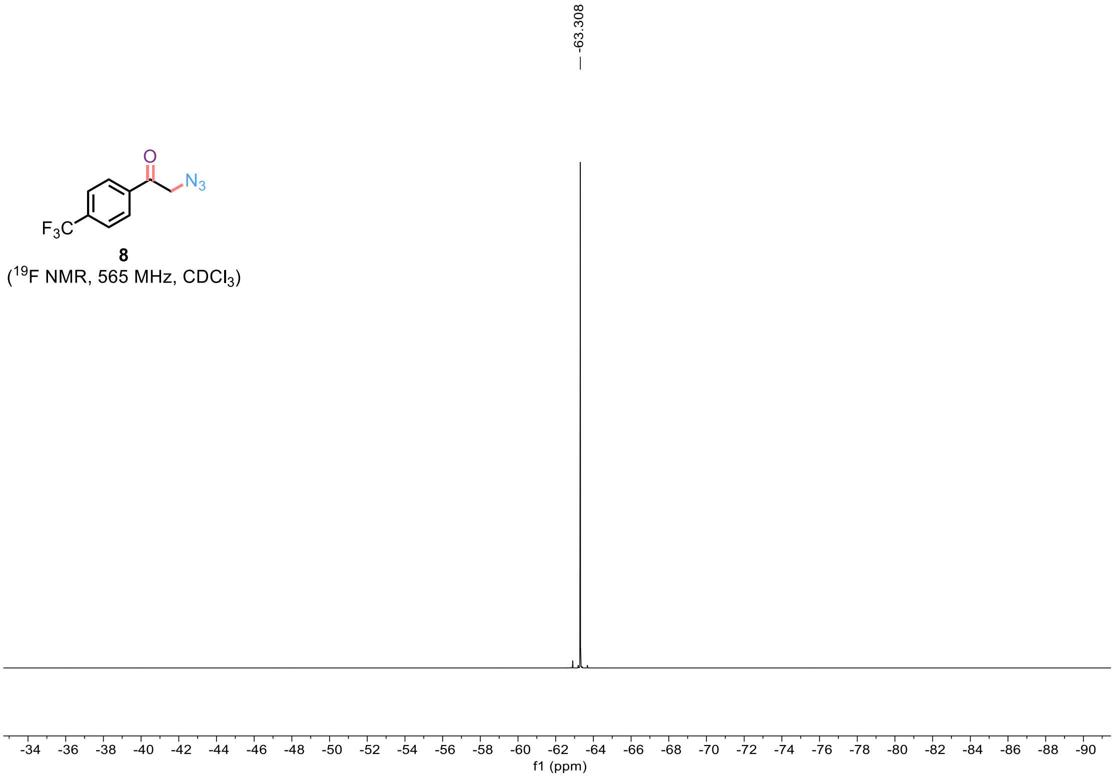

8
(^{19}F NMR, 565 MHz, $CDCl_3$)

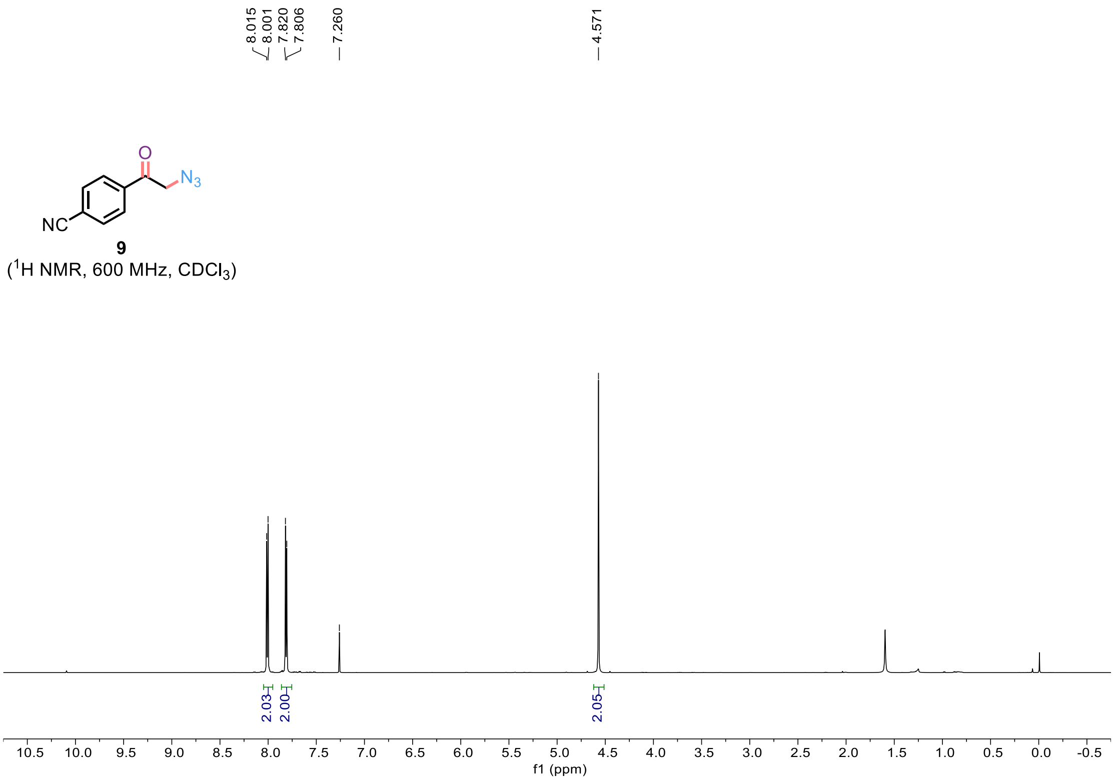

9
(^{1}H NMR, 600 MHz, $CDCl_3$)

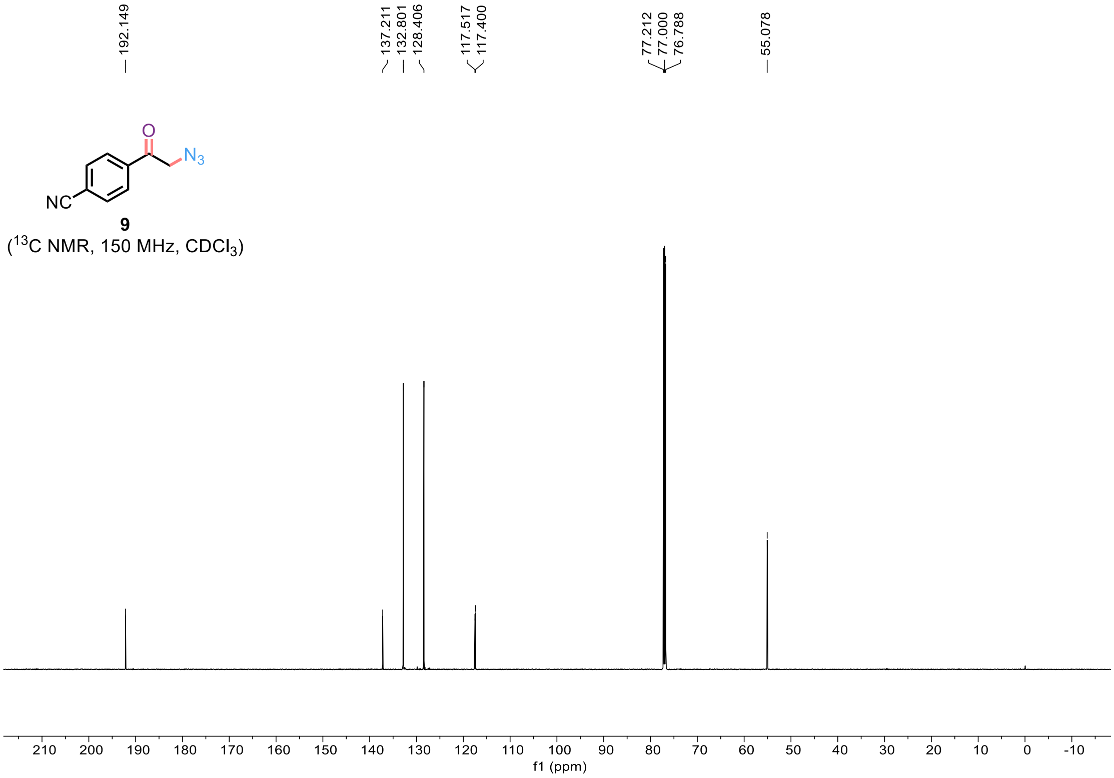

9
(^{13}C NMR, 150 MHz, $CDCl_3$)

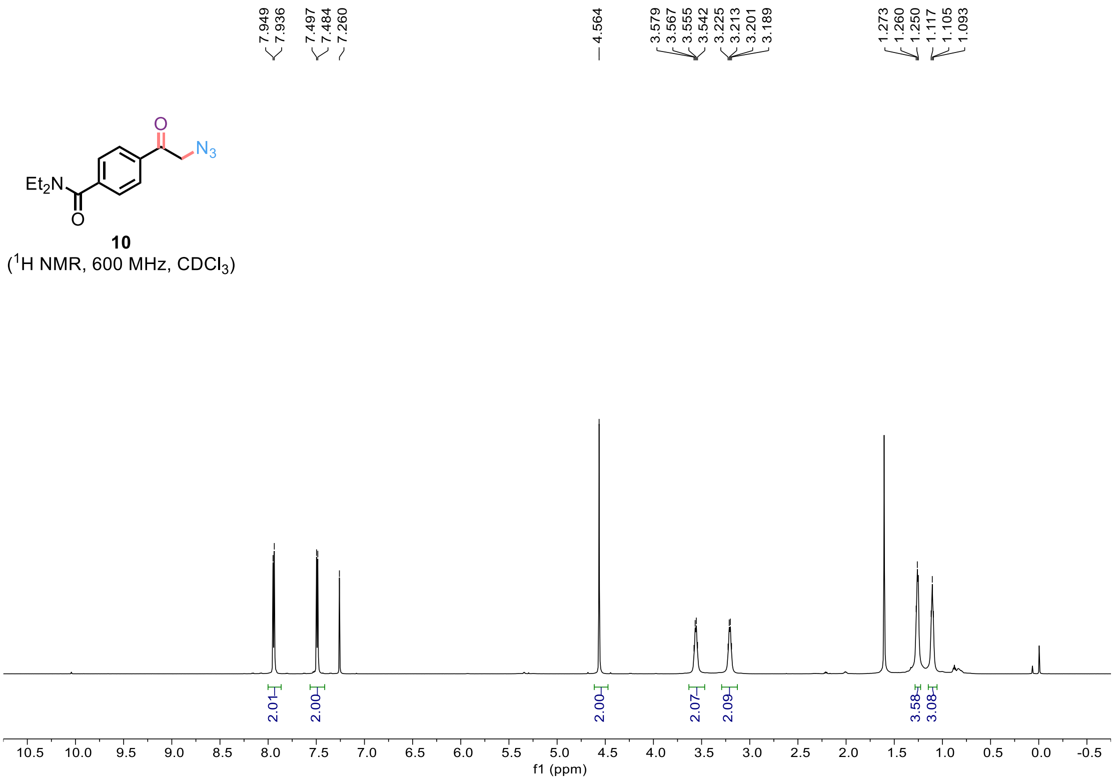
7.949
7.936
7.497
7.484
7.260
4.564
3.579
3.567
3.555
3.542
3.225
3.213
3.201
3.189
1.273
1.260
1.250
1.117
1.105
1.093
Et2N
O
N3
O
10
(1H NMR, 600 MHz, CDCl3)
2.01
2.00
2.00
2.07
2.09
3.58
3.08
f1 (ppm)

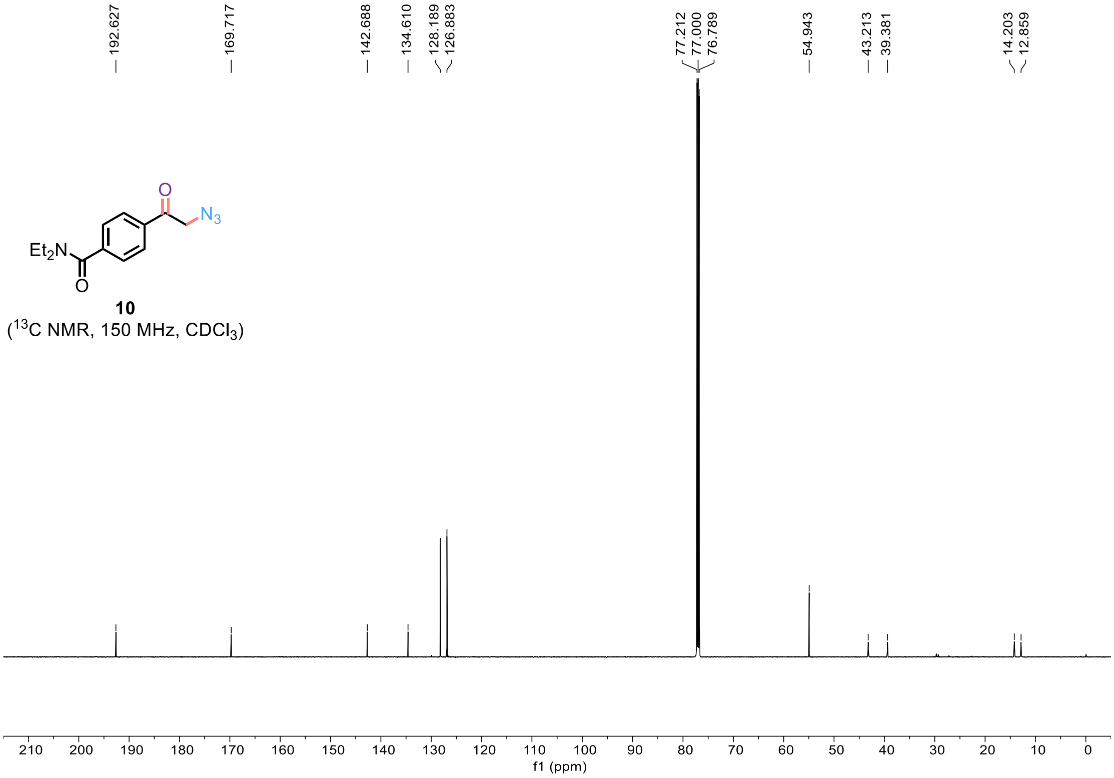
192.627
169.717
142.688
134.610
128.189
126.883
77.212
77.000
76.789
54.943
43.213
39.381
14.203
12.859
Et2N
O
N3
O
10
(13C NMR, 150 MHz, CDCl3)
f1 (ppm)

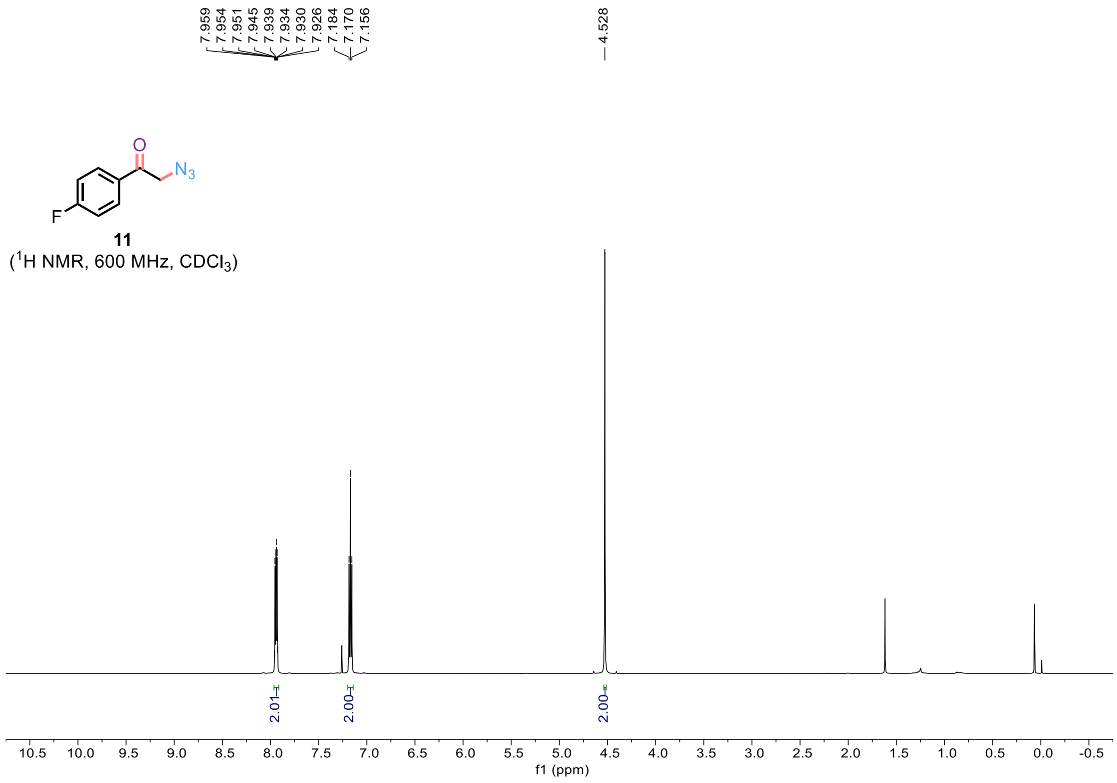
7.959
7.954
7.951
7.945
7.939
7.934
7.930
7.926
7.184
7.170
7.156
4.528
O
N3
F
11
(1H NMR, 600 MHz, CDCl3)
2.01
2.00
2.00
10.5 10.0 9.5 9.0 8.5 8.0 7.5 7.0 6.5 6.0 5.5 5.0 4.5 4.0 3.5 3.0 2.5 2.0 1.5 1.0 0.5 0.0 -0.5
f1 (ppm)

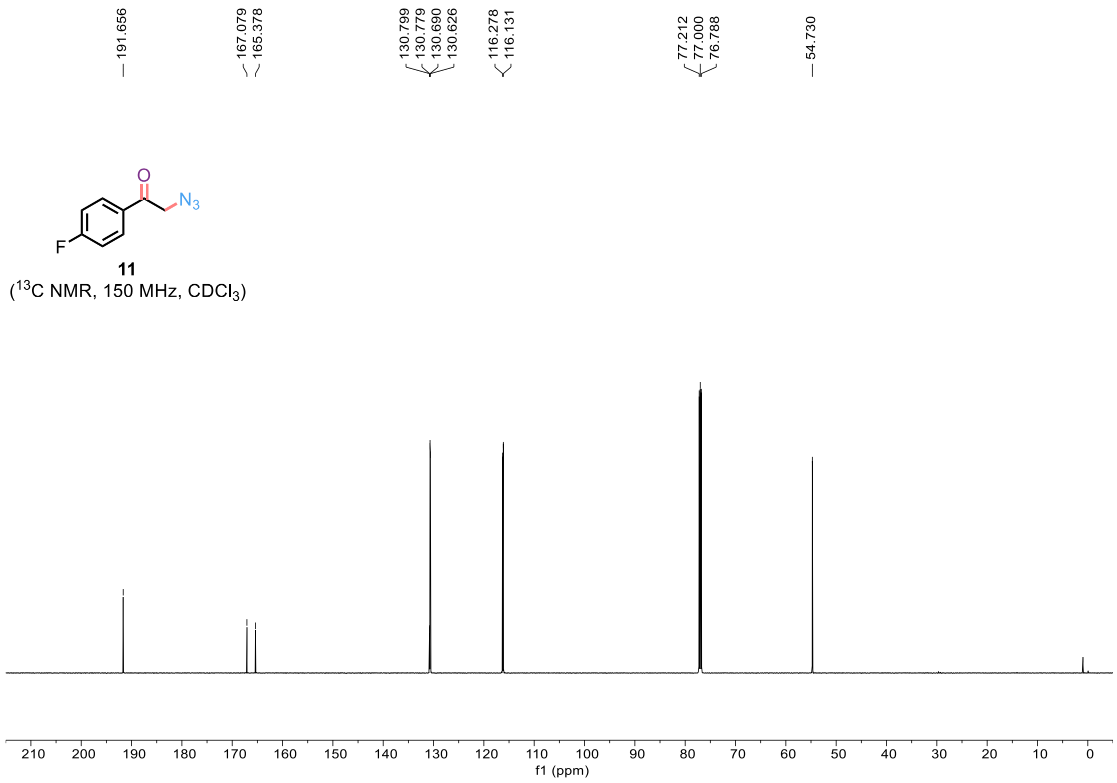
191.656
167.079
165.378
130.799
130.779
130.690
130.626
116.278
116.131
77.212
77.000
76.788
54.730
O
N3
F
11
(13C NMR, 150 MHz, CDCl3)
210 200 190 180 170 160 150 140 130 120 110 100 90 80 70 60 50 40 30 20 10 0
f1 (ppm)

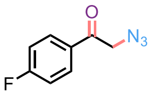

11
(^{19}F NMR, 565 MHz, $CDCl_3$)

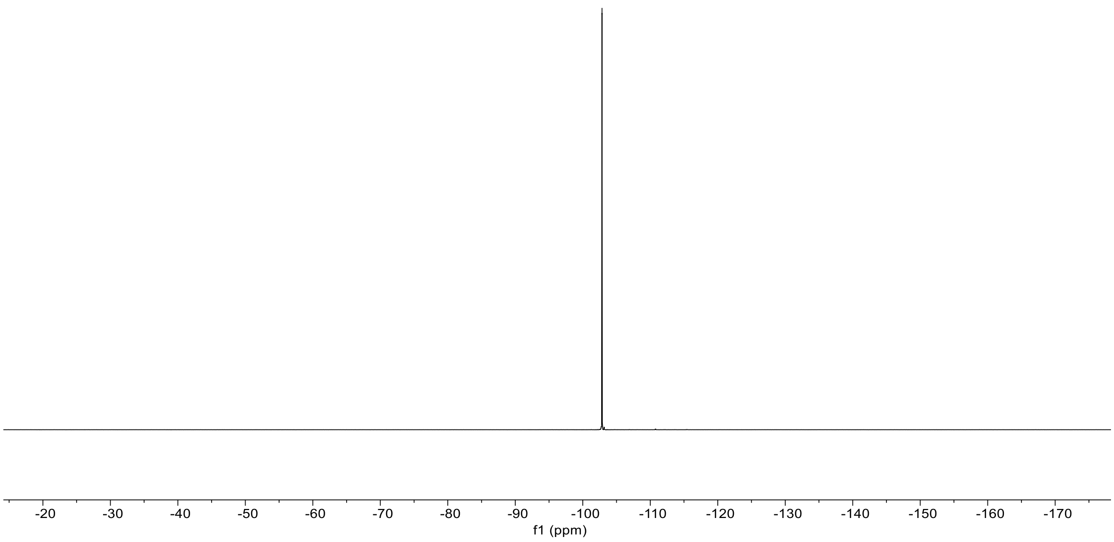

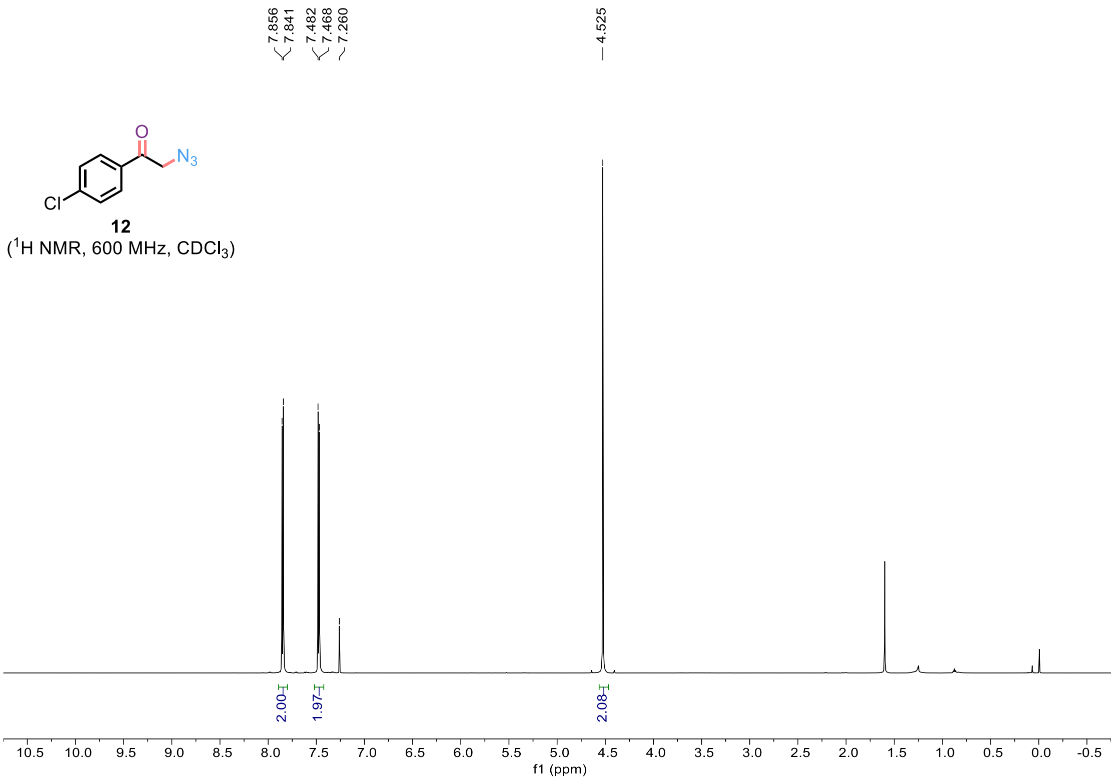

12
(^{1}H NMR, 600 MHz, $CDCl_3$)

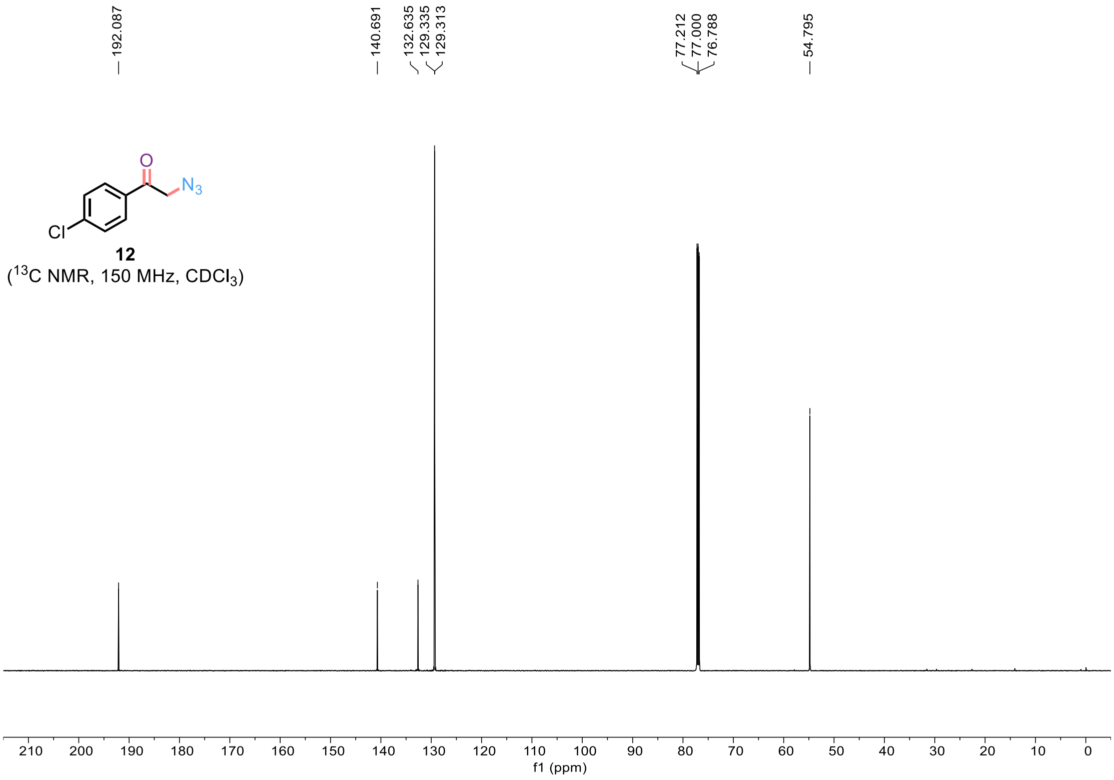

12
(^{13}C NMR, 150 MHz, $CDCl_3$)

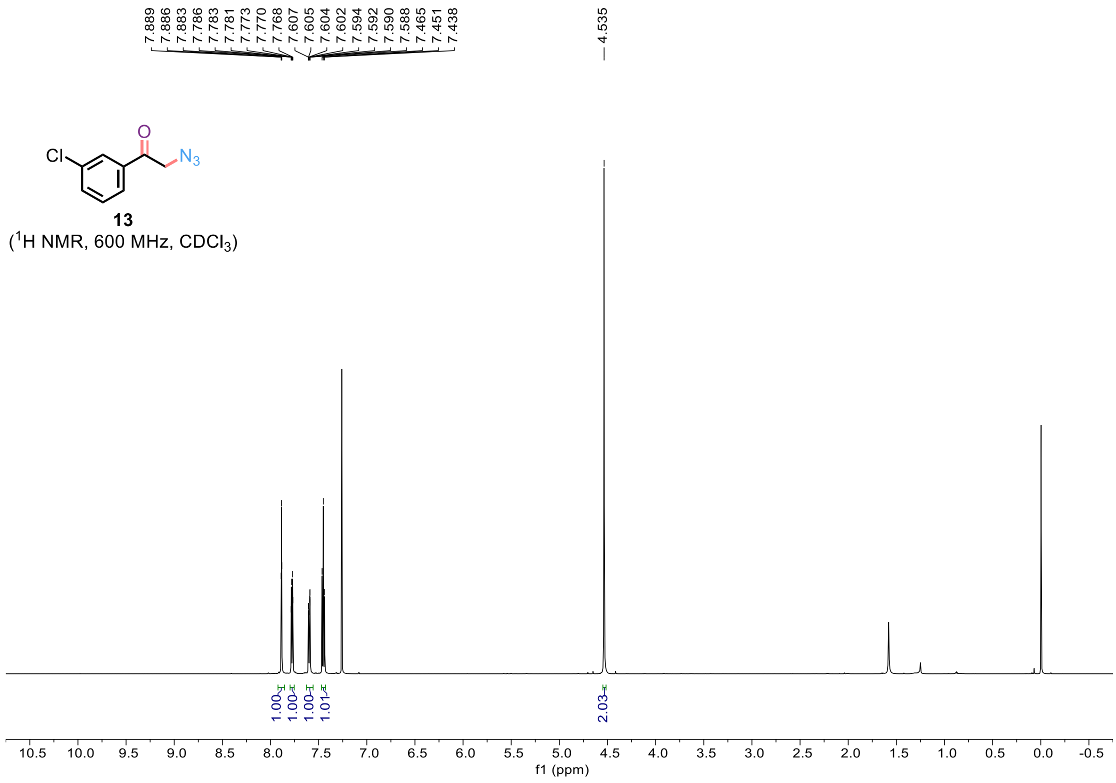

13
(^{1}H NMR, 600 MHz, $CDCl_3$)

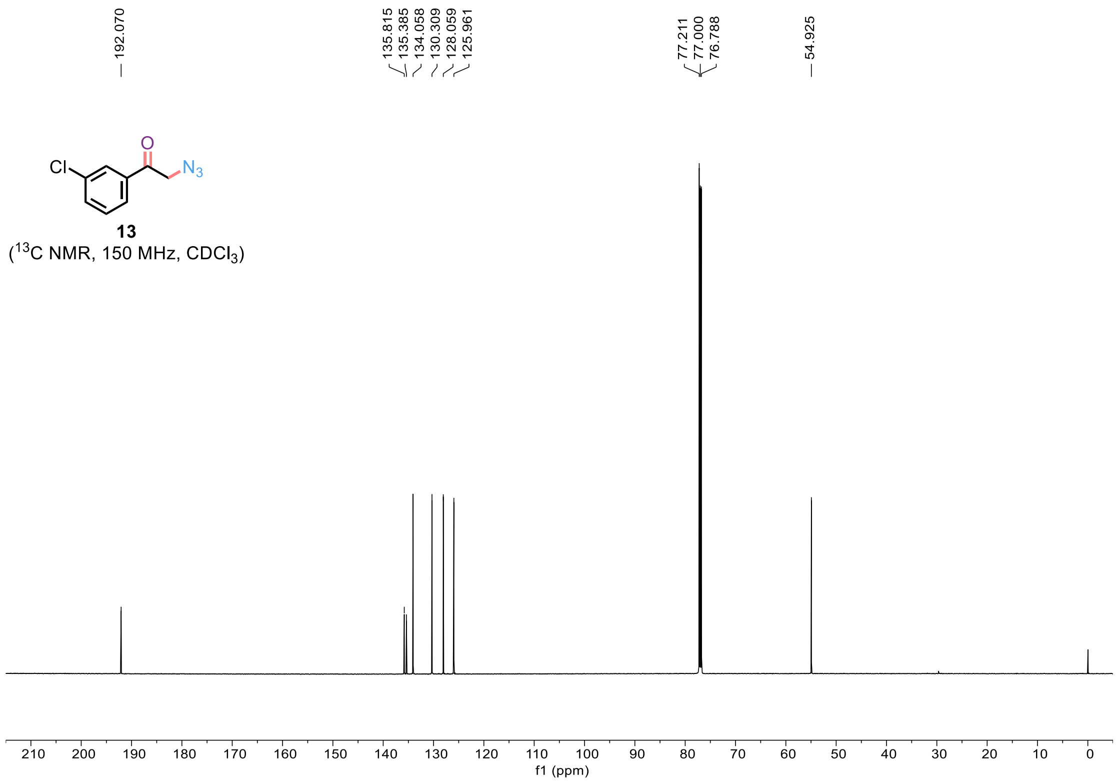

13
(^{13}C NMR, 150 MHz, $CDCl_3$)

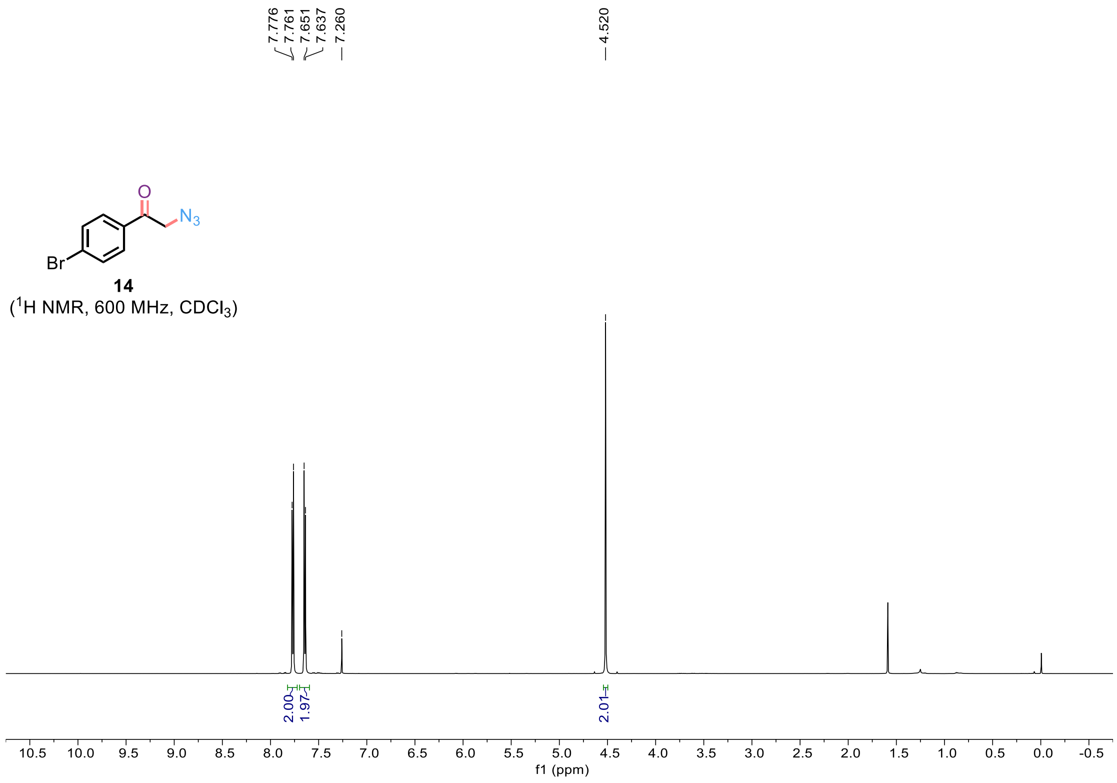
7.776
7.761
7.651
7.637
7.260
4.520
O
N3
Br
14
(1H NMR, 600 MHz, CDCl3)
2.00
1.97
2.01
10.5 10.0 9.5 9.0 8.5 8.0 7.5 7.0 6.5 6.0 5.5 5.0 4.5 4.0 3.5 3.0 2.5 2.0 1.5 1.0 0.5 0.0 -0.5
f1 (ppm)

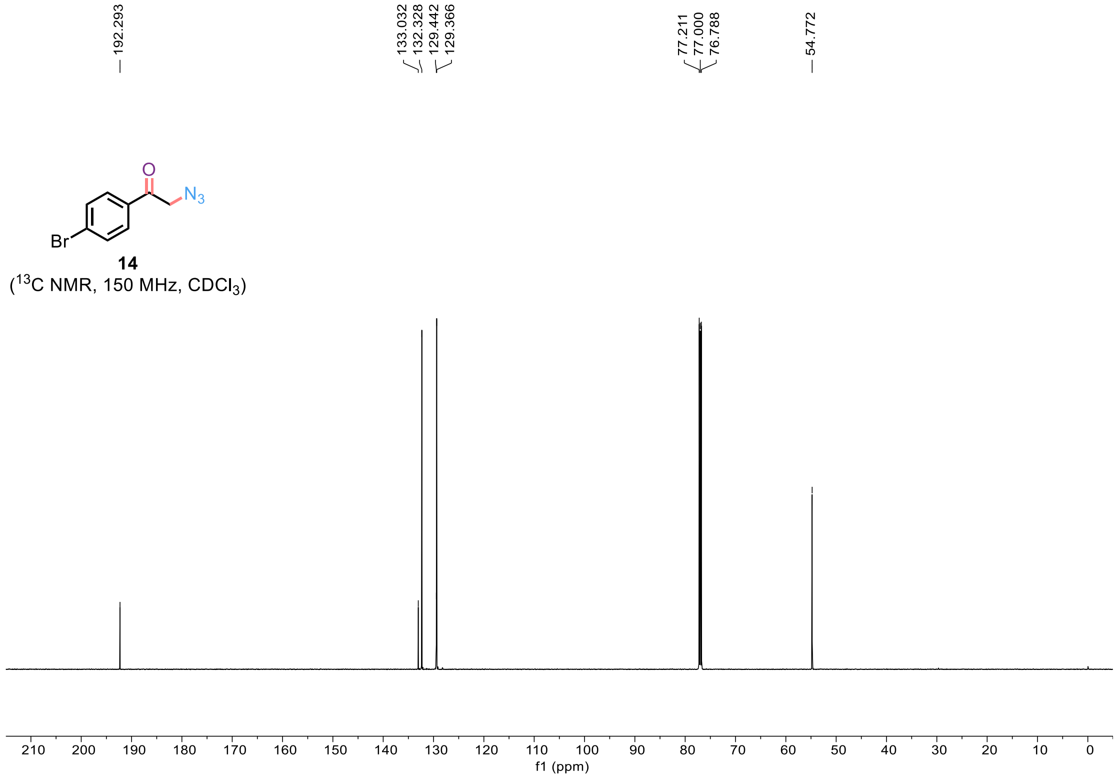
192.293
133.032
132.328
129.442
129.366
77.211
77.000
76.788
54.772
O
N3
Br
14
(13C NMR, 150 MHz, CDCl3)
210 200 190 180 170 160 150 140 130 120 110 100 90 80 70 60 50 40 30 20 10 0
f1 (ppm)

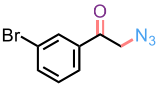

15
(^{1}H NMR, 600 MHz, $CDCl_3$)

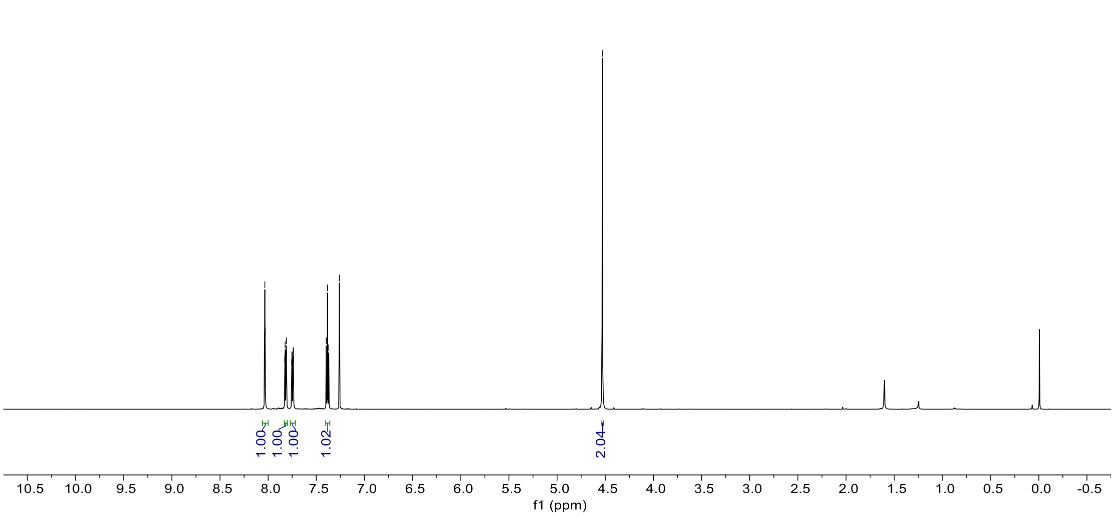

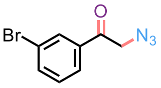

15
(^{13}C NMR, 150 MHz, $CDCl_3$)

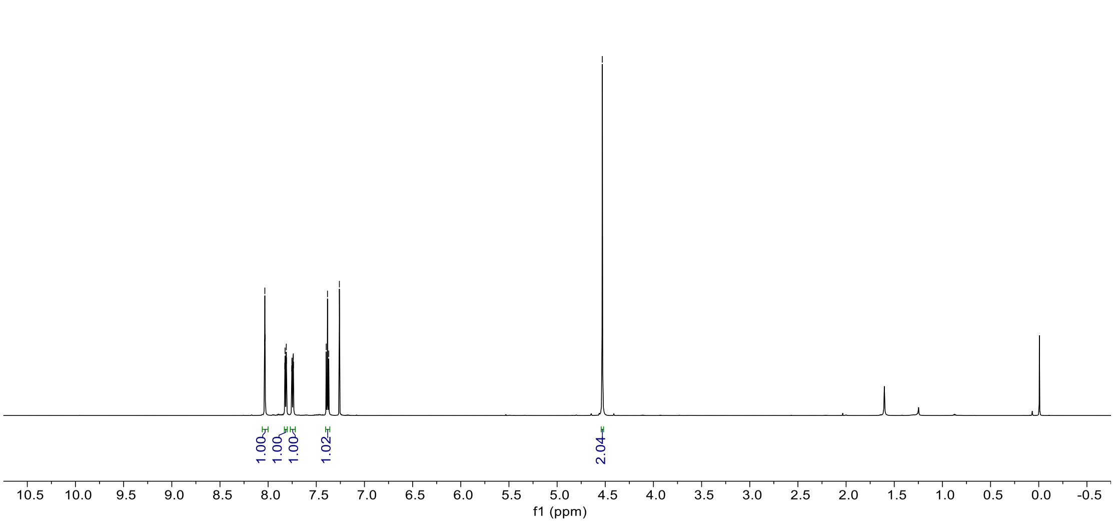

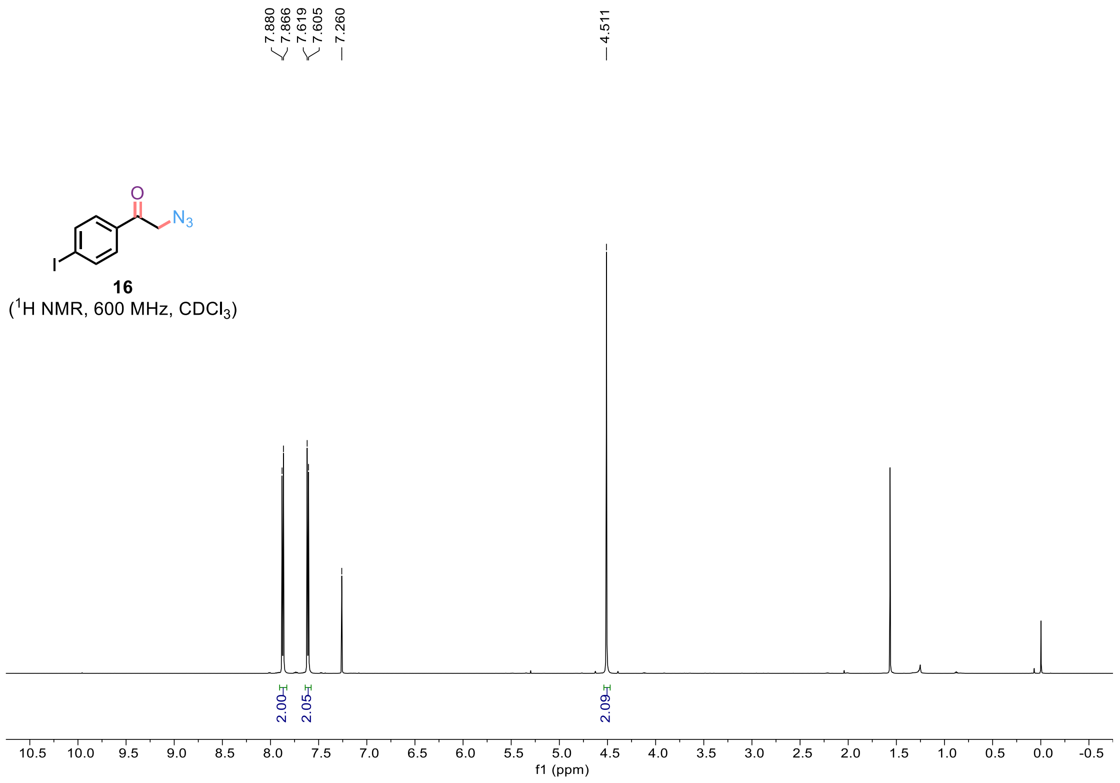
7.880
7.866
7.619
7.605
7.260
4.511
16
(¹H NMR, 600 MHz, CDCl₃)
2.00
2.05
2.09
10.5 10.0 9.5 9.0 8.5 8.0 7.5 7.0 6.5 6.0 5.5 5.0 4.5 4.0 3.5 3.0 2.5 2.0 1.5 1.0 0.5 0.0 -0.5
f1 (ppm)

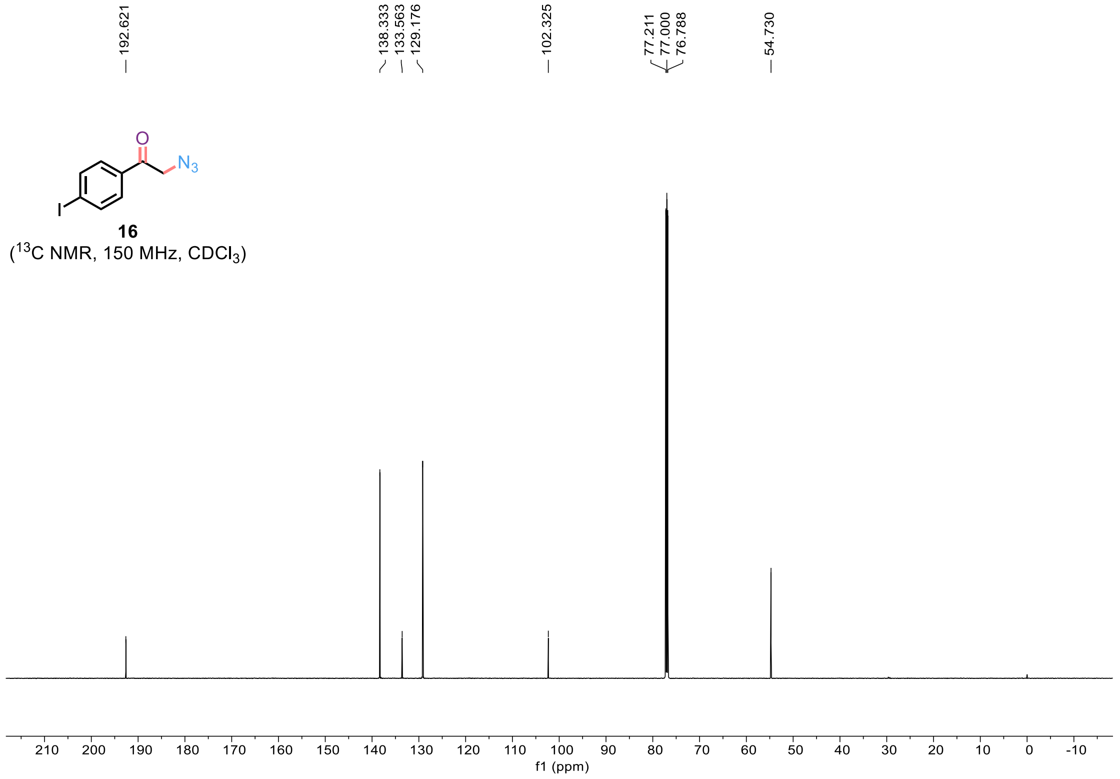
192.621
138.333
133.563
129.176
102.325
77.211
77.000
76.788
54.730
16
(¹³C NMR, 150 MHz, CDCl₃)
210 200 190 180 170 160 150 140 130 120 110 100 90 80 70 60 50 40 30 20 10 0 -10
f1 (ppm)

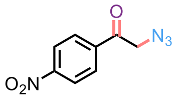

17
(^{1}H NMR, 600 MHz, $CDCl_3$)

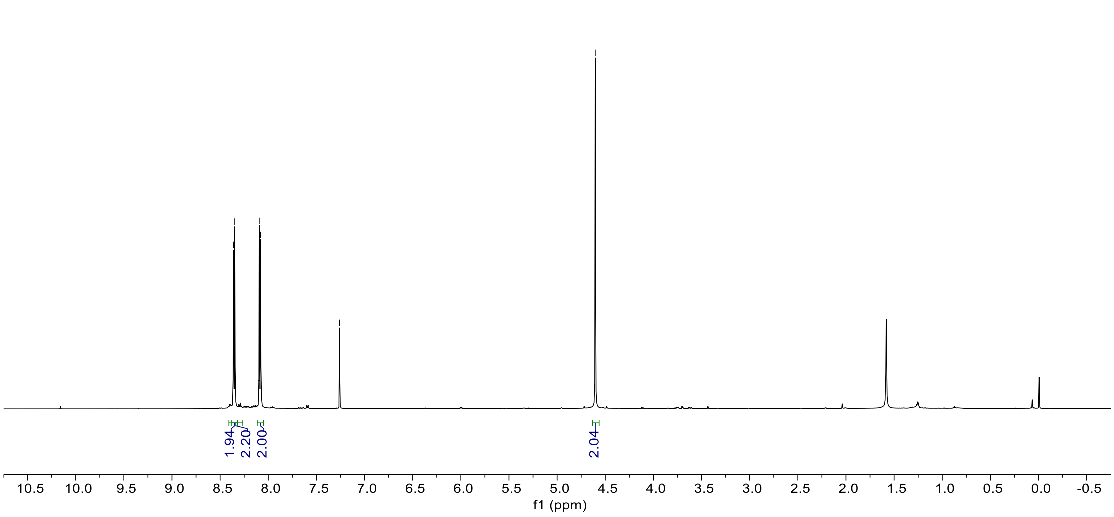

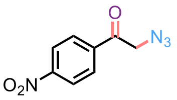

17
(^{13}C NMR, 150 MHz, $CDCl_3$)

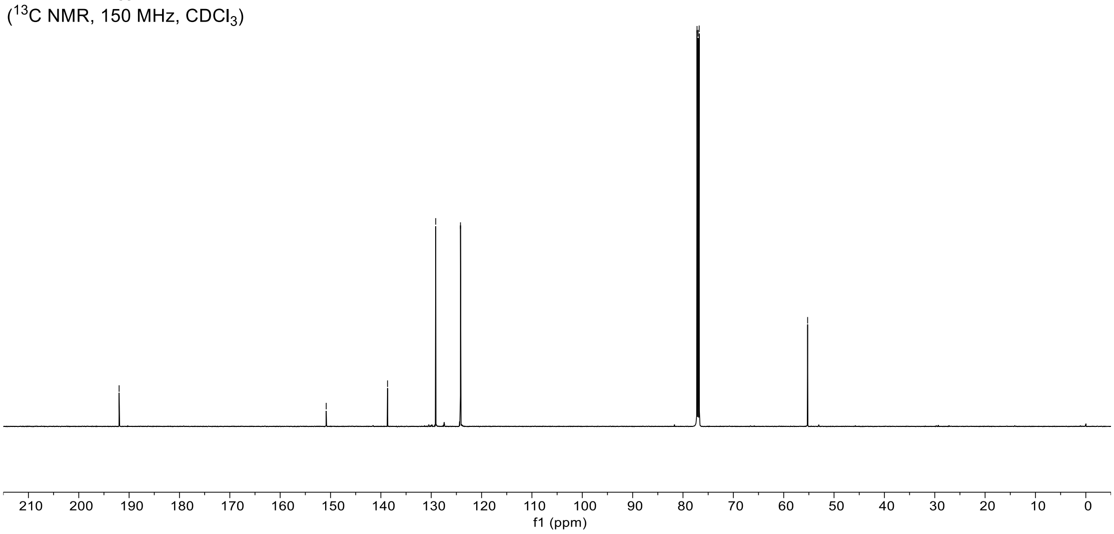

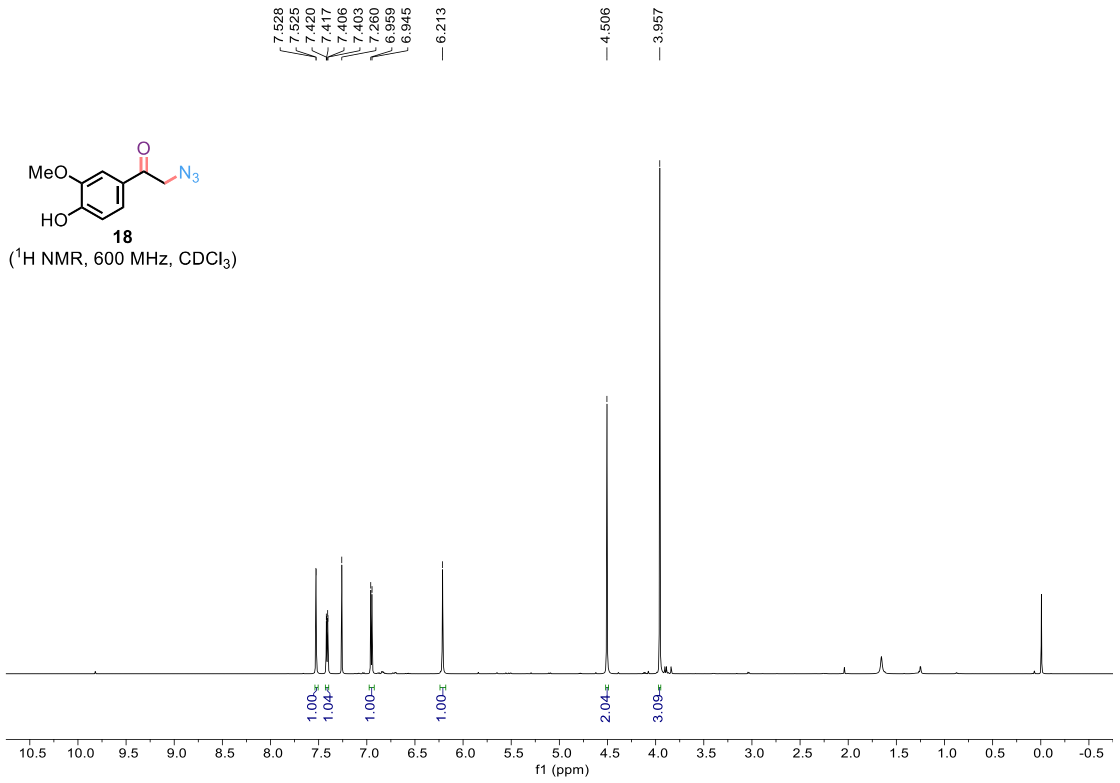
7.528
7.525
7.420
7.417
7.406
7.403
7.260
6.959
6.945
6.213
4.506
3.957
MeO
O
N3
HO
18
(1H NMR, 600 MHz, CDCl3)
1.00
1.04
1.00
1.00
2.04
3.09
10.5 10.0 9.5 9.0 8.5 8.0 7.5 7.0 6.5 6.0 5.5 5.0 4.5 4.0 3.5 3.0 2.5 2.0 1.5 1.0 0.5 0.0 -0.5
f1 (ppm)

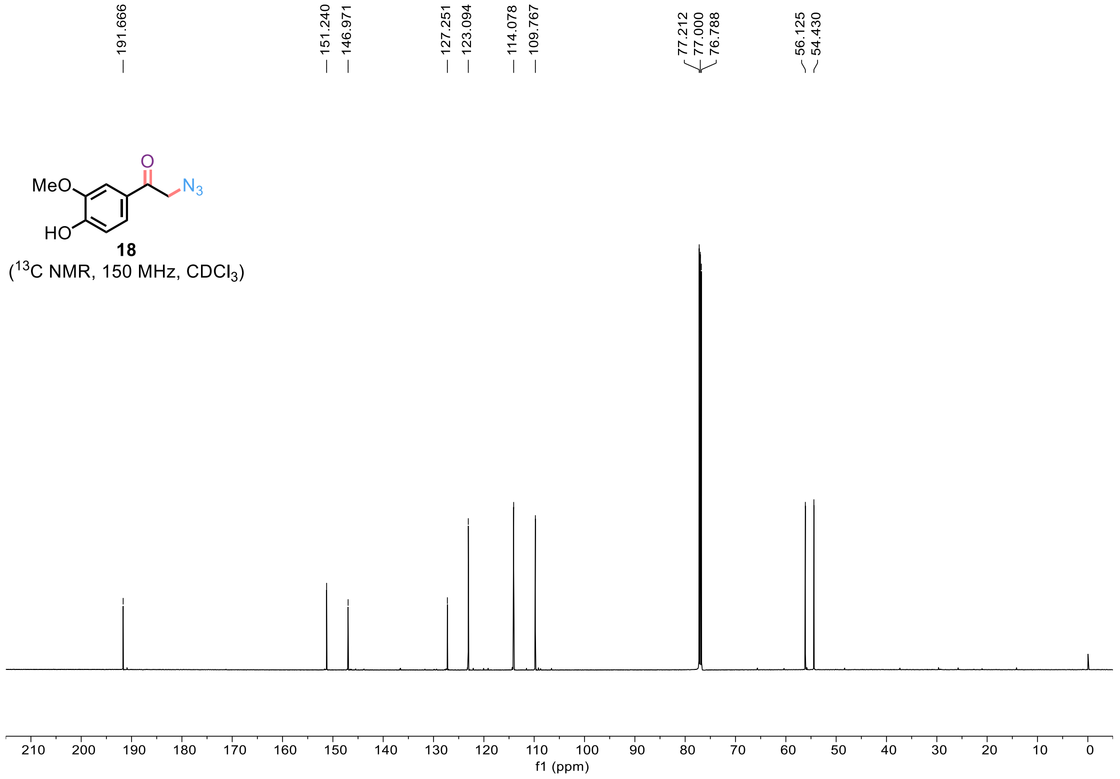
191.666
151.240
146.971
127.251
123.094
114.078
109.767
77.212
77.000
76.788
56.125
54.430
MeO
O
N3
HO
18
(13C NMR, 150 MHz, CDCl3)
210 200 190 180 170 160 150 140 130 120 110 100 90 80 70 60 50 40 30 20 10 0
f1 (ppm)

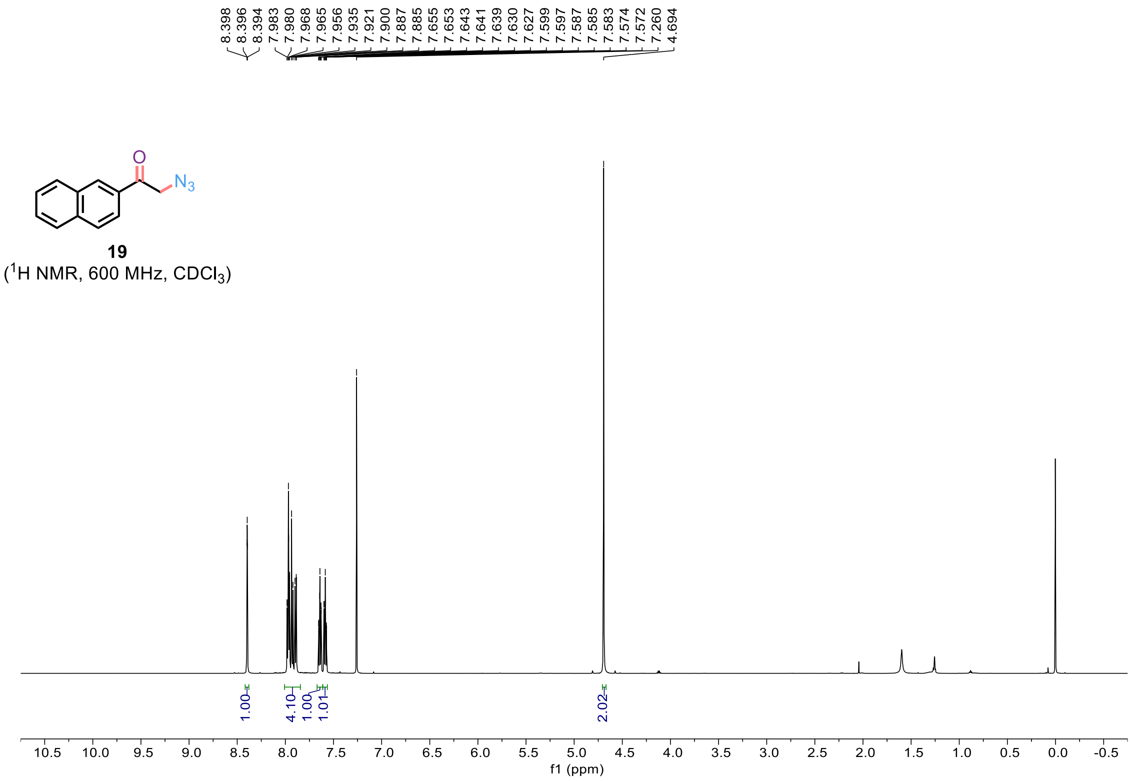
8.398
8.396
8.394
7.983
7.980
7.968
7.965
7.956
7.935
7.921
7.900
7.887
7.885
7.655
7.653
7.643
7.641
7.639
7.630
7.627
7.599
7.597
7.587
7.585
7.583
7.574
7.572
7.260
4.694
O
N3
19
(1H NMR, 600 MHz, CDCl3)
1.00
4.10
1.00
1.01
2.02
f1 (ppm)

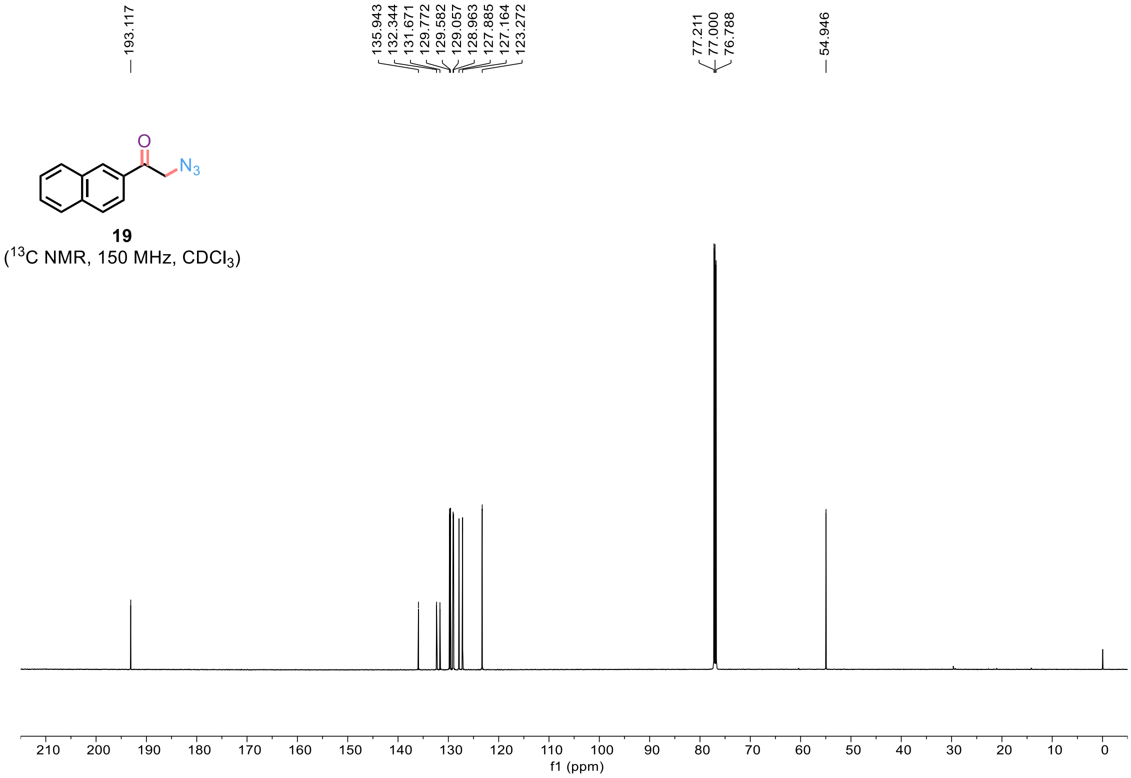
193.117
135.943
132.344
131.671
129.772
129.582
129.057
128.963
127.885
127.164
123.272
77.211
77.000
76.788
54.946
O
N3
19
(13C NMR, 150 MHz, CDCl3)
f1 (ppm)

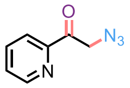

20
(^{1}H NMR, 600 MHz, $CDCl_3$)

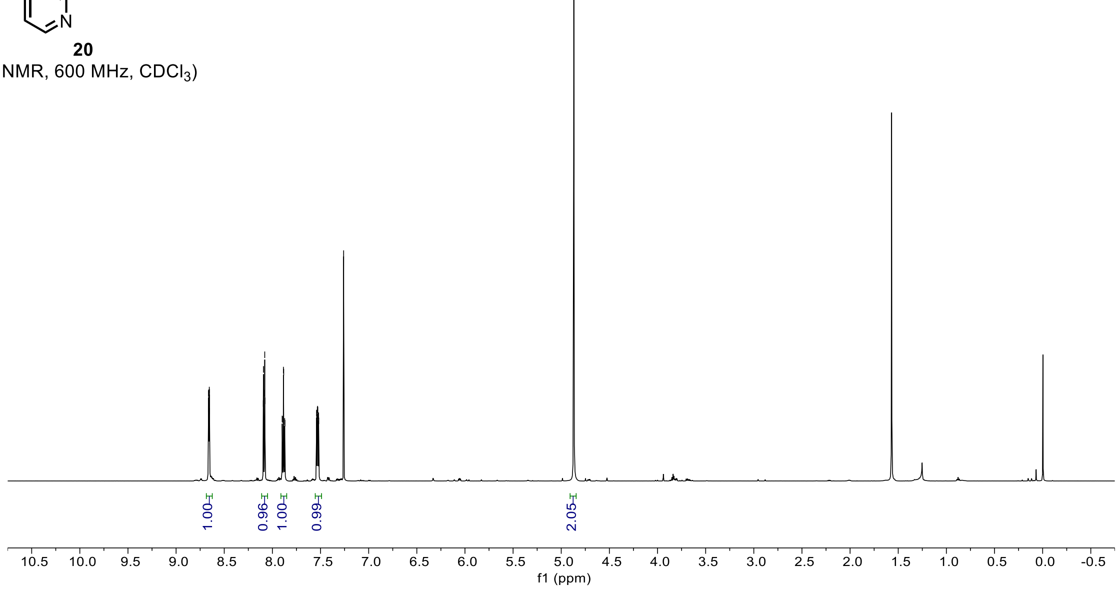

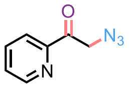

20
(^{13}C NMR, 150 MHz, $CDCl_3$)

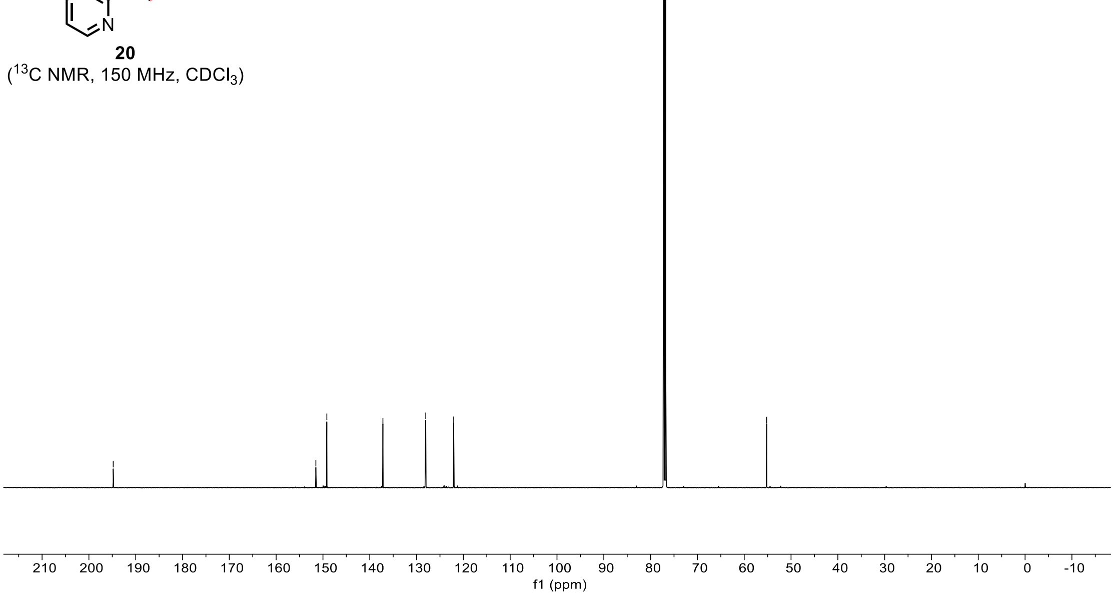

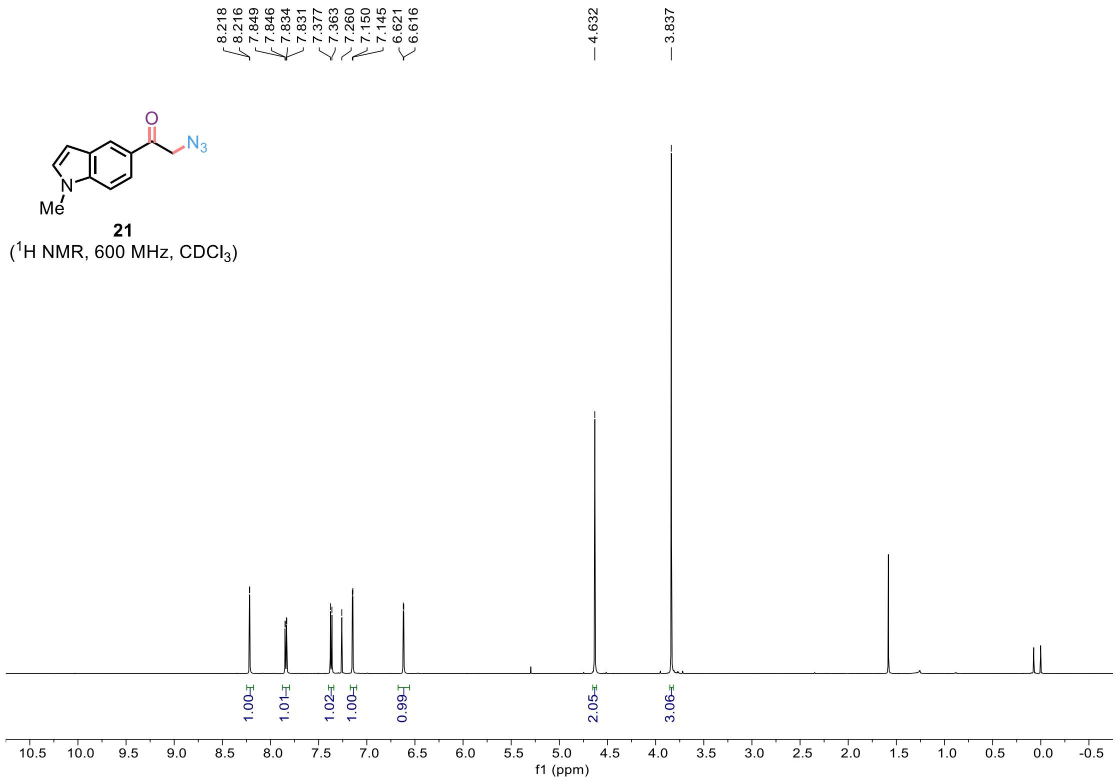

21
(^{1}H NMR, 600 MHz, $CDCl_3$)

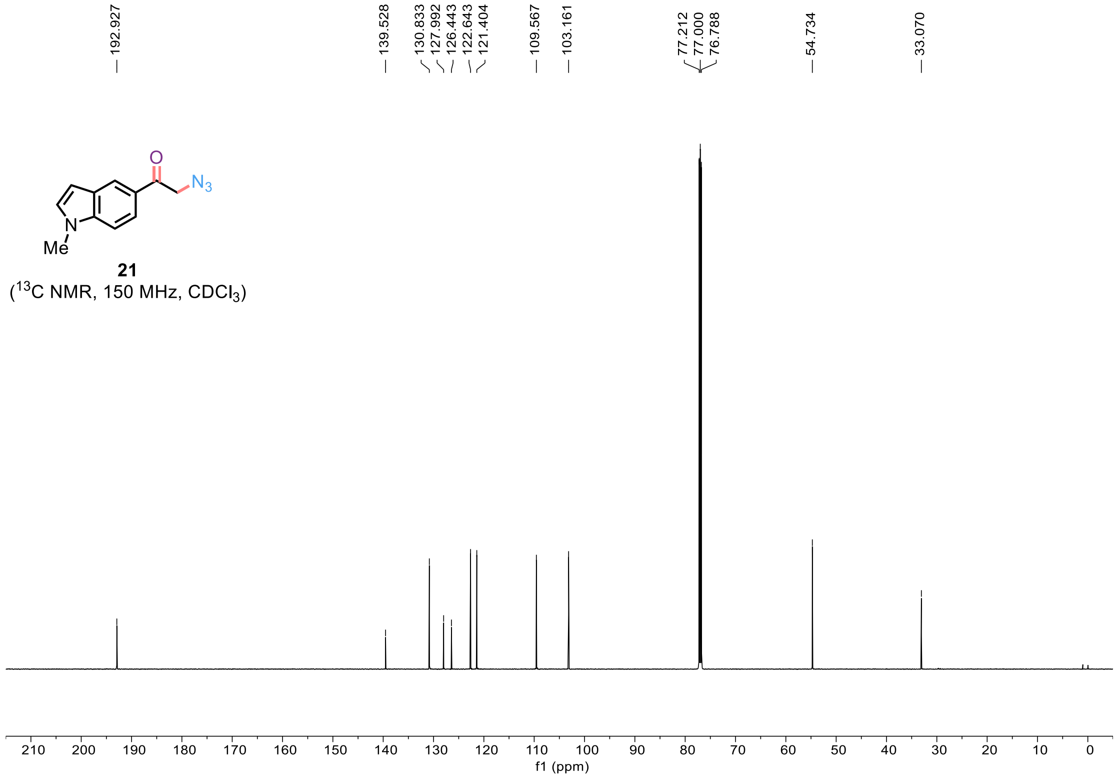

21
(^{13}C NMR, 150 MHz, $CDCl_3$)

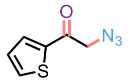

22
(^{1}H NMR, 600 MHz, $CDCl_3$)

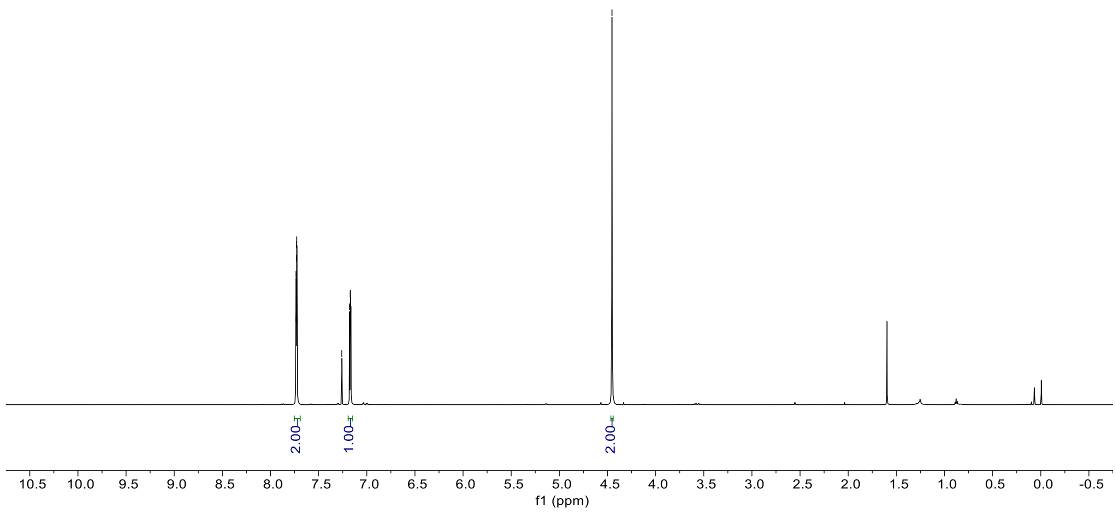

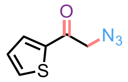

22
(^{13}C NMR, 150 MHz, $CDCl_3$)

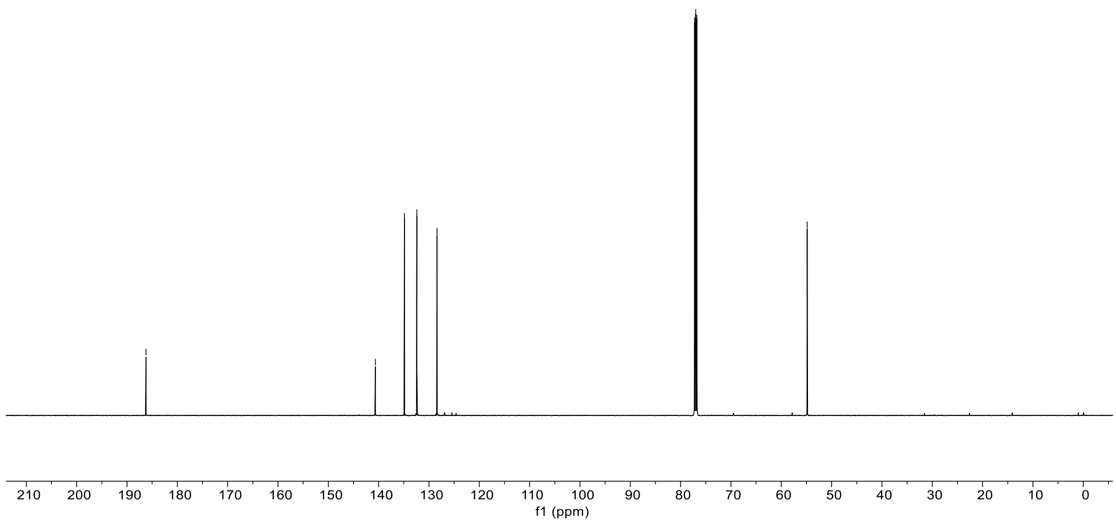

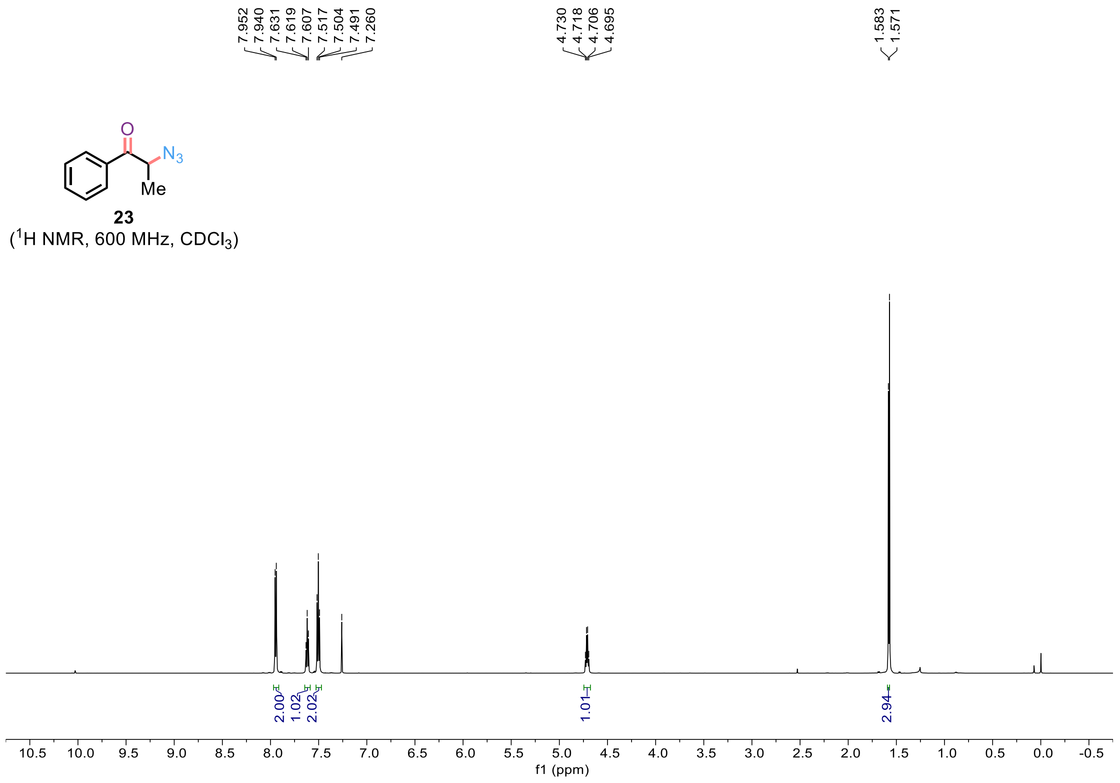
7.952
7.940
7.631
7.619
7.607
7.517
7.504
7.491
7.260
4.730
4.718
4.706
4.695
1.583
1.571
O
N3
Me
23
(1H NMR, 600 MHz, CDCl3)
2.00
1.02
2.02
1.01
2.94
10.5 10.0 9.5 9.0 8.5 8.0 7.5 7.0 6.5 6.0 5.5 5.0 4.5 4.0 3.5 3.0 2.5 2.0 1.5 1.0 0.5 0.0 -0.5
f1 (ppm)

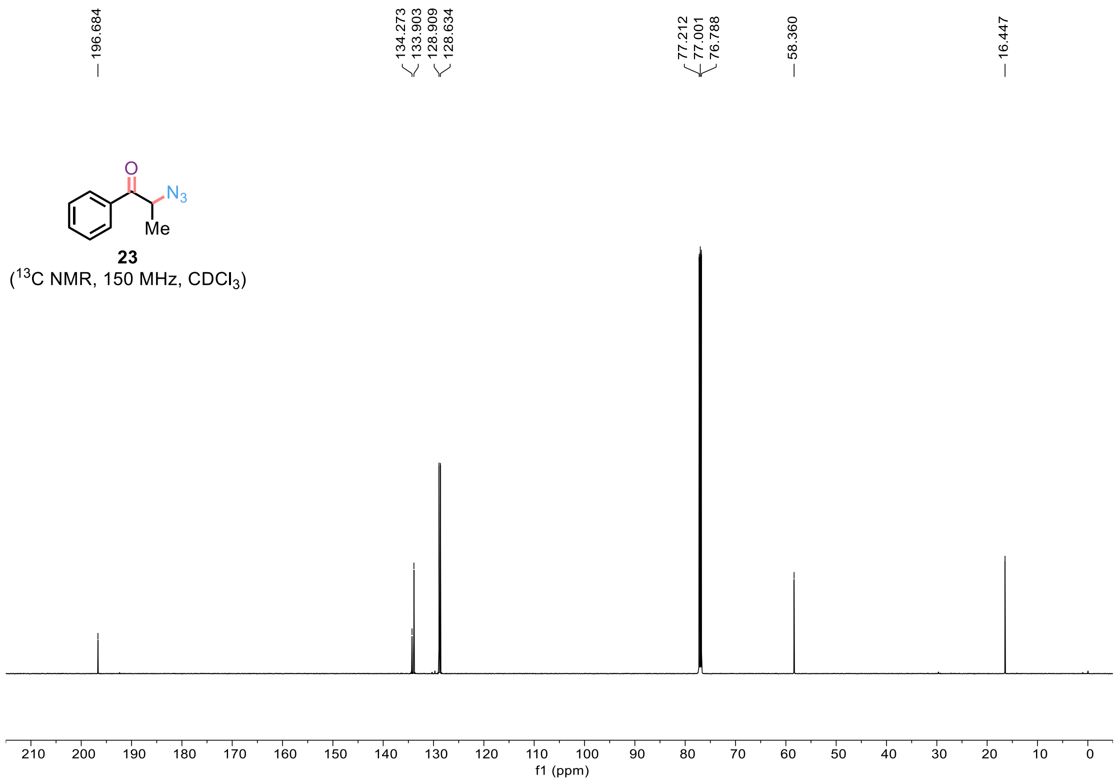
196.684
134.273
133.903
128.909
128.634
77.212
77.001
76.788
58.360
16.447
O
N3
Me
23
(13C NMR, 150 MHz, CDCl3)
210 200 190 180 170 160 150 140 130 120 110 100 90 80 70 60 50 40 30 20 10 0
f1 (ppm)

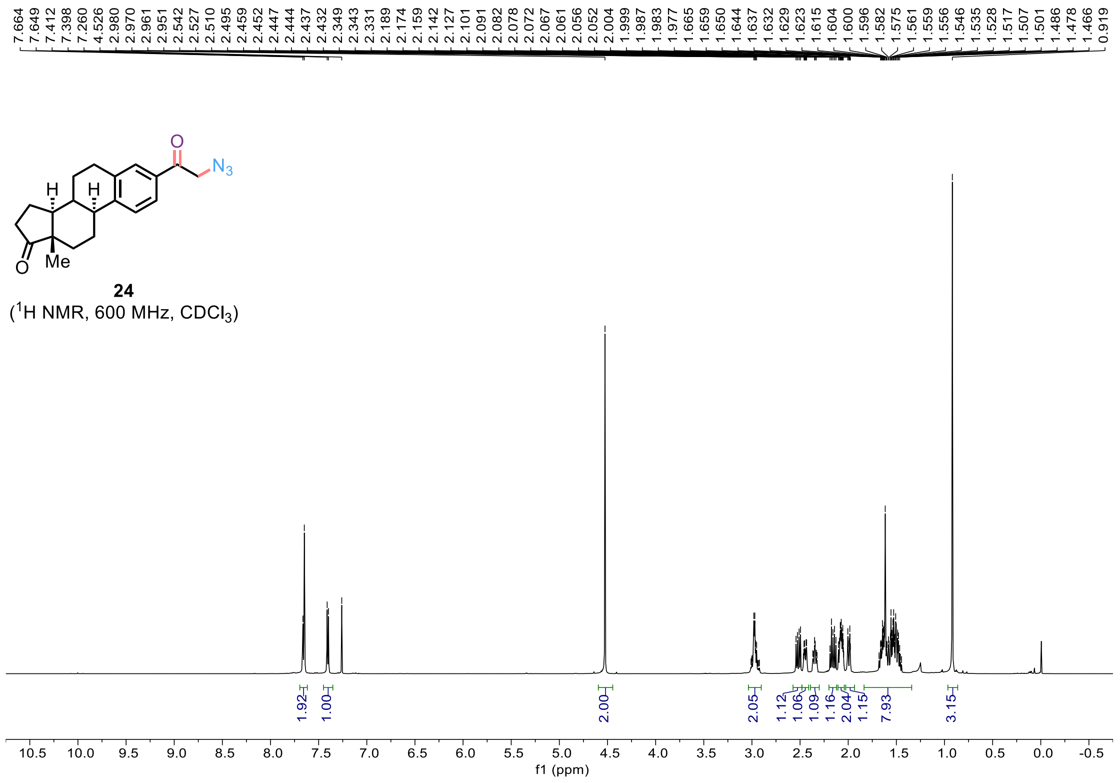
7.664 7.649 7.412 7.398 7.260 4.526 2.980 2.970 2.961 2.951 2.542 2.527 2.510 2.495 2.459 2.452 2.447 2.444 2.437 2.432 2.349 2.343 2.331 2.189 2.174 2.159 2.142 2.127 2.101 2.091 2.082 2.078 2.072 2.067 2.061 2.056 2.052 2.004 1.999 1.987 1.983 1.977 1.665 1.659 1.650 1.644 1.637 1.632 1.629 1.623 1.615 1.604 1.600 1.596 1.582 1.575 1.561 1.559 1.556 1.546 1.535 1.528 1.517 1.507 1.501 1.486 1.478 1.466 0.919
O
N3
H
H
O
Me
24
(1H NMR, 600 MHz, CDCl3)
1.92
1.00
2.00
2.05
1.12
1.06
1.09
1.16
2.04
1.15
7.93
3.15
10.5 10.0 9.5 9.0 8.5 8.0 7.5 7.0 6.5 6.0 5.5 5.0 4.5 4.0 3.5 3.0 2.5 2.0 1.5 1.0 0.5 0.0 -0.5
f1 (ppm)

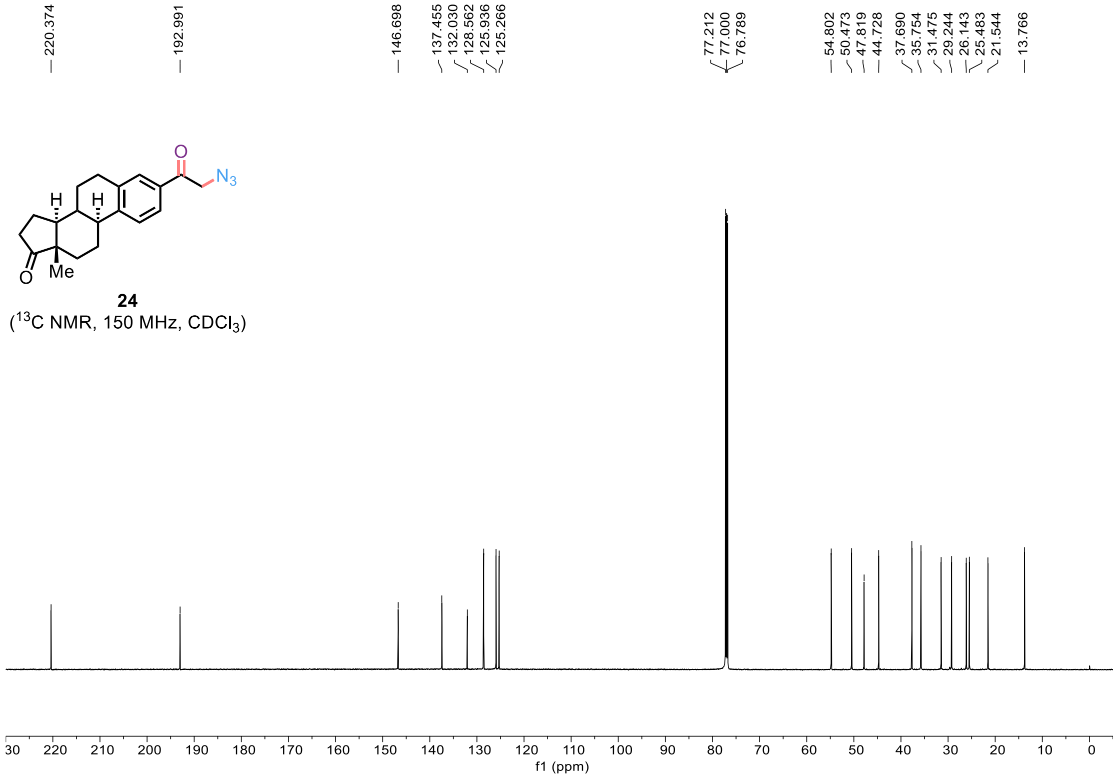
220.374
192.991
146.698
137.455 132.030 128.562 125.936 125.266
77.212 77.000 76.789
54.802 50.473 47.819 44.728 37.690 35.754 31.475 29.244 26.143 25.483 21.544
13.766
O
N3
H
H
O
Me
24
(13C NMR, 150 MHz, CDCl3)
30 220 210 200 190 180 170 160 150 140 130 120 110 100 90 80 70 60 50 40 30 20 10 0
f1 (ppm)

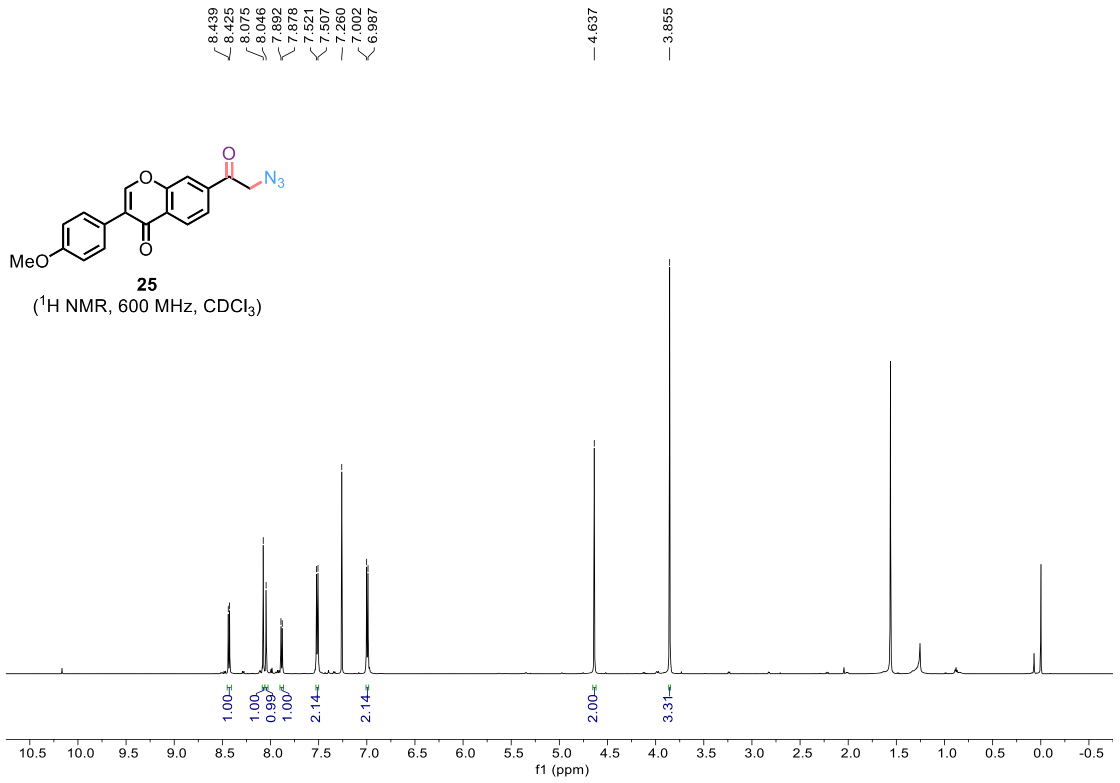
8.439
8.425
8.075
8.046
7.892
7.878
7.521
7.507
7.260
7.002
6.987
4.637
3.855
MeO
O
O
O
N3
25
(1H NMR, 600 MHz, CDCl3)
1.00
1.00
0.99
1.00
2.14
2.14
2.00
3.31
10.5 10.0 9.5 9.0 8.5 8.0 7.5 7.0 6.5 6.0 5.5 5.0 4.5 4.0 3.5 3.0 2.5 2.0 1.5 1.0 0.5 0.0 -0.5
f1 (ppm)

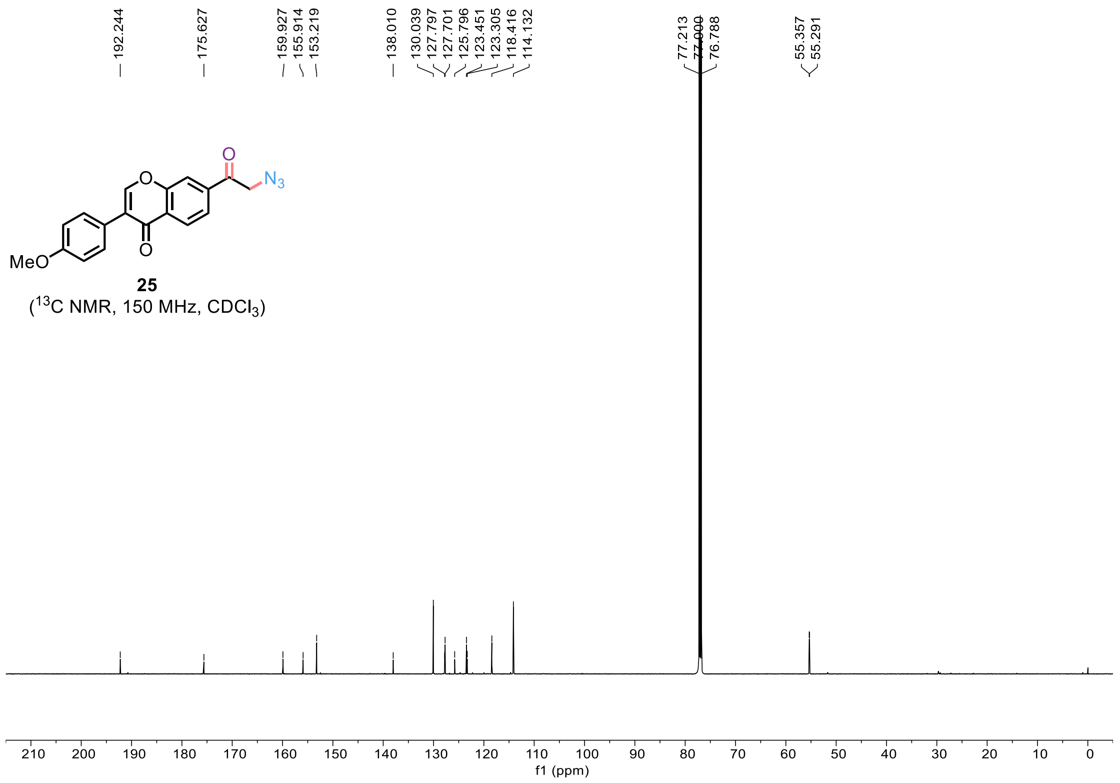
192.244
175.627
159.927
155.914
153.219
138.010
130.039
127.797
127.701
125.796
123.451
123.305
118.416
114.132
77.213
77.000
76.788
55.357
55.291
MeO
O
O
O
N3
25
(13C NMR, 150 MHz, CDCl3)
210 200 190 180 170 160 150 140 130 120 110 100 90 80 70 60 50 40 30 20 10 0
f1 (ppm)
